# Whole genome transcriptome polymorphisms in *Arabidopsis thaliana*

**DOI:** 10.1186/gb-2008-9-11-r165

**Published:** 2008-11-24

**Authors:** Xu Zhang, Jake K Byrnes, Thomas S Gal, Wen-Hsiung Li, Justin O Borevitz

**Affiliations:** 1 Department of Ecology and Evolution, University of Chicago, 1101 E. 57th Street, Chicago, IL 60637, USA

## Abstract

Whole genome tiling arrays are a key tool for profiling global genetic and expression variation. In this study we present our methods for detecting transcript level variation, splicing variation and allele specific expression in *Arabidopsis thaliana*. We also developed a generalized hidden Markov model for profiling transcribed fragment variation *de novo*. Our study demonstrates that whole genome tiling arrays are a powerful platform for dissecting natural transcriptome variation at multi-dimension and high resolution.

## Abstract 2

New methods for detecting global patterns of gene expression and splicing variation in natural Arabidopsis thaliana populations.

## Background

Natural gene expression variation represents perturbations in the cellular network underlying morphological and physiological diversity. It reveals altered signaling pathways that may include early events responsible for phenotypic variation. Gene expression phenotypes are complex traits that map to genetic loci acting in *cis *and/or *trans *[1-5]. *Trans*-acting loci affect expression of both alleles of the downstream gene, while *cis*-acting loci represent genetic polymorphisms in the regulatory elements causing allelic variation. *Cis*-regulatory variation and dosage effects of *trans *regulatory variation result in additivity of gene expression, with the expression level of F1 hybrids being intermediate to that of parents. Allele specific expression (ASE) in heterozygous individuals, which directly measures *cis *variation, is common in human [6-8], *Arabidopsis *[9] and maize [10,11]. Nonadditivity of gene expression, where the expression level of F1 hybrids deviates from the midpoint of the parental expression levels, indicates dominant *trans *regulatory variation, novel combinations of *trans *regulatory factors and/or *cis *× *trans *interaction. Additivity of gene expression has been tested globally in a few diploid organisms, including *Drosophila *[12-14], mouse [15,16], maize [11,17] and *Arabidopsis *[18]. Regulatory effects of *trans *variation and *cis *× *trans *interaction could depend on environmental conditions or developmental stages, which contribute to natural variation of gene expression plasticity.

In eukaryotic organisms, transcriptome variation may result from quantitative as well as structural differences of the transcripts. Eukaryotic genes are initially transcribed as pre-messenger RNA (pre-mRNA). The excision of introns and ligation of exons is mediated by the spliceosome, a ribonucleoprotein complex containing small nuclear RNAs and associated proteins [19,20]. Alternative combinations of exons allow a single gene to produce a variety of transcript isoforms. This diversifying process, also known as alternative splicing, is a common phenomenon in eukaryotic organisms [21]. Alternative splicing could generate mRNAs with different stability [22] or different cellular localization [23,24], and proteins with distinct functions [25]. The regulation of both constitutive and alternative splicing involves auxiliary elements and a variety of splicing factors [26-28]. The splicing process could be substantially different between animals and plants, especially in the early splicing site recognition steps [29,30]. Exon skipping is a predominant form of alternative splicing in animals, while alternative intron retention is frequently observed in plant genes [30-32].

Microarrays provide a comprehensive platform for the study of natural transcriptome variation between closely related genomes. Gene expression arrays and exon arrays, on which each annotated gene or exon is interrogated by approximately the same number of probes, have been widely used in gene expression studies [33]. The genomic coverage of these arrays is limited, however, by the completeness of annotation. On the other hand, a popular microarray design for detecting alternative splicing features oligonucleotide probes that cover exon-exon junctions of spliced transcripts [34,35]. Again, these arrays aim to investigate known alternative splicing events [36]. Whole genome tiling arrays cover the entire genome with high density oligonucleotide probes, independent of any prior knowledge of transcripts [37,38]. Gene expression is assayed across the complete gene while splicing variation can be indirectly assessed as alternative expression within the gene. The tiling array design also allows a *de novo *transcriptome profiling, by revealing new transcribed fragments, gene boundaries, and novel splicing forms. In this study, we report the natural variation in transcript level and splicing between two *A. thaliana *accessions, Columbia (Col) and Vancouver (Van), using the Affymetrix whole genome tiling array, which contains approximately 1.6 million unique features at a 35 base resolution. Using a quantitative genetics model, we dissect additive, dominance and maternal expression variation among parental and reciprocal hybrid genotypes for annotated gene/exon/intron. We also take an unbiased approach to infer differentially expressed fragments independent of the annotation. These analyses have revealed global patterns of gene expression and splicing variation between natural *A. thaliana *populations.

## Results

### Genome wide sequence polymorphisms

Natural variation in gene expression, as read out by hybridization differences on a microarray, is due to both true gene expression differences and genetic hybridization polymorphisms. The effect of single feature polymorphisms (SFPs) [39] can be significant when the analyzed unit is interrogated by only a small number of probes, or when the locus has a high level of genetic variation [40]. Copy number polymorphisms, reflected as continuous SFPs interrupted by signals from low quality probes, are an additional source of genetic polymorphisms interfering with expression analysis. In this study we did parallel hybridizations of genomic DNA and cDNA samples to the *Arabidopsis *tiling array 1.0 F (Affymetrix, Santa Clara, California, USA). Four maternal seed batch replicates (Materials and methods) were included for each of the two *A. thaliana *strains, Col and Van.

**Figure 1:**
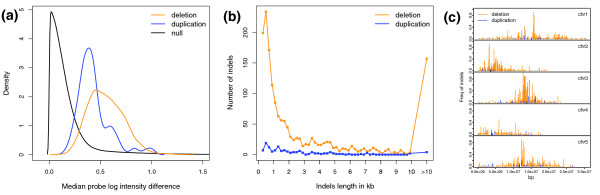
The deletions (orange) and duplications (blue) detected in Van. **(a) **The density distribution of median probe log intensity difference between Col and Van for deletions, duplications, and all analyzed probes (black). For each probe the absolute difference of mean probe log intensity between four Col replicates and four Van replicates was calculated. The medians were then obtained across deleted or duplicated regions. **(b) **The length distribution of deleted and duplicated regions. **(c) **The chromosome distribution of deleted and duplicated regions. Each chromosome was divided into 100 kb bins. Within each bin the length of deletions or duplications was divided by the bin size (y-axis). The black ticks along each chromosome mark the position of centromeres.

We first identified the SFPs between Col and Van as described previously [39]. A total of 125,043 SFPs were detected at a 5% false discovery rate (FDR; Table S1A in Additional data file 2). As the reference genotype of the 1.0 F array is Col, the 118,381 SFPs with a greater signal in Col can be located to the exact chromosome positions. The remaining 6,662 SFPs with a greater signal in Van are likely due to duplications in Van or insertions that cross-hybridize. To identify large (>200 bp) deletions and duplications in Van relative to Col, we applied a segmentation algorithm on the genomic hybridization intensities [41]. The probe-level data used here were *p*-values collected from one-sided two sample *t*-tests for the alternative hypothesis H1: μVan > μCol. Using the Akaike Information Criterion, segment boundaries for each of the five chromosomes were identified. We then defined deletions and duplications as segments with a median *p*-value > 0.99 and a median *p*-value < 0.02, respectively. Non-symmetric *p*-value cutoffs were used as the probe intensity differences of duplicated regions were generally less than that of deleted regions (Figure 1a). This is because at log scale, about one unit of signal increase is observed across duplications, while several units of signal decrease are seen for deletions. A total of 1,645 deletions and 136 duplications were detected in Van (Table S1B in Additional data file 2). The distribution of the length of indels centered around 500 bp while a few very large deletions were also detected (Figure 1b). Examination of the distribution of indels in 100-kb bins along the chromosome suggests that they tend to accumulate in the pericentromeric region (Figure 1c). Here, Van duplications were presented on the Col physical map, although they may not be tandem duplications and could map elsewhere. Interestingly, genes present in Col but deleted in Van are expressed at lower absolute levels in Col when compared with randomly sampled gene sets (Figure S1 in Additional data file 1), probably because gene expression levels are inversely correlated with their distance to the centromeres.

### Natural variation of gene expression

A total of 29,409 annotated genes are interrogated by the 1.0 F array, with exon and intron boundaries inferred from expressed clone sequences or computational prediction. Before performing gene expression analysis, the low quality probes and probes interrogating sequence polymorphisms detected from genomic hybridizations were removed from RNA hybridization data. Importantly, as overall gene expression level was estimated as the average across common exons, exon probes were defined as probes interrogating gene sequences that are present in ≥50% expressed sequence clones. Under this constraint, a total of 24,756 genes interrogated by 625,240 exon probes were then analyzed, with a mean density of 25 probes per gene (Figure S2A in Additional data file 1).

In quantitative genetics terminology, additivity of gene expression implies that the expression level of a given locus in F1 hybrids is approximately at the midpoint of that of the parental lines (henceforth called the 'mid-parent'), while dominance of gene expression indicates that expression of F1 hybrids deviates from the mid-parent (Figure S2B in Additional data file 1). In addition, maternally inherited *trans *regulatory factors or epigenetic mechanisms may cause gene expression levels that are correlated with maternal genotypes (Figure S2B in Additional data file 1). To jointly test for these effects, for each gene we applied a linear model:

Intensity = Additive + Dominant + Maternal + Error

The additive, dominant and maternal terms in the model measure the expression difference between parents (additive), between the average of F1s and the mid-parent (dominant), and between reciprocal F1s (maternal), respectively. For each term, a *d *score (Materials and methods) was obtained for each gene and a permutation based approach was applied to determine the FDR [42]. Because the null *d *score distributions of the additive, dominant and maternal terms were essentially identical (Figure S2C in Additional data file 1), we applied the same threshold to call significance for the three terms (Table S2A in Additional data file 2).

**Figure 2:**
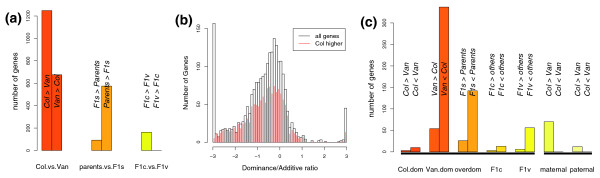
The additive, dominant and maternal effects of gene expression. **(a) **The number of genes (y-axis) significant for additive (left), dominant (middle), or maternal (right) terms. From left to right, the bars represent Col > Van, Van > Col, F1s > parents, parents > F1s, Col mother F1 > Van mother F1, Van mother F1 > Col mother F1. **(b) **Histogram of the dominance/additive ratio (x-axis) for 1,925 genes differentially expressed between Col and Van at a 2% FDR. The red lines represent the number of genes up-regulated in Col. **(c) **The inheritance pattern determined by partition of gene expression means by k-means, for genes significant for dominant (left block) or maternal (right block) terms. From left to right, the bars represent the number of genes (y-axis) showing Col dominance (Col > Van, Col < Van), Van dominance (Van > Col, Van < Col), over-dominance (F1s > parents, F1s < parents), Col mother F1 separated from the other three strains (Col mother F1 > others, Col mother F1 < others), Van mother F1 separated from the other three strains (Van mother F1 > others, Van mother F1 < others), maternal effect (Col > Van, Col < Van), and paternal effect (Col >Van, Col < Van).

Nearly 8% (1,925) of the analyzed genes were differentially expressed between Col and Van at a 2% FDR, two-thirds (1,249) of which were up-regulated in Col. About 3% (667) of genes were differentially expressed between parents and F1 hybrids at a 6% FDR, the majority (575) being repressed in the hybrids. Less than 1% (163) of genes were differentially expressed between reciprocal F1 hybrids at a 17% FDR, all of which were down-regulated in the Van-mother hybrids (Figure 2a). For the 1,925 genes differentially expressed between Col and Van, the ratio of the estimated effect of the dominant term to that of the additive term (d/a) exhibited a left-skewed normal distribution (Figure 2b), indicating dominant effects from the Van line. Genes down-regulated in Van tended to be repressed in the F1s, while a small number of genes up-regulated in Van were highly expressed in the hybrids (Figure 2b).

To examine the relative expression difference among Col, Van, and the reciprocal hybrids for genes significant for dominant or maternal terms, we partitioned the four genotypes into two groups based on their expression mean by k-means clustering (Figure 2c; Table S2B in Additional data file 2). For 667 genes differentially expressed between parents and F1 hybrids, 61% (404) exhibited normal dominance with hybrids clustering with a single parent, 25% (168) showed over-dominance, of which 142 were repressed in F1 hybrids, and 12% (78) had one F1 hybrid strain clustered separately from the other three strains. For 163 genes differentially expressed between reciprocal F1 hybrids, 70 correlated with the maternal genotype and 12 with the paternal genotype. Again, we observed strong dominant negative effects from the Van line.

The enrichment of differential gene expression in functional annotation categories was examined with a parametric gene set enrichment analysis [43] using the *d *scores for each term as summary statistics (Table S3 in Additional data file 2). We found that chlorophyll biosynthetic process, response to salt stress, response to cadmium ion, response to abscisic acid stimulus and sterol biosynthetic process were up-regulated in the Col line, while flavonoid biosynthetic process and translation were up-regulated in the Van line. An interesting pattern emerged when gene set enrichment analysis was performed for the dominant term: a large number of growth-related biological processes were suppressed while defense response pathways were up-regulated in F1 hybrids compared with those in parental lines.

### *Cis*-regulatory variation revealed by allele specific expression

Gene expression additivity could be caused by a *cis *difference or an additive *trans *difference. Direct measurement of ASE in F1 hybrids provides one approach to detect *cis *variation. The RNA hybridization intensities of SFP probes in transcribed regions reflect the overall transcript level as well as the allelic composition of that transcript (Figure S3A in Additional data file 1). To correct for gene expression variation, for each gene we estimated the fold differences in expression level using non-SFP probes, which were then subtracted from the log intensities of SFP probes. Our detection of ASE relies on a linear assumption that the binding coefficients of SFP probes for both perfect match targets and mismatch targets are constant across concentrations [44]. This implies that the mid-parent value (equal allele expression) could be estimated using genomic hybridization of F1 hybrids as reference (Materials and methods). ASE was thus detected as the deviation of log intensities of F1 hybrids from that of mid-parent for the SFP probes within the transcript. We applied a simple linear regression to test this, as the log intensity distribution of mid-parent and F1 hybrids was close to a normal distribution with stable variance (Figure S3B in Additional data file 1).

When a single threshold was applied to call significant ASE genes, a larger number of Van-ASE genes than Col-ASE genes was called. Further examination revealed that the log intensities of SFP probes for many of these Van-ASE genes were distributed at the low end (Figure S3C in Additional data file 1), suggesting possible overestimation of mid-parent values at low target concentrations. This could be addressed by excluding from analysis genes with low expression levels [44] or by applying a more stringent threshold to select Van-ASE genes with external FDR calibration [44]. At a 0.1% FDR determined by permutation analysis, a total of 209 Van-ASE genes were called significant (Table S4A in Additional data file 2), from which we randomly selected two for experimental validation and confirmed one (Table S4B in Additional data file 2). This means that the real FDR for the 209 Van-ASE genes could be 50%. Thus, the threshold appeared to be appropriate for calling Van-ASE genes.

**Figure 3:**
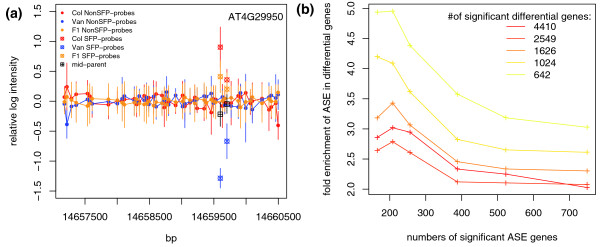
Detection of ASE in F1 hybrids. **(a) **Col ASE for gene AT4G29950. After correction of overall gene expression level, the relative log intensity (y-axis) for Col (red), Van (blue), F1 hybrids (orange), and mid-parent (black) were plotted along chromosomal positions (x-axis), with standard deviation indicated. Solid dots, non-SFP probes; crossed circles, SFP probes. **(b) **Fold enrichments of significant ASE genes within the differential genes between Col and Van. The numbers of significant calls were selected according to permutation-based FDRs.

Among 9,745 genes analyzed, 540 genes showed Col allele specific expression at a 1% FDR (Table S4A in Additional data file 2). An example of ASE genes is presented in Figure 3a. Since ASE genes contain *cis*-regulatory variations, many of them should exhibit differential expression between parental lines. We thus estimated the fold enrichment of the ASE genes in the set of differentially expressed genes between Col and Van. As expected, increasing the significance threshold for either differential expression or ASE increased the fold enrichment (Figure 3b). The ASE genes were especially enriched within differential genes of high statistical significance; the 749 ASE genes were enriched in the top 642 differential genes by more than three-fold (Figure 3b).

Under the linear assumption, a more straightforward approach to estimate the mid-parent value is to use the average of SFP probe intensities of parental RNA hybridizations (Materials and methods). This approach performed poorly, however, in comparison with that using genomic hybridization as reference. Only 30 genes were called significant at a 34% FDR (Table S4C in Additional data file 2), 25 of which overlapped the 749 ASE genes detected by using genomic hybridization as reference.

Allelic difference between reciprocal F1 hybrids, resulting from genomic imprinting, has been identified for several genes in *A. thaliana *endosperms [45]. Using the corrected SFP probe intensities, allelic differences between reciprocal F1 hybrids can be estimated. No significant imprinting effect was detected, however, in our 3-day-old seedling samples (data not shown).

### Natural variation of splicing

We next examined splicing variation between Col and Van. Since the majority of exons were expressed in our whole seedling mRNA preparations (Figure S4A in Additional data file 1), their hybridization intensities depended on overall transcript abundance as well as possible splicing variation that would modify a particular exon expression level. Thus, overall gene expression variation should be corrected for before testing for exon differences. Such a correction, however, shrinks the difference between two differentially spliced exons while simultaneously introduces a difference for the other exons in the same gene, since the overall gene expression level is underestimated in the presence of a skipped exon. Exons interrogated by >25% of the total gene probes were excluded from analysis, as they showed no enrichment for significant calls compared with null distribution, likely due to their large correlation with gene expression estimates (data not shown). A total of 68,022 exons for 15,349 genes were analyzed with a mean density of 3.7 probes per exon (Figure S4B in Additional data file 1). For each exon, probe intensities corrected by either mean gene expression or median-polished gene expression were tested with a linear model including additive, dominant and maternal terms. As an alternative approach to the probe level analysis, splicing indices [34] were also tested for the same 68,022 exons (Materials and methods).

**Figure 4:**
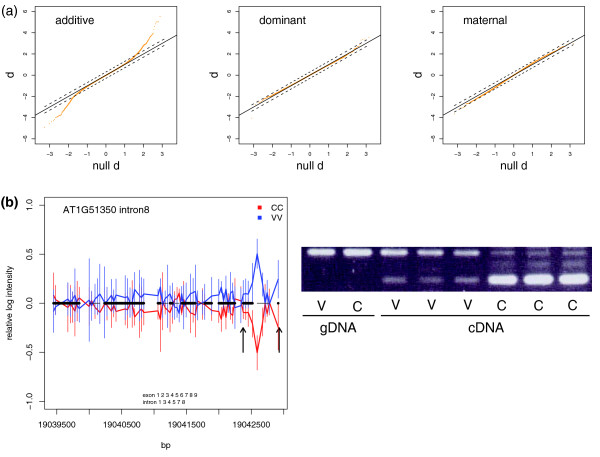
The additive, dominant and maternal effects of splicing. **(a) **Quantile-quantile plots of additive (left), dominant (middle) and maternal (right) terms for exonic splicing. The real *d *scores (y-axis) were plotted against the null *d *scores (x-axis) obtained by 1,000 permutations. **(b) **Experimental validation for AT1G51350 intron 8. The relative log intensity (y-axis) of Col (red) and Van (blue) was plotted along chromosomal positions (x-axis), with standard deviation indicated. Annotated exons and introns are indicated as thick and thin black horizontal bars, respectively, at y = 0. The arrows point to the start positions (along the forward strand) of the pair of flanking primers. Gel patterns show from left to right: Van (V) and Col (C) genomic DNA (gDNA), and three replicates of Van and Col cDNA.

Using probe intensities corrected by gene mean, only 0.35% (236) of the 68,022 analyzed exons were called significant for differential splicing between Col and Van at a FDR of 41% (Table 1). Using probe intensities corrected by gene median, 0.34% (230) of the analyzed exons were called significant at a 24% FDR while 0.74% (500) could be called at a higher FDR of 41% (Table 1). As the exons analyzed here were interrogated by ≤25% of total gene probes, estimation of gene median expression would be less affected by alternatively spliced probes. Using splicing indices, 0.38% (258) of the analyzed exons were called significant at a 4.6% FDR while 0.71% (482) were called at an 18% FDR (Table 1). Based on these different analyses, we expected that the top approximately 0.7% of exons contained true positives. We thus selected for further analysis 477 significant exons with correction by gene mean, 500 with correction by gene median, and 482 with splicing indices. Not surprisingly, a substantial number (297) of the significant calls from the three approaches overlapped. For the probe level analysis, we used a single threshold to select exons significant for additive, dominant or maternal terms (Table S5A, S5B in Additional data file 2) based on their identical null *d *score distributions (data not shown). The inheritance of differential exon splicing was predominantly additive (Figure 4a).

**Table 1:** Differential spliced exons and introns detected at different thresholds

	Delta*	Sig+^†^	Sig-^†^	Total	False^‡^	FDR (%)
Exon (gene mean)^§^	0.3	287	190	477	559	117
	0.4	177	129	306	205	67.0
	0.5	127	109	236	97	41.0
	0.6	92	86	178	55	30.8
	0.7	77	69	146	34	23.4
	0.8	57	54	111	23	20.8
	0.9	32	39	71	16	22.8
	1	28	29	57	12	20.5
						
Exon (gene median)^¶^	0.3	523	280	803	556	69.2
	0.4	328	172	500	203	40.6
	0.5	223	120	343	96	28.0
	0.6	154	76	230	54	23.5
	0.7	123	52	175	34	19.3
	0.8	101	47	148	23	15.6
	0.9	71	32	103	16	15.6
	1	56	28	84	12	13.8
						
Exon (splicing index)^¥^	0.3	402	249	651	302	46.4
	0.4	310	172	482	86	17.8
	0.5	233	132	365	30	8.16
	0.6	166	92	258	12	4.64
	0.7	134	86	220	6	2.53
	0.8	105	74	179	3	1.56
	0.9	80	60	140	2	1.16
	1	64	50	114	1	0.77
						
Intron	0.3	561	1,034	1,595	332	20.8
	0.4	405	523	928	85	9.17
	0.5	316	352	668	28	4.26
	0.6	239	220	459	12	2.61
	0.7	202	155	357	7	1.91
	0.8	176	120	296	5	1.53
	0.9	140	94	234	3	1.31
	1	120	75	195	2	1.15

The total number of analyzed exons was 68,022 for 15,349 genes, and of analyzed introns was 62,859 for17,434 genes. *Thresholds. ^†^Significant calls with greater expression in Col (Sig+) or greater expression in Van (Sig-). ^‡^False calls based on 1,000 permutations. ^§^Exonic splicing analysis using exon intensity corrected by gene mean. ^¶^Exonic splicing analysis using exon intensity corrected by gene median. ^¥^Exonic splicing analysis using splicing index.

As the default status of introns is to be spliced (Figure S4A in Additional data file 1), a direct comparison of intron probe intensities should reveal relative intron retention between genotypes. A total of 62,859 introns for 17,434 genes were analyzed with a mean density of 3.7 probes per intron (Figure S4B in Additional data file 1). For each intron, probe intensities were again tested under the same linear model with the additive, dominant and maternal terms. About 0.73% (459) of the analyzed introns were called significant for differential splicing between Col and Van at a 3% FDR, 239 retained in Col and 220 retained in Van (Table 1). Similar to exons, inheritance of the differential intron splicing was largely additive (Figure S4C in Additional data file 1). Although 0.14% (87) of analyzed introns were differentially expressed between mid-parent and F1 hybrids at a 13% FDR (Table S5D in Additional data file 2), many of which could merely reflect gene expression dominance as the probe intensity of retained introns depends on the level of gene expression as well as the level of intron retention.

We further analyzed the enrichment in Gene Ontology functional categories for genes containing the 477 differentially spliced exons (correction by gene mean) or the 459 differentially spliced introns, using Fisher's exact test (Table S6A, S6B in Additional data file 2). Differentially spliced introns were significantly enriched in the chloroplast thylakoid membrane (*p *< 4.51E-04) and thylakoid lumen (*p *< 3.93E-03) categories. Close examination of the corresponding 16 genes located in the thylakoid membrane revealed 11 genes as constituents of the photosynthetic apparatus, including the light harvest complex, photosystems, cytochrome b6f complex, P-type ATPase and electron transporters (Table S7A in Additional data file 2). In addition, many differentially spliced genes located in the thylakoid lumen functioned in proteolysis or protein folding, presumably to repair or maintain the photosynthesis apparatus (Table S7A in Additional data file 2). Differentially expressed genes were also significantly enriched in the thylakoid membranes (*p *< 3.78E-06; Table S6C in Additional data file 2), where they partially overlapped with the differentially spliced genes (Table S7B in Additional data file 2).

### Validation of differential splicing

As an *in silico *validation, we examined the fold enrichment of the detected differential exons in known alternatively spliced exons annotated in TAIR7 GenBank files. Differential exons called by each of our three approaches all showed enrichment in known alternatively spliced exons. There was 3.92-fold enrichment (*p *< 5.97E-09) for the 477 exons detected by analysis with correction by gene mean, 3.09-fold enrichment (*p *< 3.60E-06) for the 500 exons detected with correction by gene median, and 2.16-fold enrichment (*p *< 5.30E-03) for the 482 exons detected with splicing indices (Table S8A in Additional data file 2).

To provide an independent estimation of FDR for differential splicing, we tested a set of differential exons and introns by reverse transcription PCR (RT-PCR; Additional data file 3). Although the list was slightly biased toward highly significant calls, it still covered a broad range of the test statistic distribution (Figure S4D in Additional data file 1). Whenever possible, primers were designed to immediately flank the detected exon/intron region. Band patterns of RT-PCR products were compared between Col and Van across three maternal seed batch replicates. For 43 tested exons, 36 were from the list containing 477 exons (correction by gene mean) of which 44% (16/36) were suggested by RT-PCR (Table S8B in Additional data file 2). For differential introns, from the list of 459, 61% (38/62) were suggested by RT-PCR (Table S8B in Additional data file 2). Interestingly, many instances of differential splicing between Col and Van were also alternative splicing within Col or Van, as demonstrated by multiple transcript variants within genotypes. The splicing difference could be due to a novel transcript variant only occurring in one genotype or could be due to a different ratio of transcript variants between genotypes (Figure 4b). We were aware, however, that the gel-based validation was limited by sensitivity and resolution. Furthermore, the relationship between the band patterns and the probe intensities were indirect, as the probe intensity difference might reflect the sum of difference over several splicing isoforms (Additional data file 3).

### The impact of SFP probes on estimation of natural transcriptome variation

**Figure 5:**
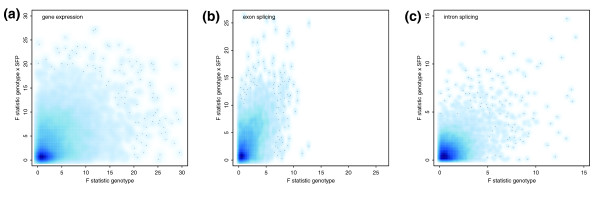
The effect of SFP probes on expression estimation. F statistics of genotype × SFP effects (y-axis) and that of genotype effects (x-axis) were obtained from the ANOVA model, Gene/exon/intron intensity = Genotype + SFP + Genotype × SFP + Error, for **(a) **gene expression, **(b) **exonic splicing, and **(c) **intronic splicing analysis.

Several microarray studies of natural transcript level variation have shown that the effect of SFP probes is small [15,16,46]; however, these studies all relied on gene expression arrays, where probe sequences are largely masked from genetic polymorphisms identified from expressed sequence tags. Whole genome tiling arrays lack this bias; their probe sequences are selected based on the relative distance along chromosomes. To estimate the effect of SFP probes on tiling array analysis, we applied variance partition on parental strain expression data for 10,764 genes, 5,280 exons and 10,931 introns that contained SFP probes. The model included genotype, SFP and genotype × SFP interaction effects. Although the variance contributed by SFP was moderate in comparison with that by genotype (Figure S5A in Additional data file 1), the variance by SFP × genotype interaction was significant, especially for exon splicing analysis (Figure 5).

We further examined the effect of SFP probes by comparing the results with or without the SFP probes included in the analysis. For gene expression, inclusion of SFP probes in the analysis generally increased the number of significant calls and decreased the permutation-based FDR at the same thresholds (Table S9 in Additional data file 2). This was caused by the overestimation of differential gene expression levels, especially in the direction of greater expression in Col as the majority of SFP probes have greater Col signals (Figure S5B in Additional data file 1). For each threshold we compared the fold enrichment of SFP-containing genes in the significant calls with or without the SFP probes included in the analysis. The SFP-containing genes were enriched in the significant calls even for the analysis in which SFP probes were excluded, since polymorphic genes tend to be differentially expressed. Nevertheless, the fold enrichments were significantly higher in the analysis that included SFP probes (Table S9 in Additional data file 2). For exon splicing analysis with SFP probes included, although the differential expression level of SFP-containing exons was generally overestimated in the direction of greater signals in Col, many non-SFP exons were overestimated in the direction of greater signals in Van (Figure S5C in Additional data file 1). This is likely because the inclusion of SFP probes caused the underestimation of Van gene expression; the signals of non-SFP exons within these genes were therefore overestimated for Van due to the correction by overall gene expression level. In comparison with gene expression and exonic splicing, the fold enrichment differences dependant on inclusion of SFP probes was not as striking for intronic splicing (Table S9 in Additional data file 2). This is likely because intronic splicing is highly correlated with sequence polymorphisms within introns. Nevertheless, the overlap of the significant calls between the analyses including and excluding SFP probes was very low (data not shown).

### *De novo *transcriptome variation

**Figure 6:**
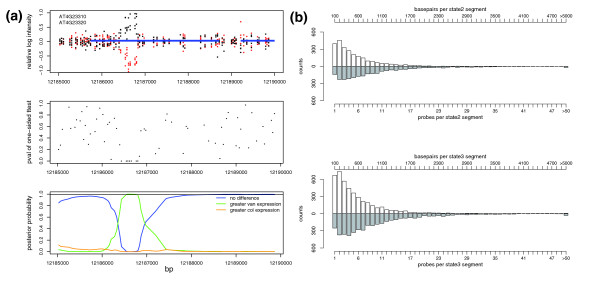
*De novo *transcriptome variation. **(a) **The generalized HMM procedure for a chromosome region. Upper panel: the relative log intensity for four Col replicates (red) and four Van replicates (black) along chromosome positions. Blue bars, annotated genes. Middle panel: probe level *p*-value was obtained by one-sided two sample *t*-test between Col and Van. Emission and transition probability was estimated by the Baum-Welch algorithm. Lower panel: posterior probabilities of no difference (blue), greater Van expression (green), and greater Col expression (orange) were determined using the Forward-Backward algorithm. Black line: 0.99 posterior probability cutoff. **(b) **The distribution of the length of differential segment (white bars) and probes per differential segment (grey bars) for state 2 (upper panel) and state 3 (lower panel) segments.

As the annotation-based approach is limited by expression library coverage, we developed a complementary approach using a generalized hidden Markov model (HMM) to detect differentially transcribed fragments between Col and Van, independent of annotation (Figure 6a). For the cDNA hybridizations, probe-level *p*-values were collected from one-sided two sample *t*-tests for the alternative hypothesis H1: μVan > μCol. Our model was then built to partition probe-level *p*-values into three hidden states, representing roughly equal expression between Col and Van (state 1, *p*-values are uniformly distributed), greater expression in Van (state 2, *p*-values are close to 0), and greater expression in Col (state 3, *p*-values are close to 1). Each hidden state contains a discrete emission distribution with 50 bins spanning [0, 1], which describes the probability of observing a given probe-level *p*-value conditioned on the hidden state. The model also contains a three-by-three base transition matrix T with three free parameters, t_11_, t_22_, and t_33_, where t_ii _is the probability of transitioning from state i to state i in a single base step. The rest of the matrix is determined by the relationship t_ij _= (1 - t_ii_)/2 when i does not equal j. To incorporate the variation of probe distance, the transition matrix was further defined as T^b ^for two probes whose midpoints are b bases apart. This heterogeneous Markov process allows more frequent state transitions between more distant neighboring probes [47]. The Baum-Welch algorithm [48] was used to estimate the emission distributions directly from the data, with a quasi-Newton bounded optimization algorithm [49] applied once every ten iterations to re-estimate the transition probabilities. This hybrid estimation approach was applied separately to each of the five chromosomes, with no significant differences in emission distributions or transition parameters observed (Figure S6 in Additional data file 1). Following parameterization, the Forward-Backward algorithm was applied to compute the posterior probability for all three states at each probe position. Segments were collected within which all probes have a state 1, state 2 or state 3 posterior probability > 0.99. A total of 6,800 differential segments were identified, 4,262 with greater expression in Col and 2,538 with greater expression in Van. The median length of the differential segments was about 330 bp and 7 probes (Figure 6b).

The differential segments fell largely within the annotated gene regions, although the exact coincidence of segment boundaries and annotated gene/exon boundaries was often undetectable, likely due to the limitation of probe density of the 1 F array. For a comparison of the HMM and the annotation-based analyses, we collected all differential segment(s) that contained ≥3 probes within the annotated gene boundaries. These differential segments, using our predefined criteria (Materials and methods), represented 2,673 differentially expressed genes, 1,222 differentially spliced genes, 109 novel gene boundaries and 85 non-annotated transcripts (Table 2). About 79% of differentially expressed genes detected by the annotation approach at a 3% FDR were also detected by the HMM analysis, while only 10% of differentially spliced genes detected by the annotation approach were also detected by the HMM analysis (Table S10 in Additional data file 2). Furthermore, 301 differentially spliced genes detected by annotation were called by the HMM analysis as differentially expressed genes, and an additional 295 genes labeled as differentially expressed by annotation were called by the HMM analysis as differentially spliced (Table S10 in Additional data file 2). Differentially spliced genes detected by the HMM analysis were enriched in known alternatively spliced genes by 1.66-fold (*p *< 2.56E-08), a fold enrichment comparable to that of the annotation approach. Among the differentially spliced genes subjected to RT-PCR validation, 22 were detected by the HMM analysis, of which 13 were suggested to be true positives.

**Table 2:** The number of significant calls by *de novo *transcriptome profiling

		Col > Van	Van > Col	Total
Annotation	Differential expression*	1,626	923	2,549
	Differential exonic splicing^†^	287	190	477
	Differential intronic splicing^‡^	239	220	459

HMM	Differential expression	1,667	1,006	2,673
	Differential splicing	765	457	1,222
	Un-annotated transcript	37	48	85
	Un-annotated 5'	31	32	63
	Un-annotated 3'	30	16	46

*The number of significant calls at a 3% FDR for differentially expressed genes. ^†^The number of significant calls for differentially spliced exons using exon probe intensity corrected by gene expression mean. ^‡^The number of significant calls at a 3% FDR for differentially spliced introns.

Several factors may explain the discrepancy between the two approaches. First, for many differentially spliced exons/introns detected by the annotation approach, the corresponding genes were expressed at different levels. Currently, our HMM method is unable to detect splicing differences in the presence of gene expression differences, as quantitative internal variation of a differential segment was not accounted for. Second, for many differential segments detected by the HMM analysis, which were called as differentially spliced, their probe intensity differences were relatively small or they involved un-annotated exon/intron structure. Such splicing differences are likely unable to be called by the annotation approach, as the correction of the probe intensities by whole gene expression level would mask additional small differences. Importantly, the cutoff to distinguish between differential expression and differential splicing for the HMM analysis (Materials and methods) was rather arbitrary. It is likely that a finer tiling array resolution would allow a finer delimitation of the start and stop positions of HMM segments.

## Discussion

The two *A. thaliana *accessions used in this study, Col and Van, were collected from distinct geographic locations. At the 3-day-old stage, overall growth and morphology was indistinguishable between Col and Van seedlings. About 8% of their genes, however, already exhibited differences in expression level. Differentially expressed genes were enriched in biological processes that depend on variable environmental factors, including light, water, salt and pathogen presence (Table S3 in Additional data file 2), suggesting a possible mechanism of adaptation to distinct habitats. Interestingly, Van harbors a null mutation at *ERECTA *[50], which has been identified as a *trans *regulatory hot spot [51]. Future mapping of expression quantitative trait loci (eQTL) in segregating populations between Col and Van is required to dissect the causative loci for these gene expression variations [51,52]. Several recent microarray studies have consistently demonstrated substantial natural variations in transcript level among *A. thaliana *accessions [18,46,51-53]. The exact proportions of differential genes reported by these studies, however, are quite different, ranging from 4% [51] to 32% [18]. Between Col and Van, 10% of differential genes were detected in a study of seven *A. thaliana *accessions [46,53]. Different biological samples, statistical approaches and microarray platforms all contribute to these differences. Distinct from previous studies, we controlled the effect of sequence polymorphisms using parallel genomic hybridizations and demonstrate that this is critical for analysis of natural transcriptome variation using whole genome tiling arrays. Even so, we can not completely exclude the possibility that there were SFP probes not removed at the selected threshold, which may introduce technical bias resulting in, for example, a smaller number of genes with greater expression in Van.

About 3% of genes exhibited expression dominance, largely contributed by Van. Haploinsufficiency of *ERECTA *signaling could be one explanation. The general down-regulation of growth-related processes and up-regulation of defense responses in F1 hybrids could also be explained by the genetic incompatibility of rapidly evolving pathogen resistance genes between Col and Van [54]. Studies on the inheritance pattern of gene expression in F1 hybrids have led to quite different conclusions. In maize F1 hybrids, only 20% of differentially expressed genes were estimated to be dominant by microarray profiling [11,17], while two-thirds of 30 genes tested by northern blot were shown to be dominant [55]. In *Arabidopsis*, Vuylsteke *et al*. [18] found that, depending on accession pair, 6-21% of genes showed dominance. Although the proportion of dominant to additive genes estimated in our study (35%) was within the range they reported, we observed much less genes exhibiting overdominance. In mouse F1 hybrids between laboratory strains, Cui *et al*. [16] estimated that the proportion of dominant to additive genes was 36%. Even less dominant effects were detected in F1 hybrids derived from natural mouse strains [15]. Thus, for maize, *Arabidopsis *and mouse, the additive effect of gene expression seems to be predominant, with a significant number of genes showing dominant inheritance. In contrast, expression inheritance of *Drosophila *is largely dominant in a sex-dependent manner [12-14]. Unlike mouse [16] and *Drosophila *[12], which show significant gene expression differences between reciprocal F1 hybrids, the parental effect is small in *Arabidopsis *[18] and maize [11,17]. In our study, less than 1% of the analyzed genes showed parental effects.

The study of gene expression additivity in an F1 hybrid system tests the sum of regulatory effects across underlying eQTL. Nevertheless, the non-additive effect of eQTL and transgression have been shown to be common [51,52,56]. Direct measurement of ASE in F1 hybrids provides an alternative to linkage analysis for detection of *cis*-regulatory variation that contributes to gene expression additivity. We determined that 8% of the analyzed genes had ASE using SFP probes located within transcripts. As the genes had to contain SFPs to be analyzed, these genes are already enriched for *cis *variation, suggesting that 8% is an overestimation of the proportion of *cis*-regulated genes. The power for detecting allelic differences in RNA samples by SFP probes was shown to be low [44]. This is partly because the detection approach relies on linear assumption of probe behavior, which could be invalid when target concentration is too low or too high in RNA samples. Estimation bias could be further introduced due to the fact that only probes for perfect match targets were present on the array. ASE detection using single nucleotide polymorphism arrays, which contain probe sequences for both alleles, will potentially solve this problem. The high fold enrichment of ASE genes in highly significant differential genes is consistent with previous observations that *cis*-variations tend to have large expression effects [51,57].

In contrast to gene expression, differential splicing showed few dominant effects in F1 hybrids, indicating that splicing variation is mainly due to *cis *variation, as expected. Nevertheless, additive *trans *variation cannot be ruled out. Qualitative differences in transcript structure could have more severe effects on normal cellular functions than quantitative differences in transcript level. On the other hand, splicing variation provides one of the major mechanisms for rapidly evolving new protein functions, which is critical for survival in unpredictable environments. A recent study suggests that proteins appear to be more tolerant of structural deletions, insertions and replacements than previously thought [58]. Interestingly, genes associated with various stress responses are especially prone to alternative splicing [32,59]. We found that many genes involved in light acclimation responses, which modulate the composition and function of the plant photosynthetic apparatus in response to the changing irradiance [60], were differentially spliced between Col and Van. Future studies in other *A. thaliana *accessions should reveal whether this is a common phenomenon.

On the affymetrix 1.0 F array, the most frequent distance between the middle bases of two adjacent probes is 35 bp. With this resolution the majority of annotated genes are well covered in terms of probe density. Thus, probe effects are generally not a major issue in the estimation of overall gene expression difference, as relative change across all probes is tested. In contrast, the estimation of splicing differences, especially exonic splicing variation, is more challenging. For annotation-based exonic splicing analysis, as discussed previously, the signal-to-noise ratio of a true differential exon is reduced by the correction for the overall gene expression difference. Probe density becomes the limiting factor here. The small number of interrogating probes (Figure S4B in Additional data file 1) indicates that the statistical power to detect a differential exon is lower than the power to detect a differential gene, and that the FDR will be higher. For example, a low quality probe may not detect a true expression difference for the gene but suggest the presence of a splicing difference for that exon. The high FDR associated with the differential exons, obtained by either permutation analysis or experimental validation, clearly demonstrates this.

Despite these limitations, our study demonstrates that the high density whole genome tiling array is a powerful platform for comparative transcriptome profiling in addition to developmental expression analysis [61]. Our strategy of annotation-based modeling of the probe level intensity for gene expression and splicing could be extended to include additional experimental treatments, such as environmental conditions and/or developmental stages. The HMM method we have developed is highly adaptable since the model requires only a string of *p*-values as input; any statistical test may be selected to appropriately extract the information from probe-wise data for the comparison of interest. Refinements on array design, specifically probe density, and our analysis tools will certainly benefit future large-scale studies such as gene expression association mapping. Next generation sequencing technology, including Roche/454, or Illumina/Solexa paired-end sequencing, represents a complementary method for transcriptome profiling. Here, the precise transcript gene structure is identified, but without multiple independent biological replicates, counted differences may not be significant. Tiling arrays and high-throughput sequencing technologies are synergistic, but so far very few studies have performed a direct comparison or leveraged the strengths of both methods [62].

## Materials and methods

### Plant material

Seeds of *A. thaliana *accessions Col-0 (accession number [CS22625]) and Van-0 (accession number [CS22627]) were obtained from the *Arabidopsis *Biological Resource Center. Seeds were planted in soil, imbibed for 5 days in a cold room at 4°C, and moved to a green house on 31 January 2005. Plants were grown in the green house with 16 h light (cool white light supplemented with incandescent) and 8 h dark at a constant temperature of 20°C. The first cross experiment was conducted on 28 February 2005, and on 1 March 2005 the second cross experiment was conducted between the same plant pairs as in the first experiment. Both cross experiments began around 9:00 am and ended around 5:00 pm. In each experiment, four replicate crosses for each of Col × Col, Van × Van, Van (♀) × Col (♂), and Col (♀) × Van (♂) were made. Each replicate cross was between individual paternal and maternal plants and each parental plant was only used once (16 Col and 16 Van plants used in total). For each replicate cross, the seeds from the two experiments were combined and used as one maternal seed batch. Thus, for each of the crosses (Col × Col, Van × Van, Van (♂) × Col (♀), and Col (♂) × Van (♀)), there were four independent maternal seed batch replicates.

Approximately 250 seeds from each maternal seed batch were grown on a single petri dish. After gas sterilization for 4 h, seeds were plated on a total of 16 0.7%-agar (Sigma, St. Louis, Missouri, USA) plates supplemented with 0.5× Murashige and Skoog salts (Sigma). Seed plates were placed horizontally in a growth chamber (Percival Scientific model E361, Percy, Iowa, USA) after stratification for 5 days at 4°C. Seedlings were grown for 78 h under a diurnal mode with 12 h light (cool white light supplemental with red light) and 12 h dark at a constant temperature of 20°C.

### Sample preparation and microarray hybridization

Seedlings grown on each plate were split for genomic DNA and RNA preparation. Genomic DNA was isolated from 100 seedlings for each plate using a DNeasy plant mini kit (Qiagen, Valencia, California, USA). About 300 ng DNA was labeled using a BioPrime DNA labeling system (Invitrogen, Carlsbad, California, USA) with conditions modified as previously described [39]. About 20 μg total RNA was isolated from an additional 120 seedlings per plate using an RNeasy plant mini kit (Qiagen). Poly-(A) RNA was enriched from total RNA using an Oligotex mRNA mini kit (Qiagen). Poly-(A) RNA was mixed with 166 ng random hexamer (Invitrogen) and subjected to first-strand cDNA synthesis (Invitrogen) according to the manufacturer's recommendations in a total volume of 40 μl at 42°C for 1 h. The 40 μl first-strand reaction was used in second-strand cDNA synthesis (Invitrogen) according to the manufacturer's recommendations in a total volume of 300 μl at 16°C for 2 h. Samples were then subjected to RNase treatment at 37°C for 20 minutes with 20 units RNaseH (Epicentre, Madison, Wisconsin, USA), 1 unit RNaseA and 40 units RNaseT (Ambion, Austin, Texas, USA). Double-stranded cDNA was further purified using a Qiaquick PCR purification kit (Qiagen), and then labeled using a BioPrime DNA labeling system (Invitrogen) as described above. About 30 μg of labeled product from genomic DNA or from double-stranded cDNA was subjected to hybridization to *Arabidopsis *Tiling 1.0 F array (Affymetrix) using a standard gene expression array washing/staining protocol (Affymetrix).

### Validation of differential splicing and allele specific expression

For each of the differential exons and introns selected for validation, gene specific primers were designed to flank the predicted exon or intron using Primer3 [63], as listed in Table S11A in Additional data file 2. Seedlings were grown as described above, with three maternal seed batch replicates for each of Col and Van. Total RNA was isolated from 120 seedlings per plate using a RNeasy plant mini kit (Qiagen). For each sample, approximately 2 μg total RNA was reverse transcribed using 40 U Superscript III (Invitrogen) and 0.5 μg Oligo(dT)12-18 primer (Invitrogen) in a total volume of 20 μl at 42°C for 1 h. First strand cDNA was amplified using gene specific primer pairs with the following PCR conditions: denature at 94°C 3 minutes, 38 cycles of 94°C 15 s, 68°C 15 s, 72°C 30 s (or 45 s depending on the product size), extension at 72°C 5 minutes. PCR products were separated on 1.2% agarose gel.

For the two selected ASE genes, a single nucleotide polymorphism within each gene was selected. The sequences of flanking PCR primers and extension primers were designed using Assay Design 3.1 (Sequenom, San Diego, California, USA), as listed in Table S11B in Additional data file 2. Reverse transcription was performed as described above for mRNA sample from a F1 hybrid. Gene specific PCR was then performed for the genomic DNA and reverse transcription products for that F1 hybrid, using conditions described above except using 42 thermo-cycles. PCR products were cleaned with a Qiaquick PCR purification kit (Qiagen), and submitted to the University of Chicago Sequencing Core for extension reaction and mass spectrometry (Sequenom).

### Data analysis

The accession numbers of the microarray data in this study have been deposited in the Gene Expression Omnibus (GEO) [64] ([GEO:GSE8891], [GEO:GSE13620]). The genome tracks of SFPs, deletions/duplications, gene expression variation, allele specific expression, splicing variation, and transcribed fragment variation are included in Additional data file 4, which can be viewed using GBrowser [65].

### Array annotation

Perfect match probes from the *Arabidopsis *tiling 1.0 F array (Affymetrix) were megablasted against the *Arabidopsis *genome release version 7 including mitochondria and chloroplast sequences with word size ≥8 and E-value ≤0.01. Single perfect matches, without a second partial match of >18/25 bp, were selected, giving a total of 1,683,620 unique probes. These were mapped to annotated mRNAs as intron, transcription unit (exon, alternative exons), intergenic, or flanking probes that span an annotated boundary.

#### Detection of deletion/duplication

Raw intensities from .CEL files of genomic hybridizations were corrected for spatial effects and log transformed as previously described [66]. Probe intensities from all 1,683,620 probes were quantile normalized using the Bioconductor package Affy. SFPs were detected using the Bioconductor package Siggenes as previously described [39]. For detection of deletion/duplication, the probe-level *p*-values were collected from one-sided two sample *t*-tests for the alternative hypothesis H1: μVan > μCol. Each of the five chromosomes was divided to 1 Mb bins and analyzed separately to reduce the memory usage. The segment algorithm [41] was applied to the probe level *p*-values, with maximum segment length set as 5,000 probes × 35 bp/probe = 175 kb, and base per segment set as 5 kb. Thus, the algorithm calculated 1 to 200 possible segments within a 1 Mb region to determine the segment boundaries. The optimal number of segments from each of the 1 Mb bins were determined by the Akaike information criterion or Bayesian information criterion. Segments with median probe *p*-value > 0.99 (deletion) or median probe *p*-value < 0.02 (duplication) were collected for each 1 Mb bin and jointed together if they were located at bin boundaries. To reduce the chance of false positives, indels were further selected only if they contained ≥6 probes and interrogated by ≥1 probe/1 kb density.

#### Annotation-based analysis

Raw intensities from .CEL files of cDNA hybridizations were corrected for spatial effects and log transformed as previously described [66]. For annotation-based analysis, probes with the 5% weakest intensity in genomic DNA hybridization, probes interrogating the 125,043 SFPs (5% FDR) and 1,781 indels, intergenic probes, probes spanning exon boundaries, and probes interrogating multiple transcripts were excluded from the RNA data. The intensities of the remaining probes were quantile normalized. Exon probes were defined as probes interrogating gene sequences that present in ≥50% of the expressed clones. Intron probes were defined as probes interrogating gene sequences that either were absent in expressed clones or present in <50% of expressed clones. For each probe, the probe effect was removed by subtracting the mean log intensity across all samples. Genes with ≥3 exon probes were analyzed for differential expression by fitting a linear model: Intensity = Additive + Dominant + Maternal + Error. The additive, dominant and maternal terms were contrasted as (1, -1, 0, 0), (0, 0, 1, 1), (0, 0, -1, 1), respectively, within the linear model. Exonic differential splicing was analyzed for genes with ≥2 exons and ≥5 exon probes. Intronic differential splicing was analyzed for genes with ≥2 exons and ≥3 exon probes. Within these selected genes, exons or introns containing ≥2 probes were subjected to further analysis.

For exonic splicing analysis using probe intensities corrected by gene mean, for each gene the probe intensities were fitted by the linear model: Intensity = Additive + Dominant + Maternal + Error. For each exon, the residuals were then fitted by the linear model: Residual = Additive + Dominant + Maternal + Error. For exonic splicing analysis using probe intensities corrected by gene median, exon probe intensities were corrected by a median-polished gene expression value estimated across strain replicates and gene probes, which were then fitted by the linear model. For exonic splicing analysis using splicing indices, the mean exon expression was summarized as the mean across exon probes for each replicate sample. Gene expression was then calculated as the average of exon means across exons and across replicate samples within genotype. The splicing indices (exon mean/gene mean) were then fitted by the linear model. Both corrected exon probe intensities and the splicing indices generally showed distributions close to a normal distribution (data not shown).

For detection of intronic differential splicing, intron probe intensities were directly fitted by the linear model: Intensity = Additive + Dominant + Maternal + Error. FDRs were determined by 1,000 permutations, with the following procedure: step 1, fit a partial model missing the term being tested; step 2, permute residuals; step 3, add permutated residuals to the predicted values; step 4, fit those data with a full model; step 5, calculate a *d *score; step 6, repeat steps 2 to 5 for 1,000 times. The null hypothesis here is that the term being tested is not significant; thus, residuals from partial modeling are assumed to be independent random variables that could be permutated by across samples. The *d *score is defined as the Coefficient/(Standard deviation + s0), where s0 is the median of standard deviations across all genes and all permutations [42]. The adding of constant s0 to the denominator is to avoid very small effect genes being called significant [42]. The *d *scores were ranked for each of the 1,000 permutations and a null distribution was obtained by averaging *d *scores across permutations for each rank. For each threshold, the FDR was calculated as the average number of permutation *d *scores exceeding the threshold divided by the number of real *d *scores exceeding that threshold [42]. We found that FDRs calculated by this method sometimes exceeded 100%. This was due to the *d *score distribution of non-significant genes in real data being tighter than the null [42], meaning no significant enrichment over background.

To detect ASE, probes with the 5% weakest intensities in genomic DNA hybridization, intergenic probes, probes spanning exon boundaries, and probes interrogating multiple transcripts were excluded from the RNA data. Exon probes were defined as probes interrogating gene sequences that present in ≥50% of the expressed clones. These exon probes, including non-SFP probes and SFP probes, were quantile normalized. A total of 9,745 genes containing ≥5 non-SFP exon probes and ≥1 SFP exon probe were selected for further analysis. For each gene, the additive, dominant and maternal effects were estimated by the linear model. These effects were subtracted from the log intensities of SFP probes to correct for fold difference of gene expression level across genotypes. The corrected SFP probe intensities reflected the relative binding (allelic composition) for a given amount of target. Under the linear assumption, the intensity of SFP probes is expressed as:

I_Col _= C_Col _× S_Col_

I_Van _= C_Van _× S_Van_

(1) I_mid _= 1/2 × C_Col _× S_Col _+ 1/2 × C_Van _× S_Van_

Here, I represents probe intensity, C target concentration, and S binding coefficient for the corresponding target. Suppose C_m-Col _= C_m-Van _= C_m _and C_g-Col _= C_g-Van _= C_g_, where subscript m- and g- represent RNA and DNA hybridization, respectively, it follows that:

I_m-Col_/I_g-Col _= I_m-Van_/I_g-Van _= I_m-mid_/I_g-mid _= C_m_/C_g_

or

(2) log I_m-Col _- log I_g-Col _= log I_m-Van _- log I_g-Van _= log I_m-mid _- log I_g-mid_

Re-arranging equation 2, we have:

log I_m-mid _= log I_g-mid _+ 1/2 × (log I_m-Col _- log I_g-Col _+ log I_m-Van _- log I_g-Van_) = log I_g-mid _+ 1/2 × D

We included both Col and Van genomic hybridizations here to estimate the mid-parent values, in an attempt to reduce the estimation bias associated with different targets. For each SFP probe, D was calculated using mean log intensities across four replicates of genomic or RNA hybridization for Col and Van. We then obtained eight independent mid-parent values from the eight replicates of F1 genomic hybridization. For each gene, the eight replicates of F1 RNA hybridization were tested for deviation from the mid-parent values by a simple linear regression across SFP probes within the gene. A permutation approach was applied to determine the FDR.

ASE was also analyzed using mid-parent values estimated as the average natural intensities of parents for RNA hybridizations (equation 1). The log transformed probe intensities were first converted back to natural intensities. Using non-SFP probes, the median-polished gene intensity was estimated for each genotype. The SFP probe intensities were divided by the corresponding gene intensities to correct for expression difference. Then, for each SFP probe, the mid-parent values were estimated as the average of parental values. Four independent data points were obtained by pairing four replicates of Col and Van RNA hybridizations without replacement. The eight replicates of F1 RNA hybridization and four mid-parent values were then log transformed for linear regression. A permutation approach was applied to determine the FDR.

To estimate the effect of SFP probes on the analysis of natural transcirptome variation, we applied variance partition for genes/exons/introns that contained ≥1 non-SFP probe and ≥1 SFP probe on parental strain expression data. An ANOVA model, Gene/exon/intron intensity = Genotype + SFP + Genotype × SFP + Error, was tested for each gene/exon/intron. Here, the probes with the 5% weakest signals, probes interrogating the 125,043 SFPs and 1,781 indels were not removed from the RNA data, as if there were no genomic DNA hybridization data available. The same set of genes/exons/introns was compared for analyses with and without SFP probes.

The annotation-based analysis was coded as R scripts (Additional data file 5).

#### Hidden Markov model

For HMM analysis, probes with the 5% weakest intensities in genomic DNA hybridization, probes interrogating the 125,043 SFPs (5% FDR) and 1,781 indels were excluded from the RNA data. The intensities of the remaining probes were quantile normalized. Probe level *p*-values were collected from one-sided two sample *t*-test for the alternative hypothesis H1: μVan > μCol. We included three hidden states in our model to distinguish equal, Col higher, and Van higher expression. Each state contained a discrete emission distribution with 50 bins equally spaced from zero to one. The probability of state transitions was represented by a 3 × 3 base transition matrix T, which contained three free parameters, t_ii _for i = {1, 2, 3}, where t_ii _is the probability of staying in the same state (i) from one base to the next. The off-diagonal elements in this matrix were determined by the equation t_ij _= (1-t_ii_)/2. Since probes were not always equally spaced, we treated state transitions as a simple Markov chain such that the probability of a state transition t_ij _* between two probes whose midpoints are b bases apart was defined by the matrix T* = T^b^. We used a modified version of the Baum-Welch algorithm [48] to obtain estimates for the emission distributions for the three states and the transition probabilities t_ii_. In our modified approach, the standard expectation-maximization procedure was applied to estimate the emission distributions during each iteration of the Baum-Welch algorithm. During the convergence of the Baum-Welch algorithm, a quasi-Newton bounded optimization algorithm known as L-BFGS-B [49] was applied once every ten iterations to re-estimate the transition probabilities within the bounded interval [0.95, 1] for i = {1, 2, 3}. It does not appear that our bounds affected transition estimates as all transitions were estimated to take values at some distance from the bounds. Parameter estimation was done twice for each chromosome, using different starting emission distributions to make sure that the Baum-Welch algorithm was converging to the global maximum of the parameter likelihood surface rather than local peaks. Following parameter estimation, the Forward-Backward algorithm was applied to determine the posterior probability of each state for each probe. Segments were collected within which all probes have a state 1, state 2 or state 3 posterior probability > 0.99.

Differential segments (state 2 and 3 segments) were compared with annotation. For each of the annotated genes interrogated by ≥3 probes, we collected all differential segment(s) that contained ≥3 probes within the annotated gene boundaries. Differentially expressed genes were defined as those annotated genes that had ≥1/3 probes located within the observed differential segment(s). Differentially spliced genes were defined as those annotated genes that had <1/3 probes located within the differential segment(s), or those genes that contained ≥2 differential segments from opposing hidden states. Differential segments overlapping with annotated gene boundaries and extending those boundaries by ≥3 probes detected novel gene boundaries. In addition, these differential segments for novel gene boundaries were either not overlapped with adjacent genes, or overlapped but the distances between the two neighboring gene boundaries were ≥10 probes. Differential segments (≥5 probes) completely outside of any annotated gene region represented novel transcripts.

The implementation of HMM and the related analysis was coded as R scripts (Additional data file 6).

## Abbreviations

ASE: allele specific expression; eQTL: expression quantitative trait loci; FDR: false discovery rate; HMM: hidden Markov model; RT-PCR: reverse transcription PCR; SFP: single feature polymorphism.

## Authors' contributions

JOB conceived the study. XZ carried out the experiments. XZ, JKB (implemented the generalized HMM) and JOB analyzed the data. XZ, JKB and JOB drafted the manuscript. TG carried out the array annotation. W-HL advised the implementation of the generalized HMM. All authors read and approved the final manuscript.

## Additional data files

The following additional data are available with the online version of this paper. Additional data file 1 provides supplemental figures. Additional data file 2 provides supplemental tables. Additional data file 3 provides gene plots and gel pictures for splicing validation. Additional data file 4 provides genome tracks of SFPs, indels, expression variation, ASE, splicing variation and transcribed fragment variation. Additional data file 5 provides R codes for annotation-based analysis. Additional data file 6 provides R codes for HMM analysis.

## Supplementary Material

Additional data file 1 Supplemental figures. Click here for file

Additional data file 2 Supplemental tables. Click here for file

Additional data file 3 Gene plots and gel pictures for splicing validation. Click here for file

Additional data file 4 Genome tracks of SFPs, indels, expression variation, ASE, splicing variation and transcribed fragment variation. Click here for file

Additional data file 5 R codes for annotation-based analysis. Click here for file

Additional data file 6 R codes for HMM analysis. Click here for file
