# Supplementary material for: Whole genome transcriptome polymorphisms in Arabidopsis thaliana
Source: Genome Biol. 2008 Nov 24;9(11):R165. doi: 10.1186/gb-2008-9-11-r165 (PMC2614497; doi:10.1186/gb-2008-9-11-r165)
Supplement: Additional data file 1 — Supplemental figures. [file gb-2008-9-11-r165-S1.pdf]

Supplemental Figure 1

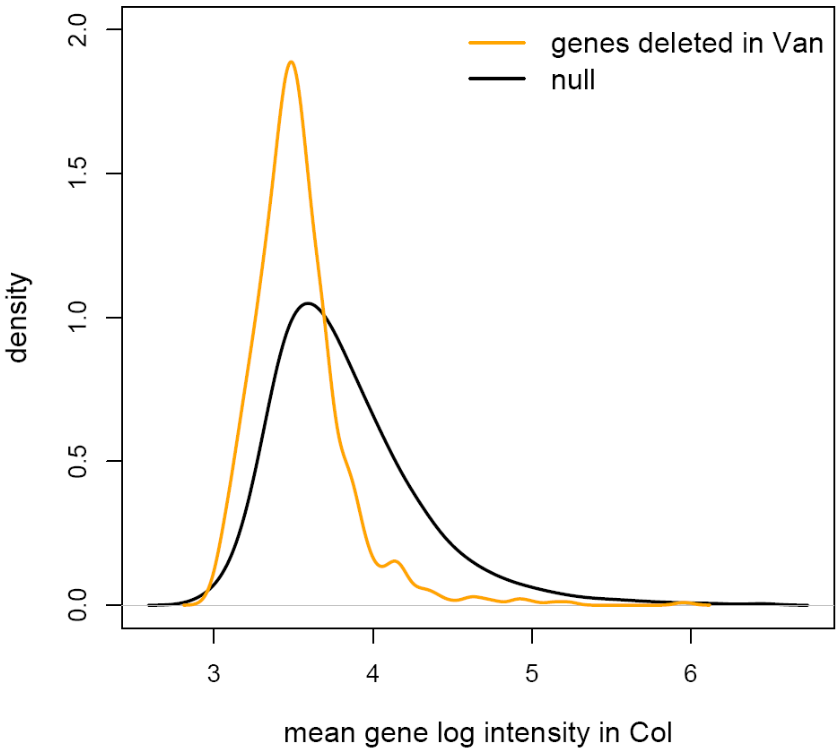

The distribution of absolute gene expression levels for 773 genes in Col which are located completely within deleted regions in Van (orange). The mean probe log intensity across four Col replicates and across gene probes (x-axis) was collected for each annotated gene interrogated by >=3 probes. The density distribution of the 773 genes deleted in Van was compared with a null distribution (black) obtained by sampling the same number of genes 1,000 times for the four Col replicates.

Supplemental Figure 2. Additive, dominant and maternal effects of gene expression.

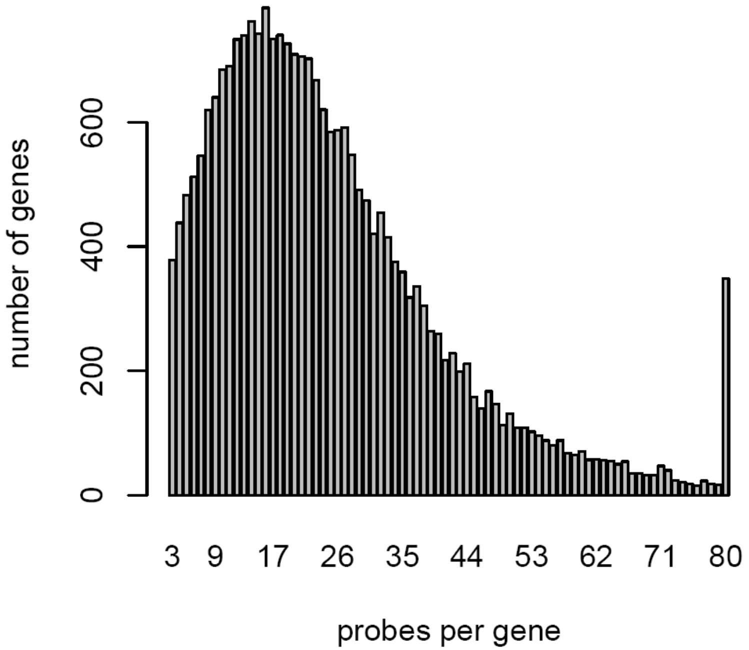

Supplemental Figure 2A. The distribution of the number of exon probes per gene.

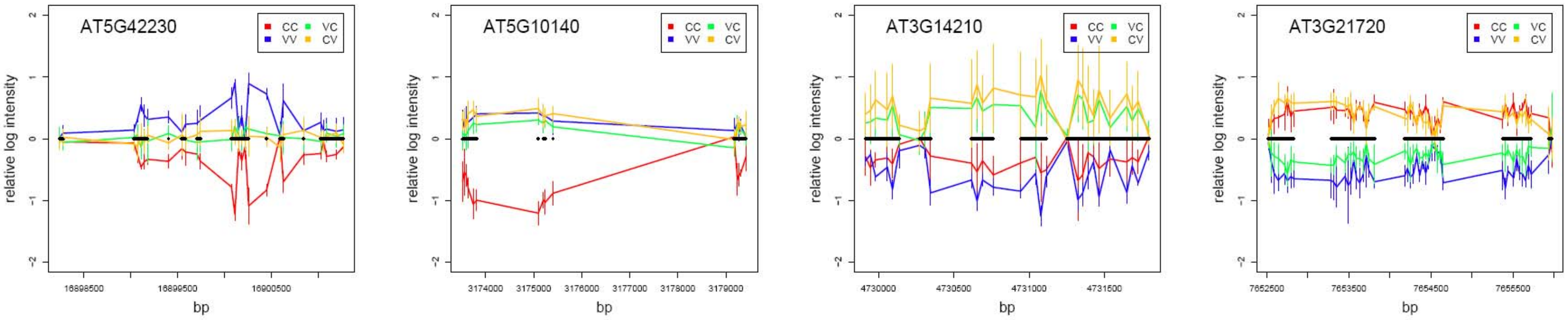

Supplemental Figure 2B. Examples of additive (AT5G42230), dominant (AT5G10140), over-dominance (AT3G14210) and maternal (AT3G21720) gene expression effects. The log intensity differences (y-axis) among Col (red), Van (blue), Col-mother F1 (orange), and Van-mother F1 (green) were plotted along chromosomal positions (x-axis), with standard deviation labeled. Exons were indicated as thick black horizontal bars at y=0.

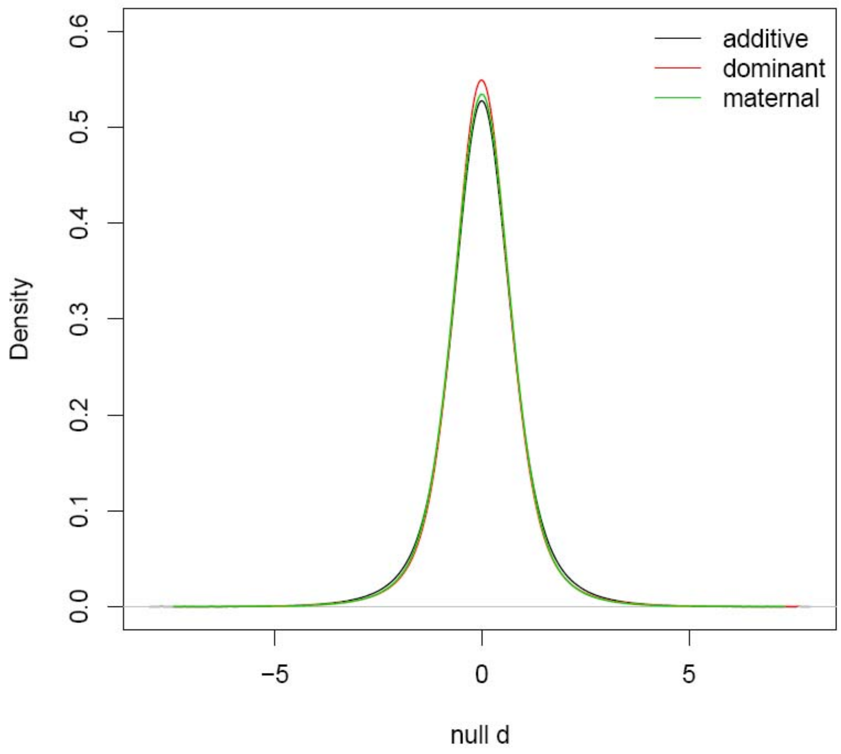

Supplemental Figure 2C. The null d score distributions of additive, dominant and maternal terms for gene expression, obtained by 1,000 permutations.

Supplemental Figure 3. Detection of ASE.

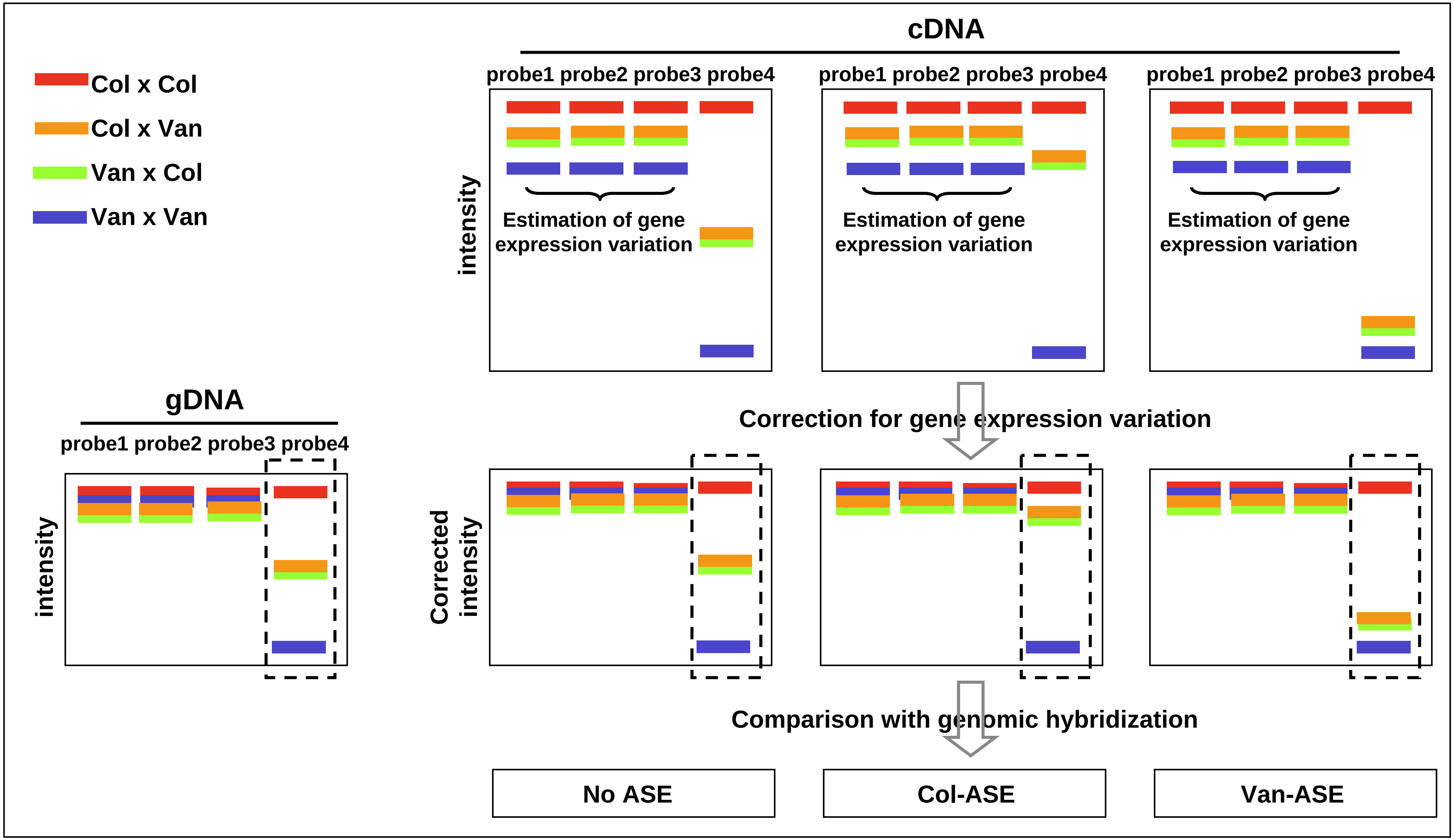

Supplemental Figure 3A. Detection of ASE by SFP probe. The probe intensity is plotted as natural intensity for illustration purpose. The analysis is however performed at log scale. The probe intensities (y-axis) of four probes (x-axis) within a transcribed gene are present for Col (red), Van (blue), Col-mother F1 (orange) and Van-mother F1 (green). The 4th probe interrogates a SFP. For cDNA hybridization, gene expression variations, estimated using non-SFP probe intensities, are corrected. After correction of gene expression variation, the SFP probe intensity ratios among Col, Van and F1 hybrids of cDNA hybridization are compared with the corresponding SFP probe intensity ratios of genomic hybridization to infer ASE (see Materials and Methods).

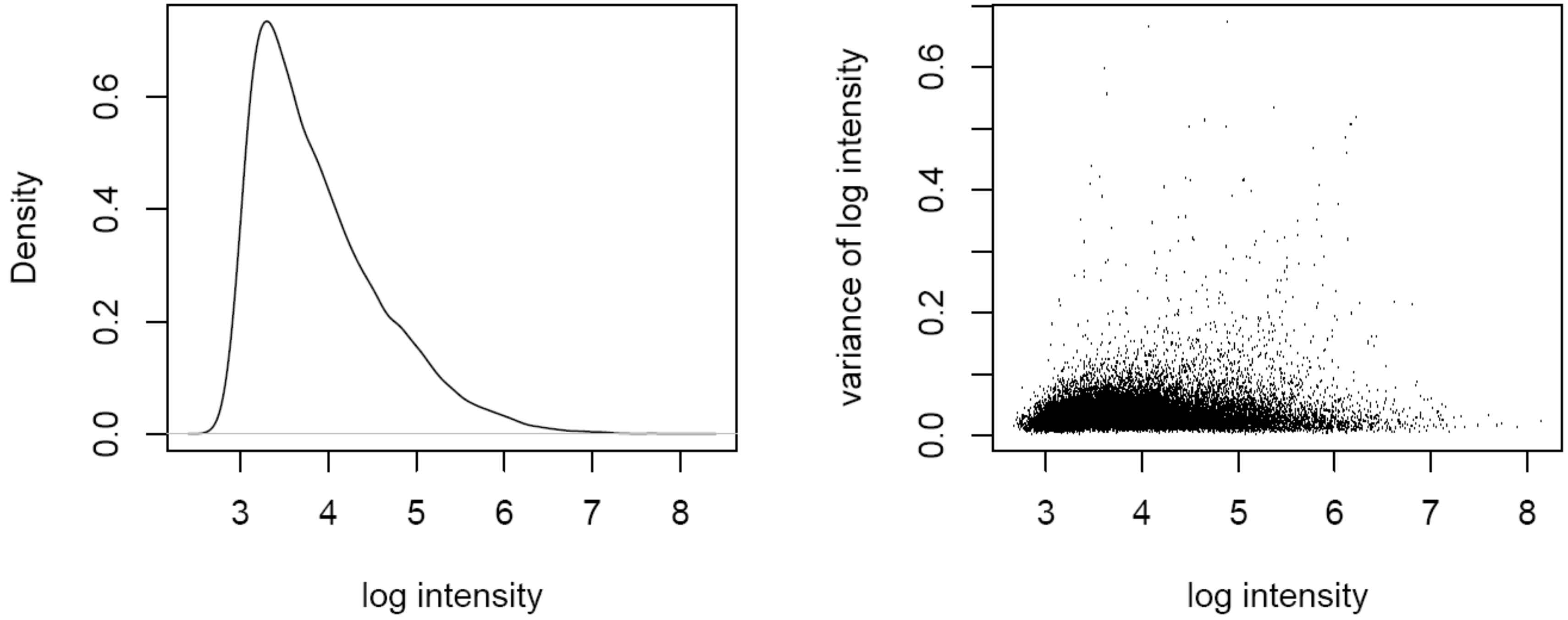

Supplemental Figure 3B. The distribution of mean log intensities (left) and variance (right) across mid-parent and F1 hybrids for SFP probes interrogating transcribed regions. The log intensities of F1 hybrids were corrected for expression variations.

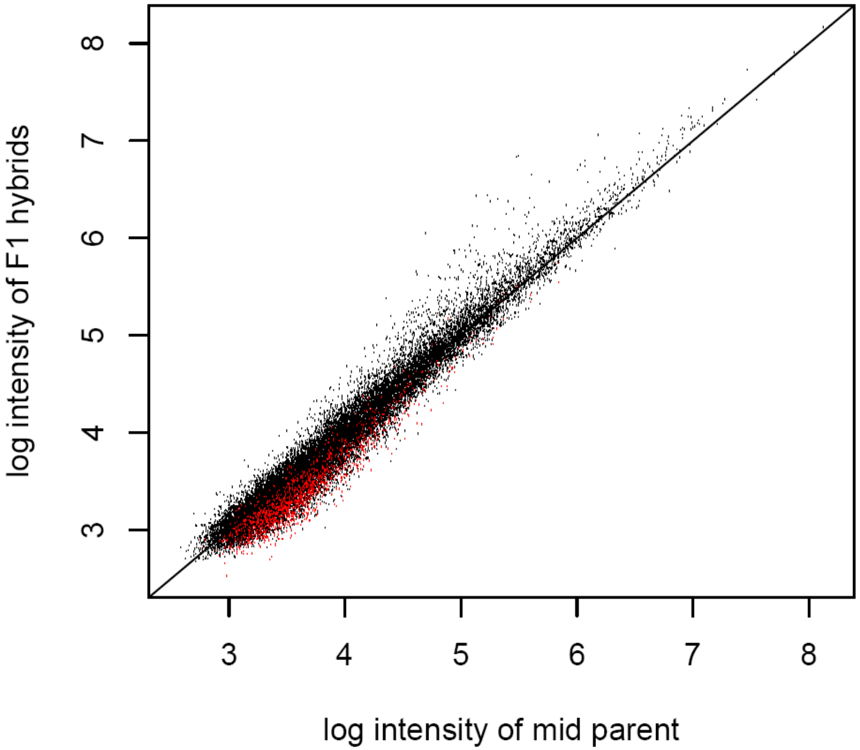

Supplemental Figure 3C. Scatter plot of the mean log intensities of F1 hybrids across sample replicates (y-axis) against that of mid-parent (x-axis) for SFP probes interrogating transcribed regions. The SFP probes detecting the top 458 Van ASE genes were colored red.

Supplemental Figure 4. Detection of differential exonic and intronic splicing.

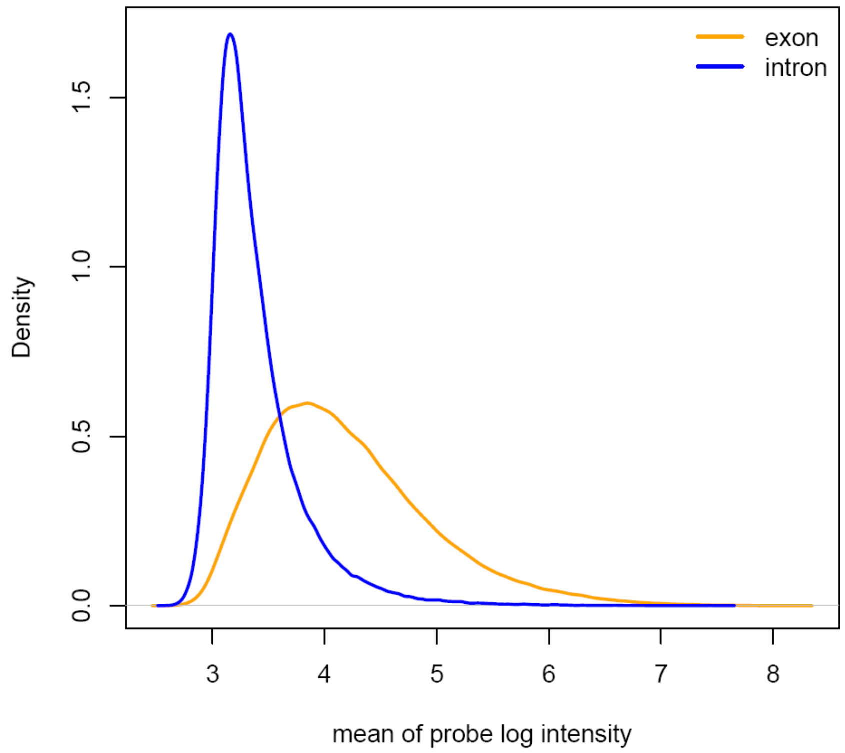

Supplemental Figure 4A. The mean probe log intensity distribution of all annotated exons (orange) and introns (blue). The x-axis is the mean exon/intron probe log intensity across four replicates for Col and Van.

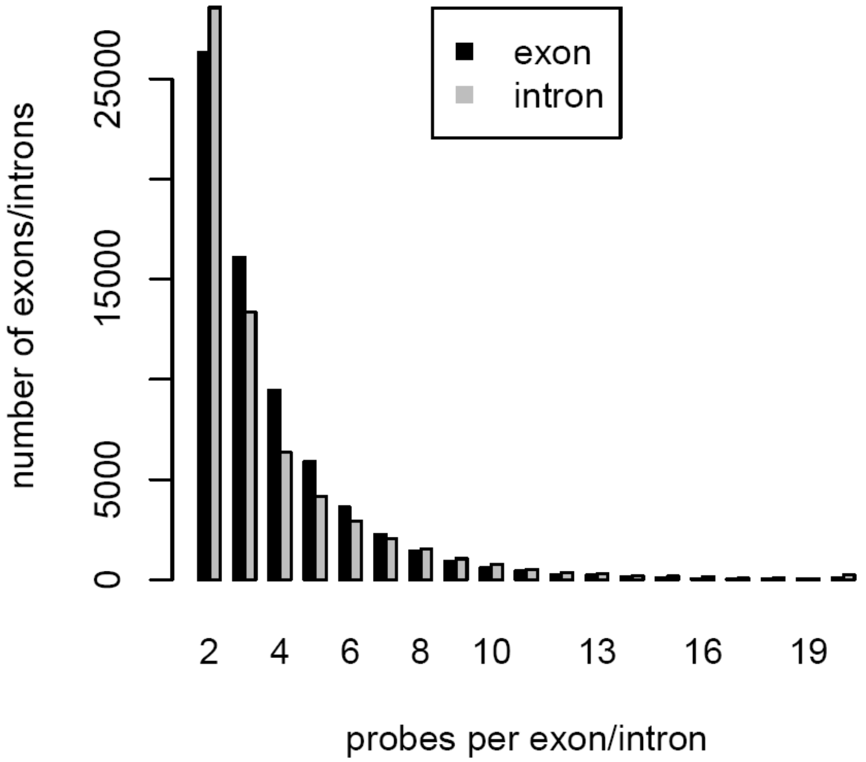

Supplemental Figure 4B. The distribution of the number of exon probes per exon (black bars) and the number of intron probes per intron (grey bars).

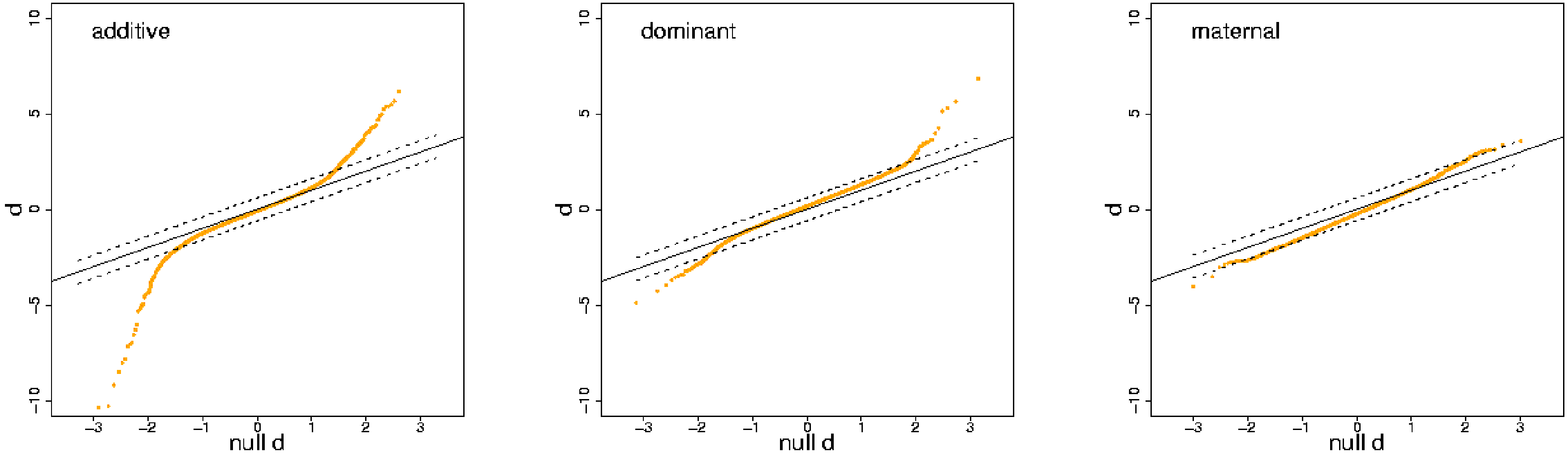

Supplemental Figure 4C. Quantile-quantile plots for the additive (left), dominant (middle) and maternal (right) terms of differential intron splicing. The real d scores (y-axis) were plotted against the null d scores (x-axis) obtained by 1,000 permutations.

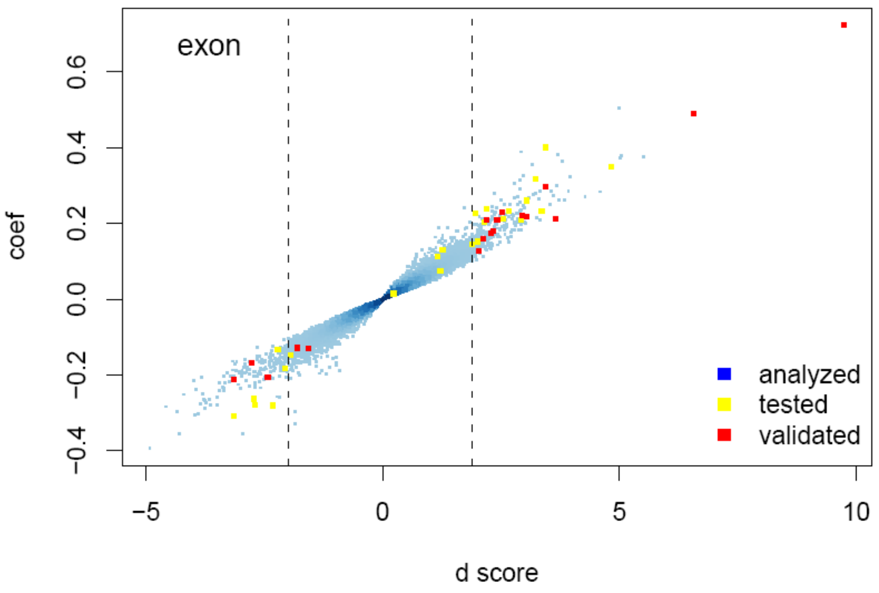

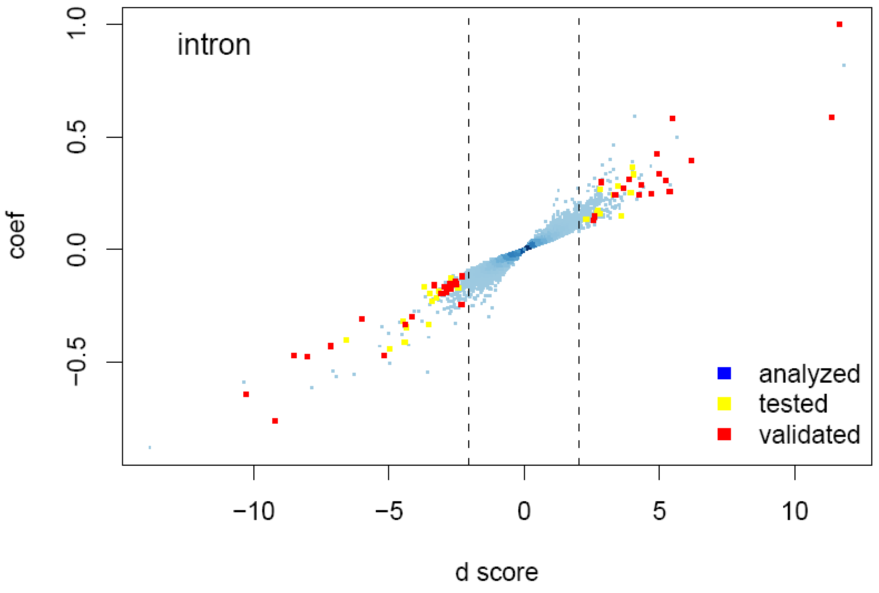

Supplemental Figure 4D. The distribution of regression coefficients (y-axis) and d scores (x-axis) of additive term for exonic (upper) and intronic (lower) splicing. Blue dots represent all exons/introns analyzed, yellow dots represent exons/introns tested by RT-PCR, red dots represent exon/introns suggested to be differentially spliced by RT-PCR. Dashed lines represent the d score thresholds to call the 477 significant exons and 459 significant introns.

Supplemental Figure 5. The effects of SFP probes on expression estimation.

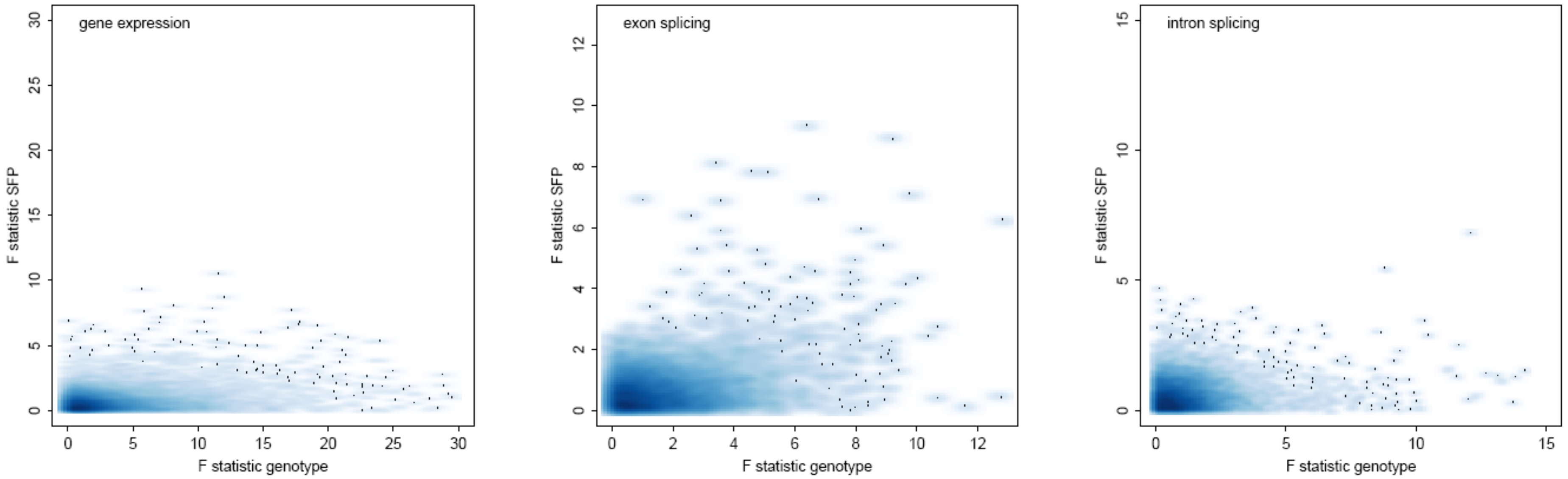

Supplemental Figure 5A. F statistics of SFP effects (y-axis) and that of genotype effects (x-axis) were obtained from ANOVA model: gene/exon/intron intensity = genotype + SFP + genotype × SFP + error, for gene expression (left), exonic splicing (middle) and intronic splicing (right).

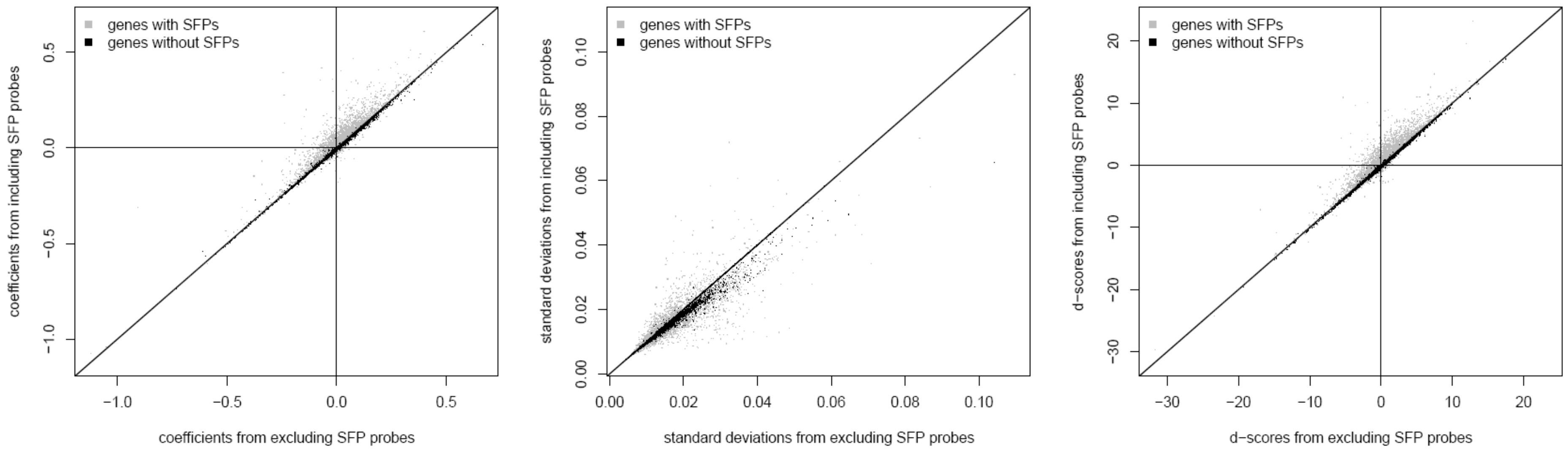

Supplemental Figure 5B. The distribution of coefficients (left), standard deviations (middle) and d scores (right) obtained from gene expression analysis including SFP probes (y-axis) were plotted against those obtained from analysis excluding SFP probes (x-axis). Grey dots: genes containing SFPs; black dots: genes without SFPs.

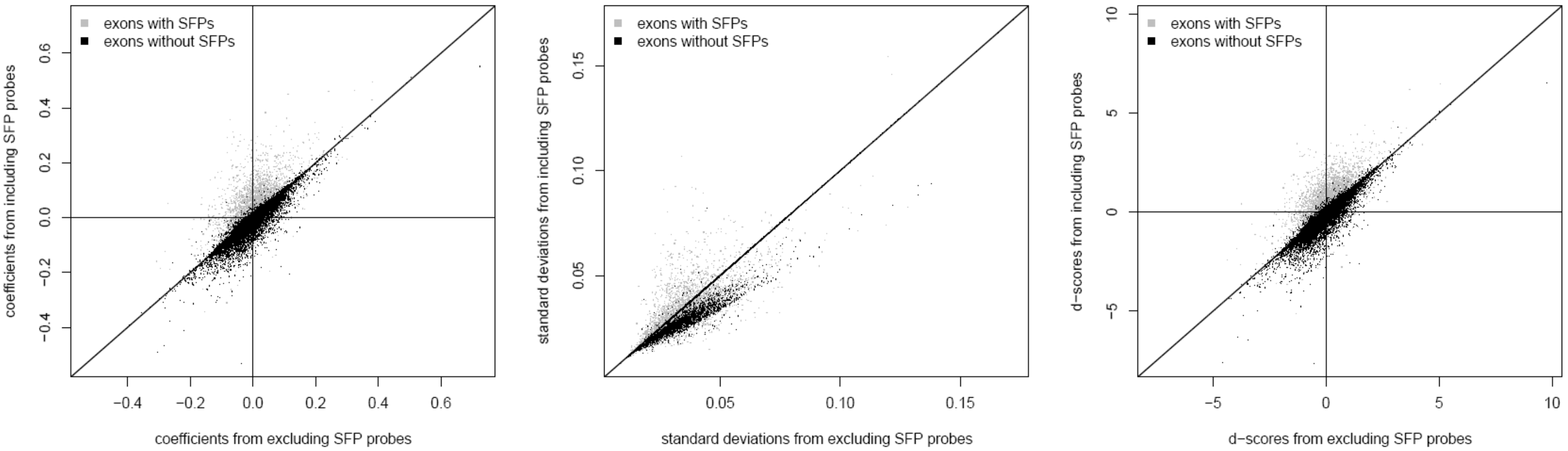

Supplemental Figure 5C. The distribution of coefficients (left), standard deviations (middle) and d scores (right) obtained from exonic splicing analysis including SFP probes (y-axis) were plotted against those obtained from analysis excluding SFP probes (x-axis). Grey dots: exons containing SFPs; black dots: exons without SFPs.

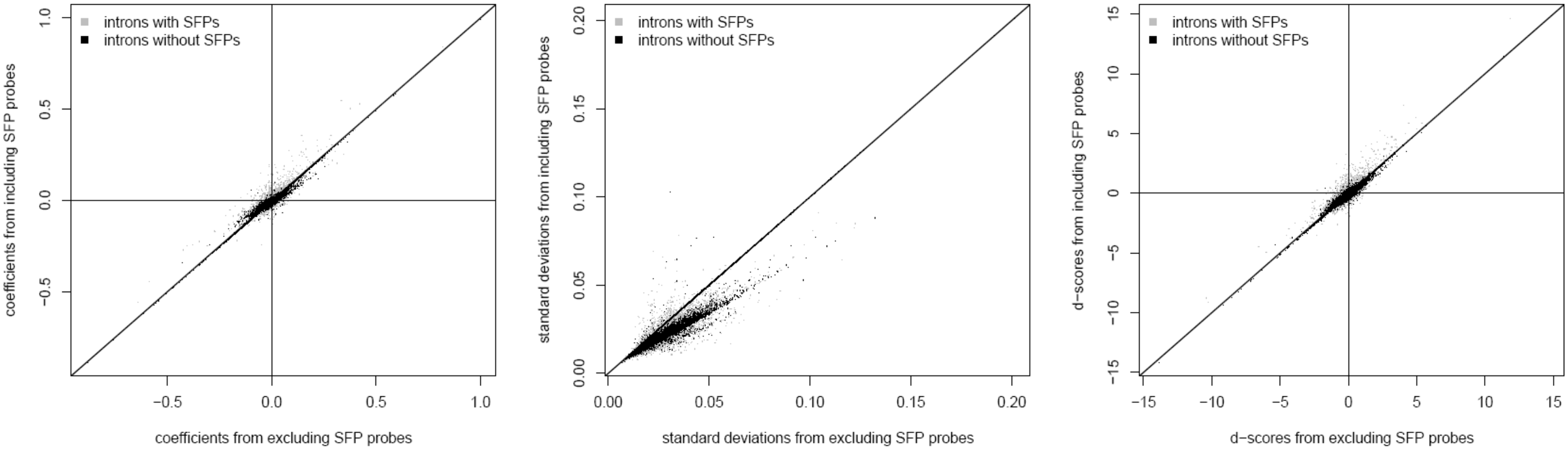

Supplemental Figure 5D. The distribution of coefficients (left), standard deviations (middle) and d scores (right) obtained from intronic splicing analysis including SFP probes (y-axis) were plotted against those obtained from analysis excluding SFP probes (x-axis). Grey dots: introns containing SFPs; black dots: introns without SFPs.

Supplemental Figure 6. Parameterization of the hidden Markov model using a modified Baum-Welch algorithm.

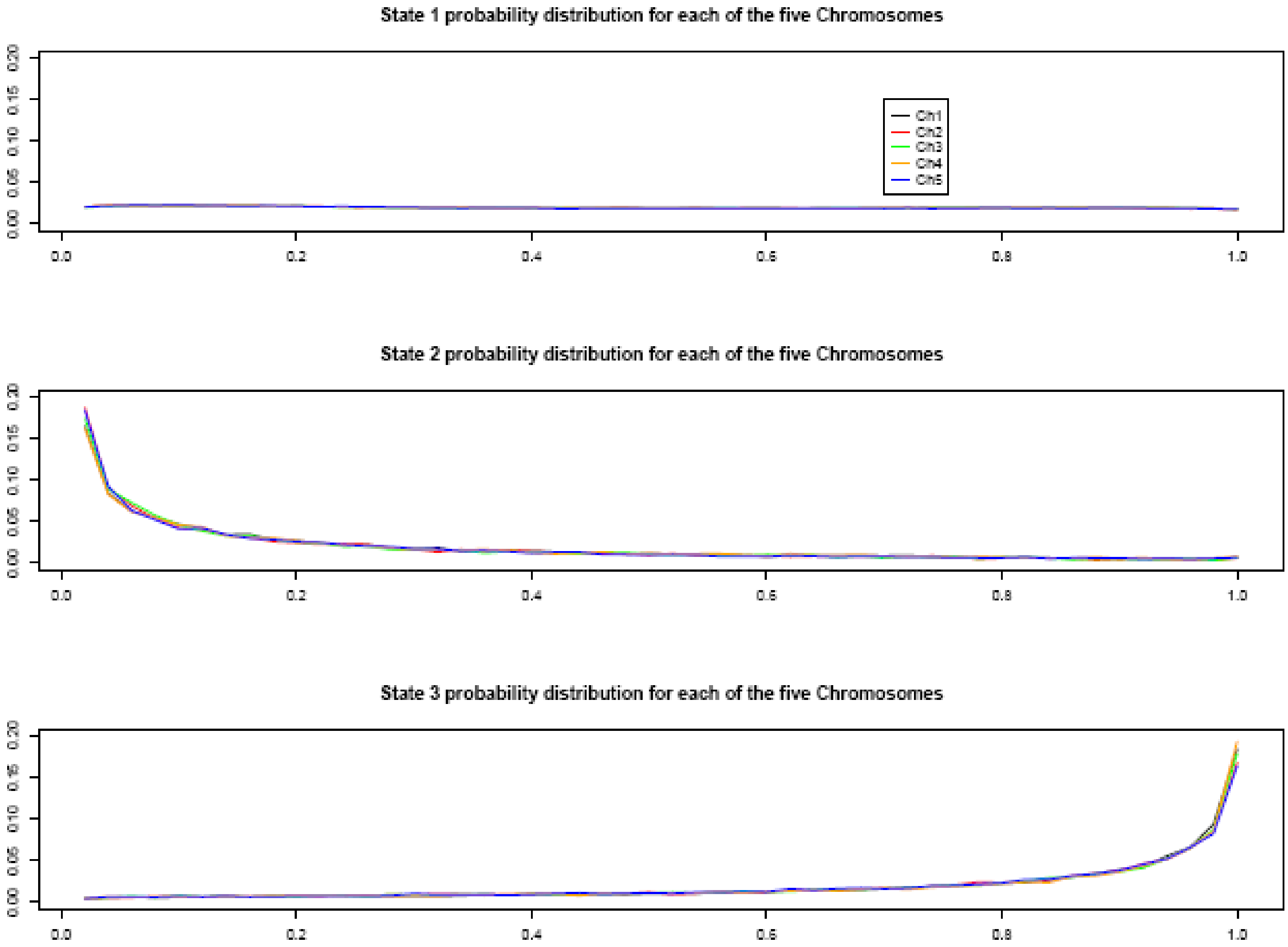

Supplemental Figure 6A. The emission probability distributions for state 1 (top), state 2 (middle), and state 3 (bottom) for each of five chromosomes.

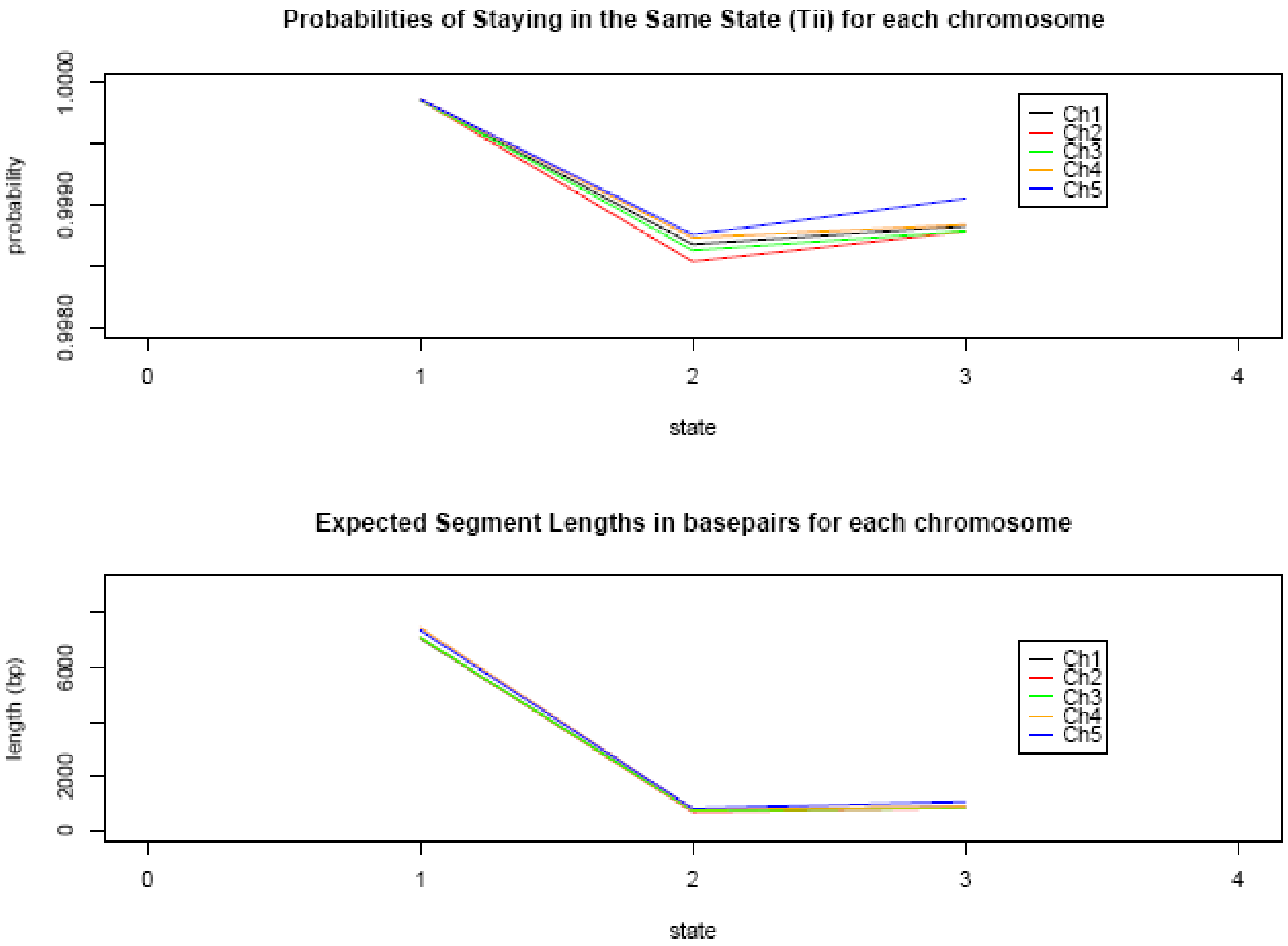

Supplemental Figure 6B. The three estimated transition parameters t_{ii} in the base transition matrix T for each of five chromosomes (top). Higher rate of transitions to other states from state 2 and state 3 than from state 1 was observed. Given these parameters, we can calculate the expected segment lengths for each state for each of five chromosomes (bottom).
