# Supplementary material for: Whole genome transcriptome polymorphisms in Arabidopsis thaliana
Source: Genome Biol. 2008 Nov 24;9(11):R165. doi: 10.1186/gb-2008-9-11-r165 (PMC2614497; doi:10.1186/gb-2008-9-11-r165)
Supplement: Additional data file 3 — Gene plots and gel pictures for splicing validation. [file gb-2008-9-11-r165-S3.pdf]

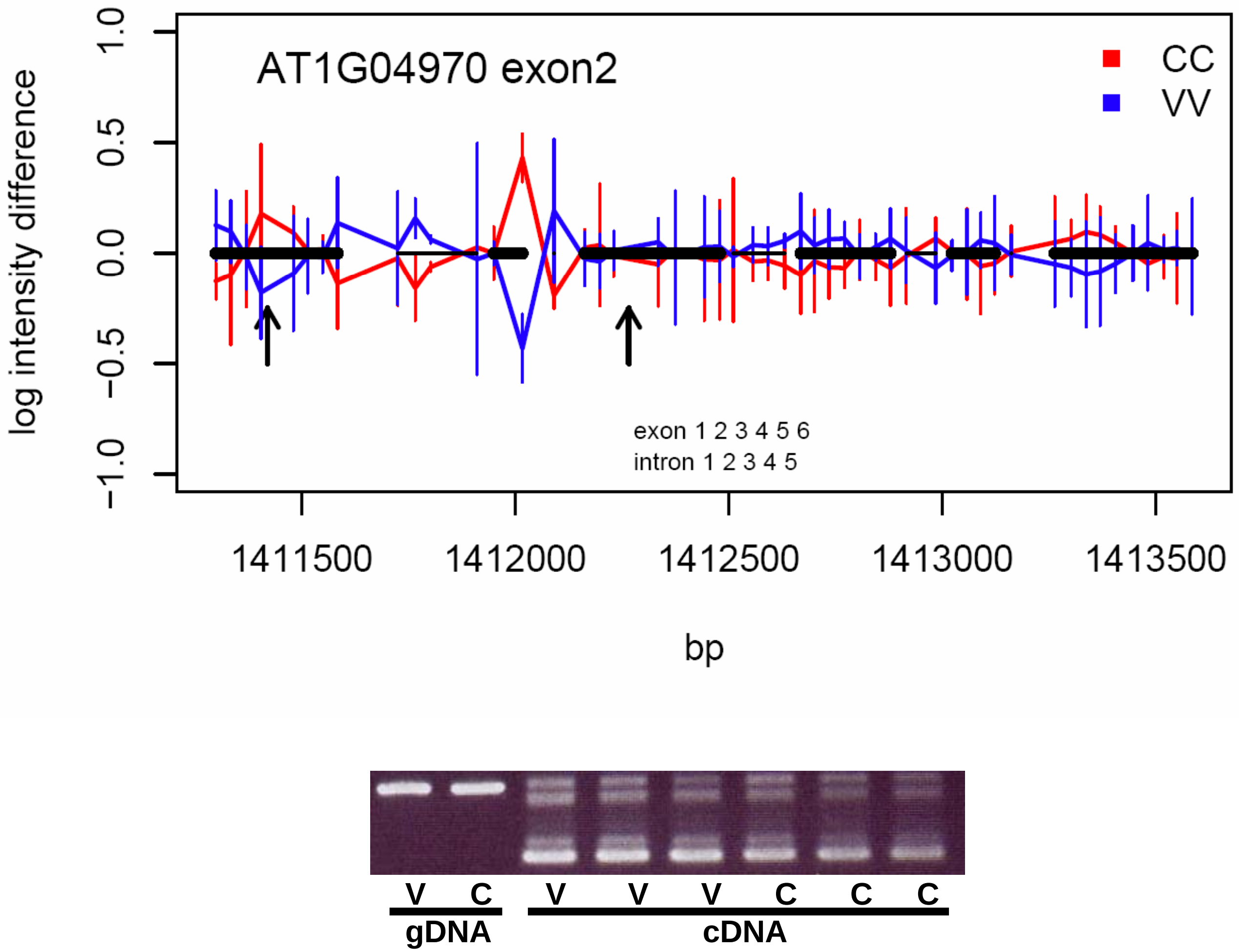
AT1G04970 exon2
CC
VV
log intensity difference
1.0
0.5
0.0
−0.5
−1.0
exon 1 2 3 4 5 6
intron 1 2 3 4 5
1411500
1412000
1412500
1413000
1413500
bp
V C V V V C C C
gDNA
cDNA

√

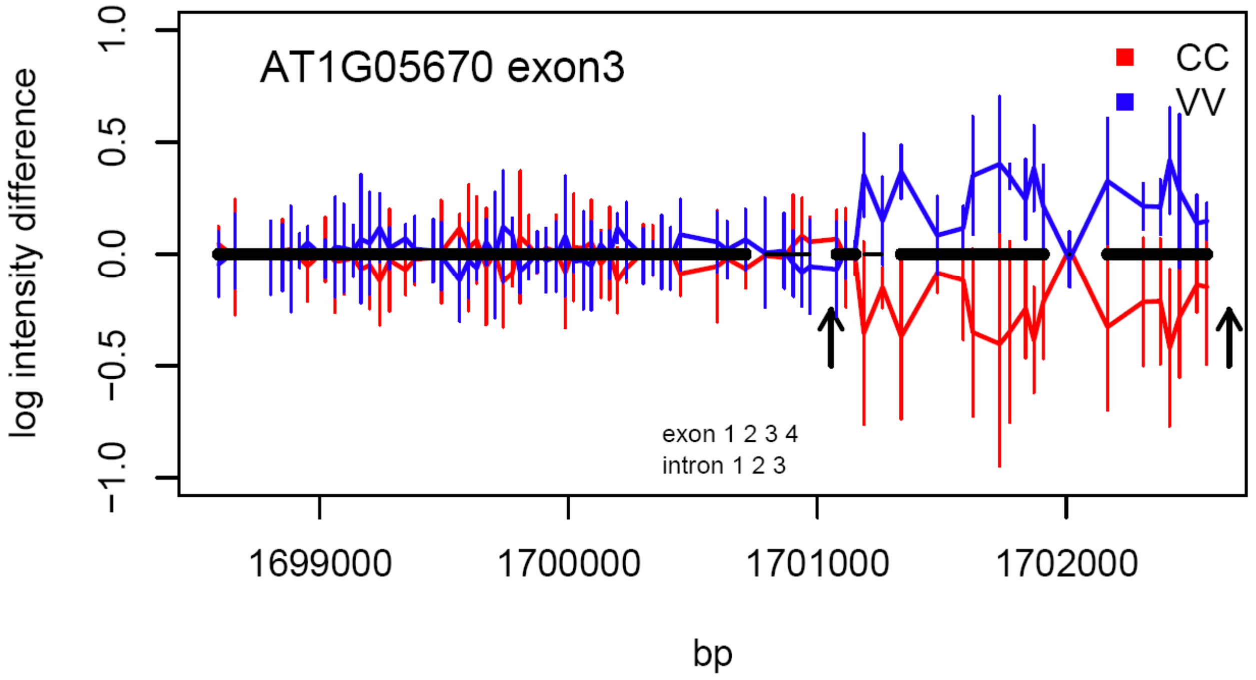

AT1G05670 exon3
CC
VV
log intensity difference
1.0
0.5
0.0
−0.5
−1.0
exon 1 2 3 4
intron 1 2 3
1699000
1700000
1701000
1702000
bp

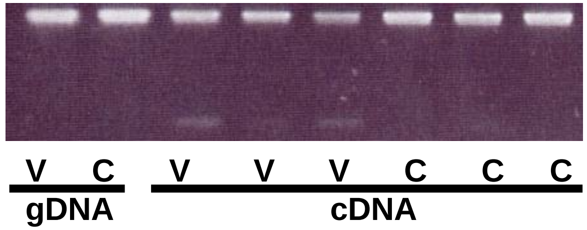

V C V V V C C C
gDNA
cDNA

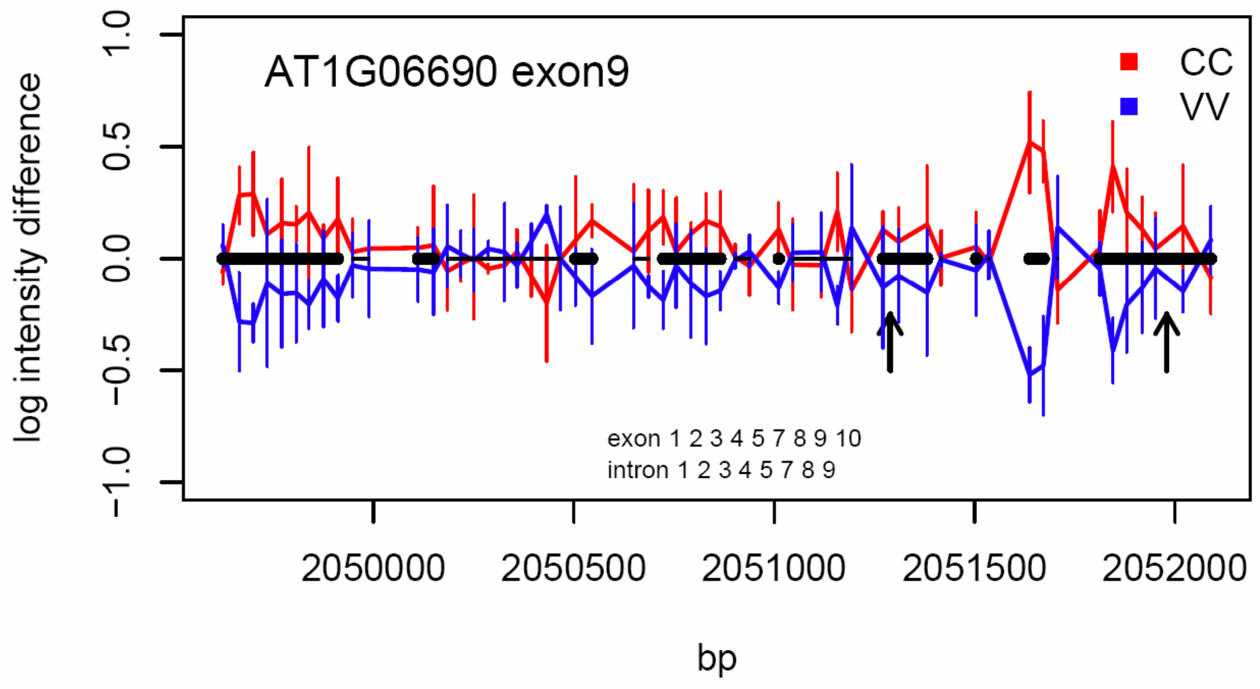
AT1G06690 exon9
CC
VV
log intensity difference
1.0
0.5
0.0
−0.5
−1.0
exon 1 2 3 4 5 7 8 9 10
intron 1 2 3 4 5 7 8 9
2050000
2050500
2051000
2051500
2052000
bp

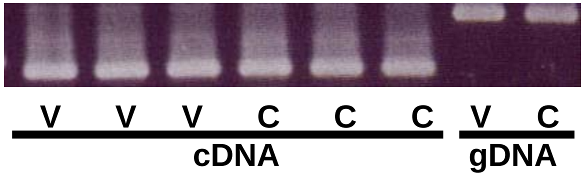
V V V C C C V C
cDNA
gDNA

√

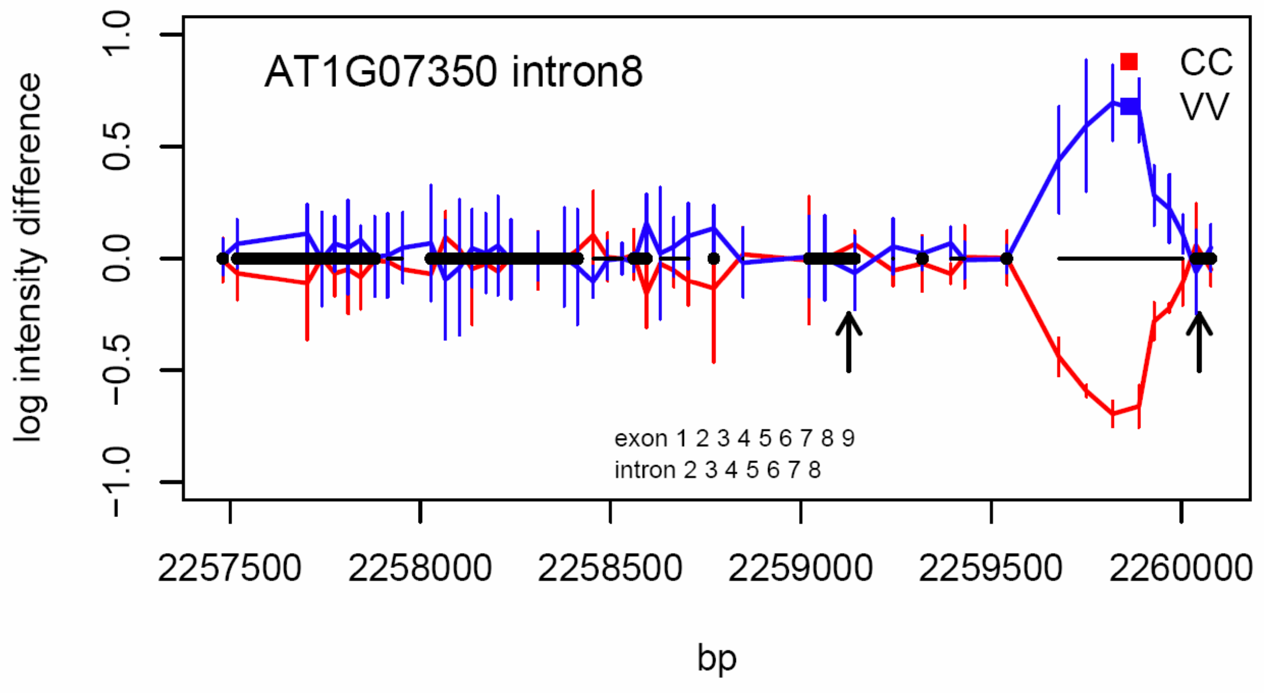
AT1G07350 intron8
CC
VV
log intensity difference
1.0
0.5
0.0
-0.5
-1.0
exon 1 2 3 4 5 6 7 8 9
intron 2 3 4 5 6 7 8
2257500
2258000
2258500
2259000
2259500
2260000
bp

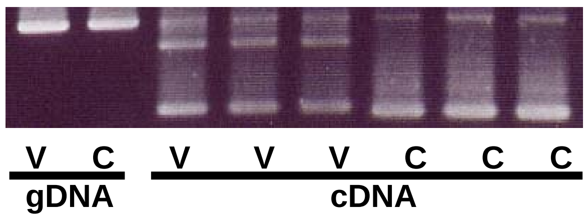
V C V V V C C C
gDNA
cDNA

√

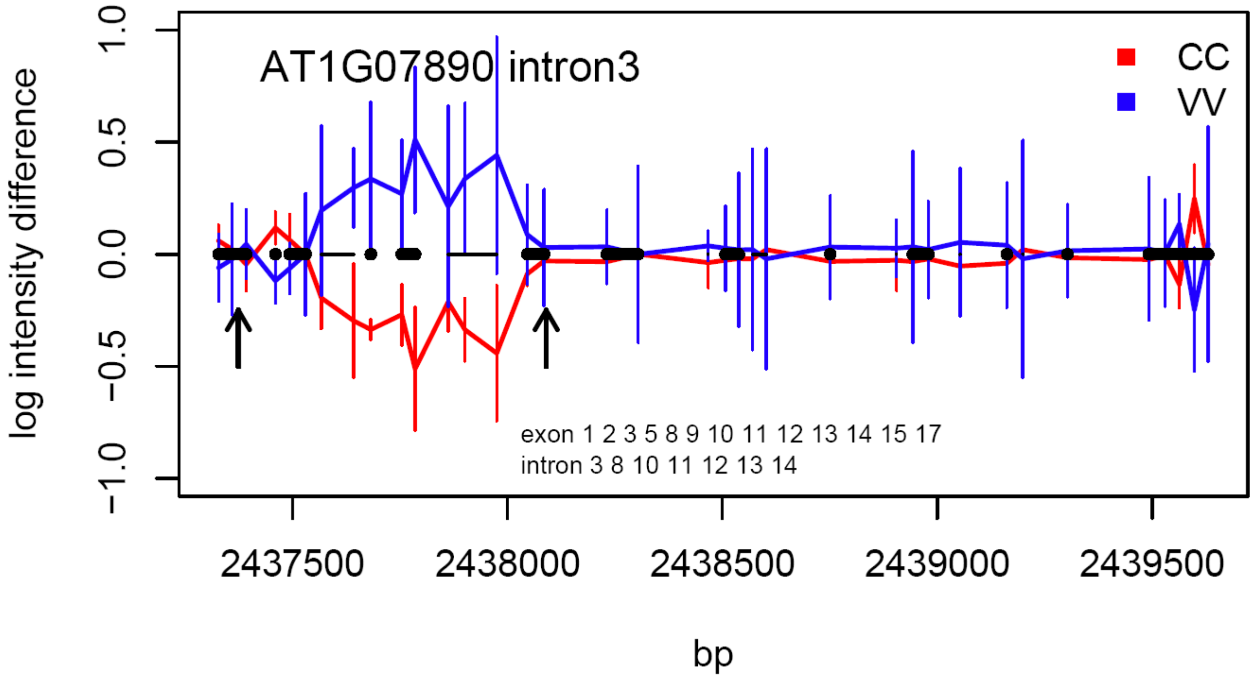
AT1G07890 intron3
CC
VV
log intensity difference
1.0
0.5
0.0
−0.5
−1.0
exon 1 2 3 5 8 9 10 11 12 13 14 15 17
intron 3 8 10 11 12 13 14
2437500
2438000
2438500
2439000
2439500
bp

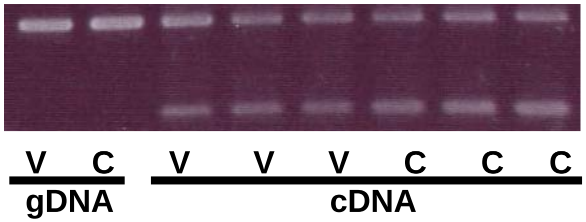
V C V V V C C C
gDNA
cDNA

√

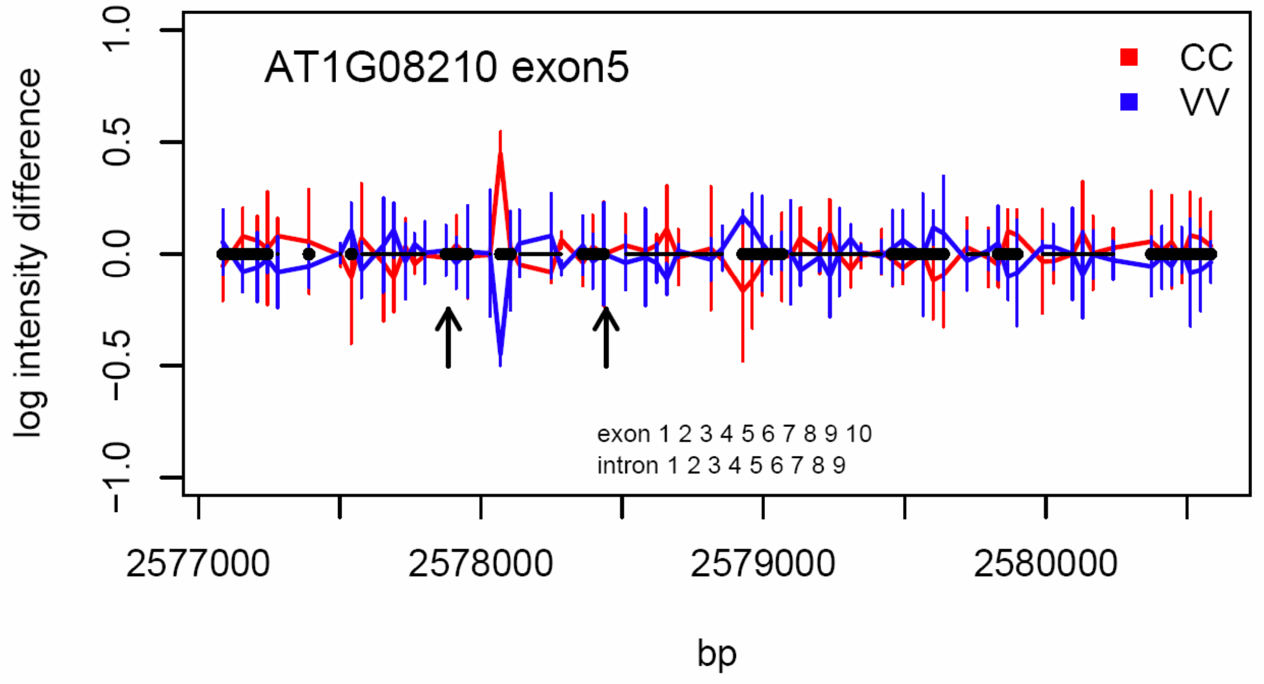

AT1G08210 exon5
CC
VV
log intensity difference
1.0
0.5
0.0
-0.5
-1.0
exon 1 2 3 4 5 6 7 8 9 10
intron 1 2 3 4 5 6 7 8 9
2577000
2578000
2579000
2580000
bp

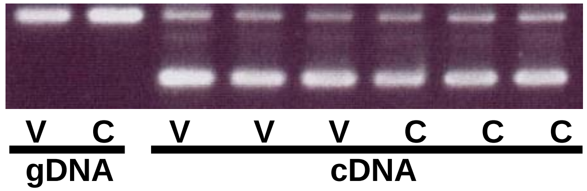

V C V V V C C C
gDNA
cDNA

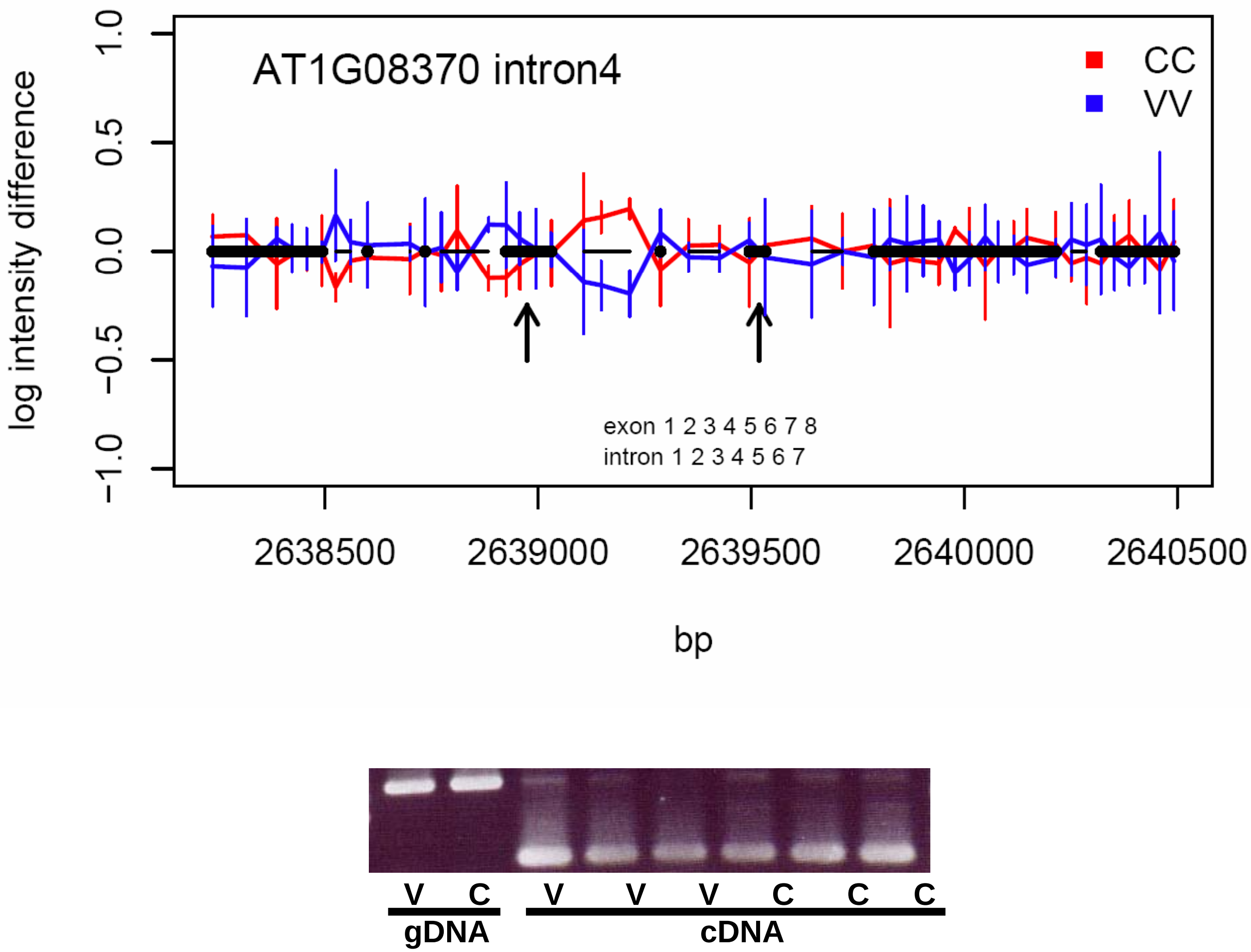
AT1G08370 intron4
CC
VV
log intensity difference
1.0
0.5
0.0
−0.5
−1.0
exon 1 2 3 4 5 6 7 8
intron 1 2 3 4 5 6 7
2638500
2639000
2639500
2640000
2640500
bp
V C V V V C C C
gDNA
cDNA

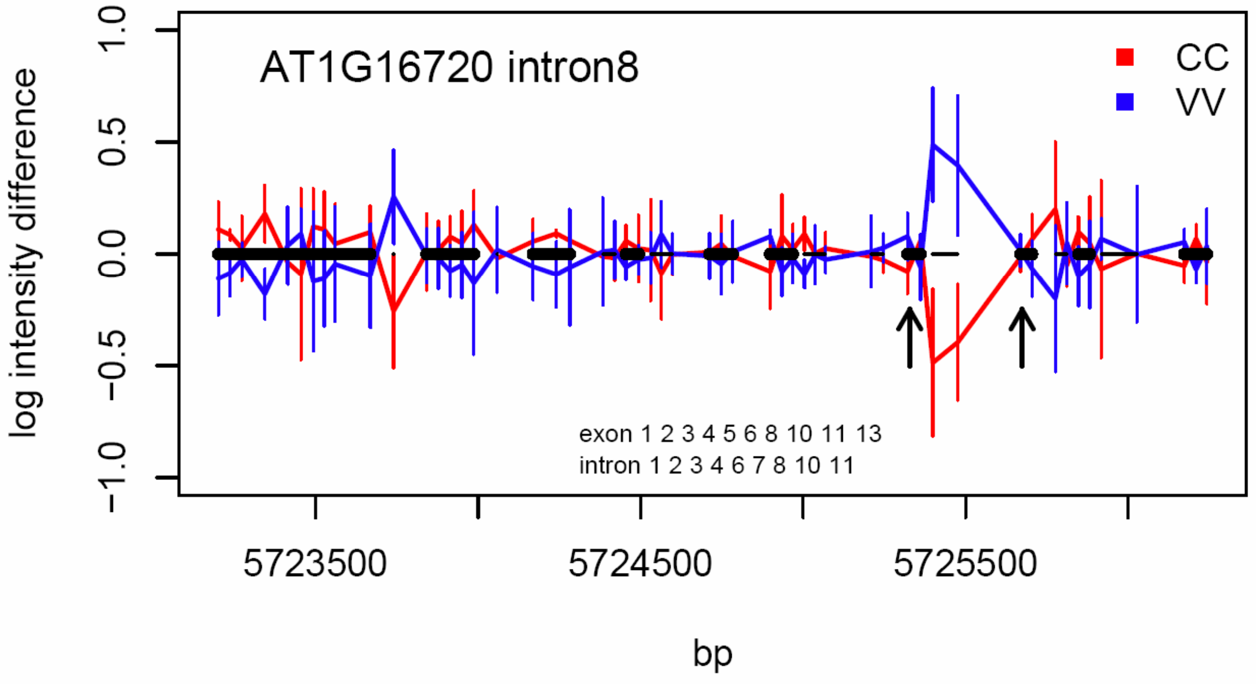
AT1G16720 intron8
CC
VV
log intensity difference
1.0
0.5
0.0
−0.5
−1.0
exon 1 2 3 4 5 6 8 10 11 13
intron 1 2 3 4 6 7 8 10 11
5723500
5724500
5725500
bp

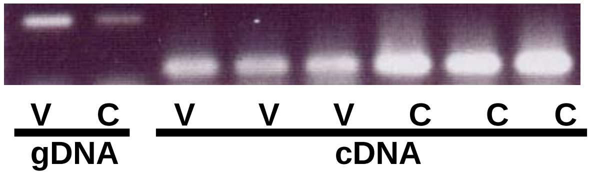
V C V V V C C C
gDNA
cDNA

√

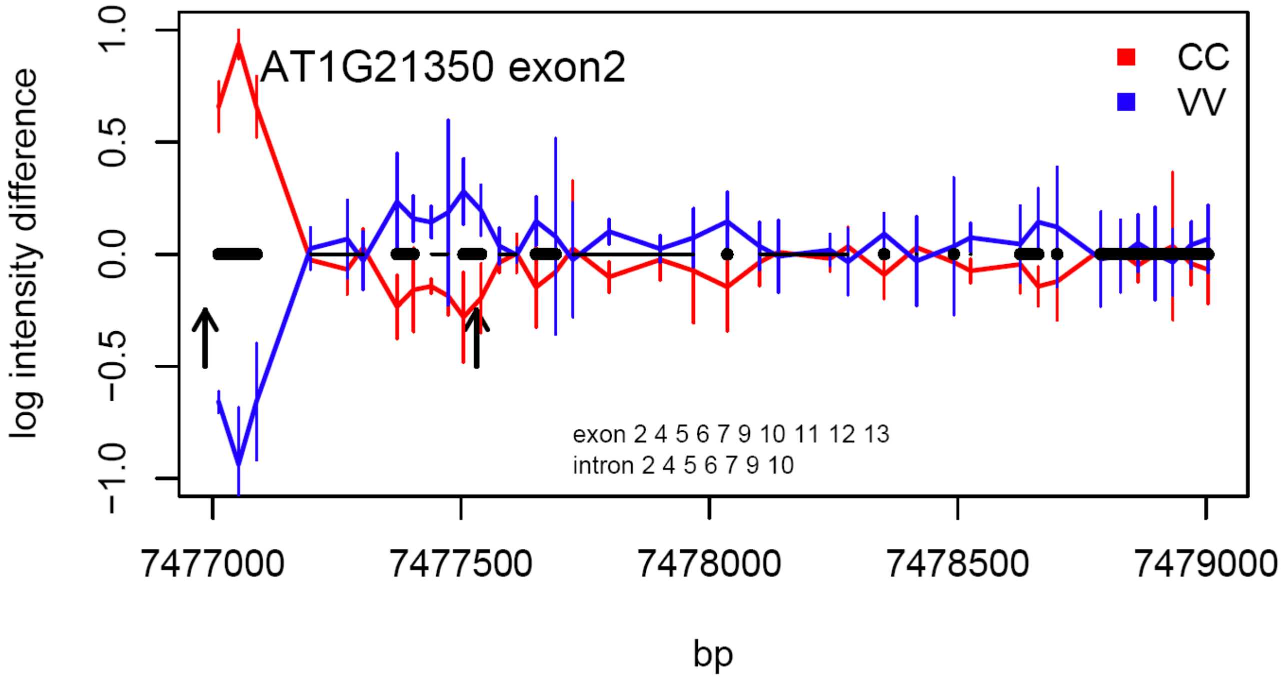
AT1G21350 exon2
CC
VV
log intensity difference
1.0
0.5
0.0
−0.5
−1.0
exon 2 4 5 6 7 9 10 11 12 13
intron 2 4 5 6 7 9 10
7477000
7477500
7478000
7478500
7479000
bp

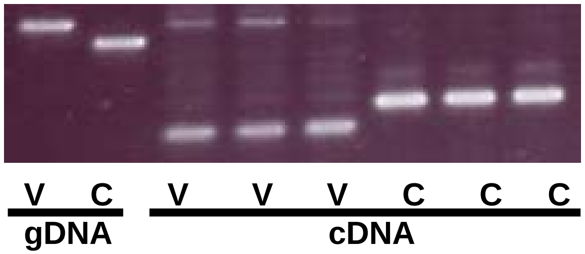
V C V V V C C C
gDNA
cDNA

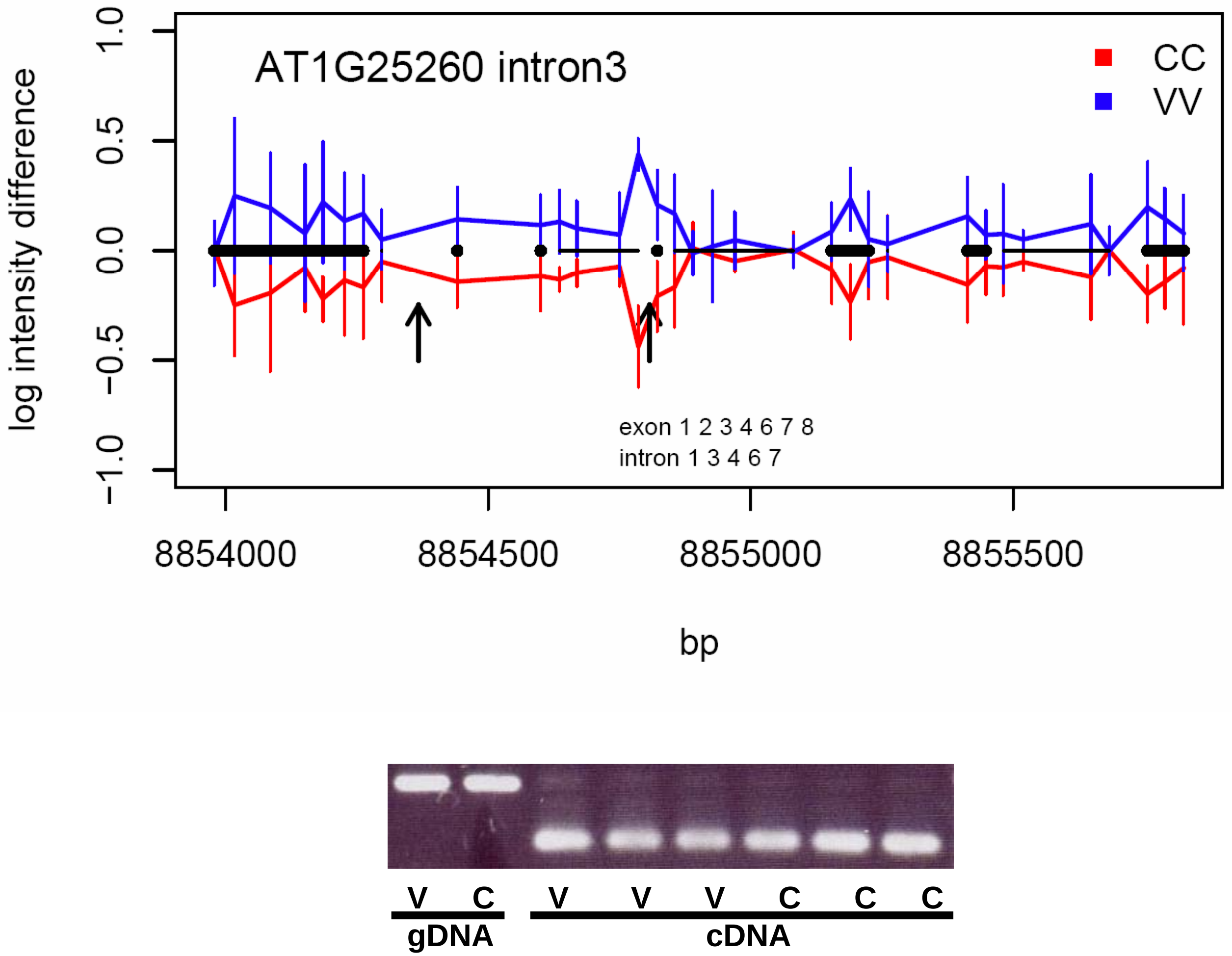

AT1G25260 intron3
CC
VV
log intensity difference
1.0
0.5
0.0
−0.5
−1.0
exon 1 2 3 4 6 7 8
intron 1 3 4 6 7
8854000
8854500
8855000
8855500
bp
V C V V V C C C
gDNA
cDNA

√

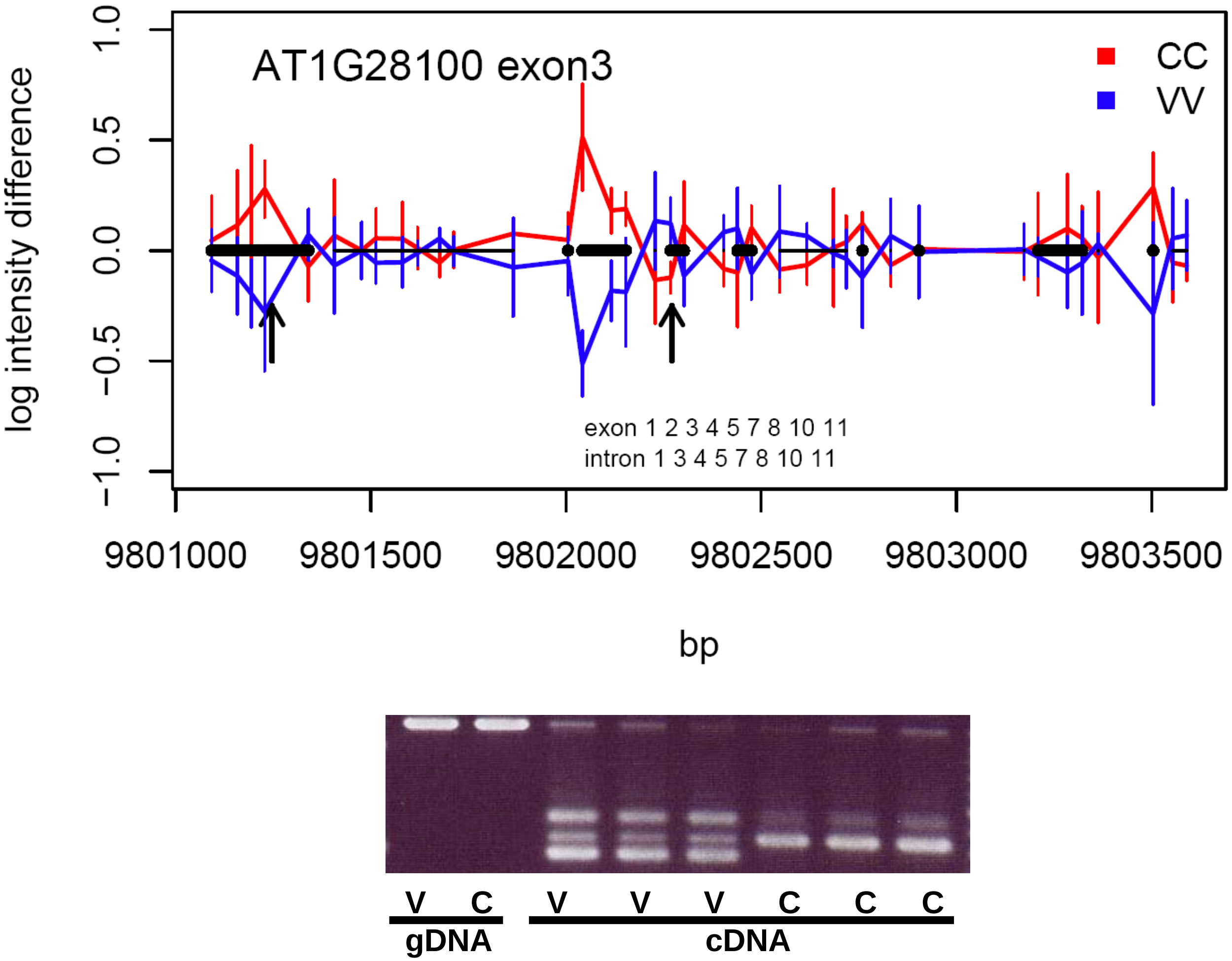
AT1G28100 exon3
CC
VV
log intensity difference
1.0
0.5
0.0
−0.5
−1.0
exon 1 2 3 4 5 7 8 10 11
intron 1 3 4 5 7 8 10 11
9801000
9801500
9802000
9802500
9803000
9803500
bp
V C V V V C C C
gDNA
cDNA

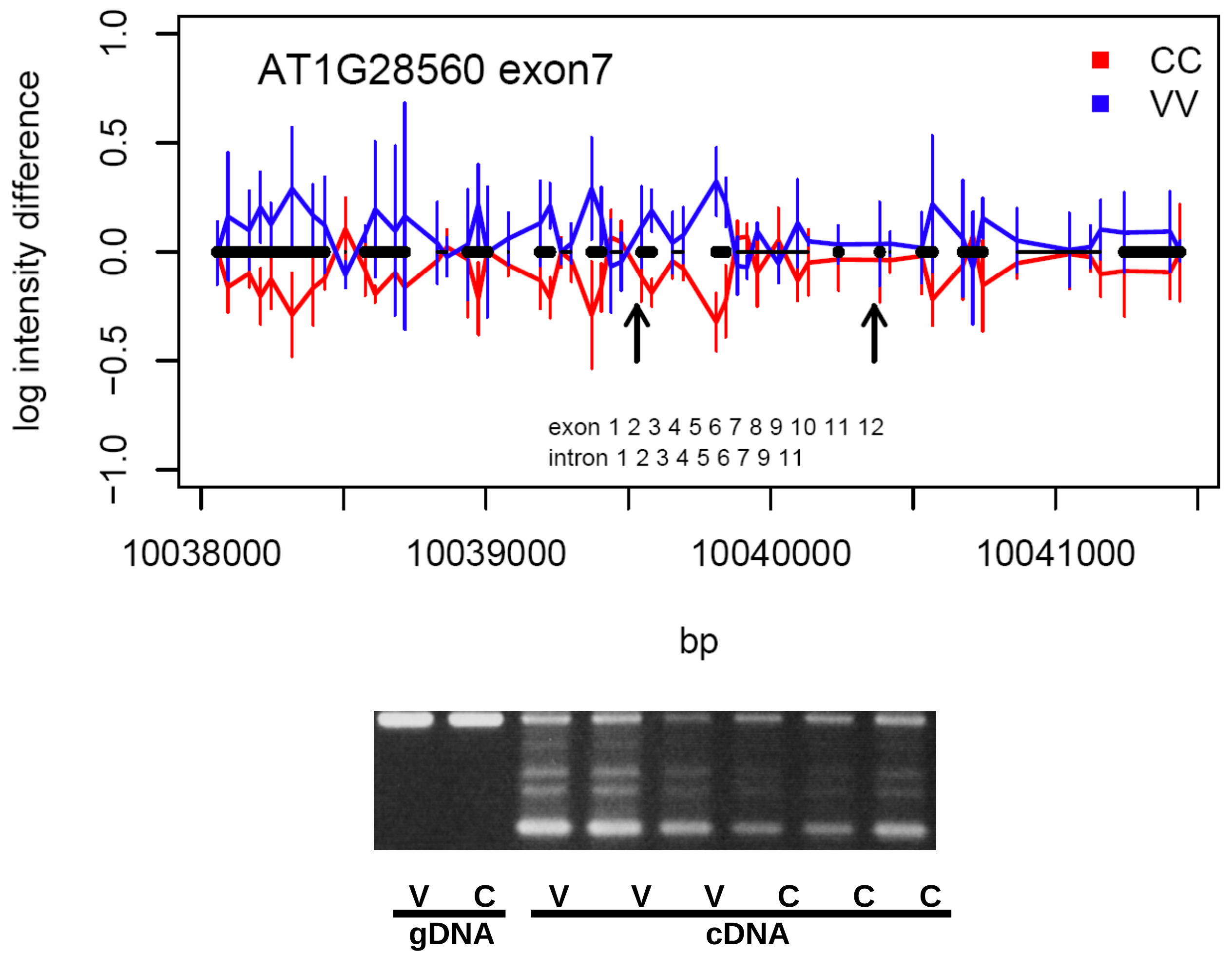
AT1G28560 exon7
CC
VV
log intensity difference
1.0
0.5
0.0
−0.5
−1.0
exon 1 2 3 4 5 6 7 8 9 10 11 12
intron 1 2 3 4 5 6 7 9 11
10038000
10039000
10040000
10041000
bp
V C V V V C C C
gDNA
cDNA

√

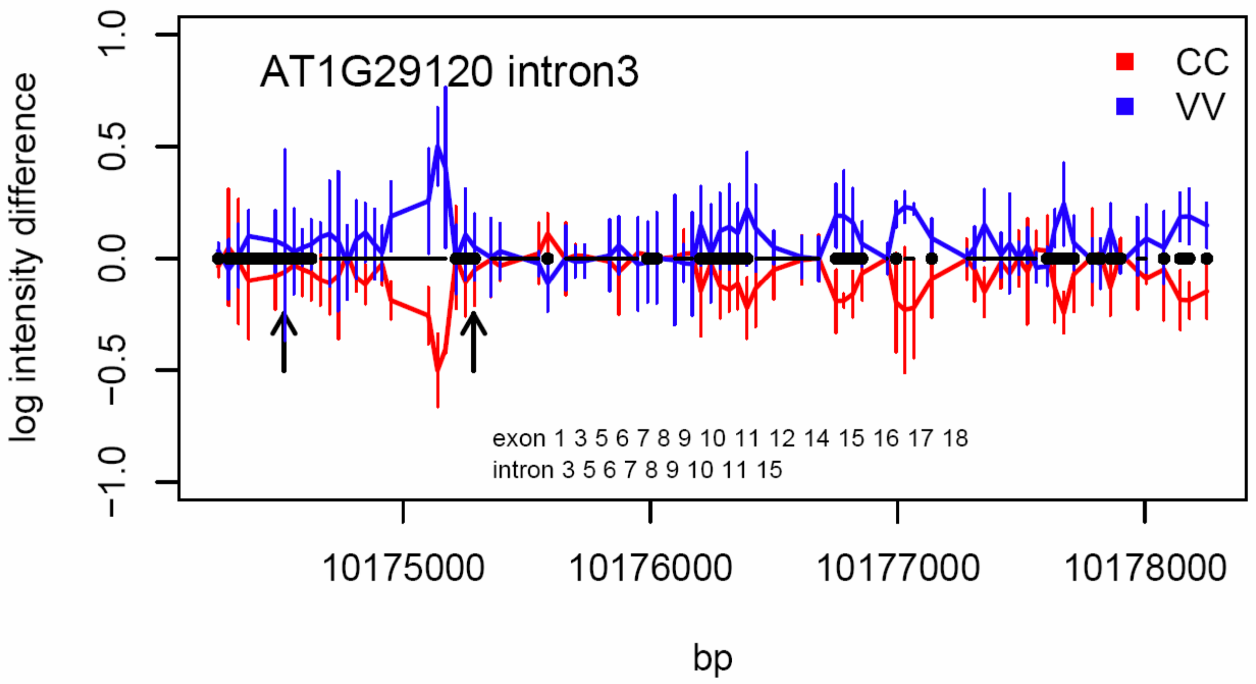
AT1G29120 intron3
CC
VV
log intensity difference
1.0
0.5
0.0
−0.5
−1.0
exon 1 3 5 6 7 8 9 10 11 12 14 15 16 17 18
intron 3 5 6 7 8 9 10 11 15
10175000
10176000
10177000
10178000
bp

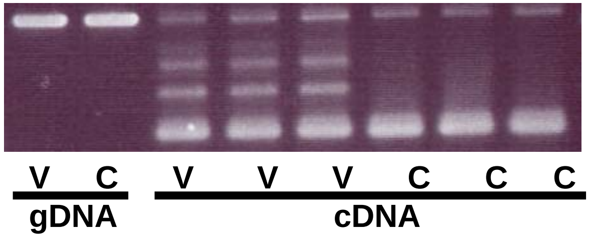
V C V V V C C C
gDNA
cDNA

√

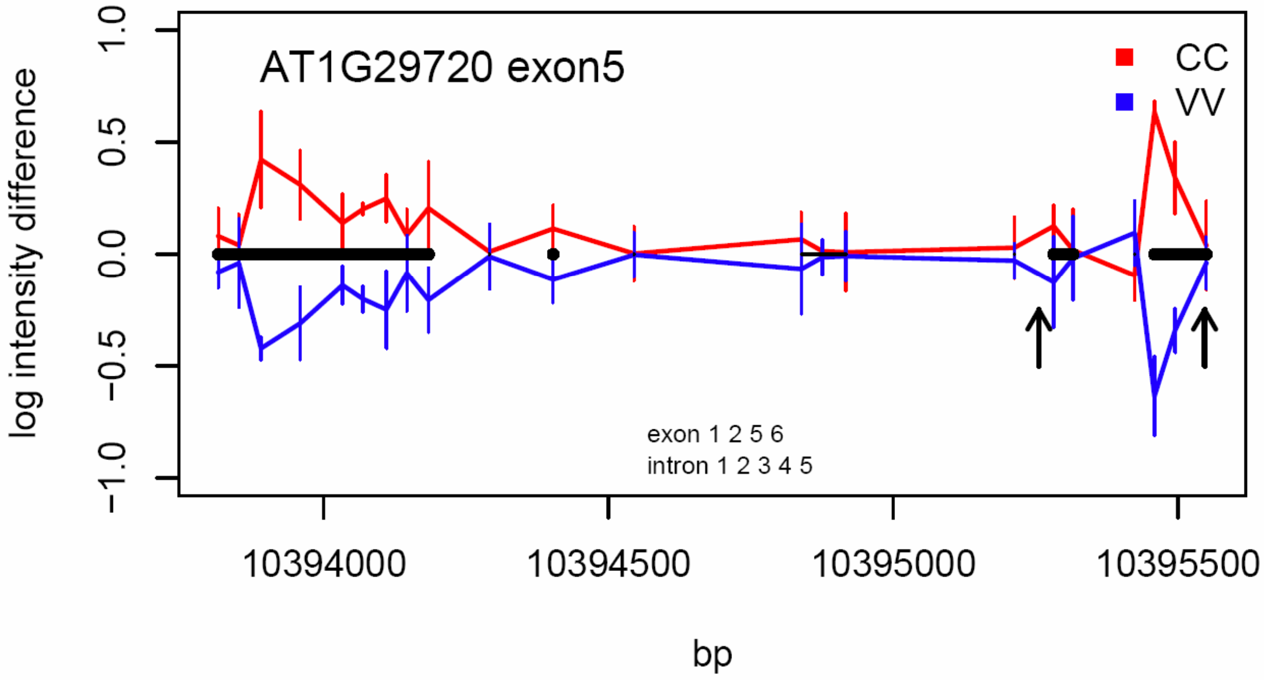

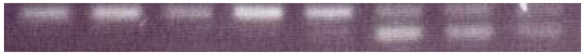

V C V V V C C C
gDNA cDNA

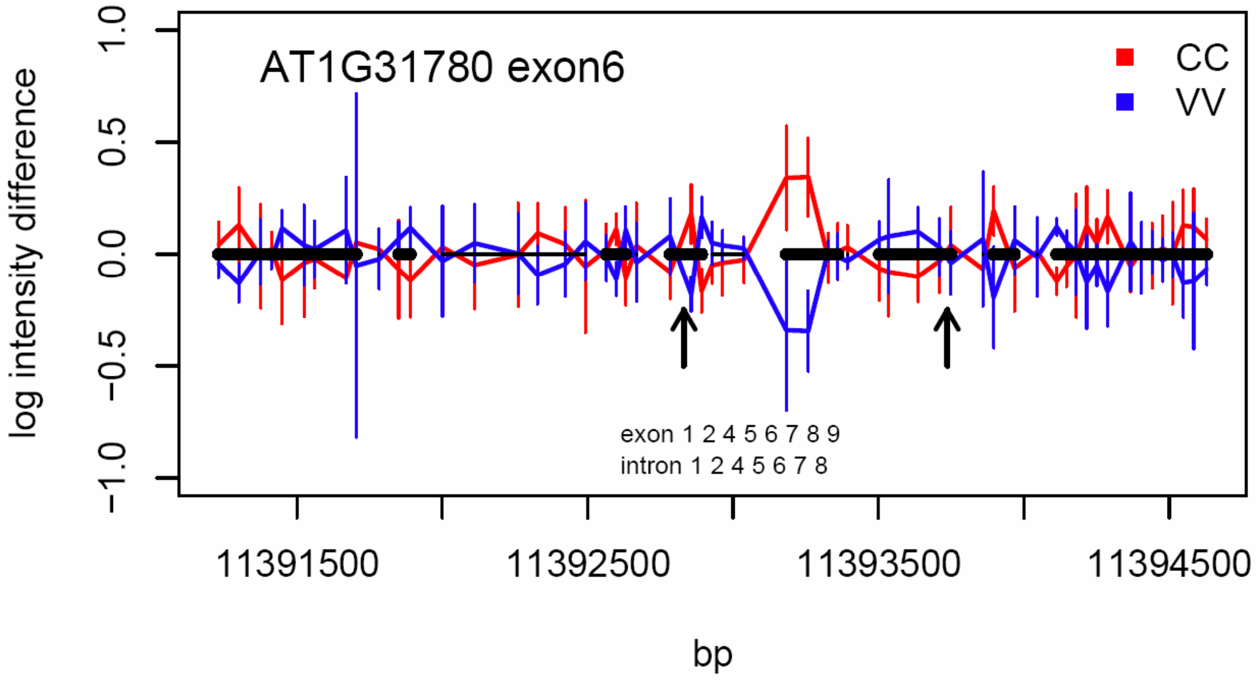
AT1G31780 exon6
CC
VV
log intensity difference
1.0
0.5
0.0
−0.5
−1.0
exon 1 2 4 5 6 7 8 9
intron 1 2 4 5 6 7 8
11391500
11392500
11393500
11394500
bp

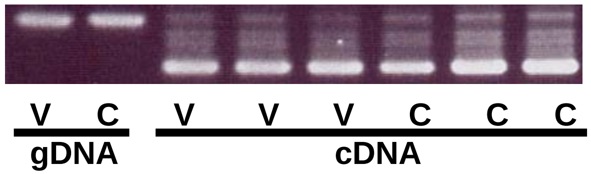
V C V V V C C C
gDNA
cDNA

√

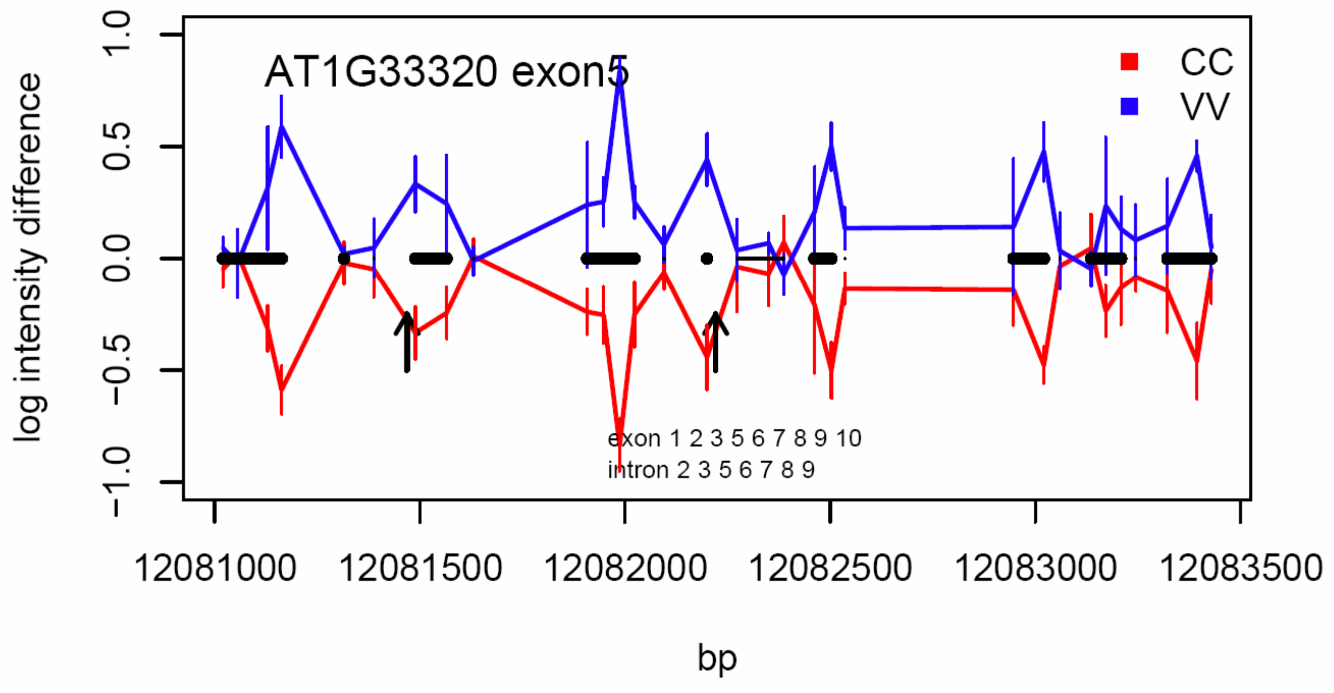
AT1G33320 exon5
CC
VV
log intensity difference
1.0
0.5
0.0
−0.5
−1.0
exon 1 2 3 5 6 7 8 9 10
intron 2 3 5 6 7 8 9
12081000
12081500
12082000
12082500
12083000
12083500
bp

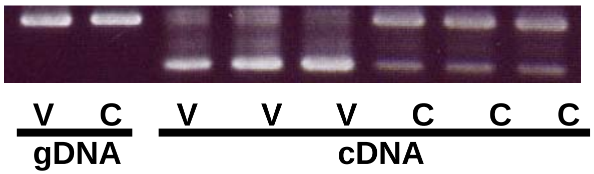
V C V V V C C C
gDNA
cDNA

Not included for analysis

√

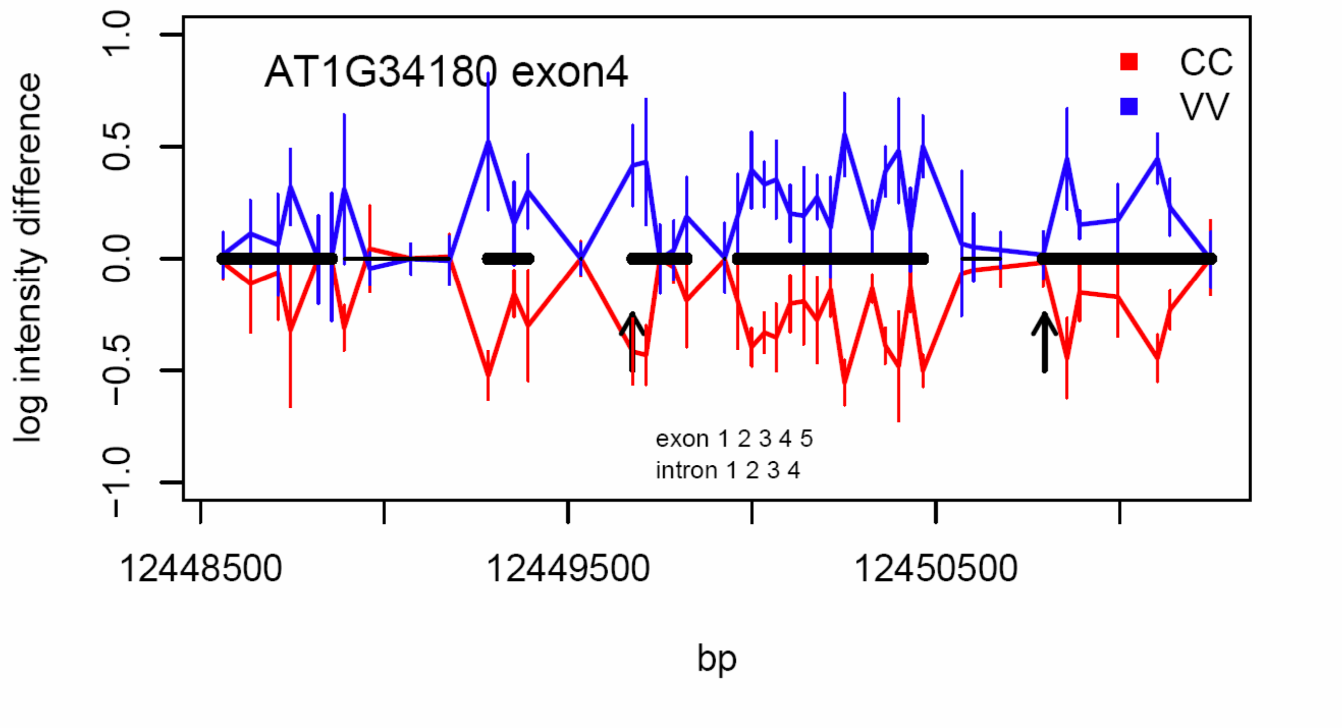

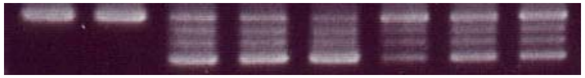

V C V V V C C C

gDNA cDNA

√

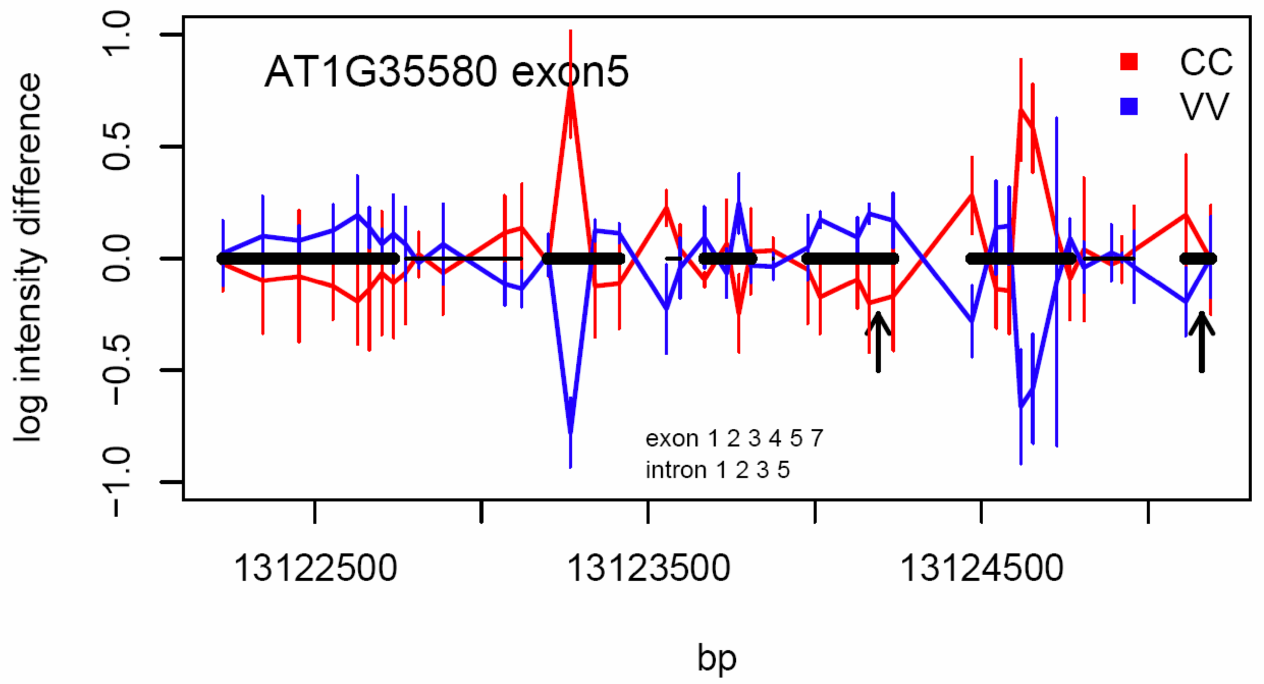

AT1G35580 exon5
CC
VV
log intensity difference
1.0
0.5
0.0
−0.5
−1.0
exon 1 2 3 4 5 7
intron 1 2 3 5
13122500
13123500
13124500
bp

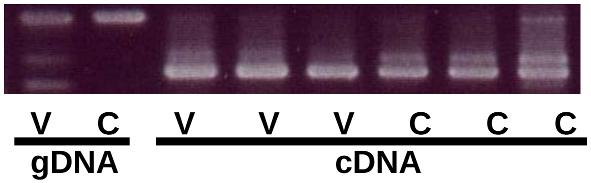

V C V V V C C C
gDNA
cDNA

√

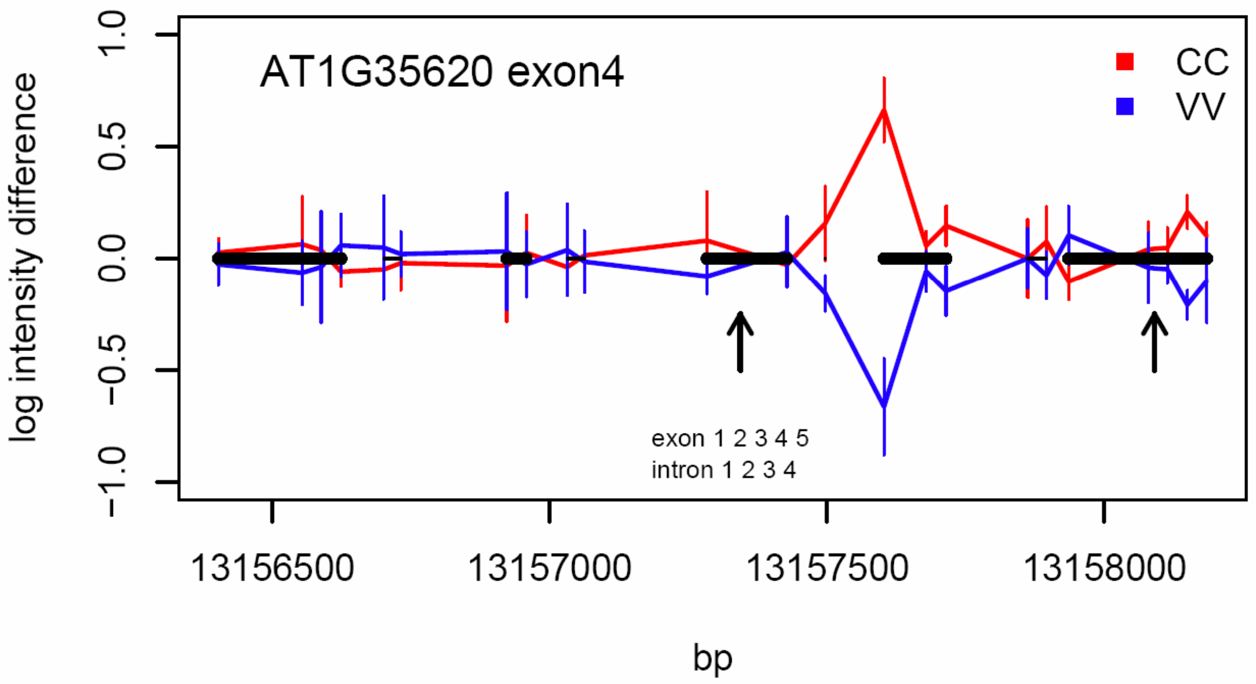
AT1G35620 exon4
CC
VV
log intensity difference
1.0
0.5
0.0
−0.5
−1.0
exon 1 2 3 4 5
intron 1 2 3 4
13156500
13157000
13157500
13158000
bp

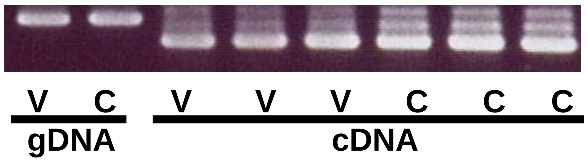
V C V V V C C C
gDNA
cDNA

√

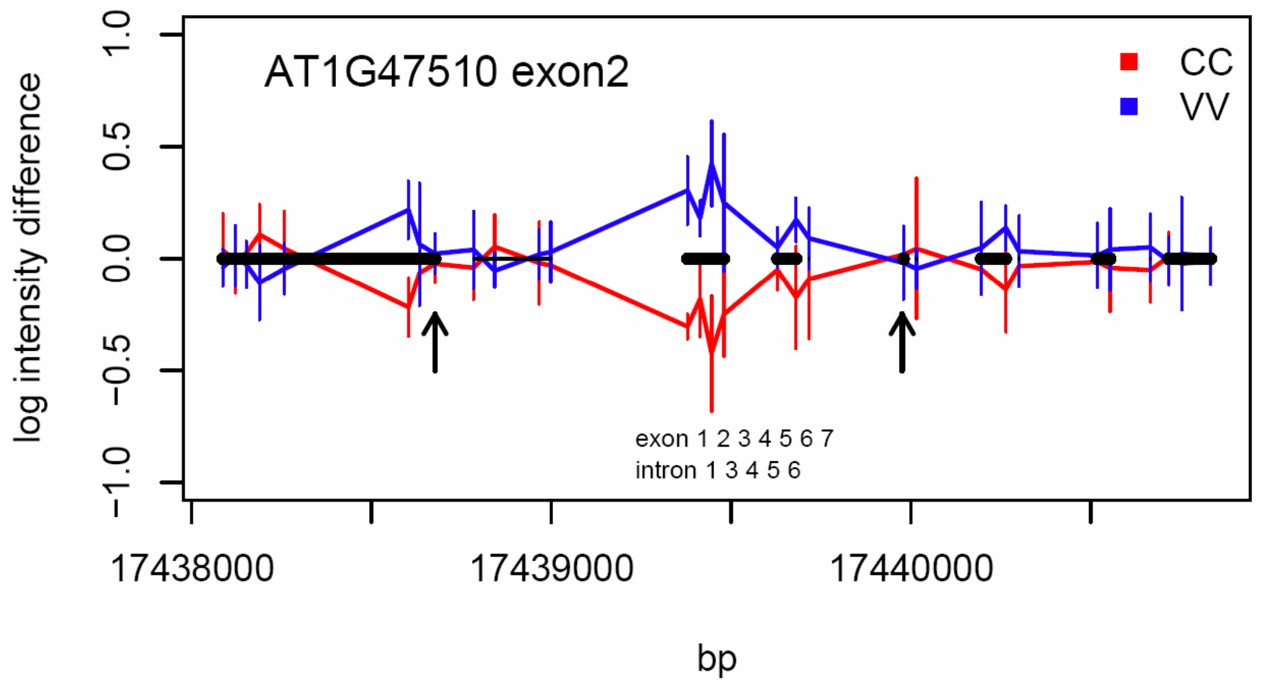
AT1G47510 exon2
CC
VV
log intensity difference
1.0
0.5
0.0
−0.5
−1.0
exon 1 2 3 4 5 6 7
intron 1 3 4 5 6
17438000
17439000
17440000
bp

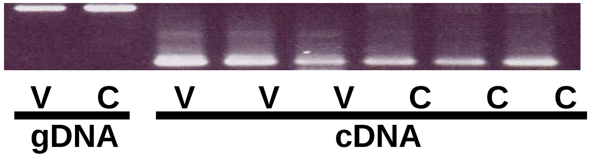
V C V V V C C C
gDNA
cDNA

√

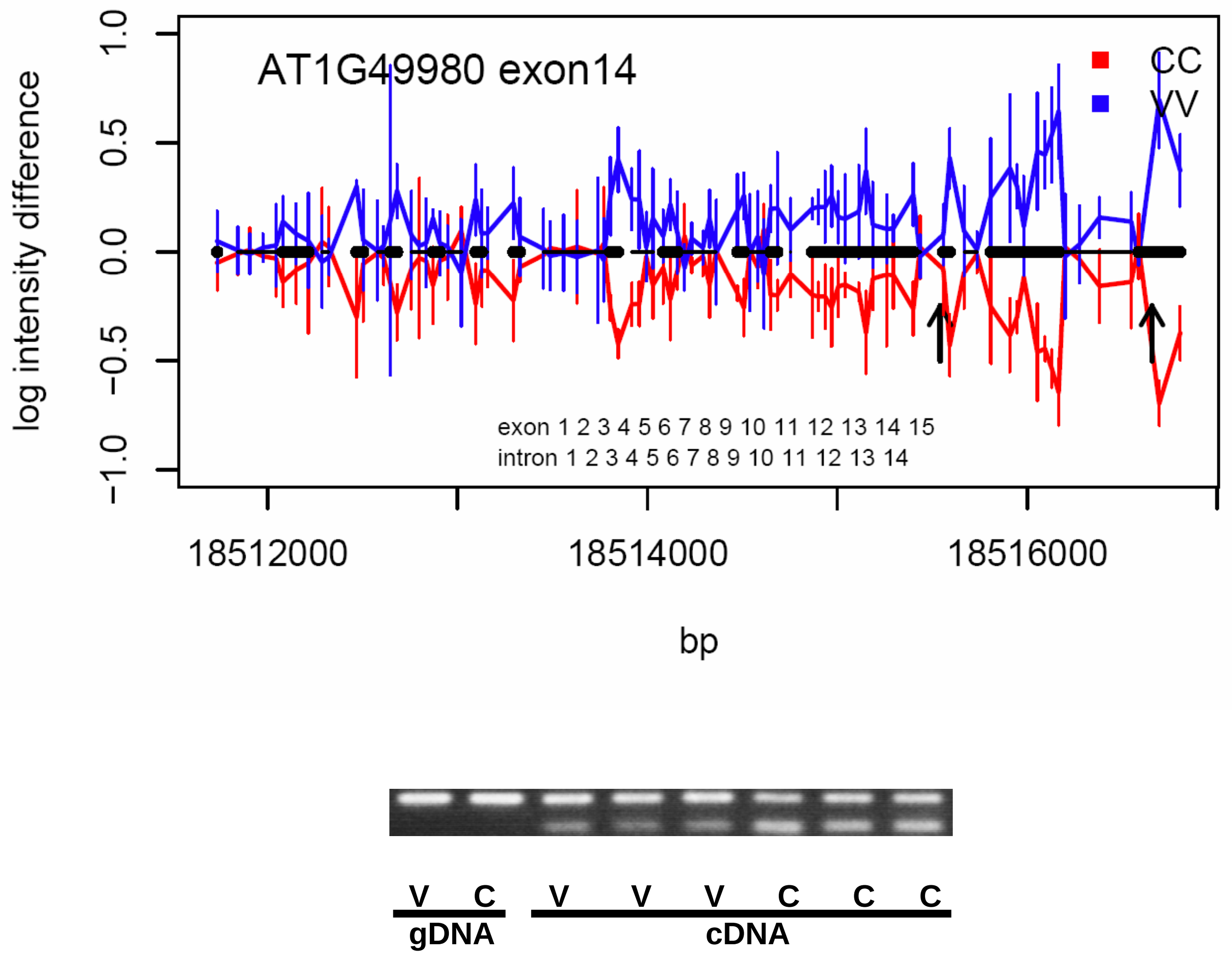
AT1G49980 exon14
CC
VV
log intensity difference
1.0
0.5
0.0
−0.5
−1.0
exon 1 2 3 4 5 6 7 8 9 10 11 12 13 14 15
intron 1 2 3 4 5 6 7 8 9 10 11 12 13 14
18512000
18514000
18516000
bp
V C V V V C C C
gDNA
cDNA

√

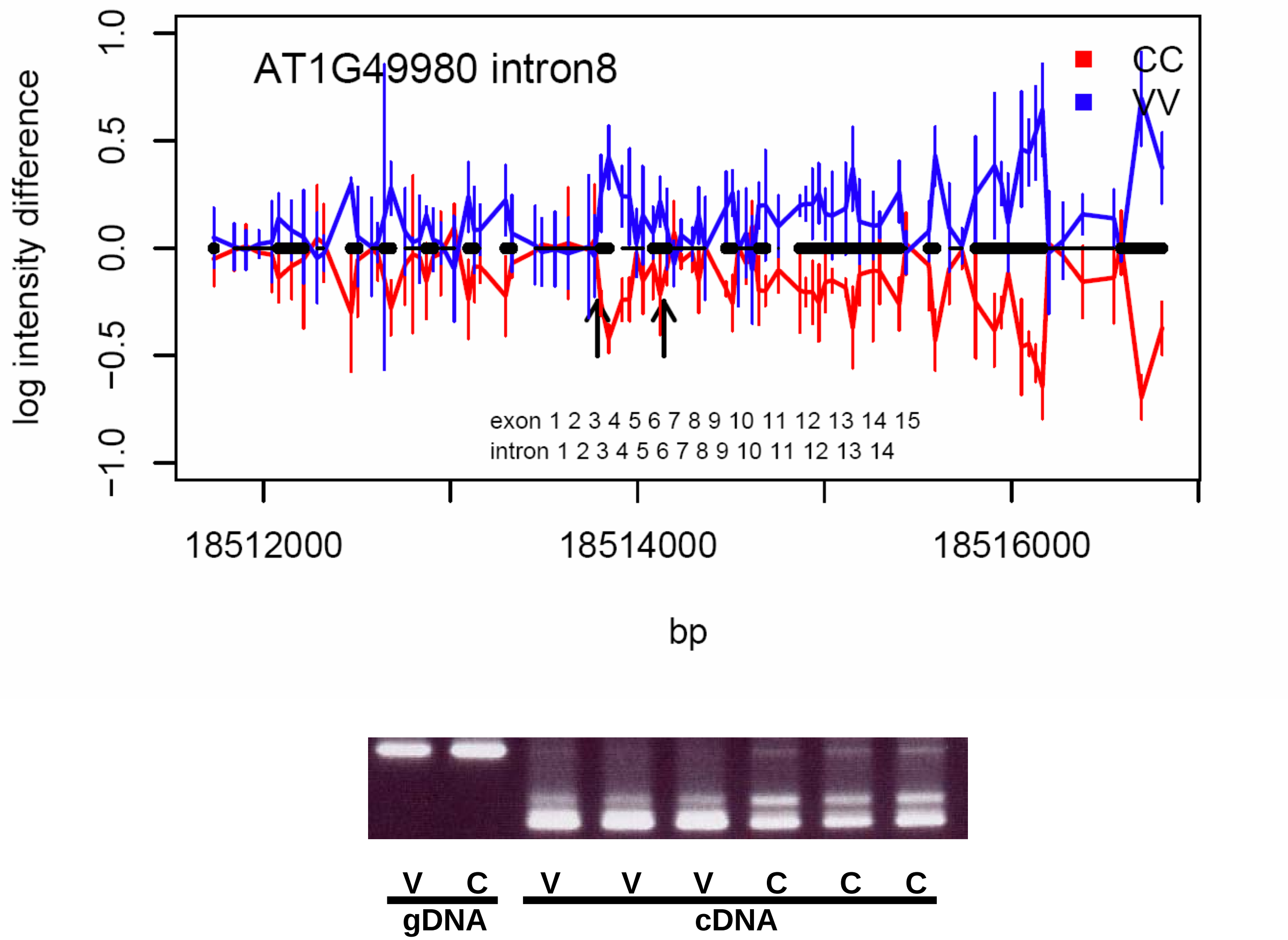
AT1G49980 intron8
CC
VV
log intensity difference
1.0
0.5
0.0
−0.5
−1.0
exon 1 2 3 4 5 6 7 8 9 10 11 12 13 14 15
intron 1 2 3 4 5 6 7 8 9 10 11 12 13 14
18512000
18514000
18516000
bp
V C V V V C C C
gDNA
cDNA

√

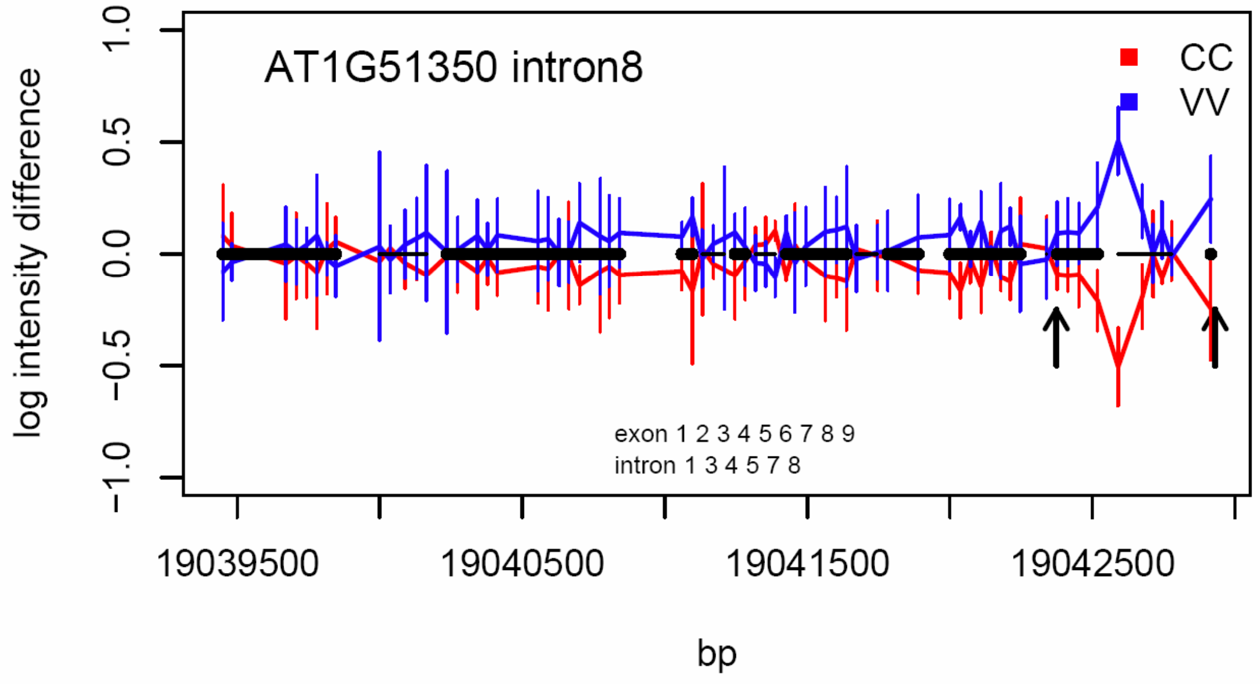
AT1G51350 intron8
CC
VV
log intensity difference
1.0
0.5
0.0
−0.5
−1.0
exon 1 2 3 4 5 6 7 8 9
intron 1 3 4 5 7 8
19039500
19040500
19041500
19042500
bp

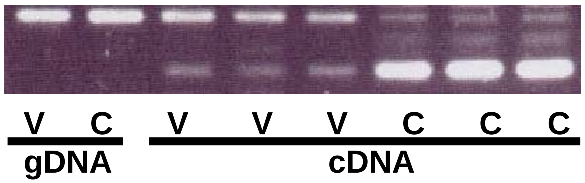
V C V V V C C C
gDNA
cDNA

√

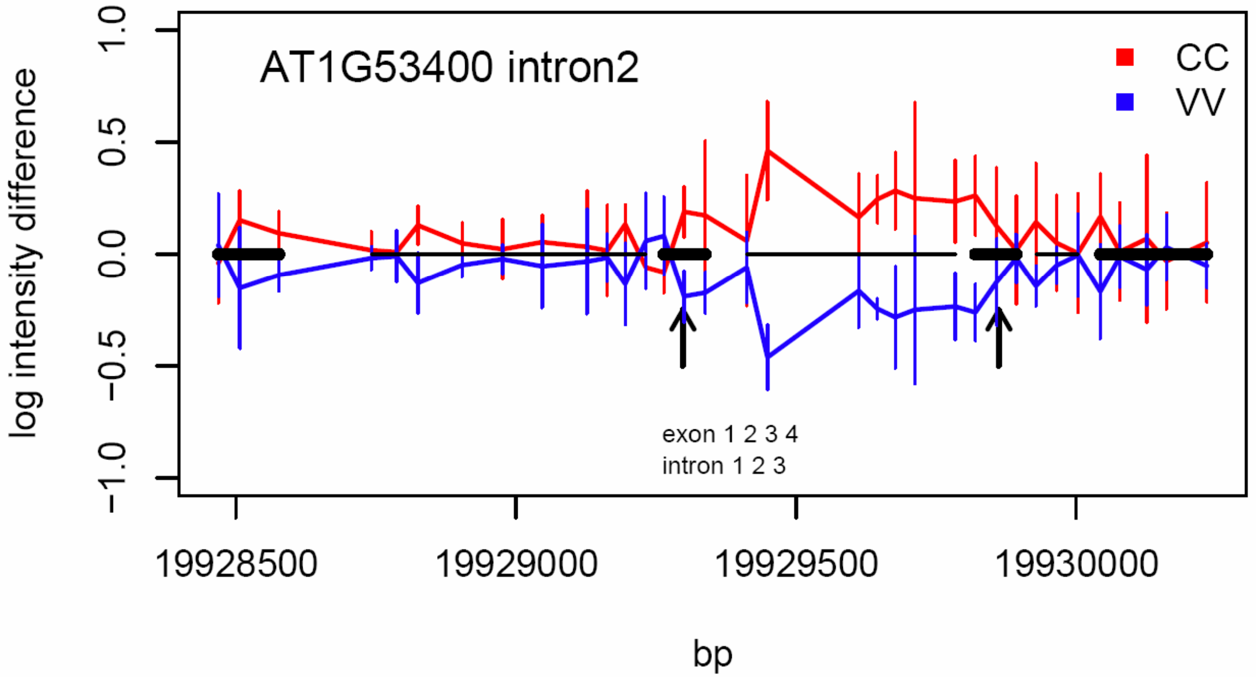
AT1G53400 intron2
CC
VV
log intensity difference
1.0
0.5
0.0
−0.5
−1.0
exon 1 2 3 4
intron 1 2 3
19928500
19929000
19929500
19930000
bp

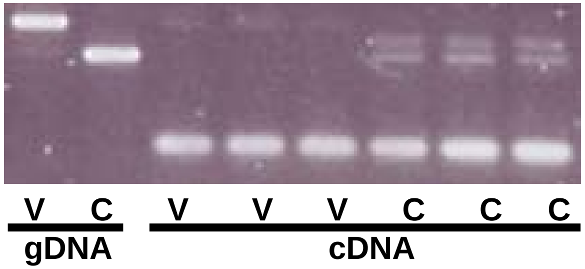
V C V V V C C C
gDNA
cDNA

√

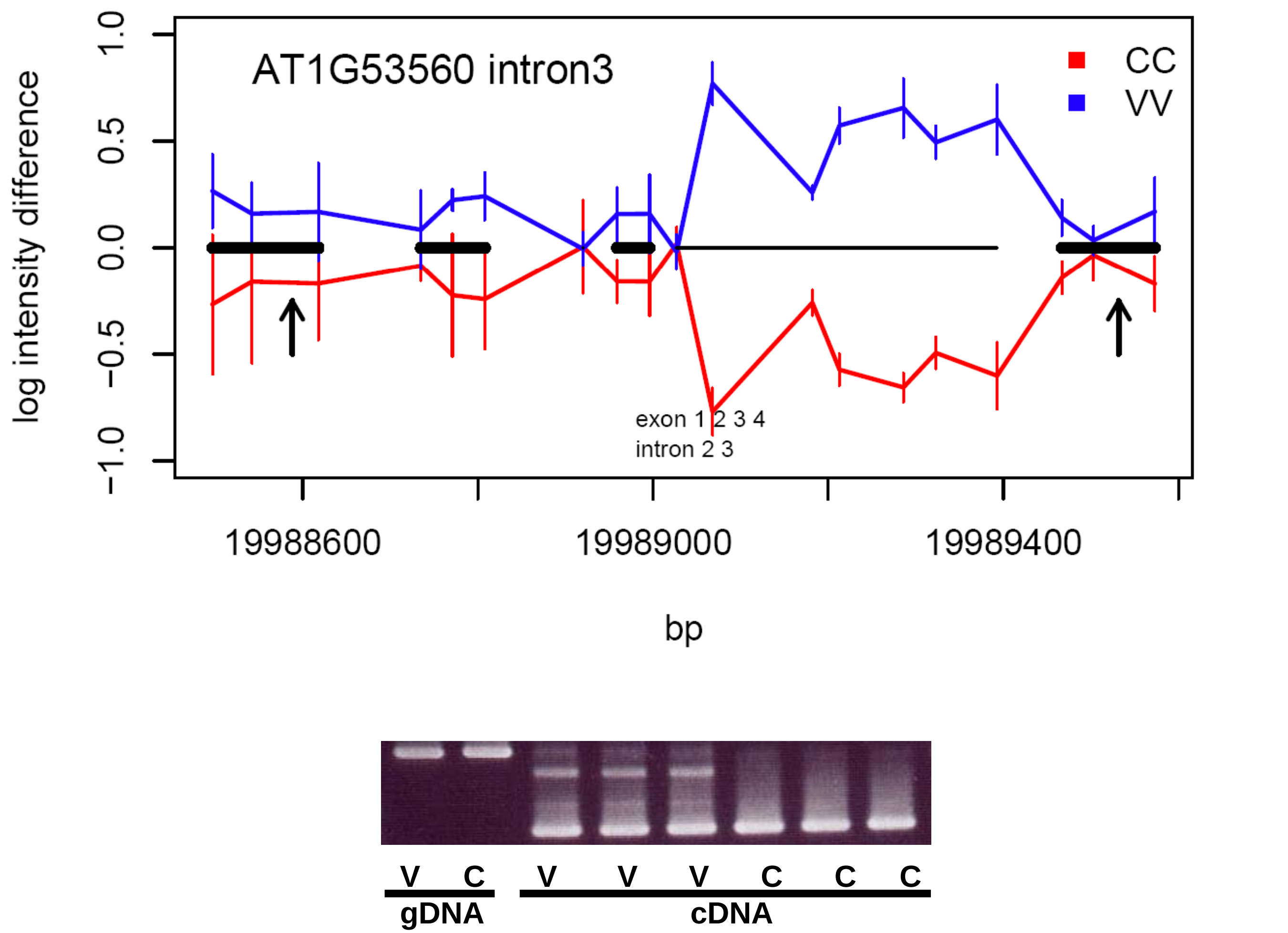
AT1G53560 intron3
CC
VV
log intensity difference
1.0
0.5
0.0
−0.5
−1.0
exon 1 2 3 4
intron 2 3
19988600
19989000
19989400
bp
V C V V V C C C
gDNA
cDNA

Not included for analysis

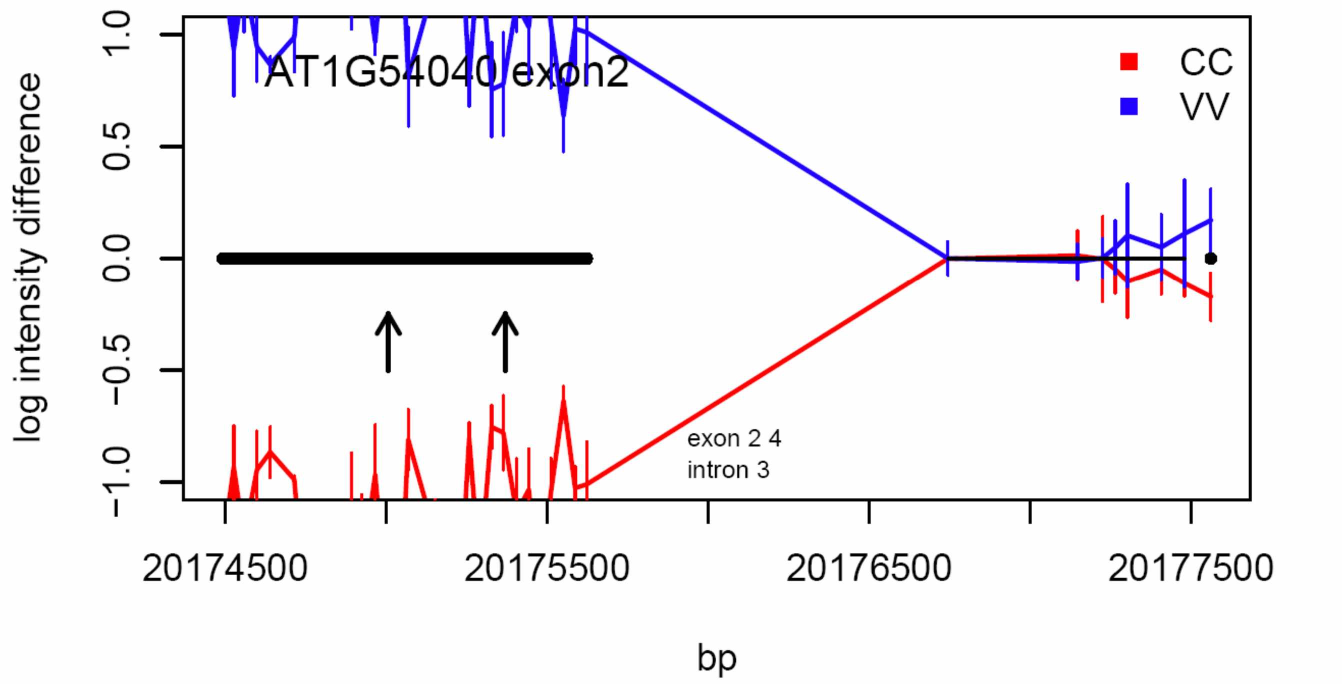

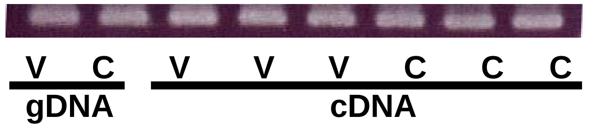

Not included for analysis

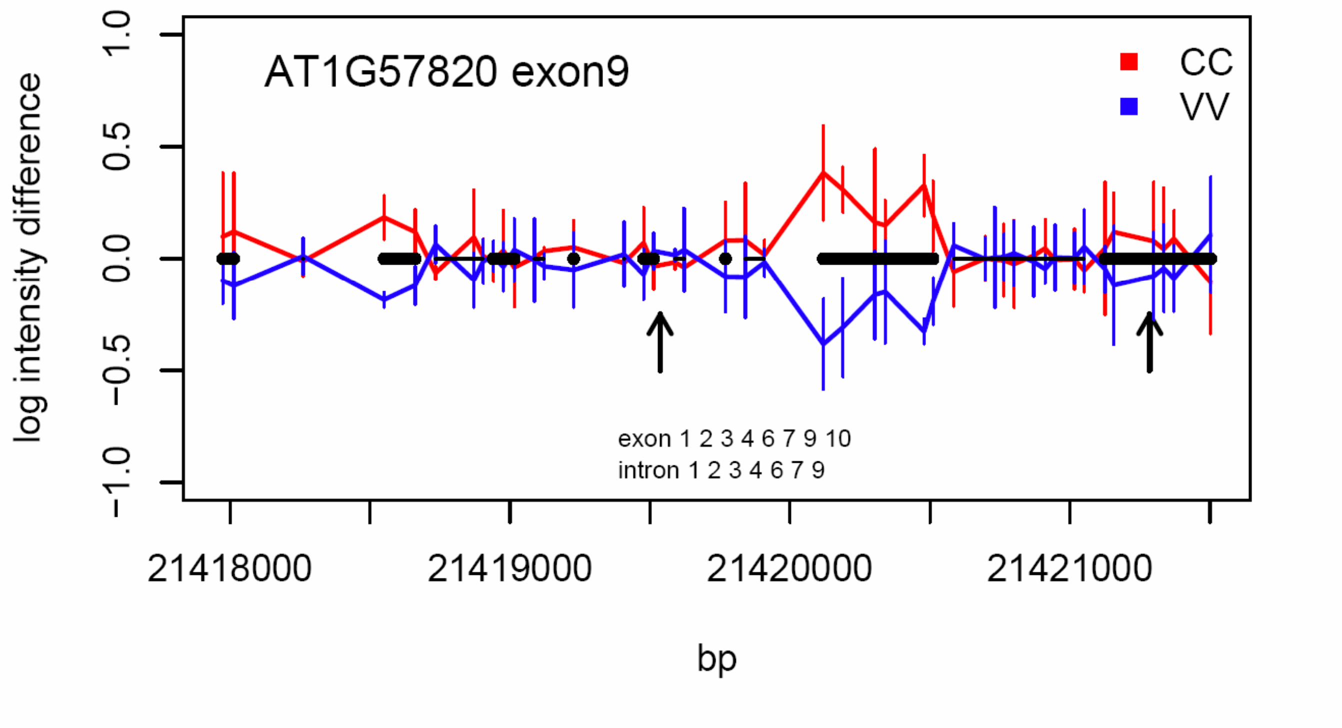

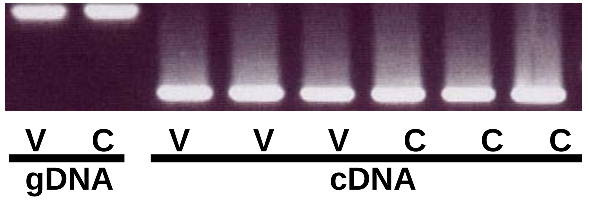

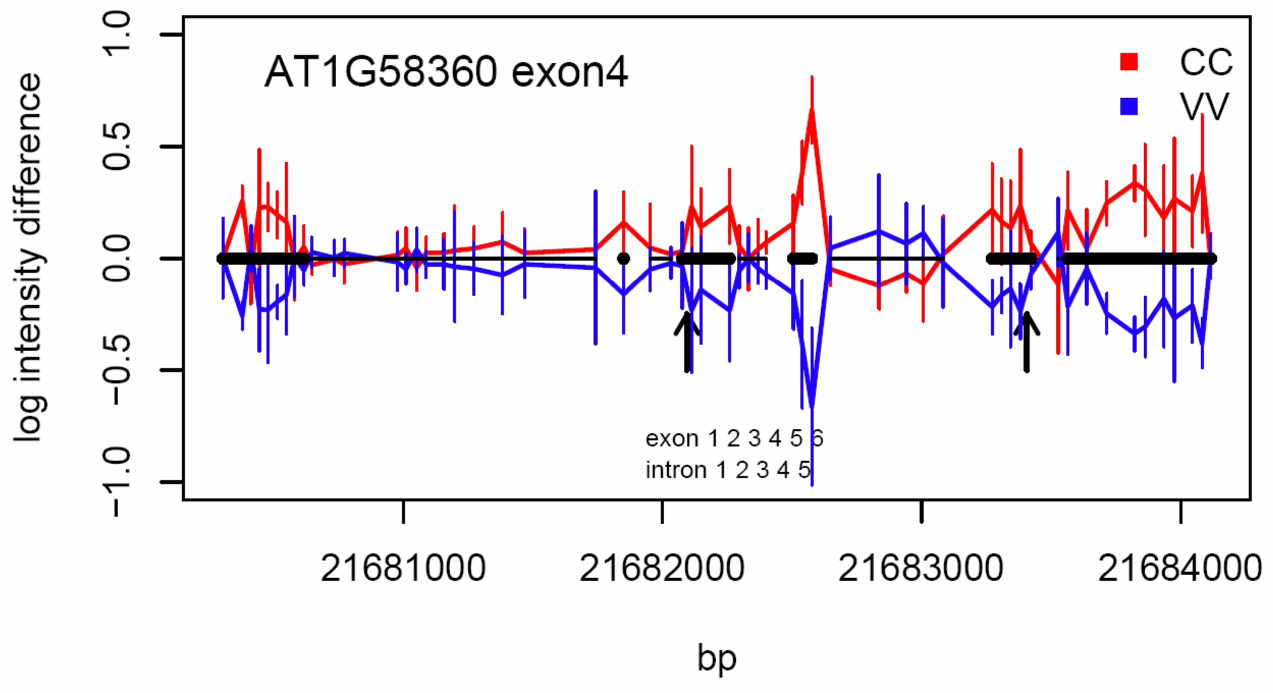
AT1G58360 exon4
CC
VV
log intensity difference
1.0
0.5
0.0
−0.5
−1.0
exon 1 2 3 4 5 6
intron 1 2 3 4 5
21681000
21682000
21683000
21684000
bp

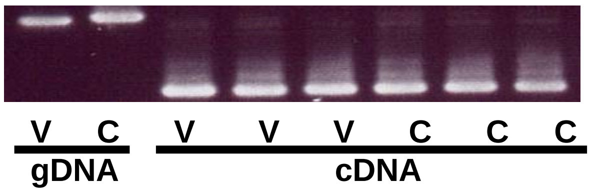
V C V V V C C C
gDNA
cDNA

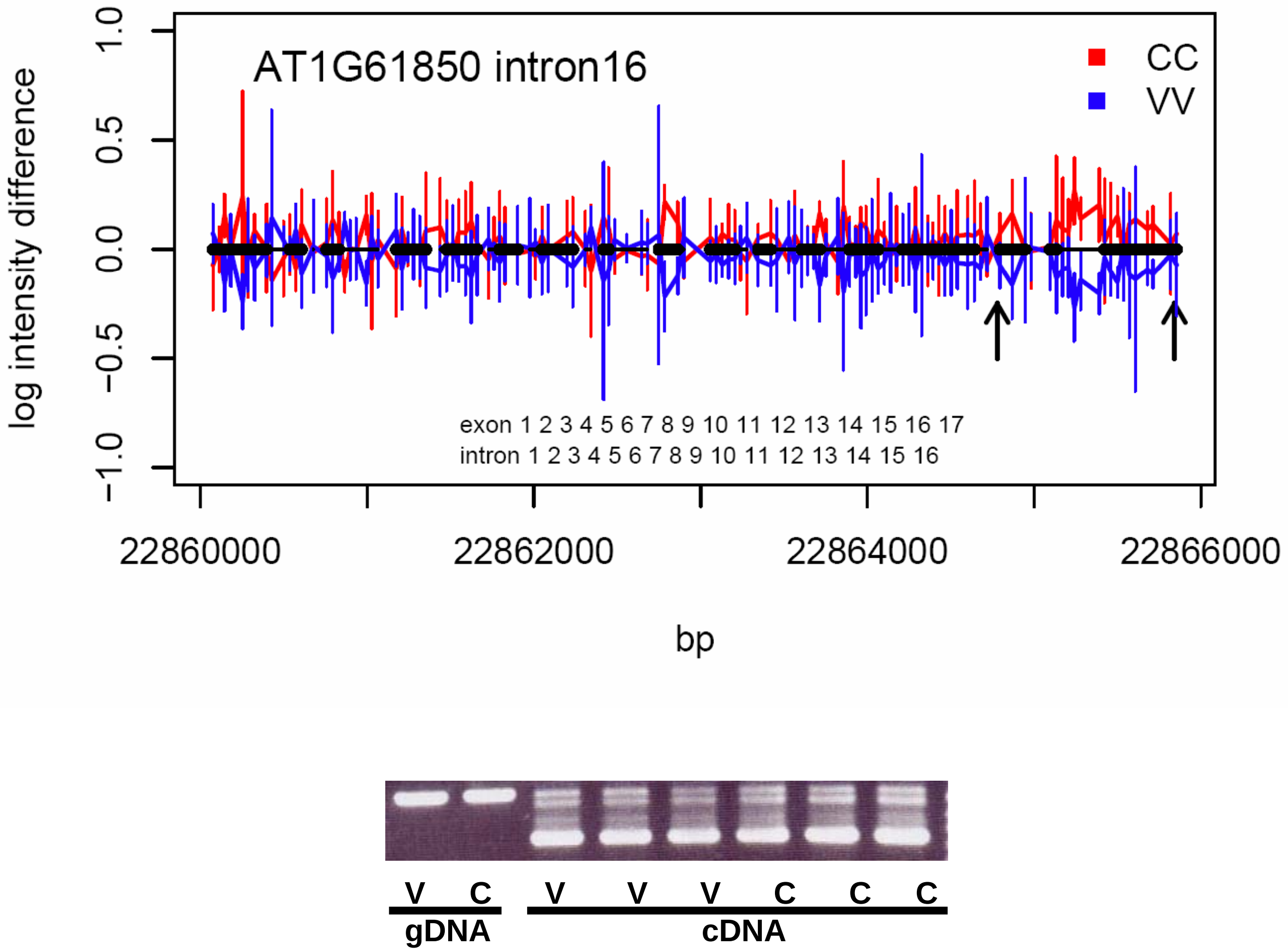
AT1G61850 intron16
CC
VV
log intensity difference
1.0
0.5
0.0
−0.5
−1.0
exon 1 2 3 4 5 6 7 8 9 10 11 12 13 14 15 16 17
intron 1 2 3 4 5 6 7 8 9 10 11 12 13 14 15 16
22860000
22862000
22864000
22866000
bp
V C V V V C C C
gDNA
cDNA

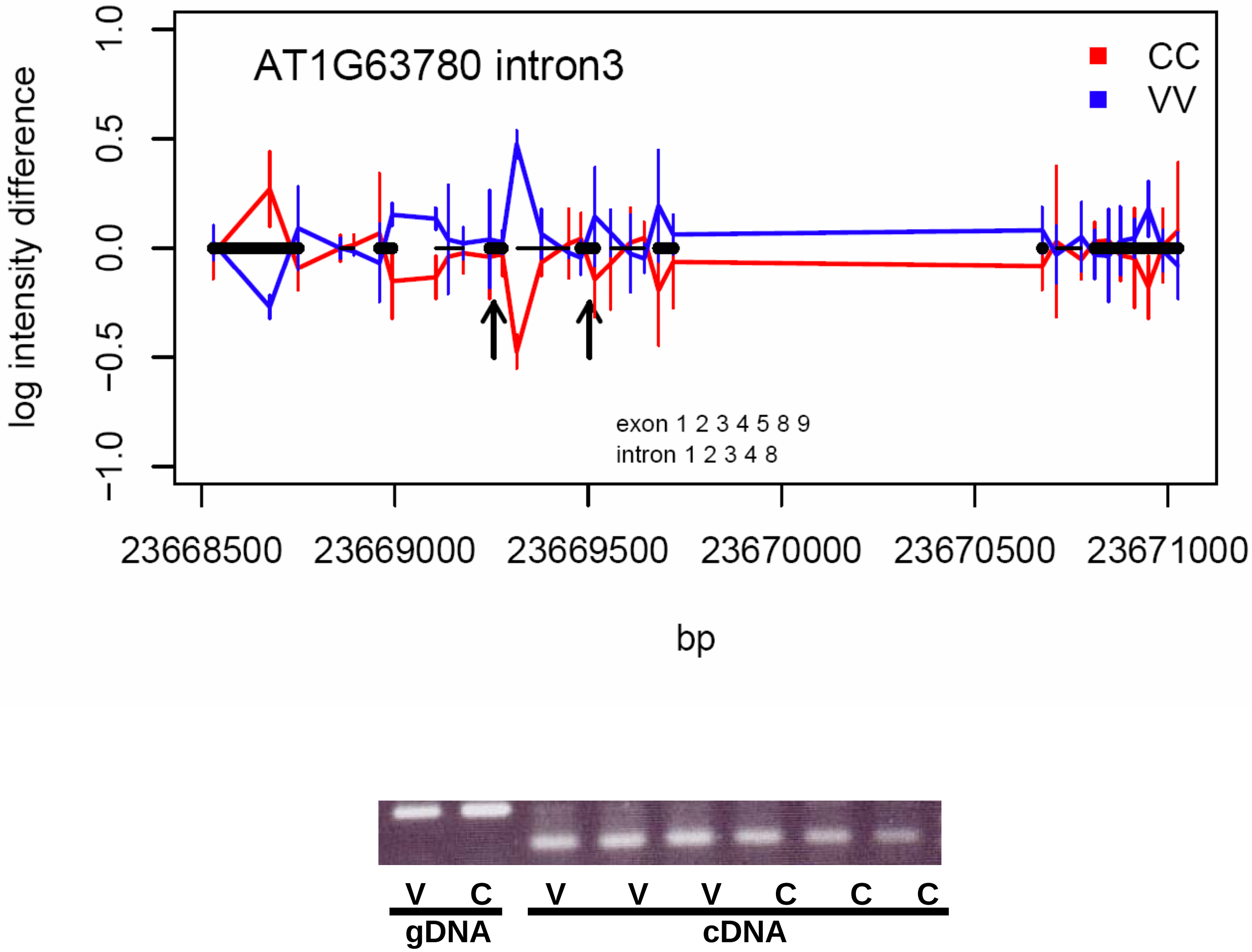
AT1G63780 intron3
CC
VV
log intensity difference
1.0
0.5
0.0
−0.5
−1.0
exon 1 2 3 4 5 8 9
intron 1 2 3 4 8
23668500
23669000
23669500
23670000
23670500
23671000
bp
V C V V V C C C
gDNA
cDNA

√

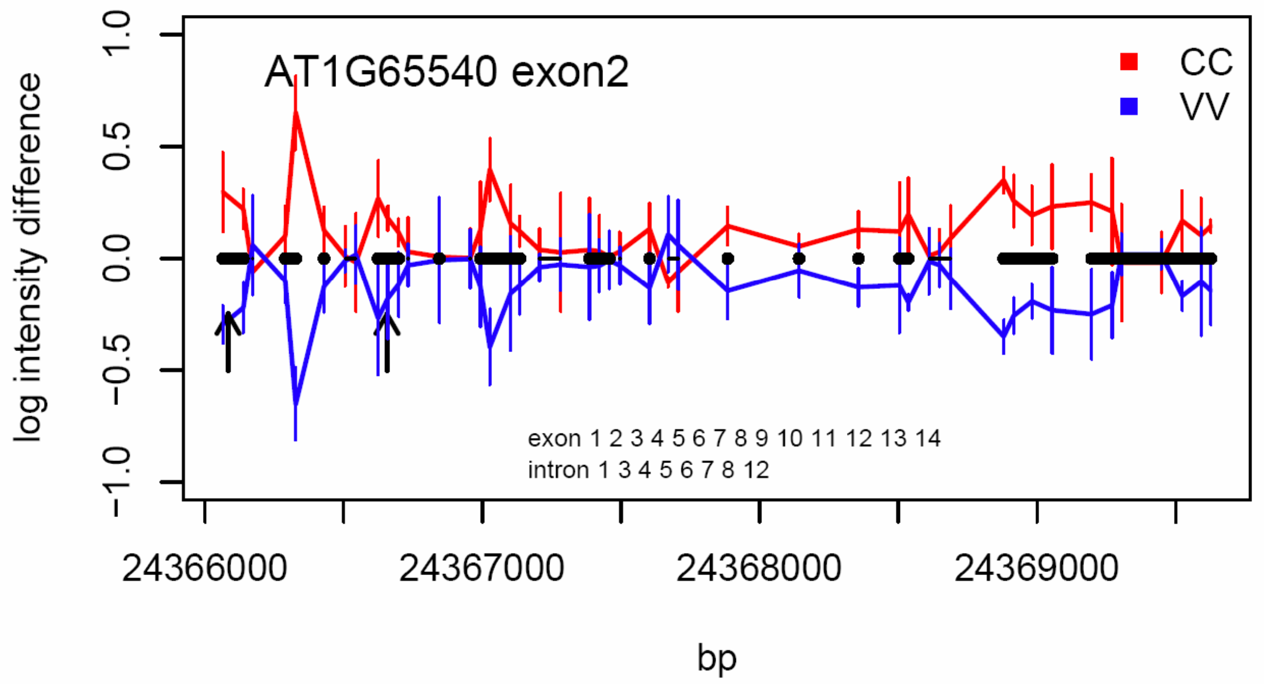
AT1G65540 exon2
CC
VV
log intensity difference
1.0
0.5
0.0
−0.5
−1.0
exon 1 2 3 4 5 6 7 8 9 10 11 12 13 14
intron 1 3 4 5 6 7 8 12
24366000
24367000
24368000
24369000
bp

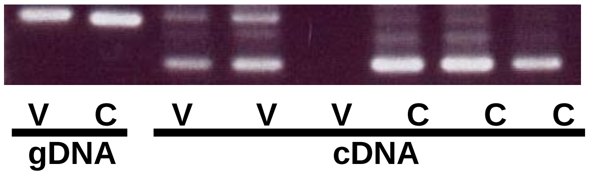
V C V V V C C C
gDNA
cDNA

Not included for analysis

√

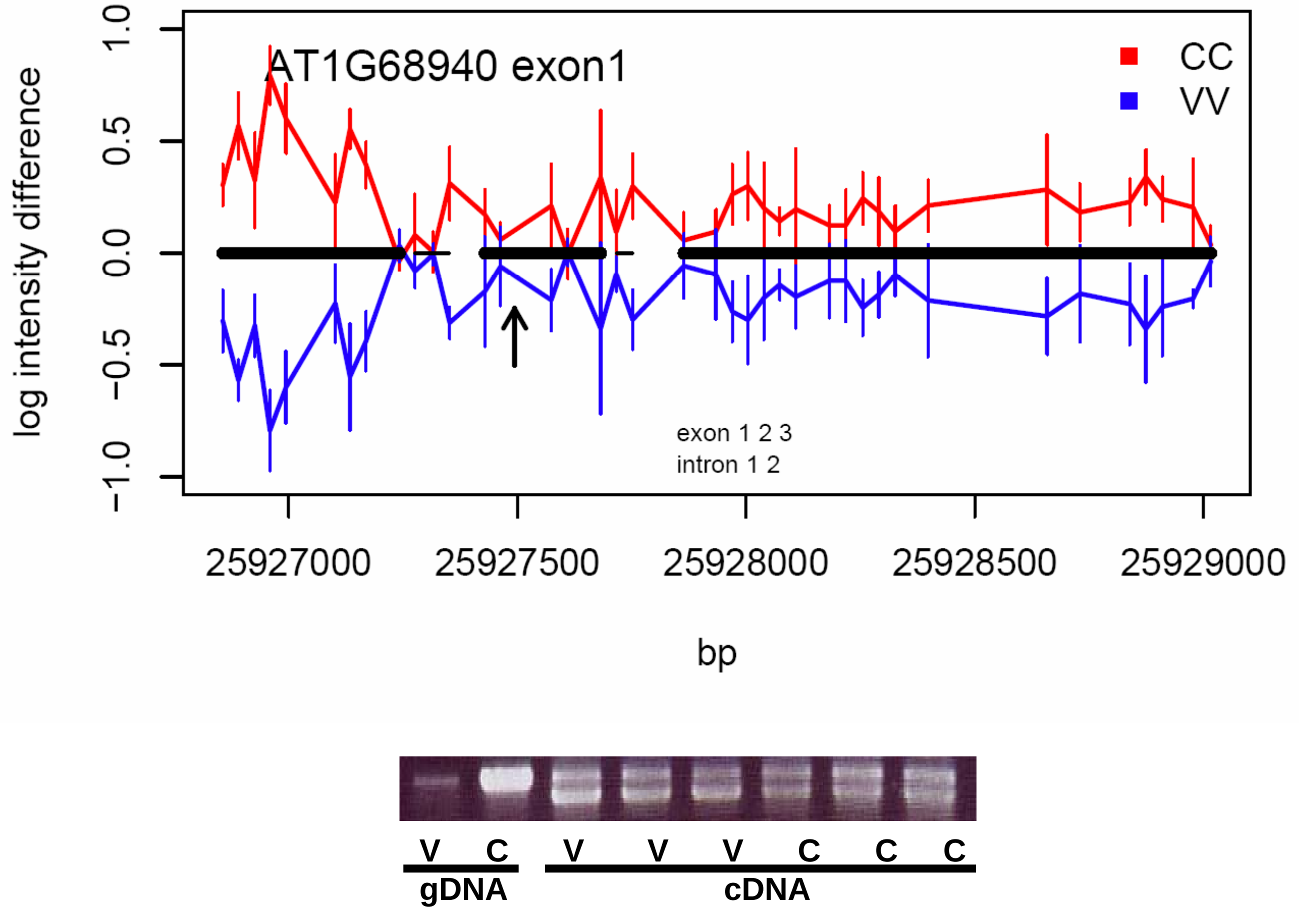

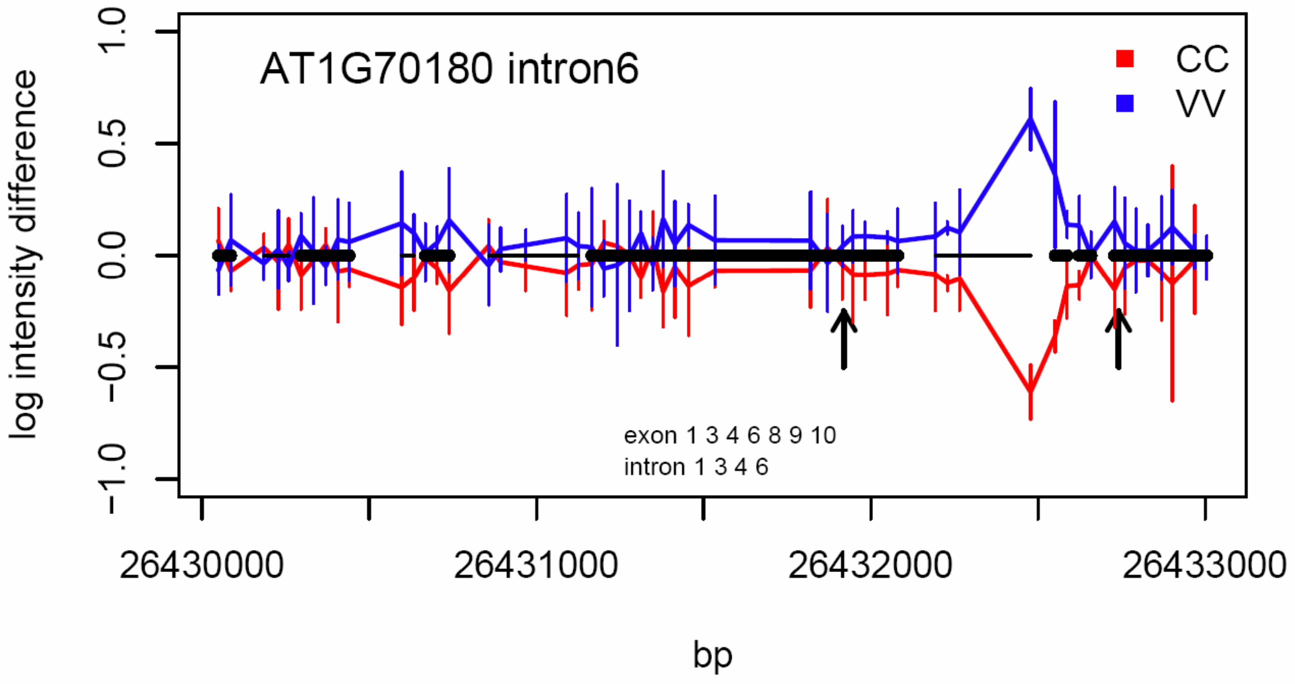
AT1G70180 intron6
CC
VV
log intensity difference
1.0
0.5
0.0
−0.5
−1.0
exon 1 3 4 6 8 9 10
intron 1 3 4 6
26430000
26431000
26432000
26433000
bp

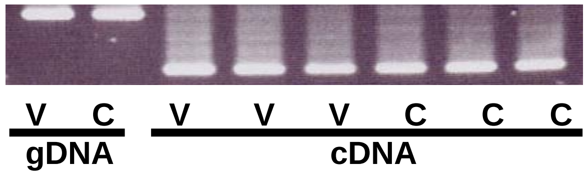
V C V V V C C C
gDNA
cDNA

√

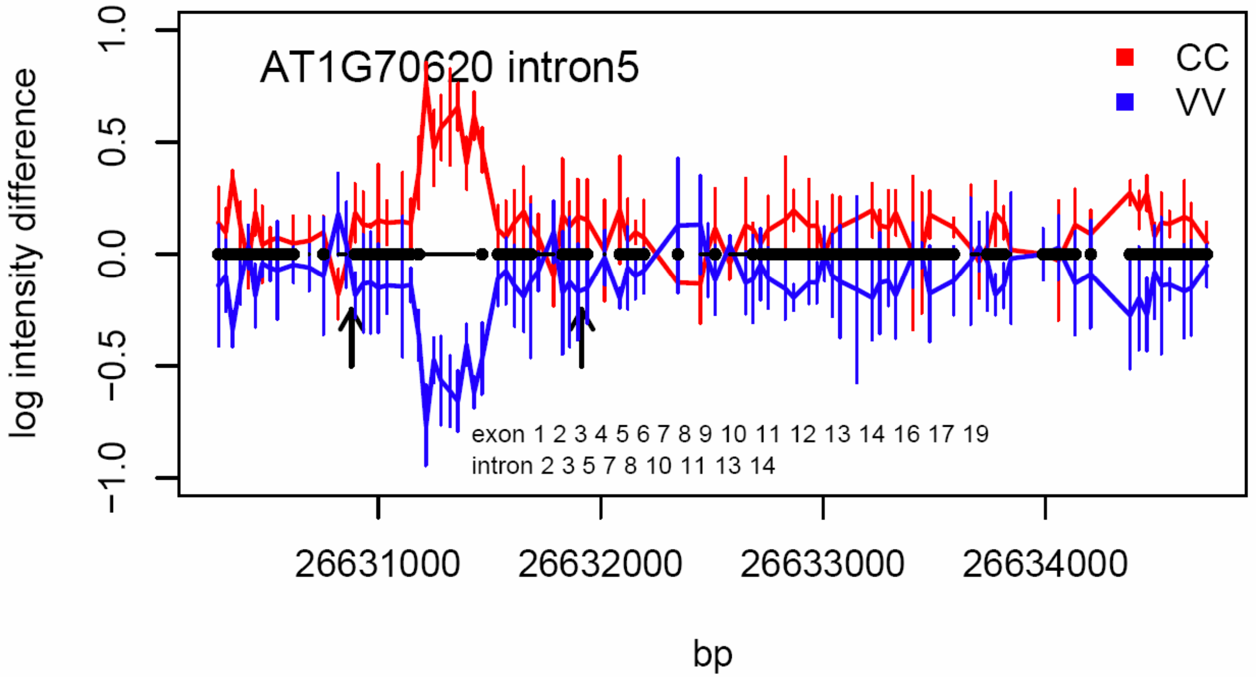
AT1G70620 intron5
CC
VV
log intensity difference
1.0
0.5
0.0
−0.5
−1.0
exon 1 2 3 4 5 6 7 8 9 10 11 12 13 14 16 17 19
intron 2 3 5 7 8 10 11 13 14
26631000
26632000
26633000
26634000
bp

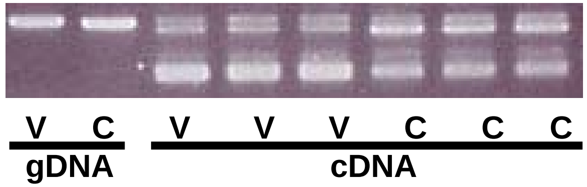
V C V V V C C C
gDNA
cDNA

√

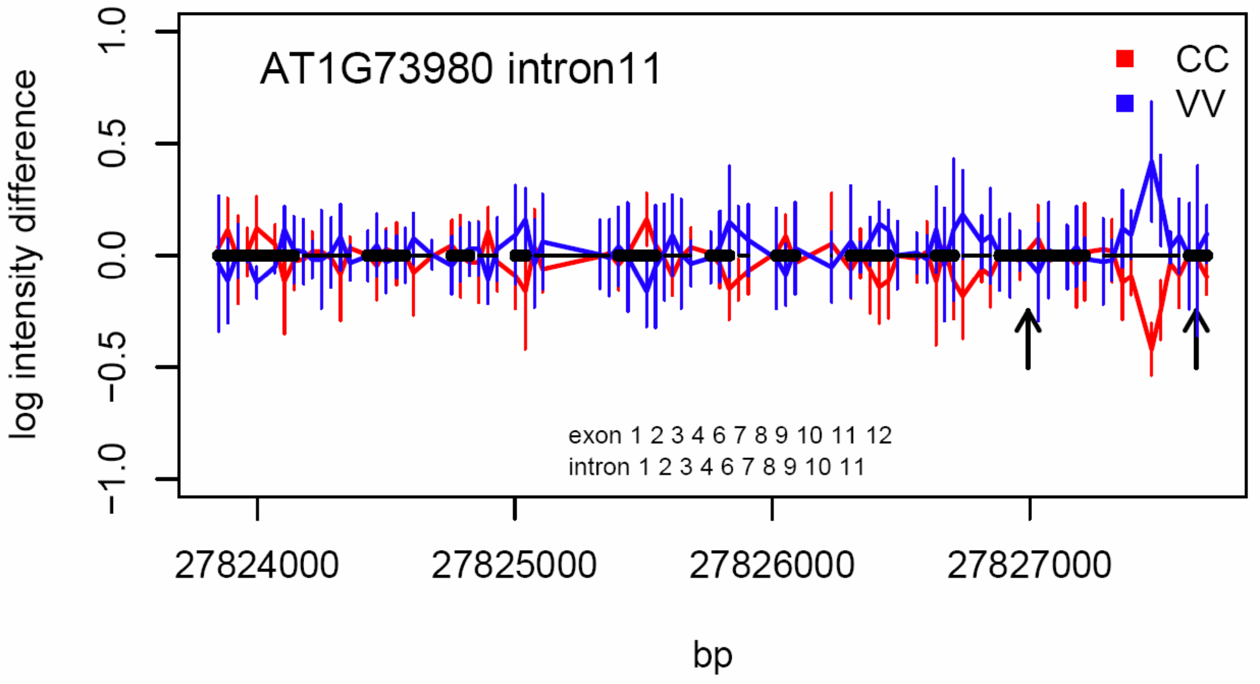
AT1G73980 intron11
CC
VV
log intensity difference
1.0
0.5
0.0
−0.5
−1.0
exon 1 2 3 4 6 7 8 9 10 11 12
intron 1 2 3 4 6 7 8 9 10 11
27824000
27825000
27826000
27827000
bp

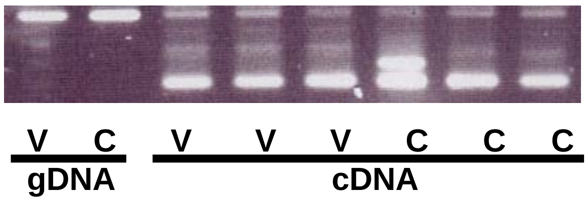
V C V V V C C C
gDNA
cDNA

√

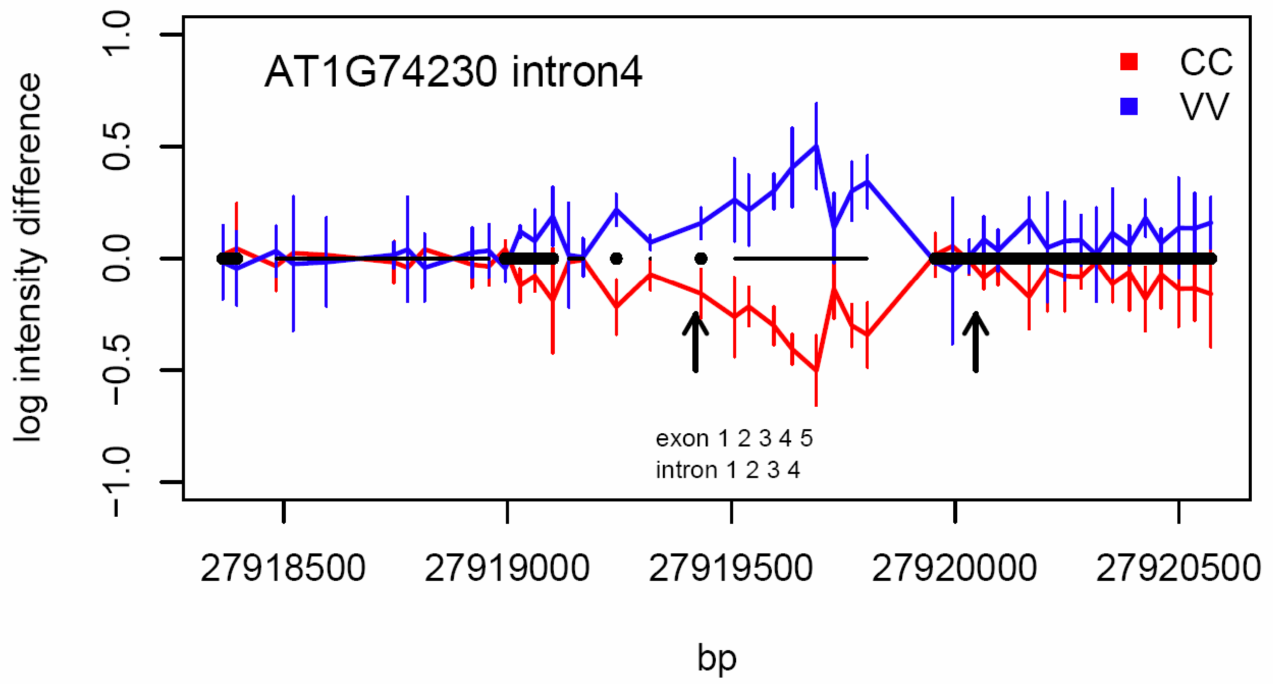
AT1G74230 intron4
CC
VV
log intensity difference
1.0
0.5
0.0
−0.5
−1.0
exon 1 2 3 4 5
intron 1 2 3 4
27918500
27919000
27919500
27920000
27920500
bp

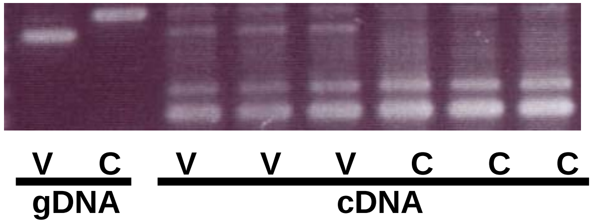
V C
gDNA
V V V C C C
cDNA

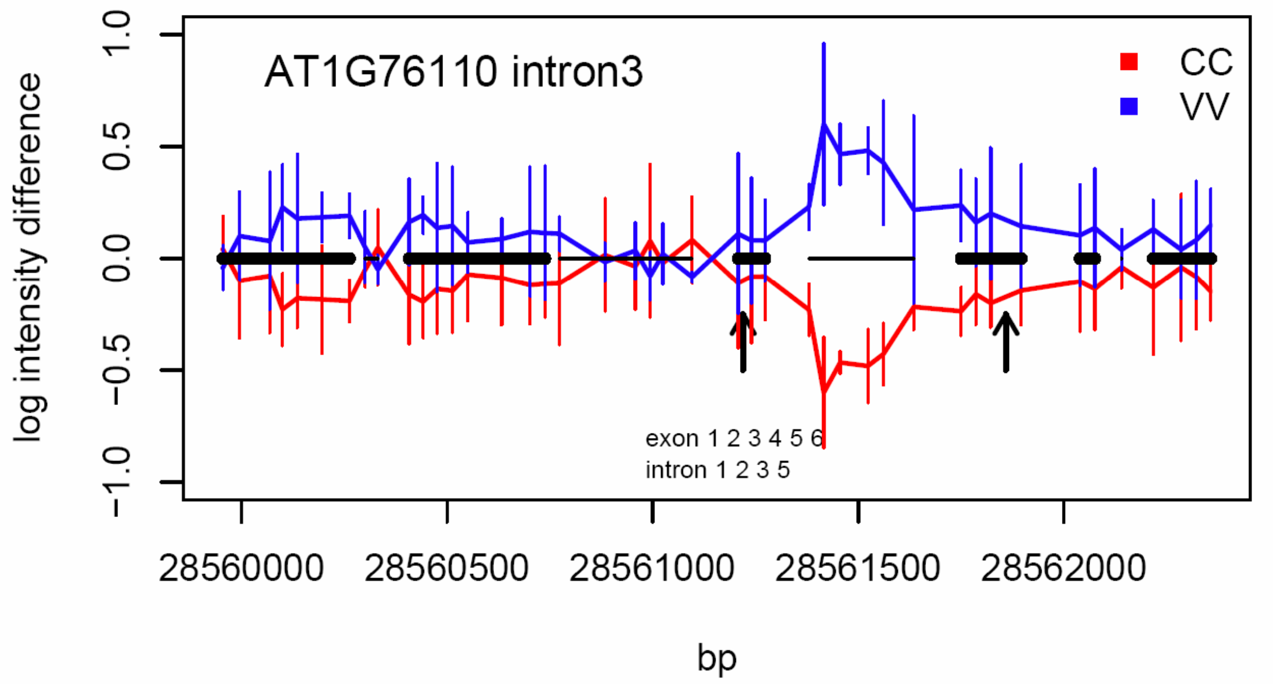
AT1G76110 intron3
CC
VV
log intensity difference
1.0
0.5
0.0
−0.5
−1.0
exon 1 2 3 4 5 6
intron 1 2 3 5
28560000
28560500
28561000
28561500
28562000
bp

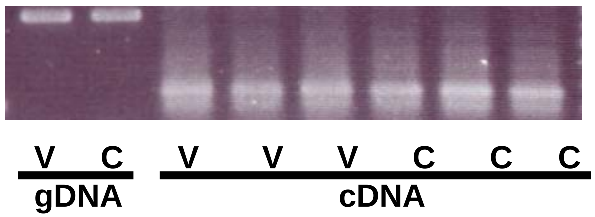
V C V V V C C C
gDNA
cDNA

√

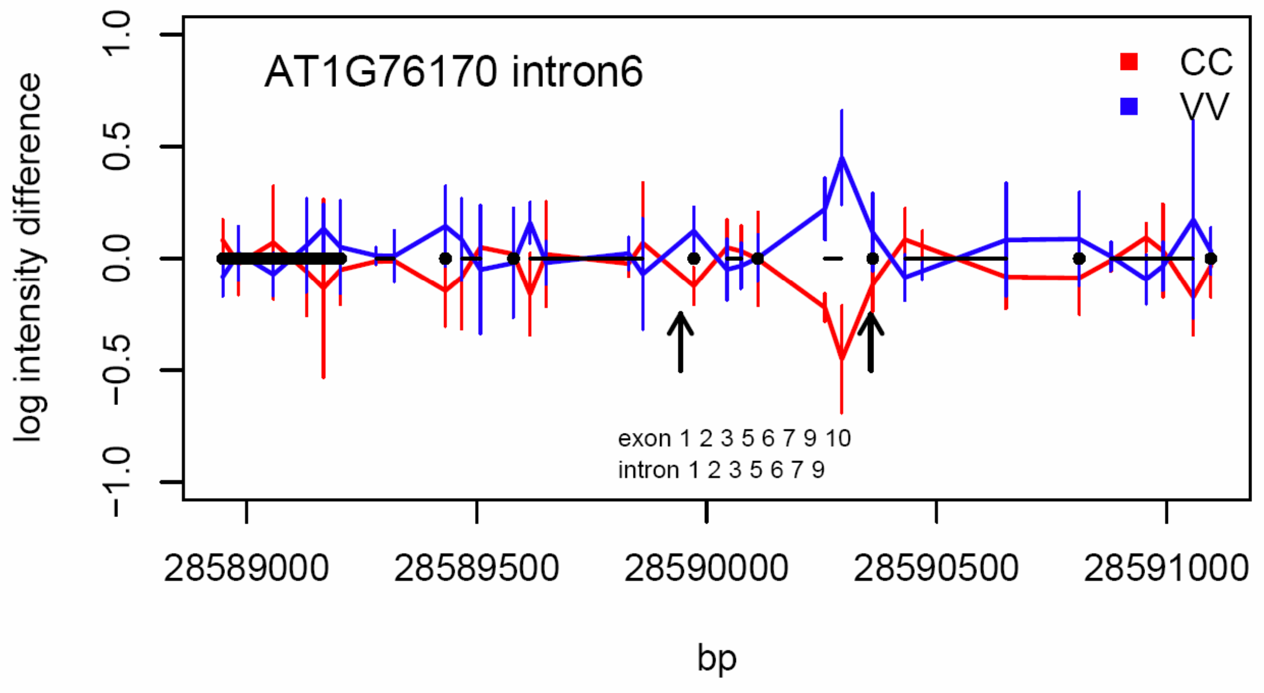
AT1G76170 intron6
CC
VV
log intensity difference
1.0
0.5
0.0
−0.5
−1.0
exon 1 2 3 5 6 7 9 10
intron 1 2 3 5 6 7 9
28589000
28589500
28590000
28590500
28591000
bp

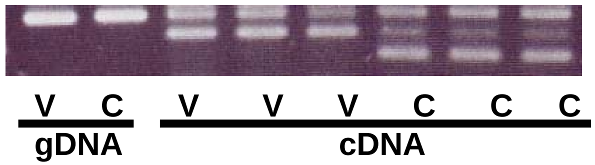
V C V V V C C C
gDNA
cDNA

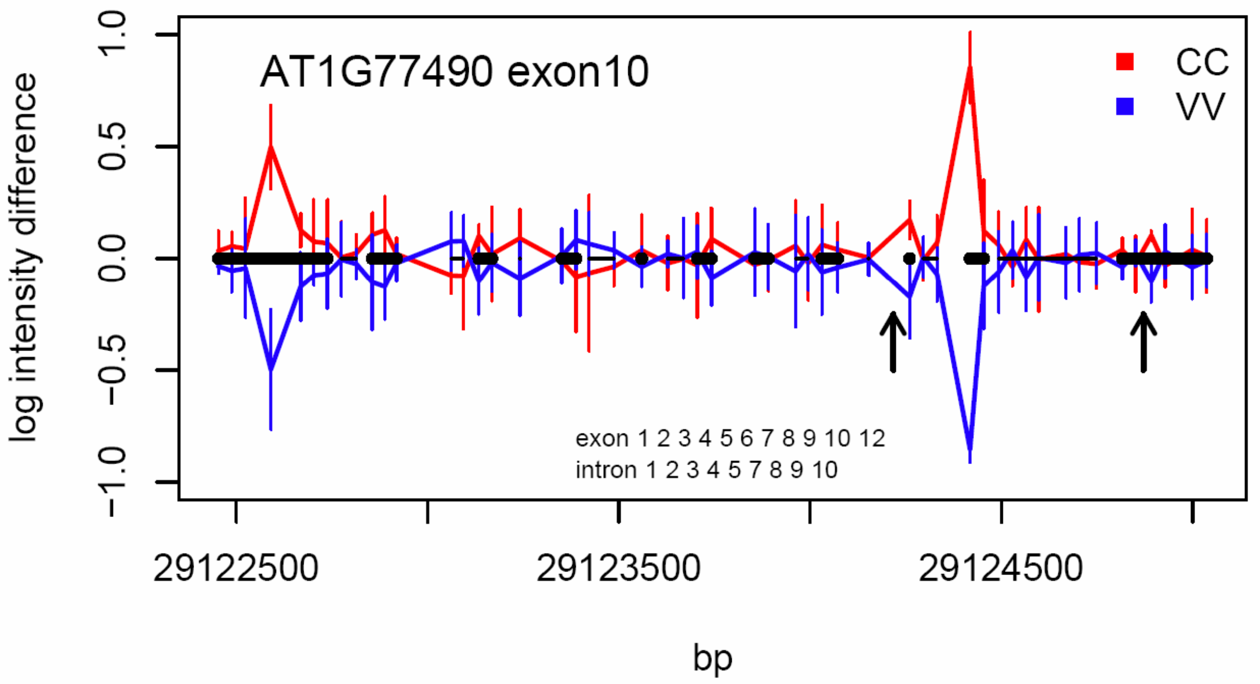
AT1G77490 exon10
CC
VV
log intensity difference
1.0
0.5
0.0
−0.5
−1.0
exon 1 2 3 4 5 6 7 8 9 10 12
intron 1 2 3 4 5 7 8 9 10
29122500
29123500
29124500
bp

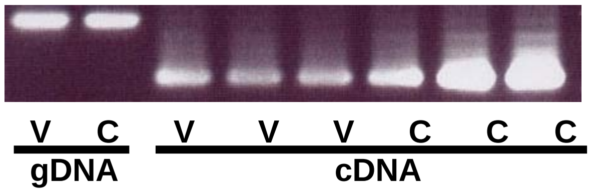
V C V V V C C C
gDNA
cDNA

√

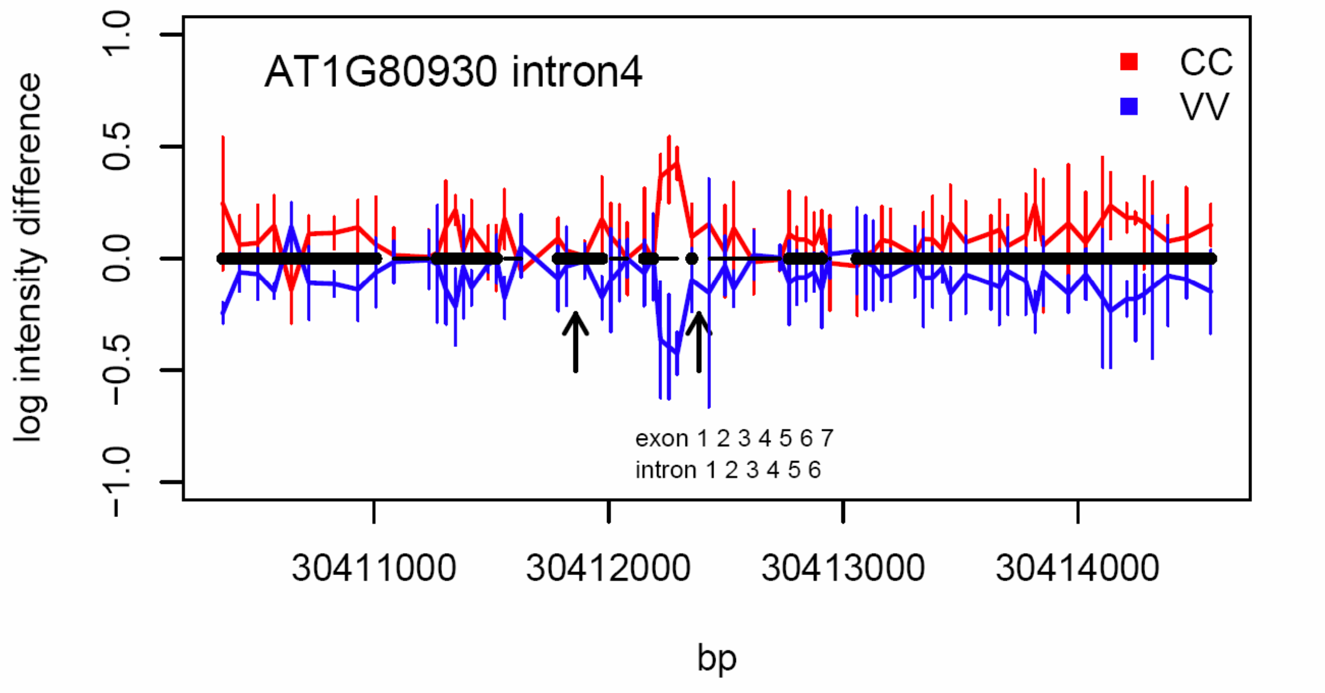
AT1G80930 intron4
CC
VV
log intensity difference
1.0
0.5
0.0
−0.5
−1.0
exon 1 2 3 4 5 6 7
intron 1 2 3 4 5 6
30411000
30412000
30413000
30414000
bp

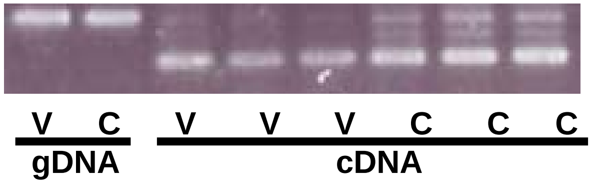
V C V V V C C C
gDNA
cDNA

√

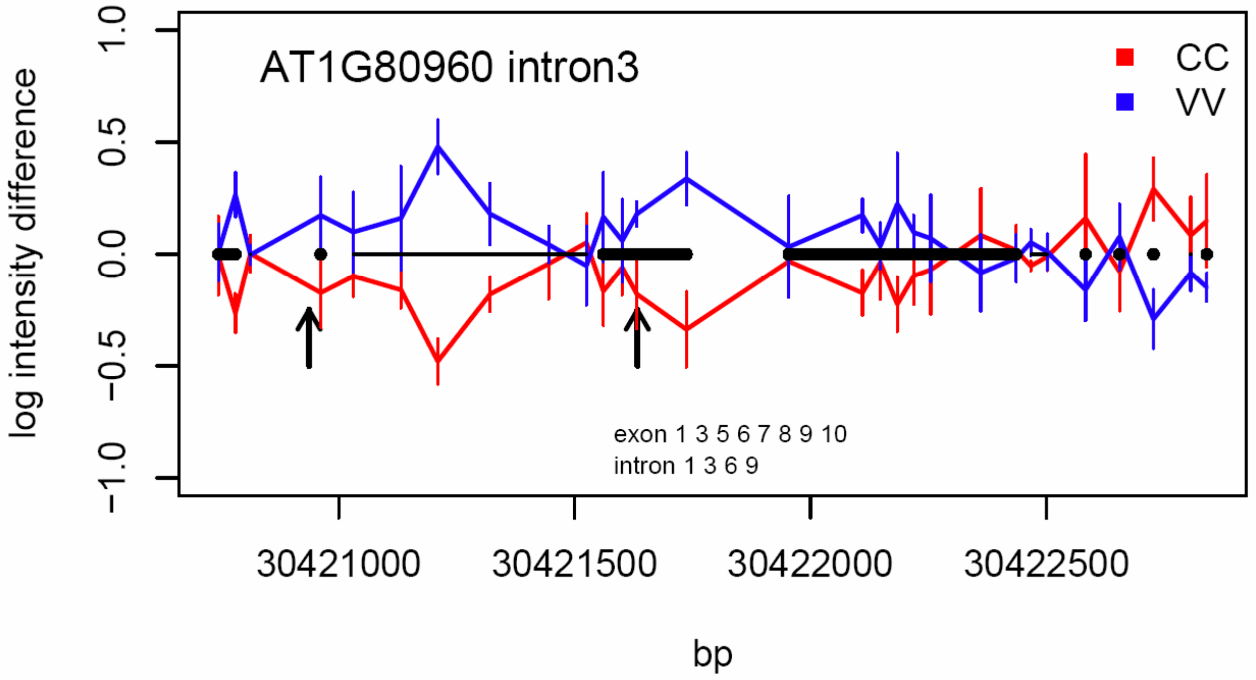
AT1G80960 intron3
CC
VV
log intensity difference
1.0
0.5
0.0
−0.5
−1.0
exon 1 3 5 6 7 8 9 10
intron 1 3 6 9
30421000
30421500
30422000
30422500
bp

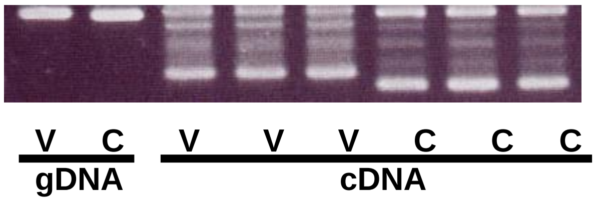
V C V V V C C C
gDNA
cDNA

√

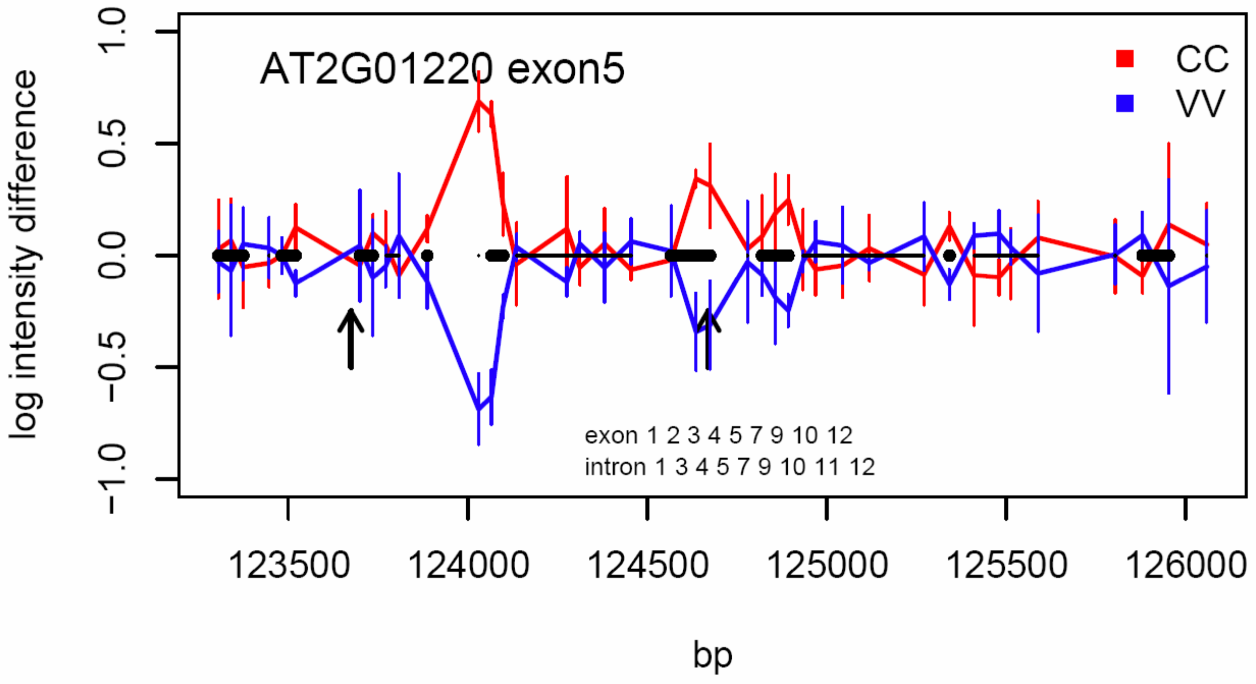
AT2G01220 exon5
CC
VV
log intensity difference
1.0
0.5
0.0
−0.5
−1.0
exon 1 2 3 4 5 7 9 10 12
intron 1 3 4 5 7 9 10 11 12
123500
124000
124500
125000
125500
126000
bp

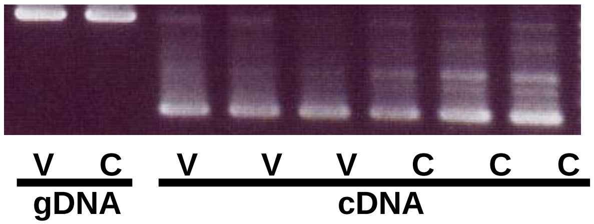
V C V V V C C C
gDNA
cDNA

Not included for analysis

√

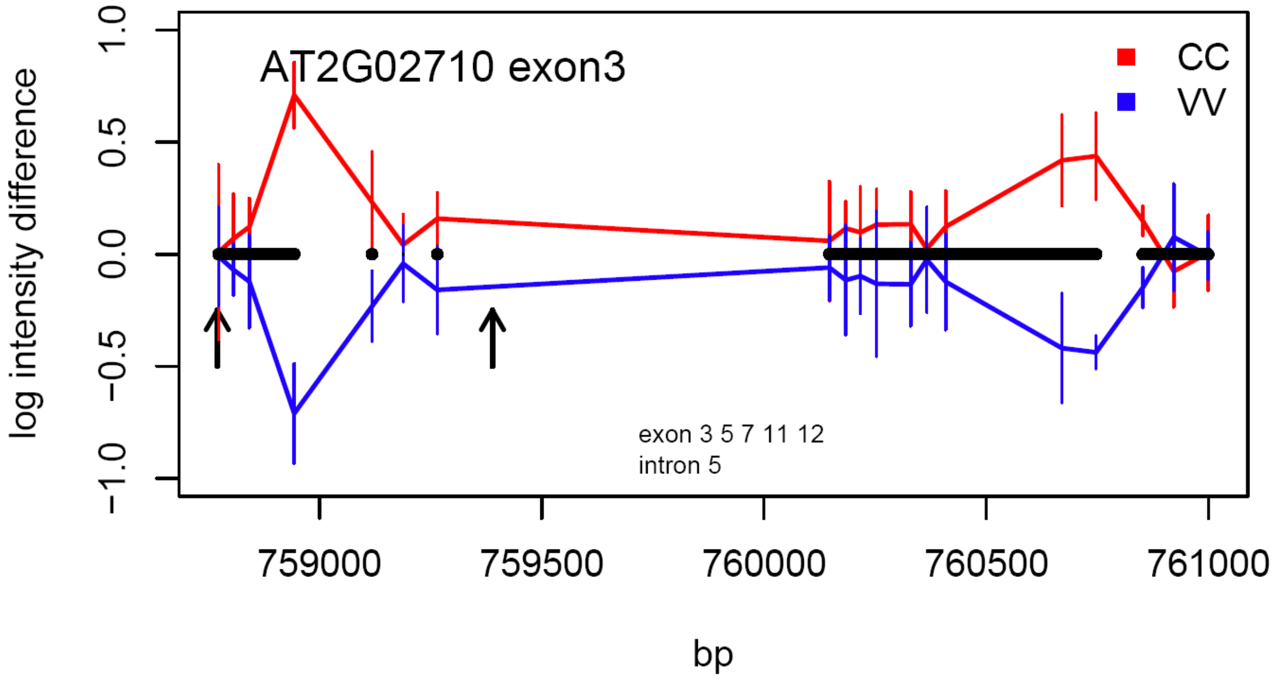

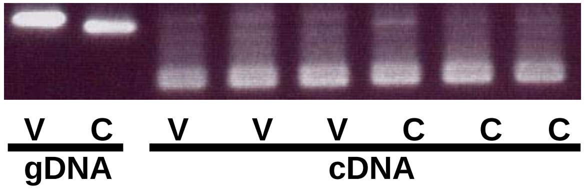

Not included for analysis

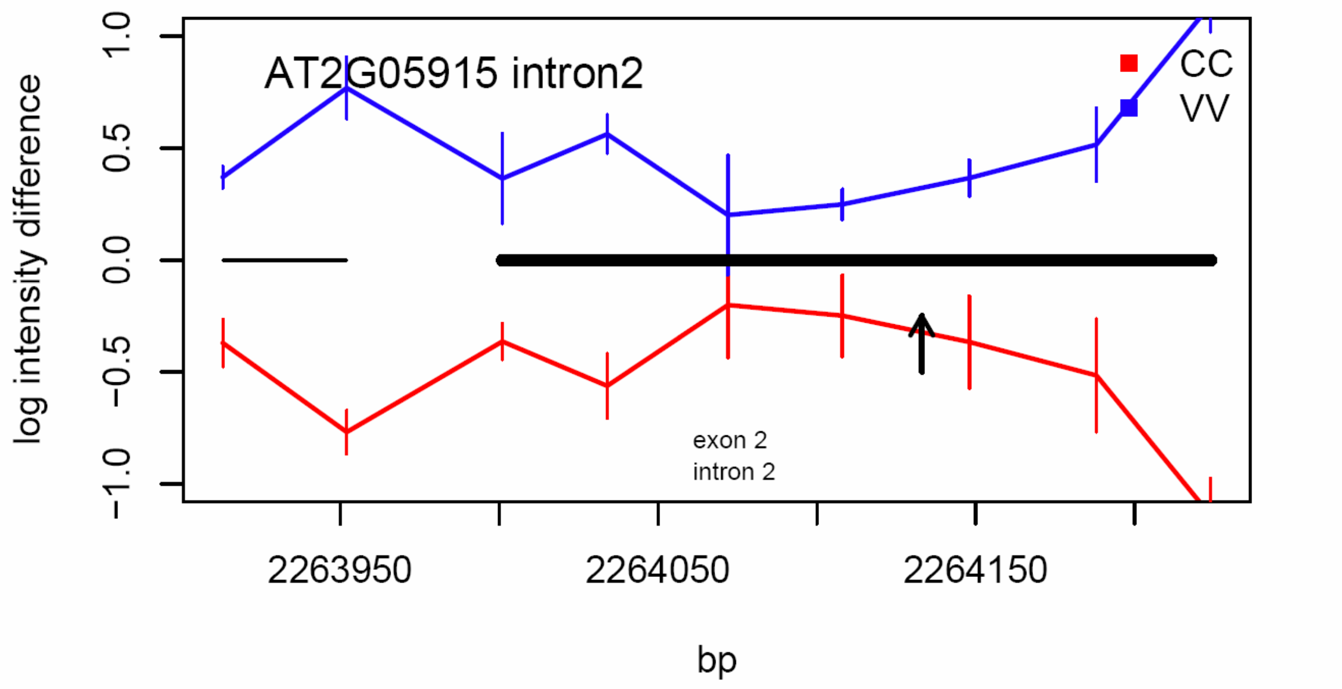

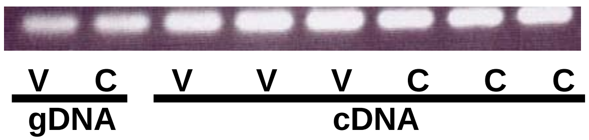

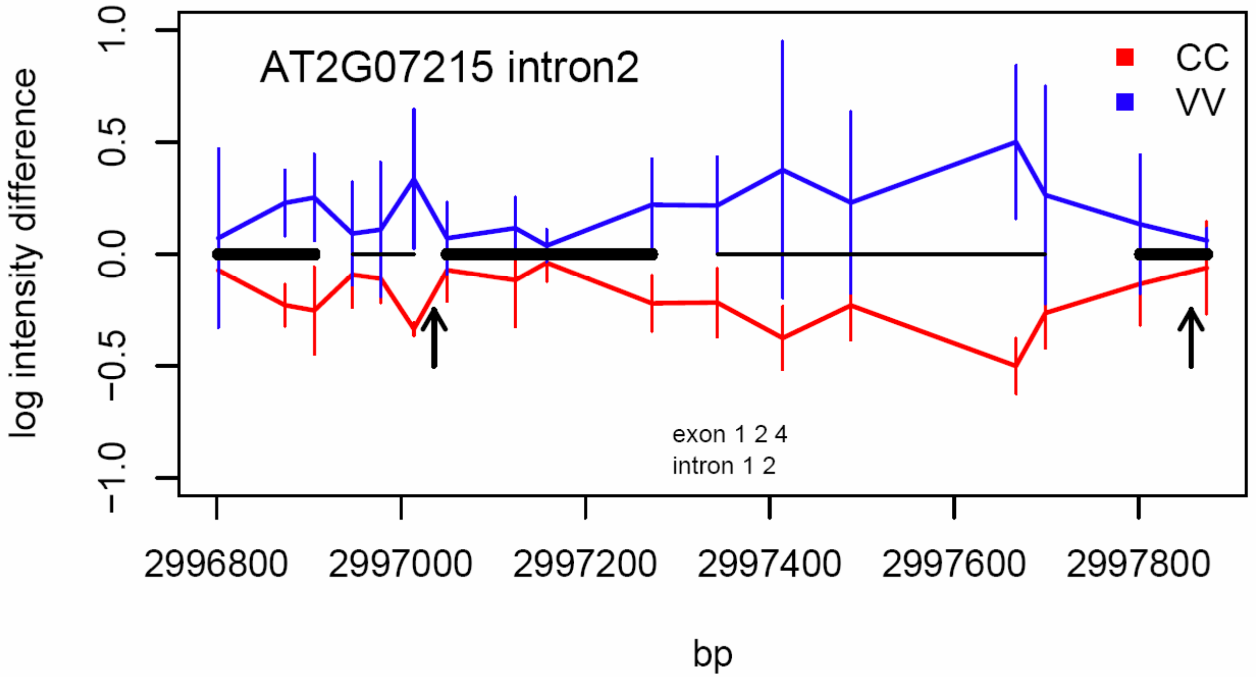
AT2G07215 intron2
CC
VV
log intensity difference
1.0
0.5
0.0
−0.5
−1.0
exon 1 2 4
intron 1 2
2996800
2997000
2997200
2997400
2997600
2997800
bp

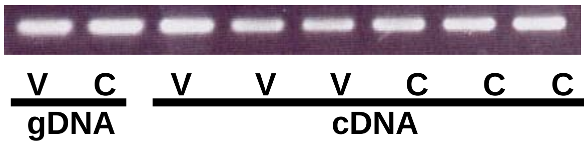
V C V V V C C C
gDNA
cDNA

√

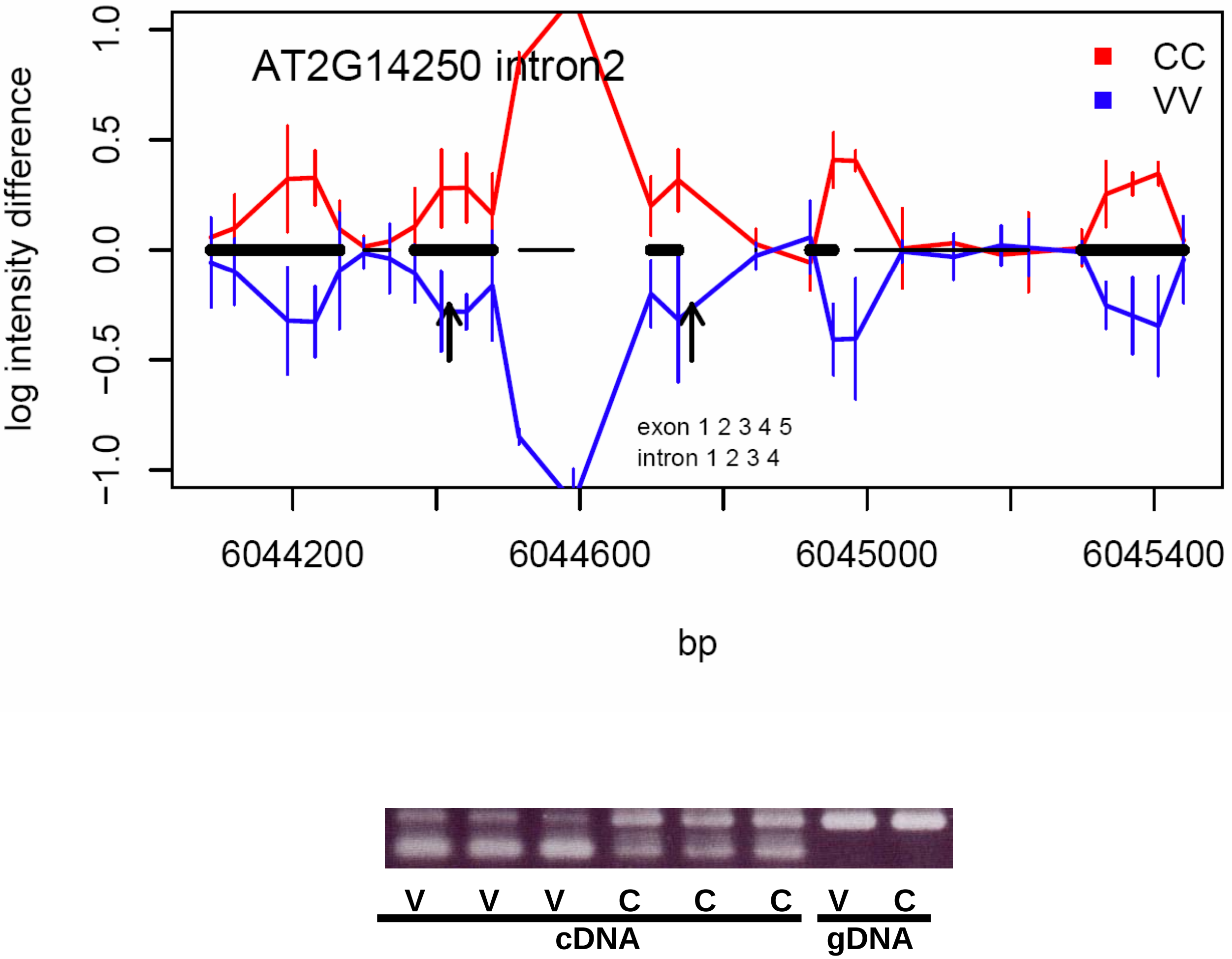
AT2G14250 intron2
CC
VV
log intensity difference
1.0
0.5
0.0
−0.5
−1.0
exon 1 2 3 4 5
intron 1 2 3 4
6044200
6044600
6045000
6045400
bp
V V V C C C V C
cDNA
gDNA

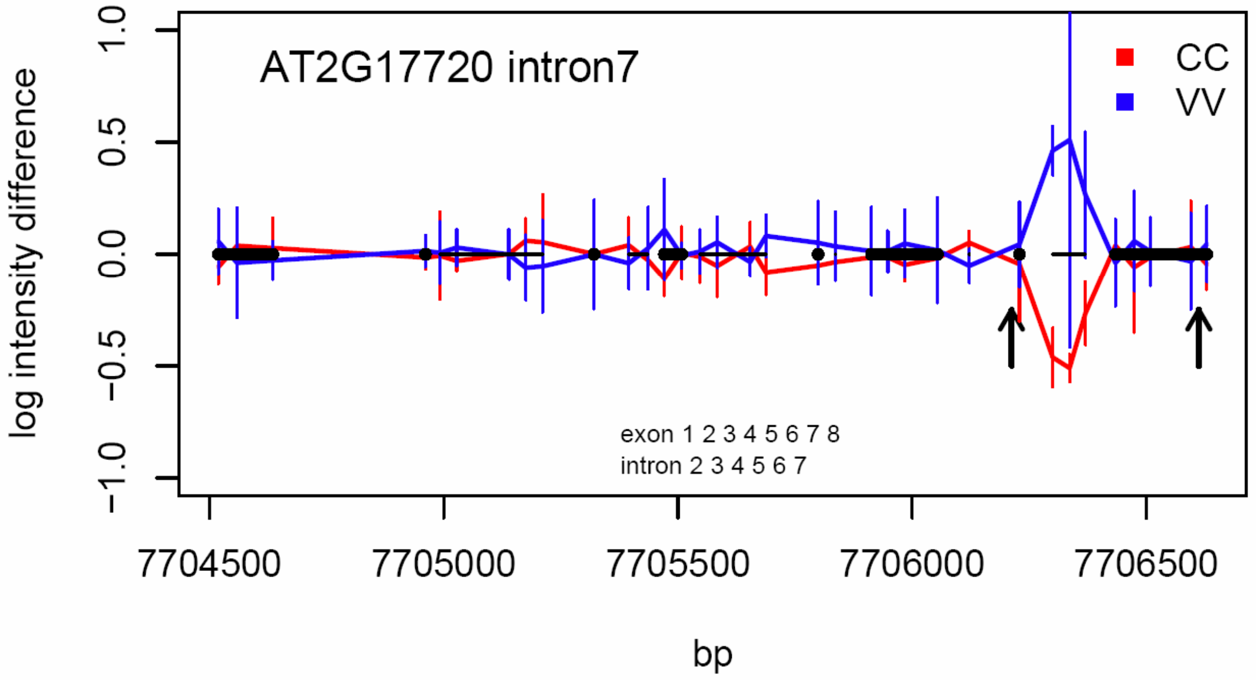
AT2G17720 intron7
CC
VV
log intensity difference
1.0
0.5
0.0
−0.5
−1.0
exon 1 2 3 4 5 6 7 8
intron 2 3 4 5 6 7
7704500
7705000
7705500
7706000
7706500
bp

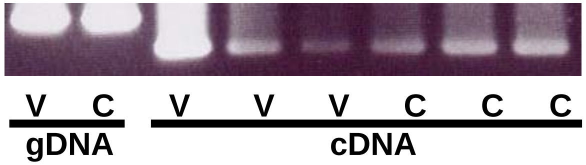
V C V V V C C C
gDNA
cDNA

√

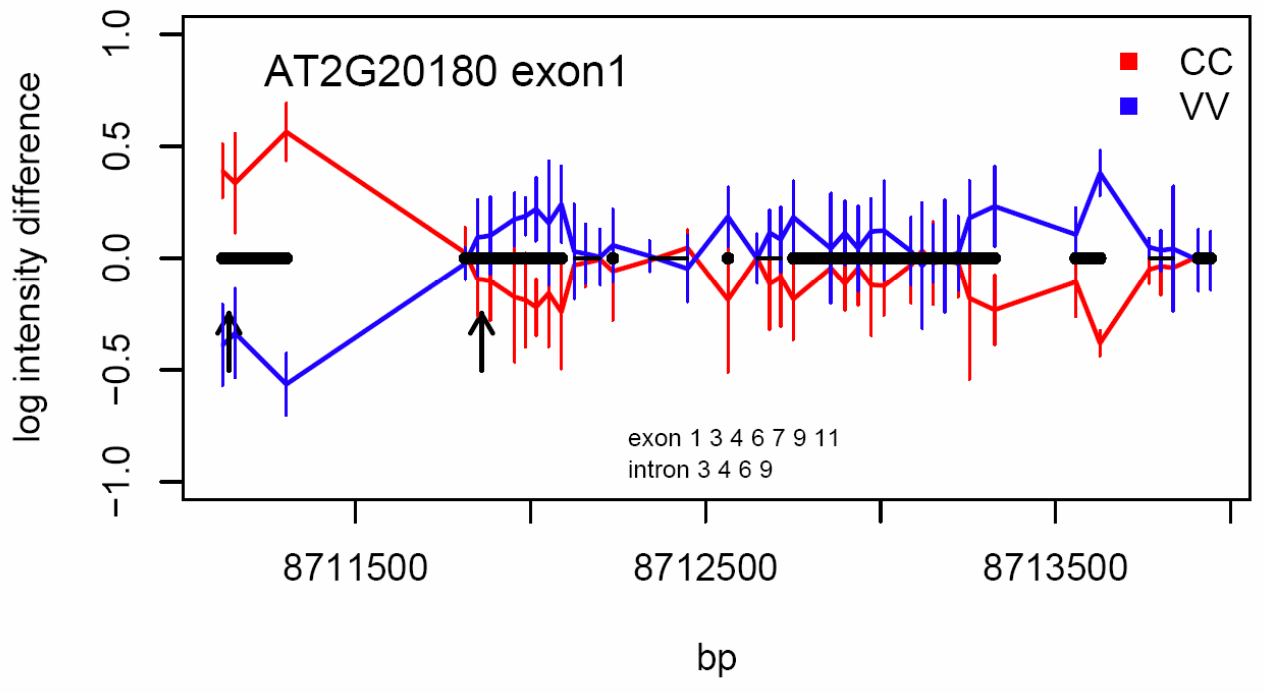

AT2G20180 exon1
CC
VV
log intensity difference
1.0
0.5
0.0
−0.5
−1.0
exon 1 3 4 6 7 9 11
intron 3 4 6 9
8711500
8712500
8713500
bp

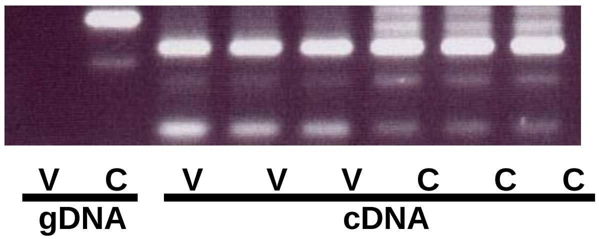

V C V V V C C C
gDNA
cDNA

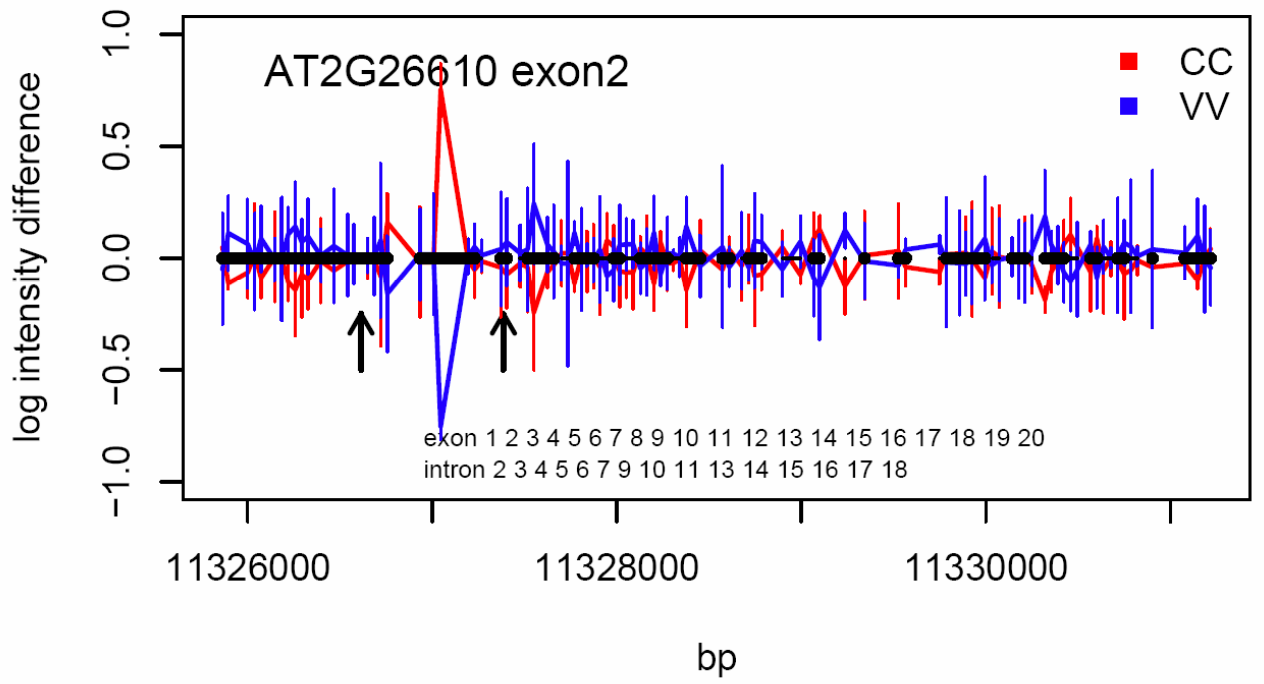

AT2G26610 exon2
CC
VV
log intensity difference
1.0
0.5
0.0
−0.5
−1.0
exon 1 2 3 4 5 6 7 8 9 10 11 12 13 14 15 16 17 18 19 20
intron 2 3 4 5 6 7 9 10 11 13 14 15 16 17 18
11326000
11328000
11330000
bp

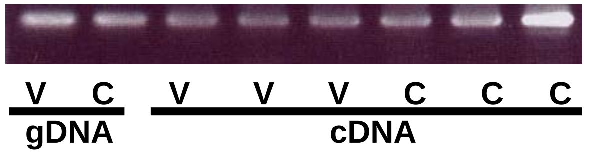

V C V V V C C C
gDNA
cDNA

√

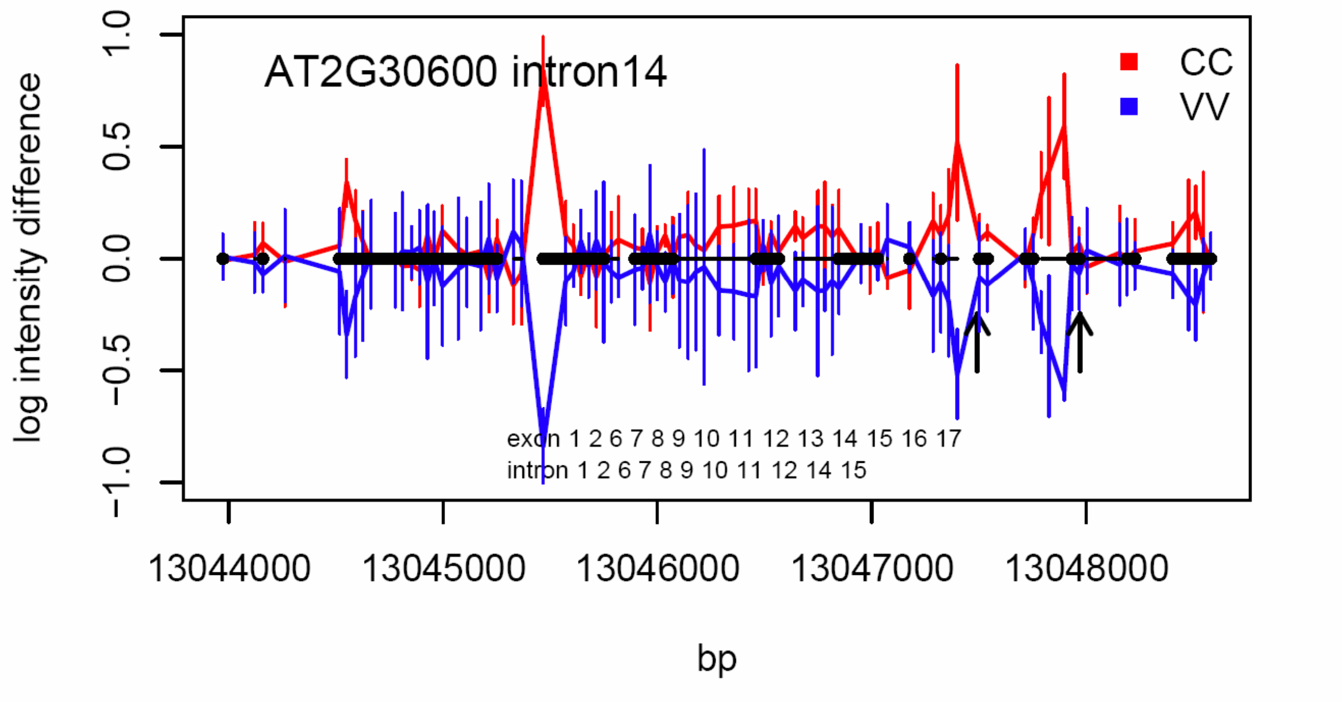
AT2G30600 intron14
CC
VV
log intensity difference
1.0
0.5
0.0
−0.5
−1.0
exon 1 2 6 7 8 9 10 11 12 13 14 15 16 17
intron 1 2 6 7 8 9 10 11 12 14 15
13044000
13045000
13046000
13047000
13048000
bp

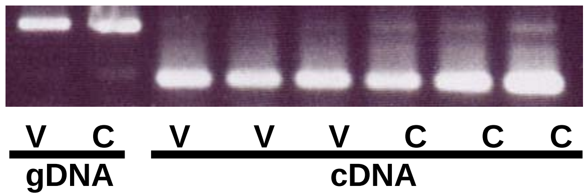
V C V V V C C C
gDNA
cDNA

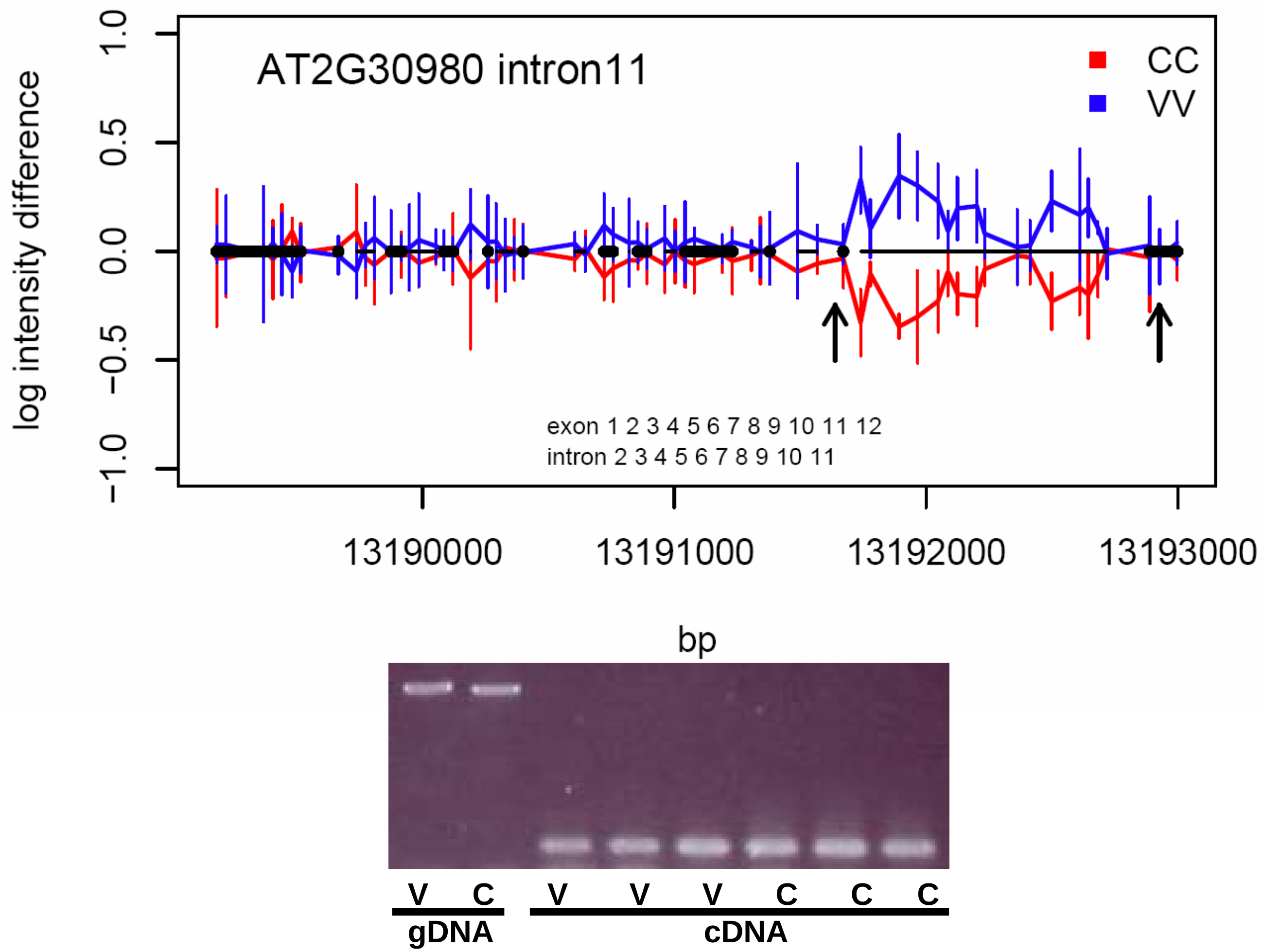
AT2G30980 intron11
CC
VV
log intensity difference
1.0
0.5
0.0
−0.5
−1.0
exon 1 2 3 4 5 6 7 8 9 10 11 12
intron 2 3 4 5 6 7 8 9 10 11
13190000
13191000
13192000
13193000
bp
V C V V V C C C
gDNA
cDNA

Not included for analysis

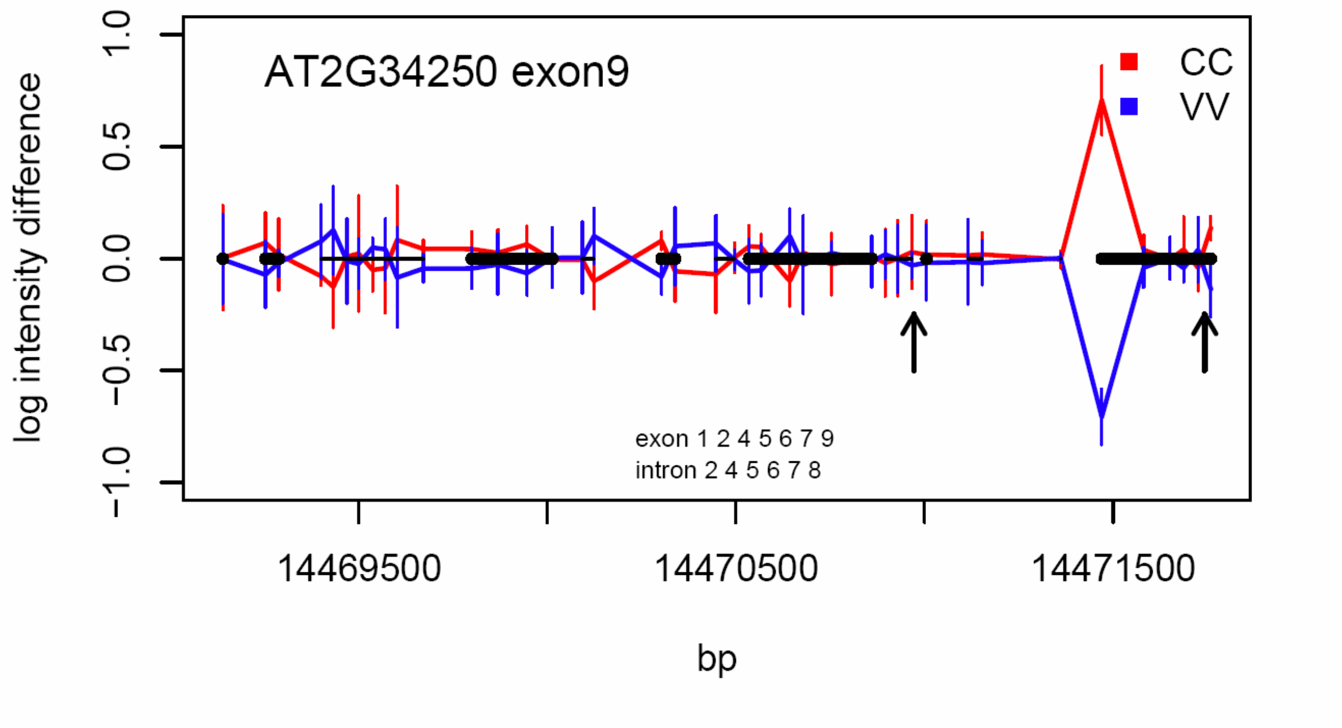

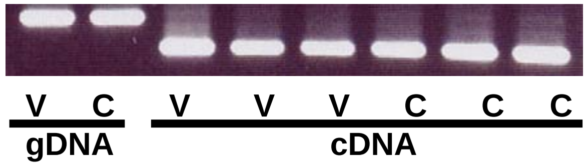

√

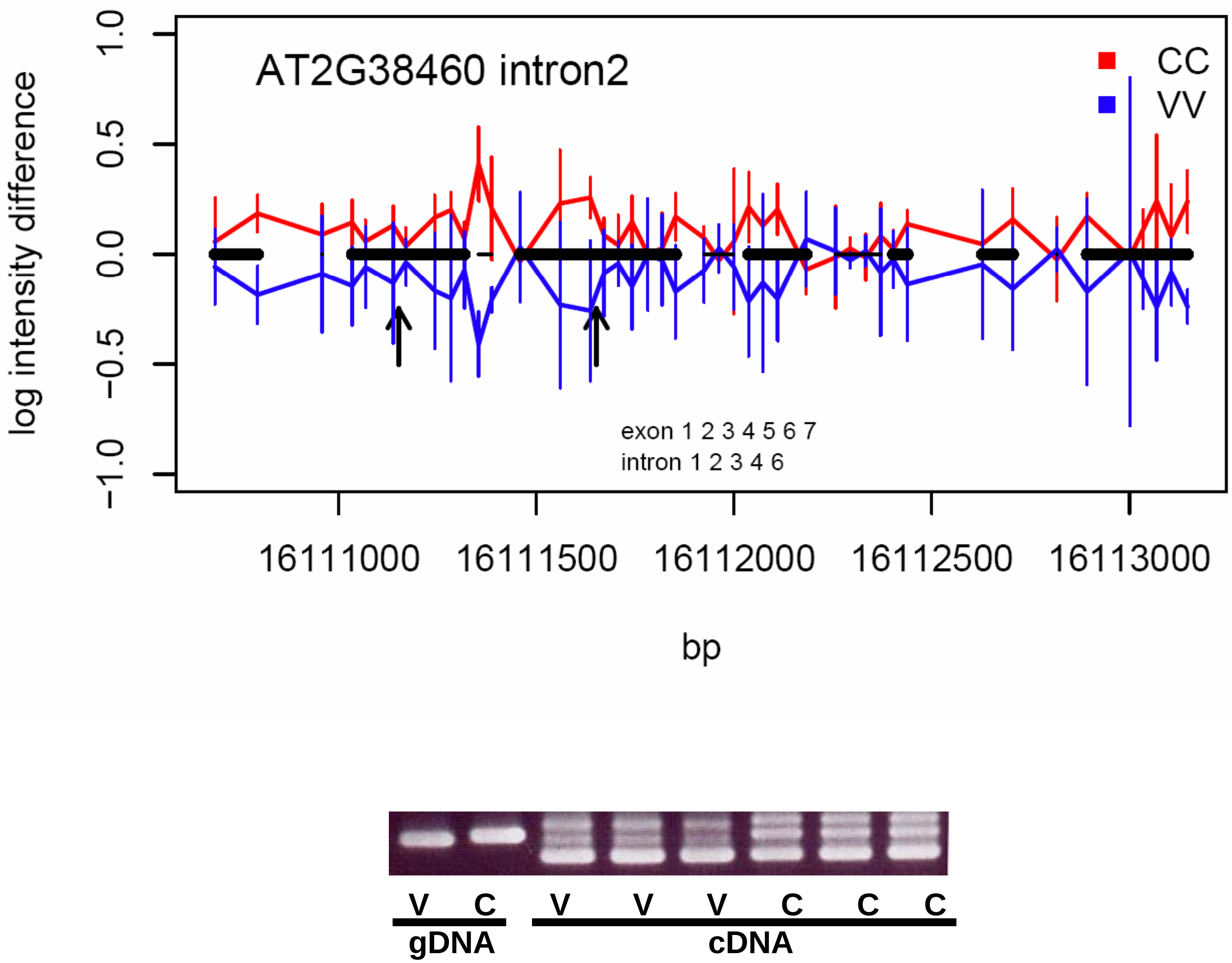
AT2G38460 intron2
CC
VV
log intensity difference
1.0
0.5
0.0
−0.5
−1.0
exon 1 2 3 4 5 6 7
intron 1 2 3 4 6
16111000
16111500
16112000
16112500
16113000
bp
V C V V V C C C
gDNA
cDNA

√

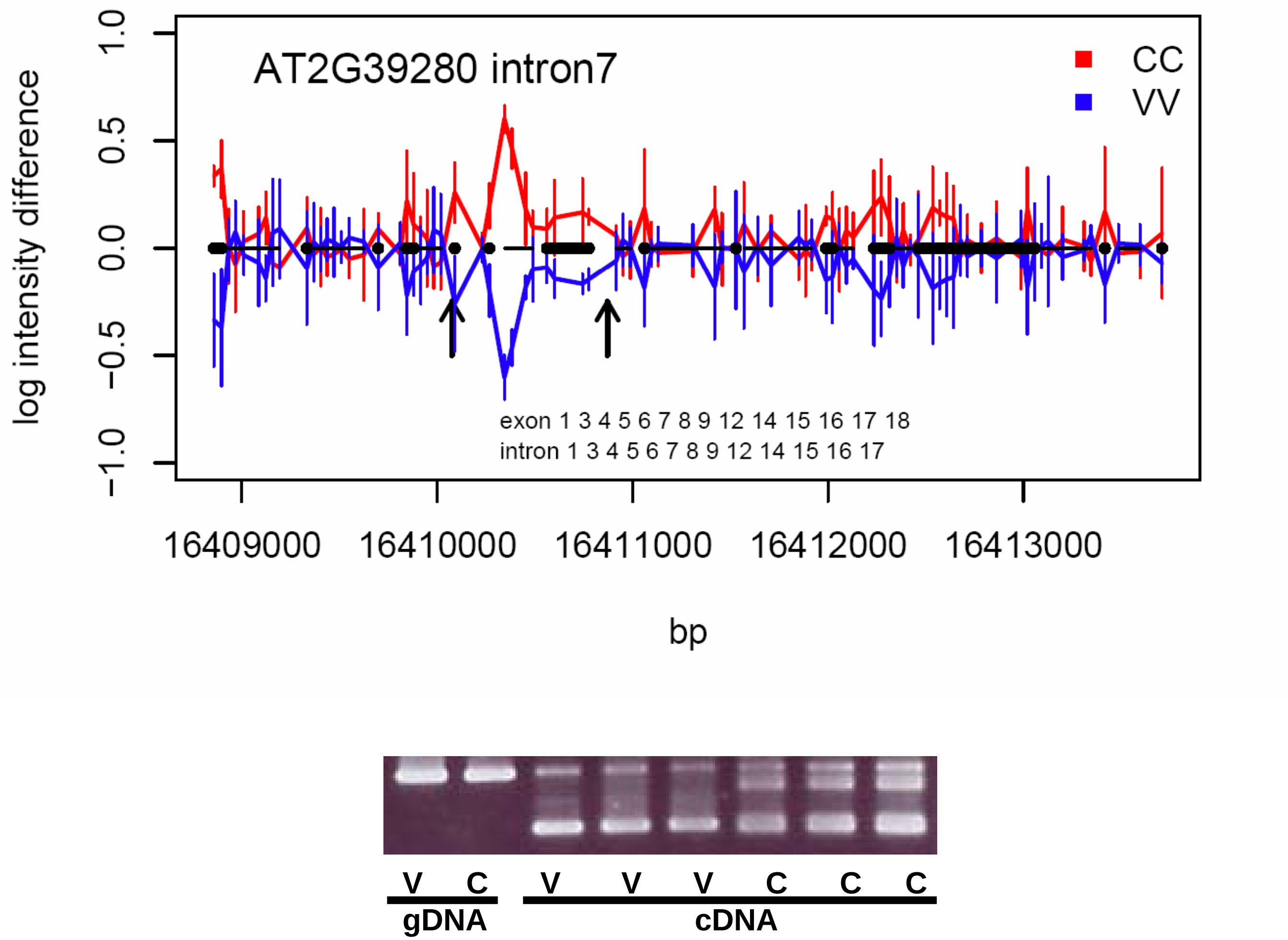
AT2G39280 intron7
CC
VV
log intensity difference
1.0
0.5
0.0
−0.5
−1.0
exon 1 3 4 5 6 7 8 9 12 14 15 16 17 18
intron 1 3 4 5 6 7 8 9 12 14 15 16 17
16409000
16410000
16411000
16412000
16413000
bp
V C V V V C C C
gDNA
cDNA

√

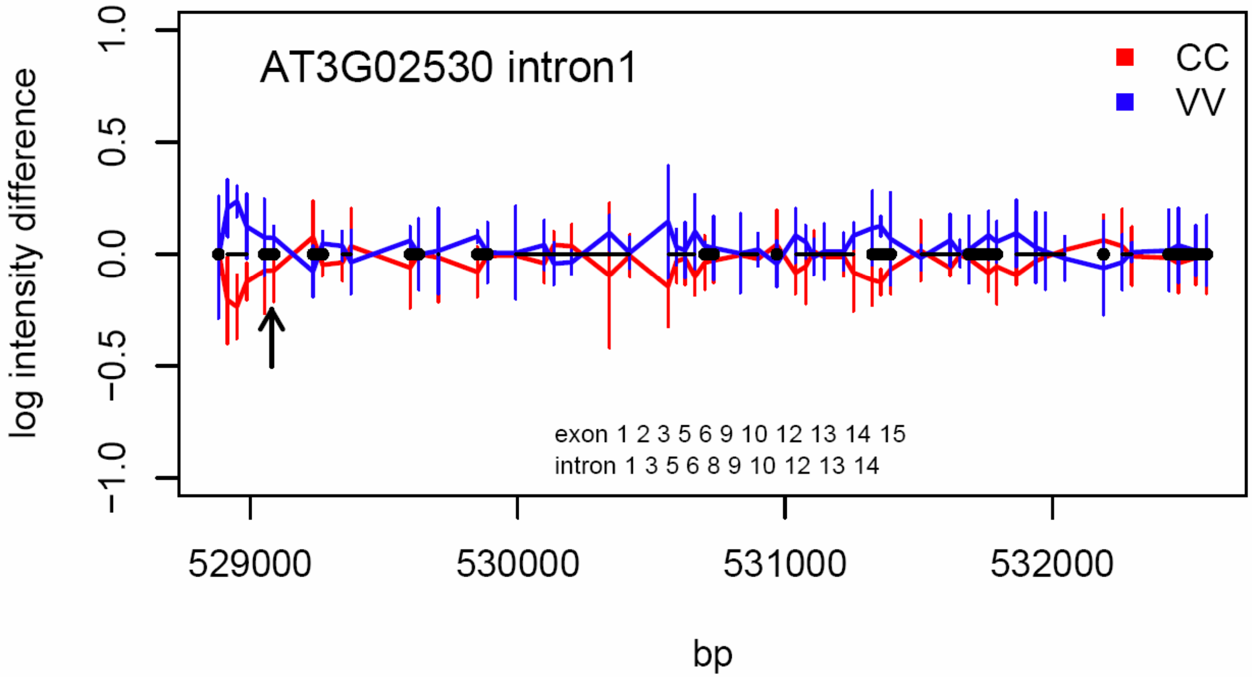
AT3G02530 intron1
CC
VV
log intensity difference
1.0
0.5
0.0
−0.5
−1.0
exon 1 2 3 5 6 9 10 12 13 14 15
intron 1 3 5 6 8 9 10 12 13 14
529000
530000
531000
532000
bp

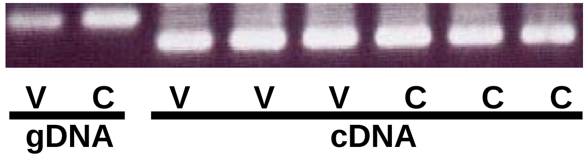
V C V V V C C C
gDNA
cDNA

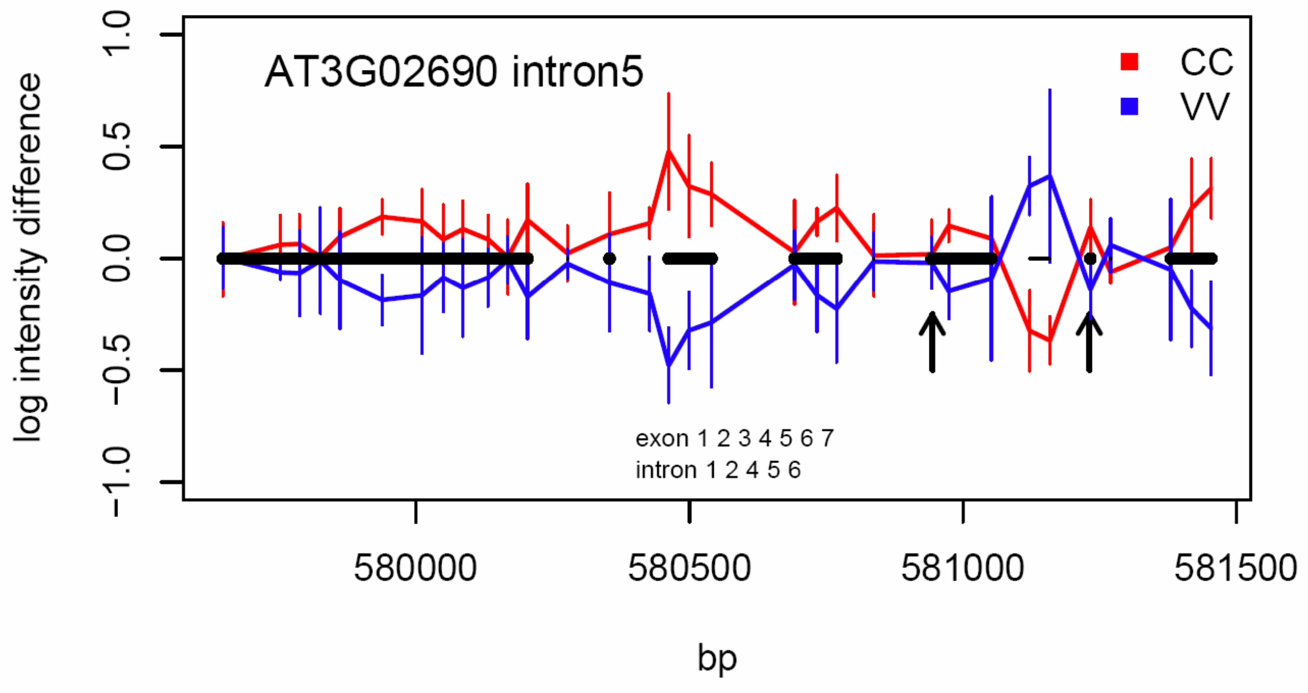
AT3G02690 intron5
CC
VV
log intensity difference
1.0
0.5
0.0
−0.5
−1.0
exon 1 2 3 4 5 6 7
intron 1 2 4 5 6
580000
580500
581000
581500
bp

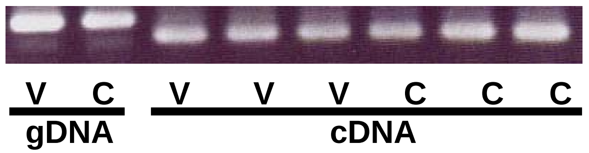
V C V V V C C C
gDNA
cDNA

√

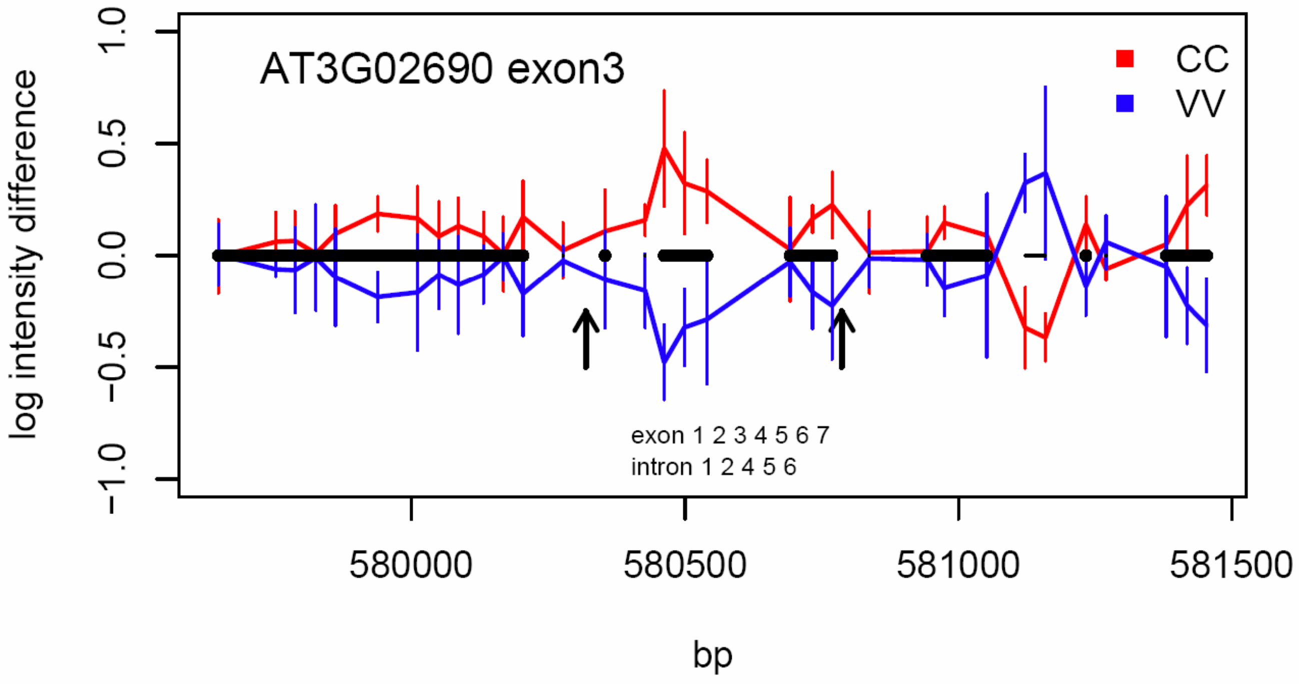
AT3G02690 exon3
CC
VV
log intensity difference
1.0
0.5
0.0
−0.5
−1.0
exon 1 2 3 4 5 6 7
intron 1 2 4 5 6
580000
580500
581000
581500
bp

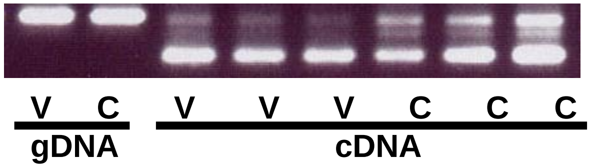
V C V V V C C C
gDNA
cDNA

√

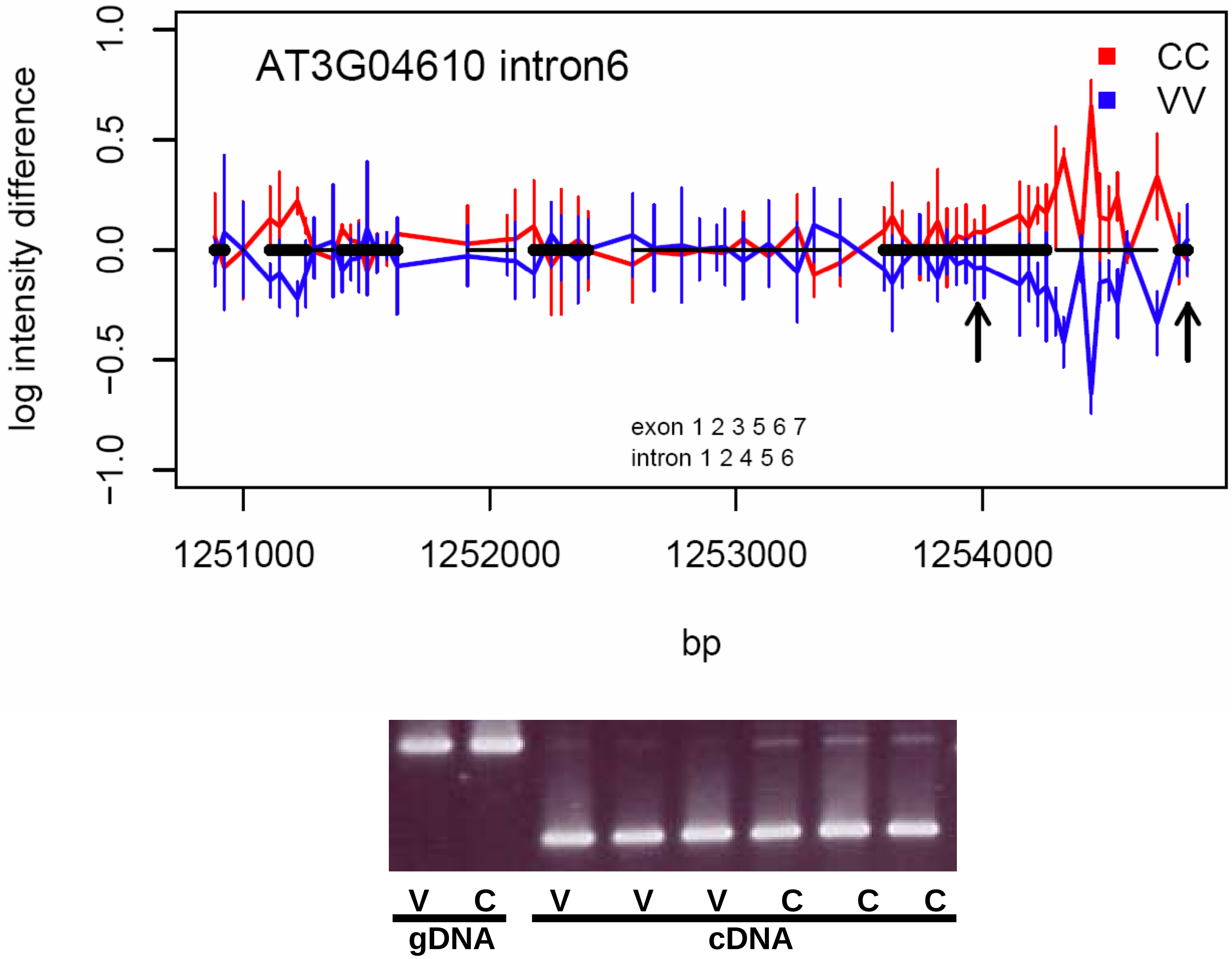
AT3G04610 intron6
CC
VV
log intensity difference
1.0
0.5
0.0
−0.5
−1.0
exon 1 2 3 5 6 7
intron 1 2 4 5 6
1251000
1252000
1253000
1254000
bp
V C V V V C C C
gDNA
cDNA

√

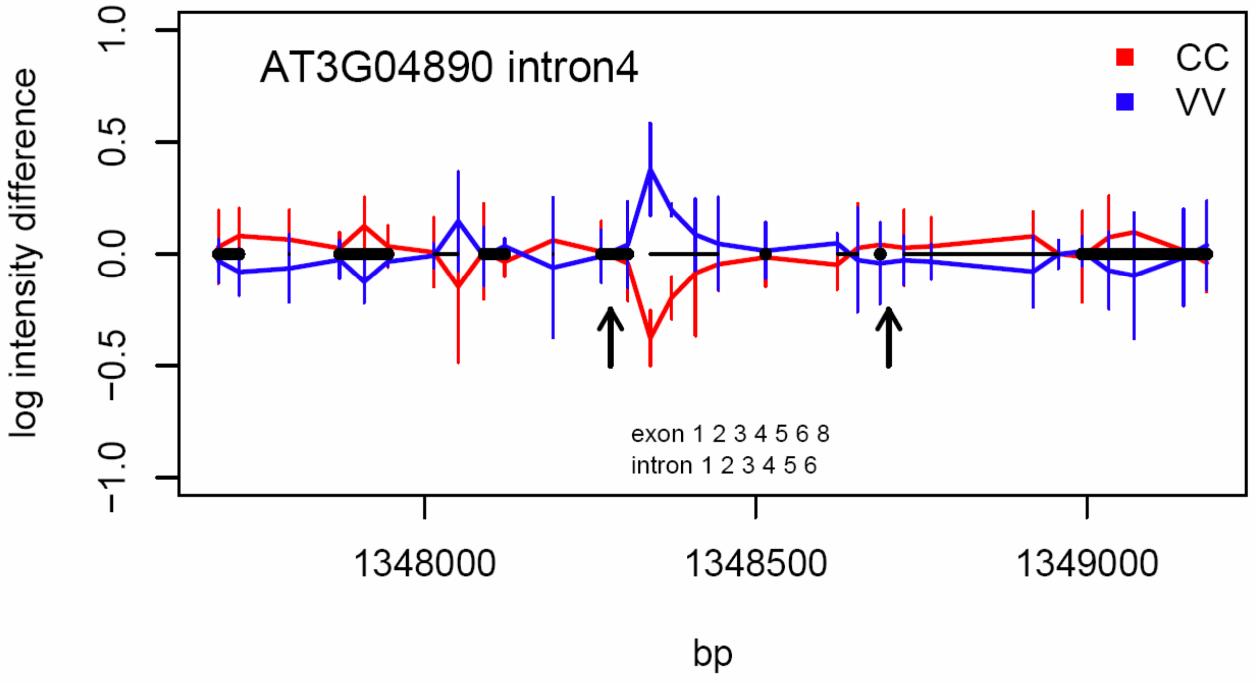
AT3G04890 intron4
CC
VV
log intensity difference
1.0
0.5
0.0
−0.5
−1.0
exon 1 2 3 4 5 6 8
intron 1 2 3 4 5 6
1348000
1348500
1349000
bp

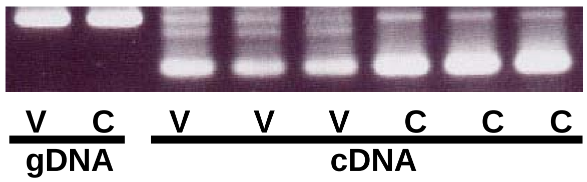
V C V V V C C C
gDNA
cDNA

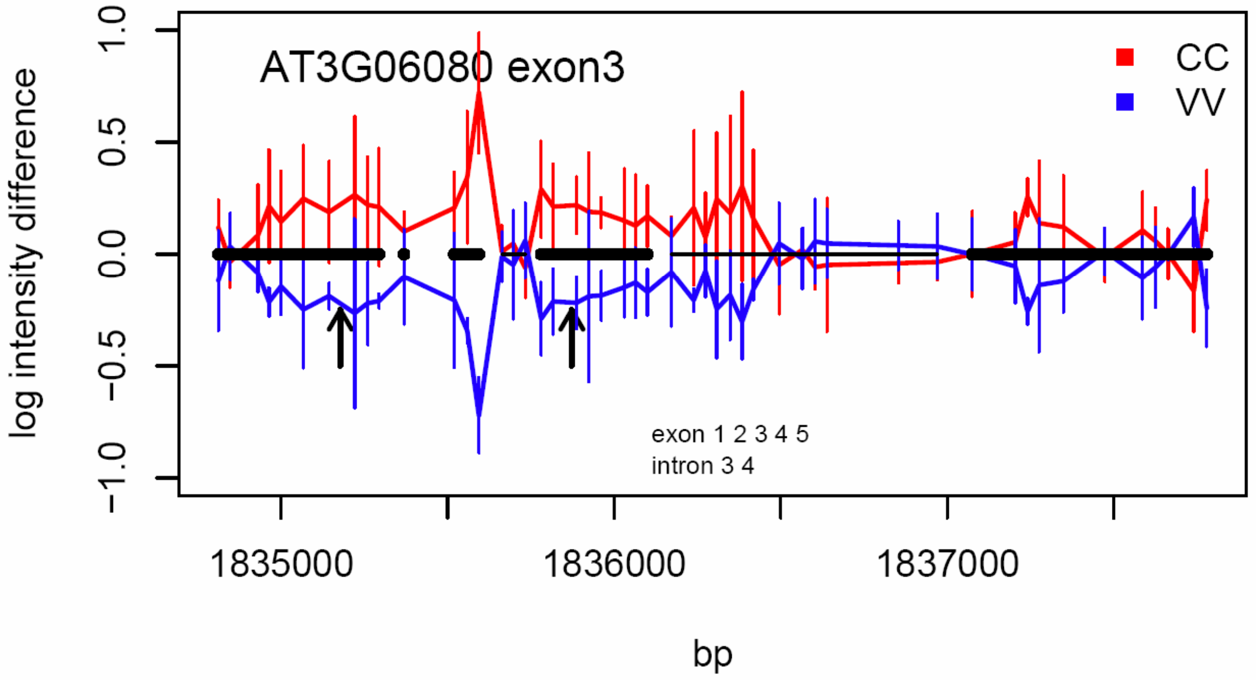
AT3G06080 exon3
CC
VV
log intensity difference
1.0
0.5
0.0
−0.5
−1.0
exon 1 2 3 4 5
intron 3 4
1835000
1836000
1837000
bp

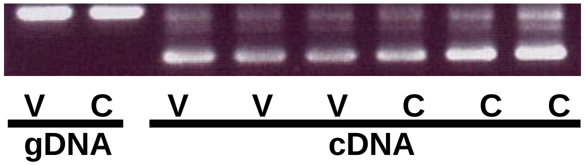
V C V V V C C C
gDNA
cDNA

√

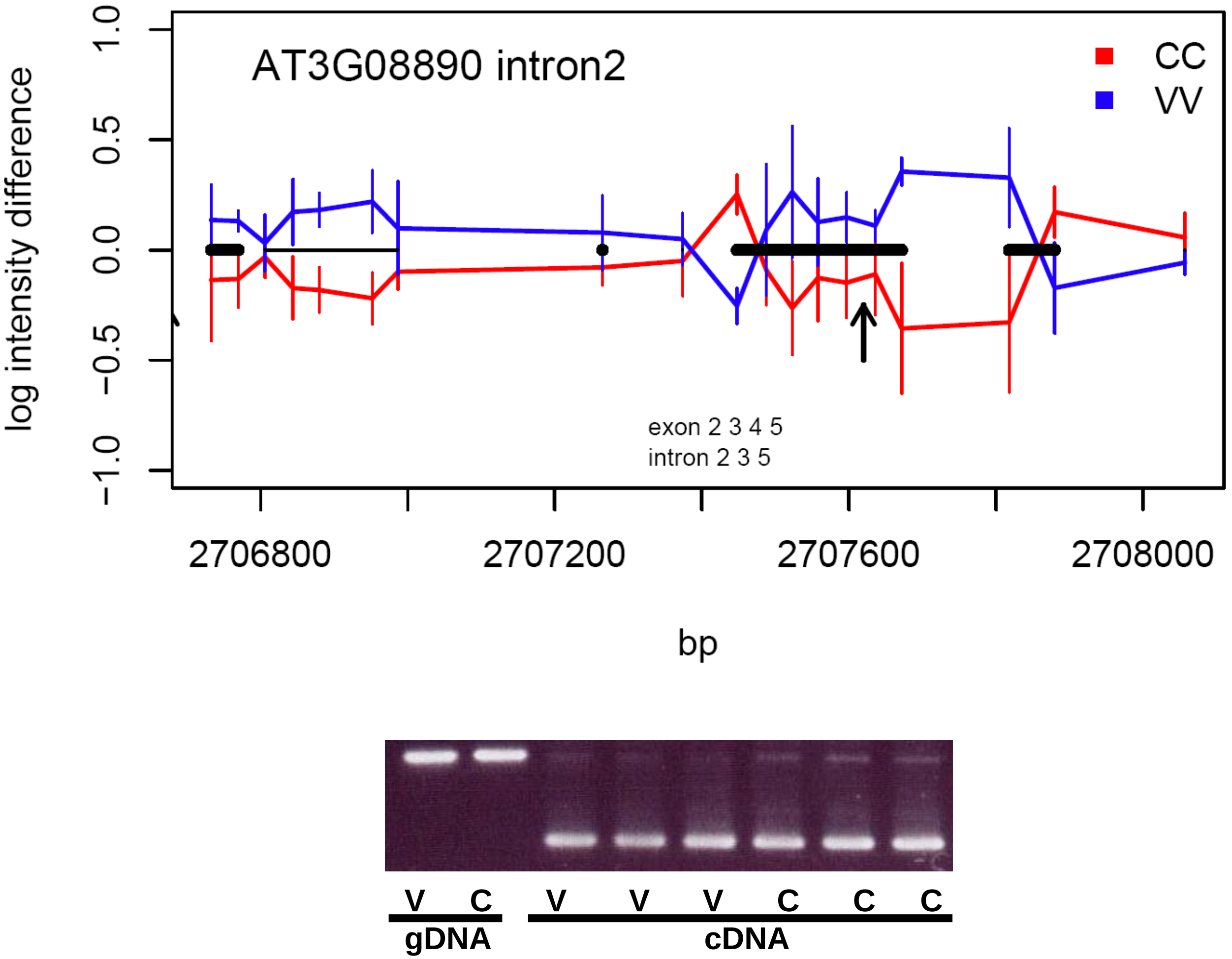
AT3G08890 intron2
CC
VV
log intensity difference
1.0
0.5
0.0
−0.5
−1.0
exon 2 3 4 5
intron 2 3 5
2706800
2707200
2707600
2708000
bp
V C V V V C C C
gDNA
cDNA

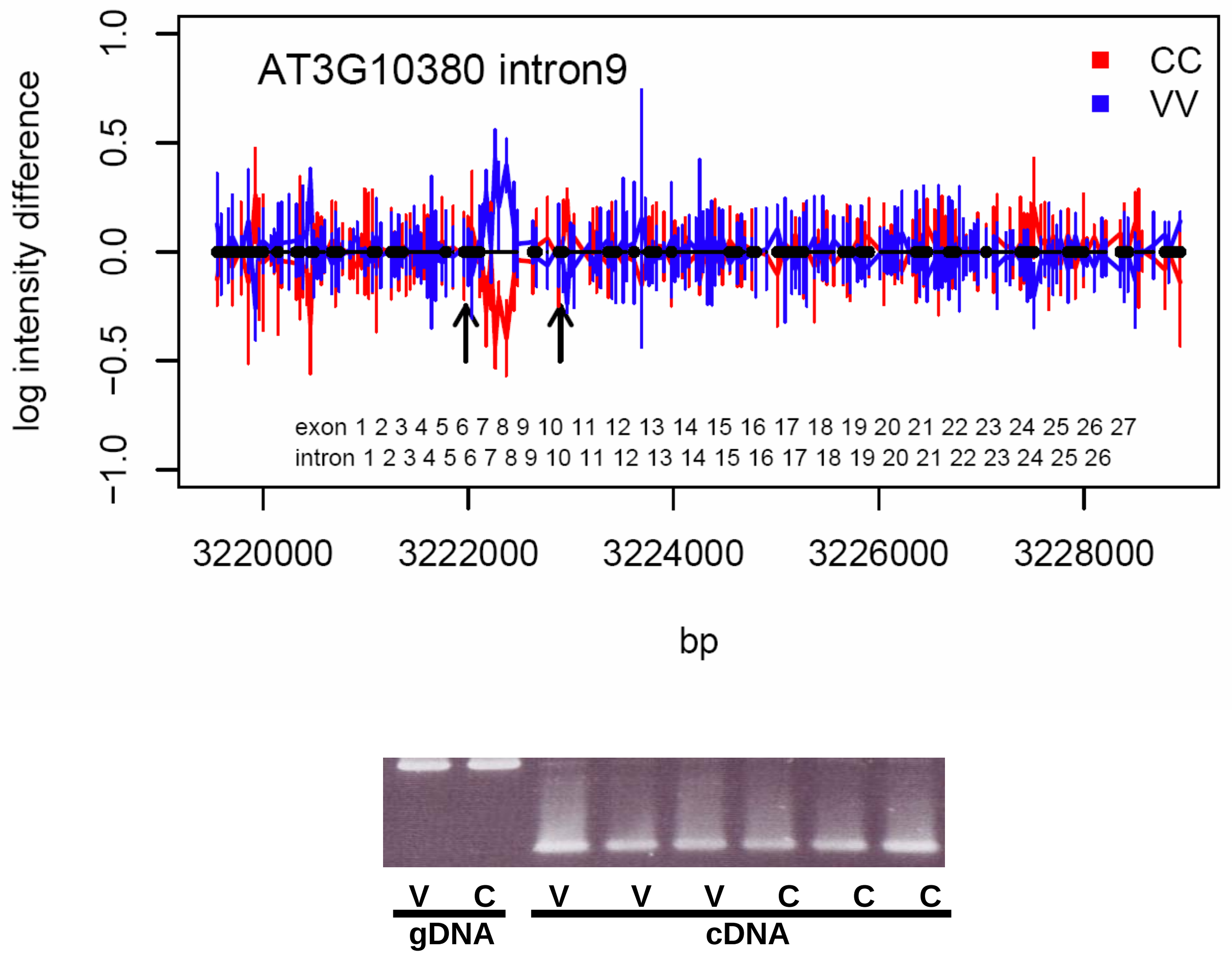
AT3G10380 intron9
CC
VV
log intensity difference
1.0
0.5
0.0
−0.5
−1.0
exon 1 2 3 4 5 6 7 8 9 10 11 12 13 14 15 16 17 18 19 20 21 22 23 24 25 26 27
intron 1 2 3 4 5 6 7 8 9 10 11 12 13 14 15 16 17 18 19 20 21 22 23 24 25 26
3220000
3222000
3224000
3226000
3228000
bp
V C V V V C C C
gDNA
cDNA

√

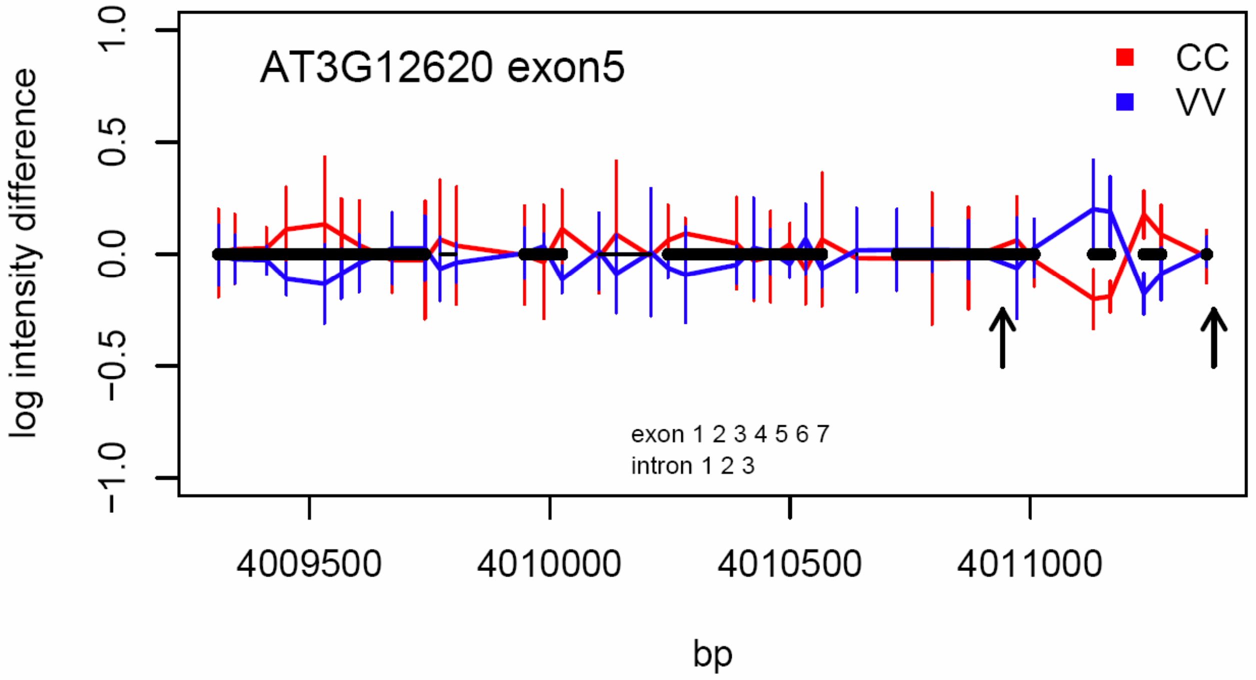
AT3G12620 exon5
CC
VV
log intensity difference
1.0
0.5
0.0
−0.5
−1.0
exon 1 2 3 4 5 6 7
intron 1 2 3
4009500
4010000
4010500
4011000
bp

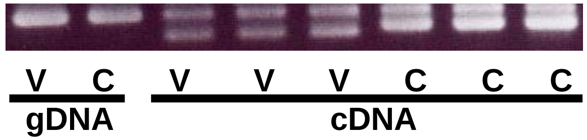
V C V V V C C C
gDNA
cDNA

√

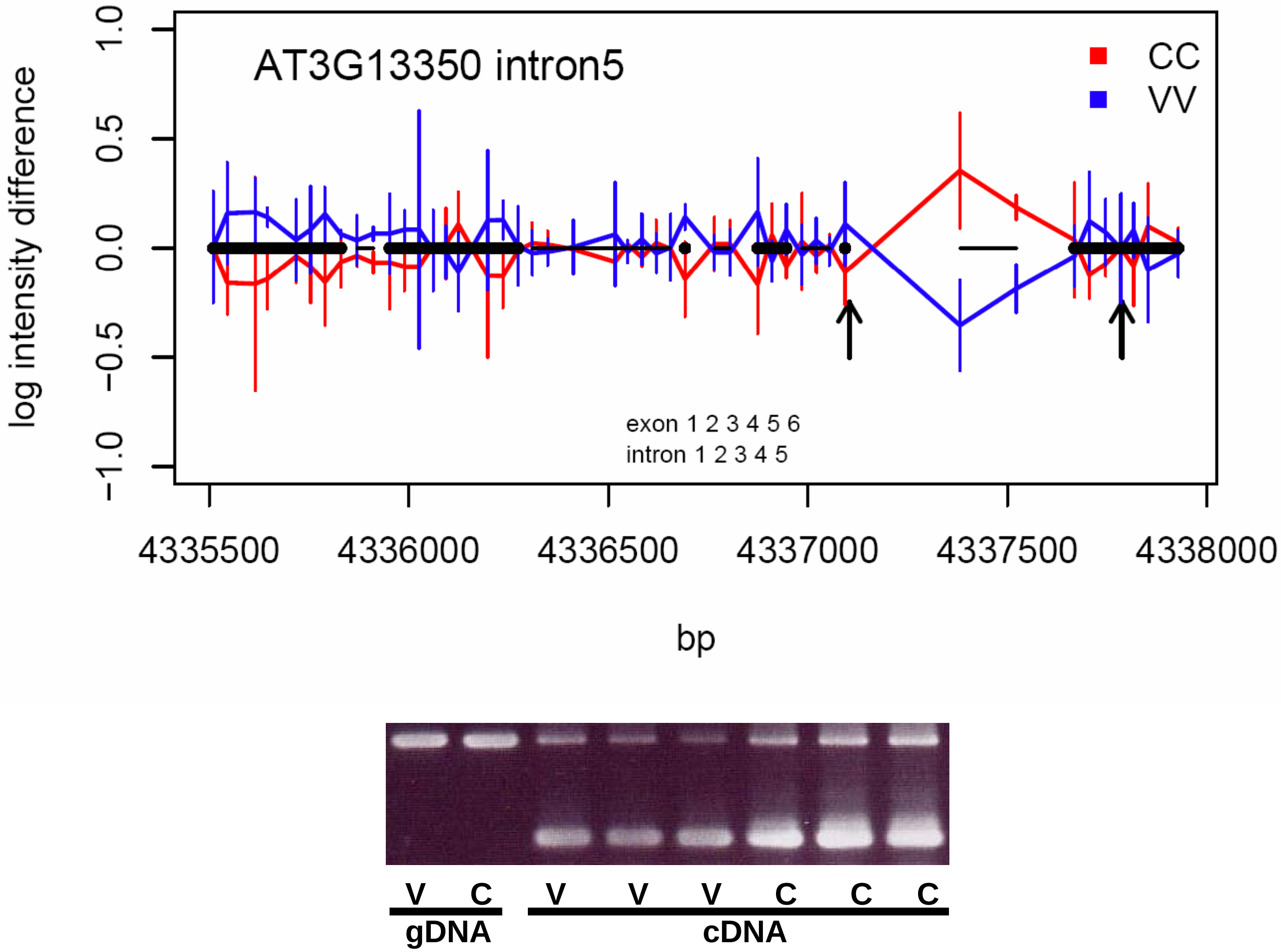

AT3G13350 intron5
CC
VV
log intensity difference
1.0
0.5
0.0
−0.5
−1.0
exon 1 2 3 4 5 6
intron 1 2 3 4 5
4335500
4336000
4336500
4337000
4337500
4338000
bp
V C V V V C C C
gDNA
cDNA

√

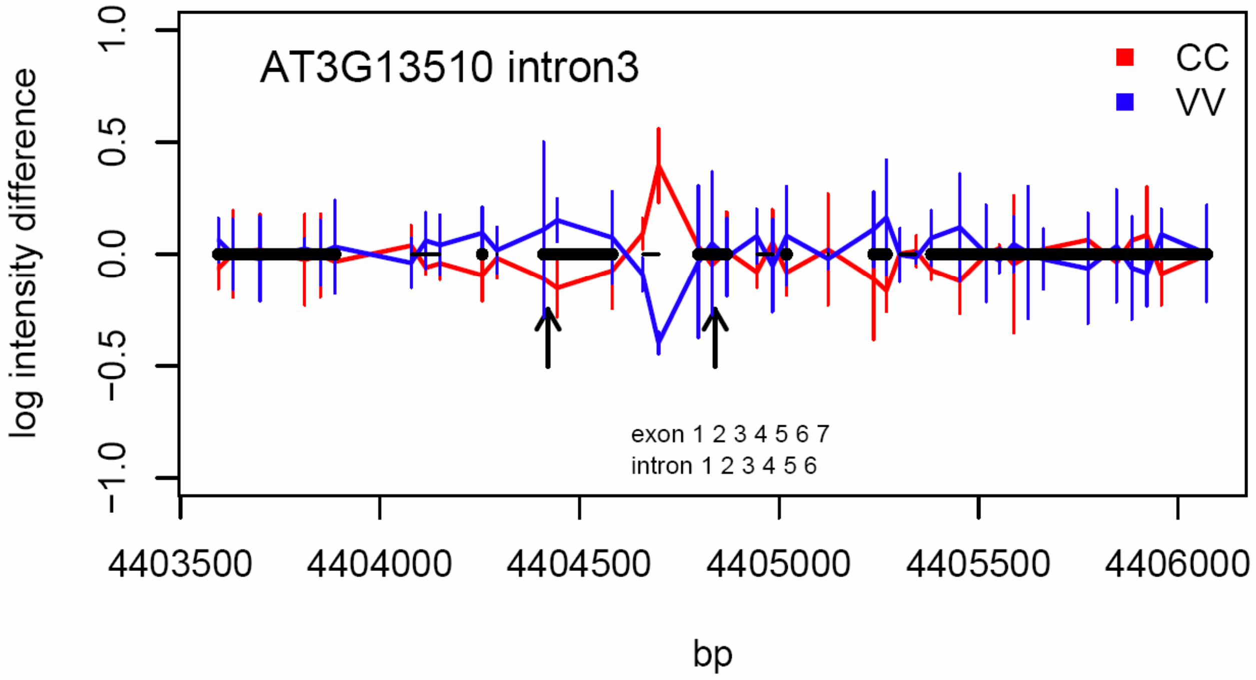
AT3G13510 intron3
CC
VV
log intensity difference
1.0
0.5
0.0
−0.5
−1.0
exon 1 2 3 4 5 6 7
intron 1 2 3 4 5 6
4403500
4404000
4404500
4405000
4405500
4406000
bp

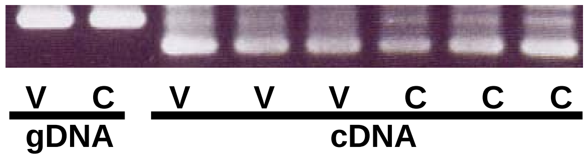
V C V V V C C C
gDNA
cDNA

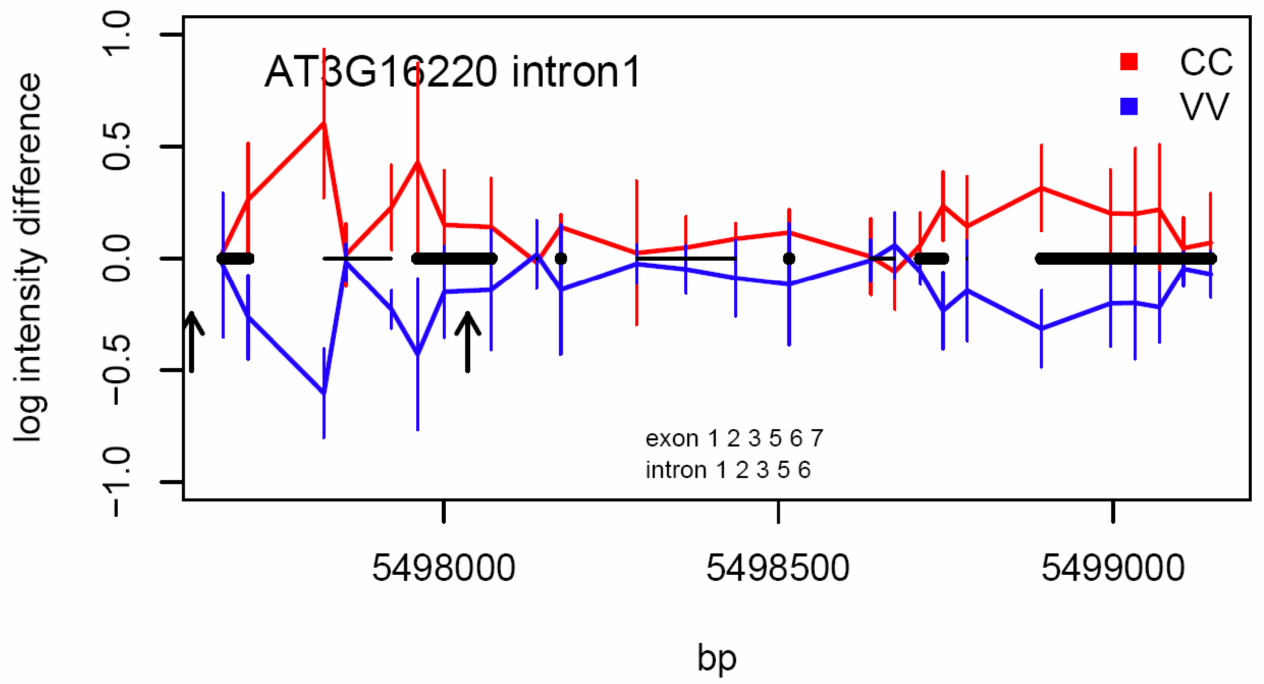
AT3G16220 intron1
CC
VV
log intensity difference
1.0
0.5
0.0
−0.5
−1.0
exon 1 2 3 5 6 7
intron 1 2 3 5 6
5498000
5498500
5499000
bp

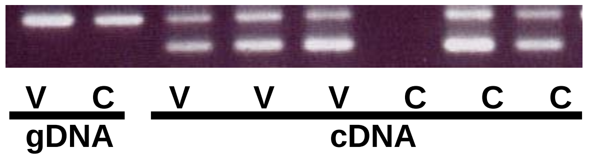
V C V V V C C C
gDNA
cDNA

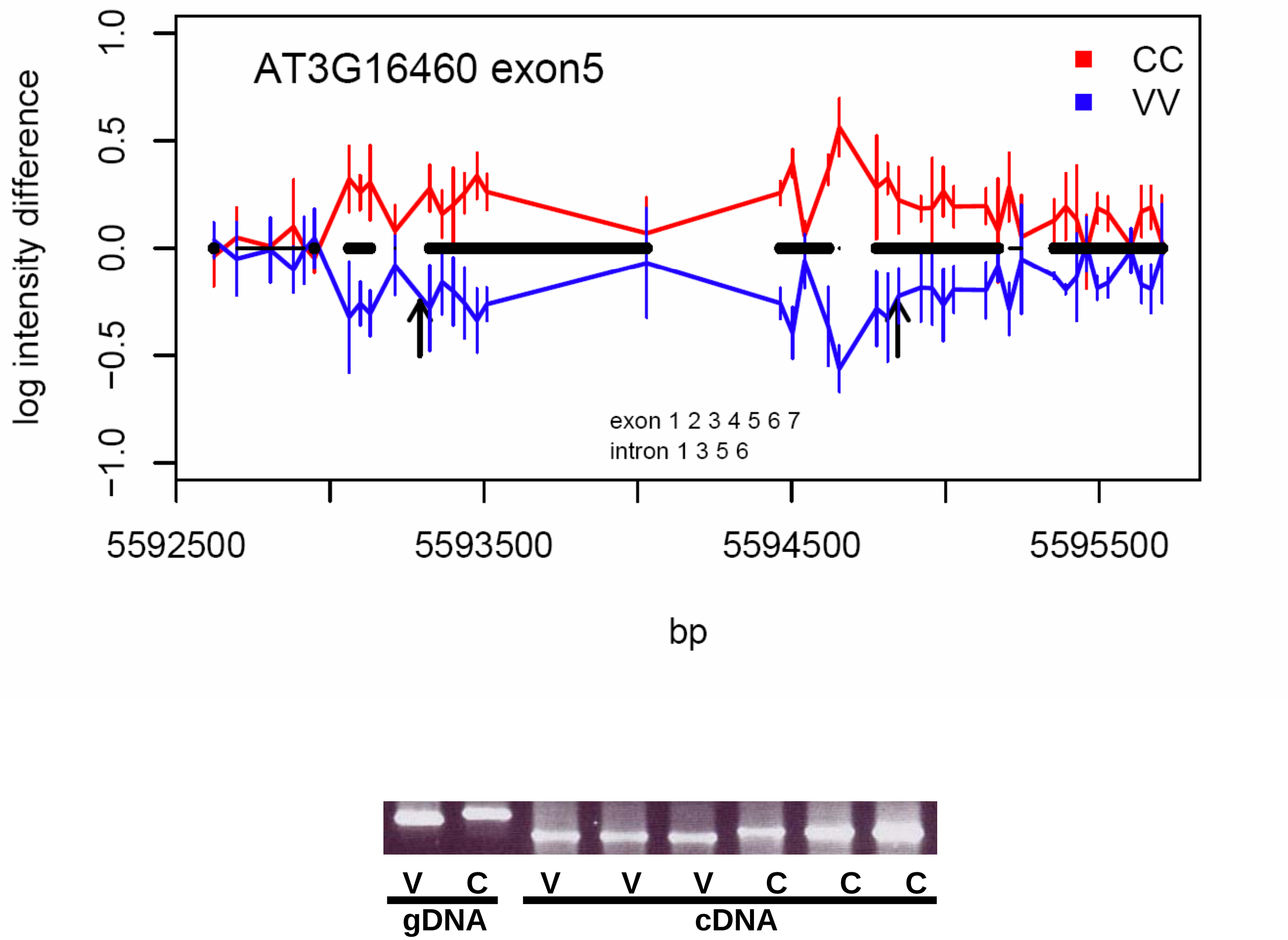
AT3G16460 exon5
CC
VV
log intensity difference
1.0
0.5
0.0
−0.5
−1.0
exon 1 2 3 4 5 6 7
intron 1 3 5 6
5592500
5593500
5594500
5595500
bp
V C V V V C C C
gDNA
cDNA

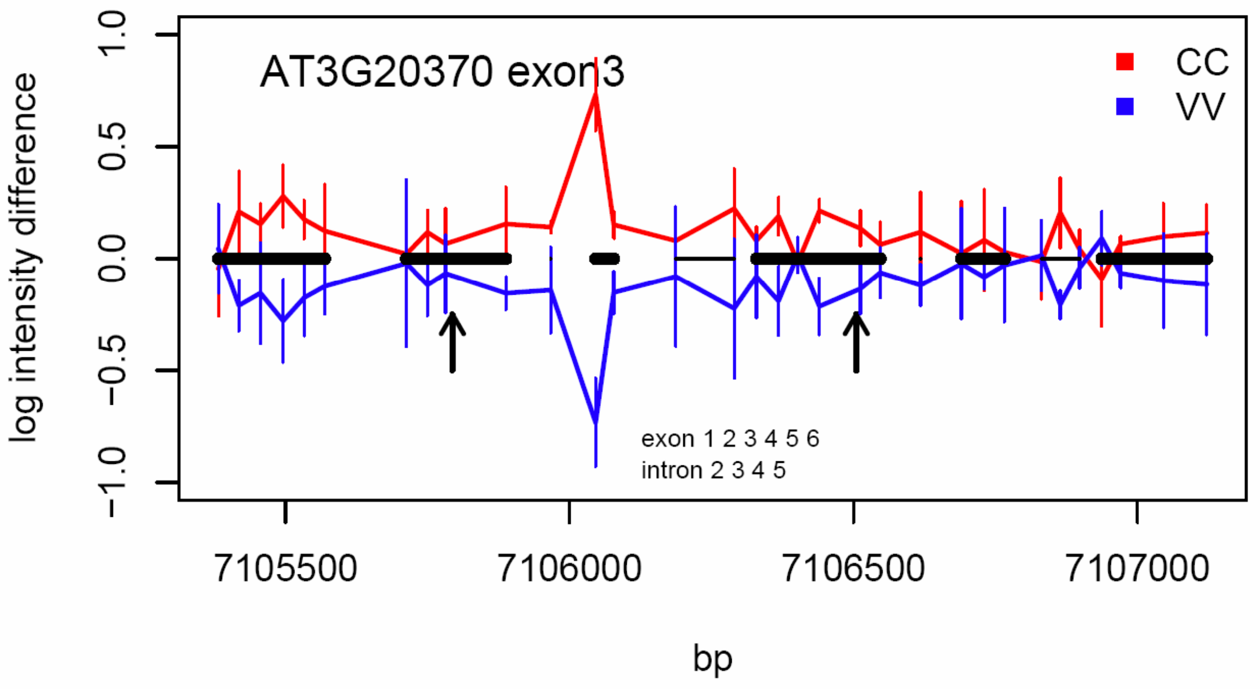
AT3G20370 exon3
CC
VV
log intensity difference
1.0
0.5
0.0
−0.5
−1.0
exon 1 2 3 4 5 6
intron 2 3 4 5
7105500
7106000
7106500
7107000
bp

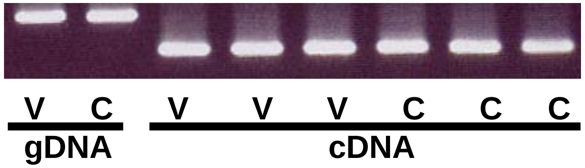
V C V V V C C C
gDNA
cDNA

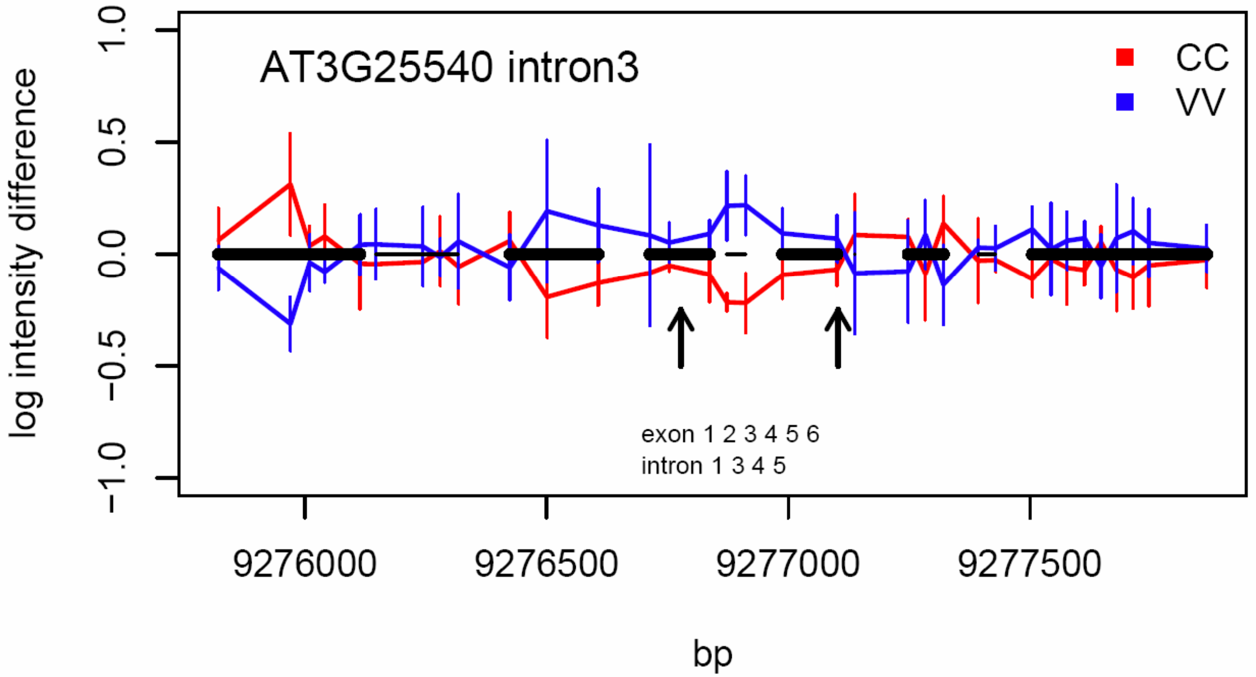
AT3G25540 intron3
CC
VV
log intensity difference
1.0
0.5
0.0
−0.5
−1.0
exon 1 2 3 4 5 6
intron 1 3 4 5
9276000
9276500
9277000
9277500
bp

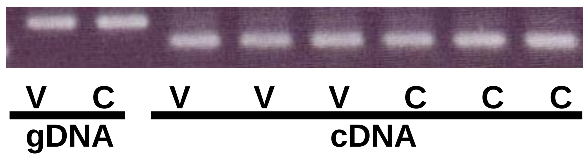
V C V V V C C C
gDNA
cDNA

Not included for analysis

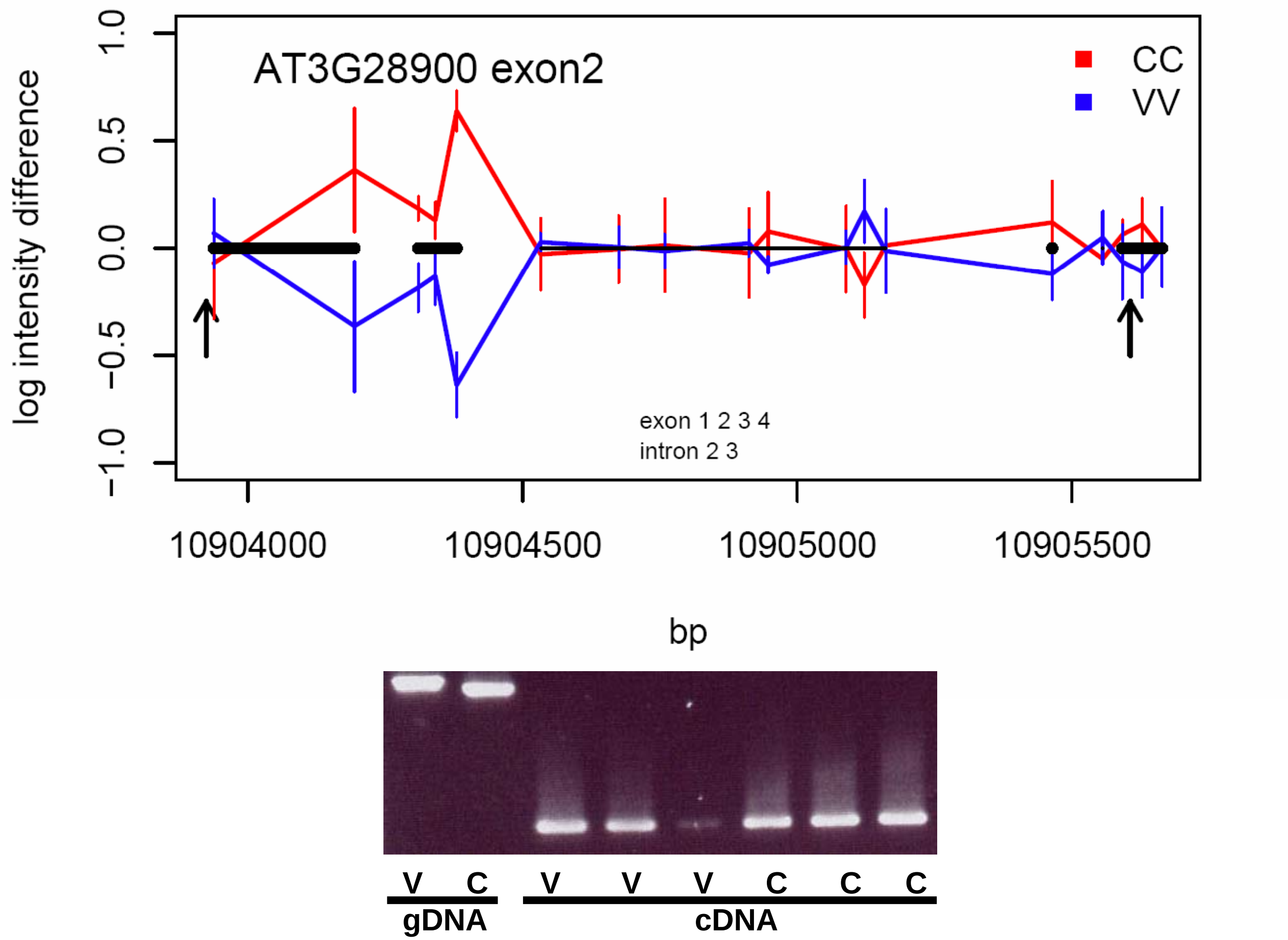

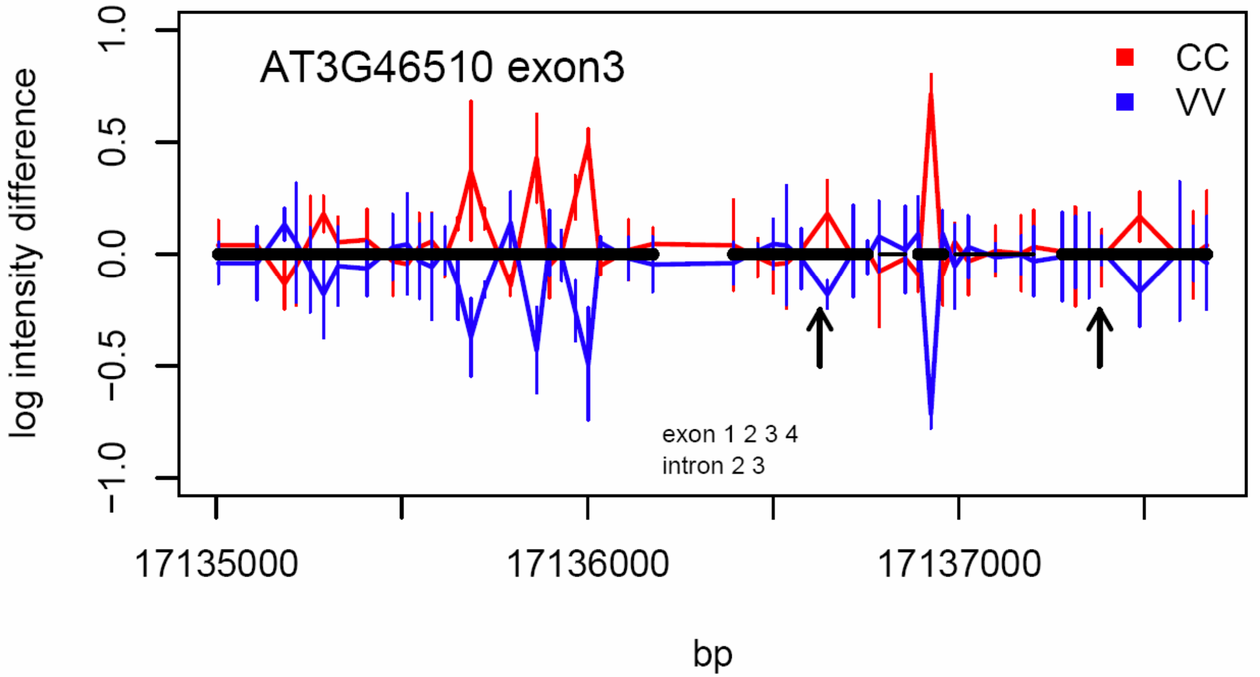
AT3G46510 exon3
CC
VV
log intensity difference
1.0
0.5
0.0
−0.5
−1.0
exon 1 2 3 4
intron 2 3
17135000
17136000
17137000
bp

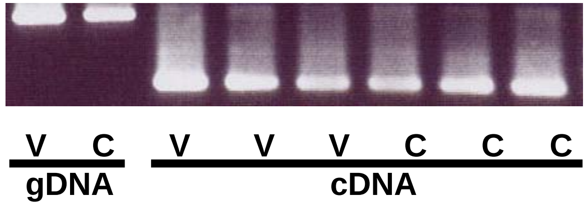
V C V V V C C C
gDNA
cDNA

√

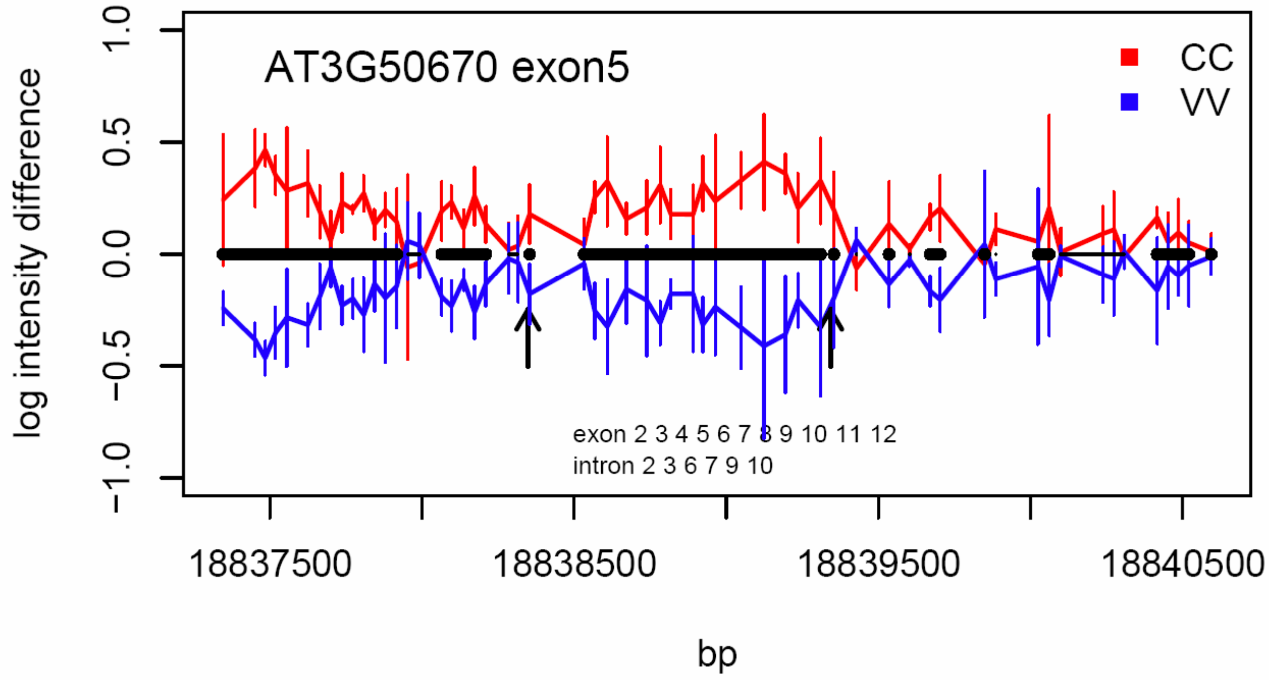

AT3G50670 exon5
CC
VV
log intensity difference
1.0
0.5
0.0
−0.5
−1.0
exon 2 3 4 5 6 7 8 9 10 11 12
intron 2 3 6 7 9 10
18837500
18838500
18839500
18840500
bp

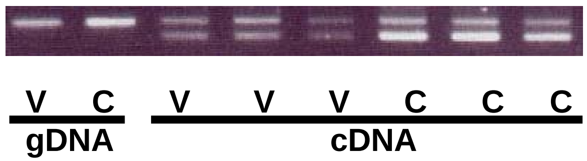

V C V V V C C C
gDNA
cDNA

√

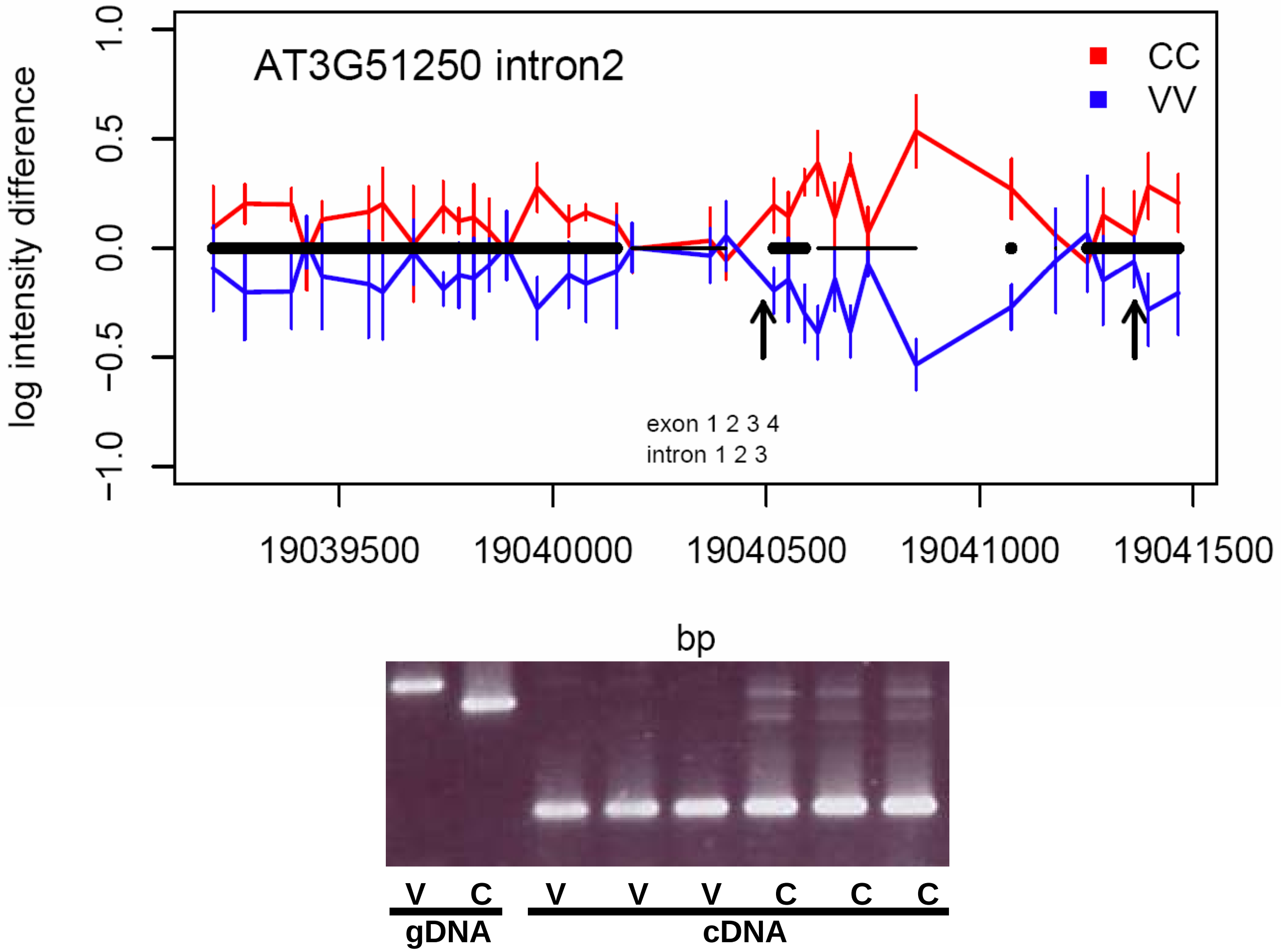

AT3G51250 intron2
CC
VV
log intensity difference
1.0
0.5
0.0
−0.5
−1.0
exon 1 2 3 4
intron 1 2 3
19039500
19040000
19040500
19041000
19041500
bp
V C V V V C C C
gDNA
cDNA

Not included for analysis

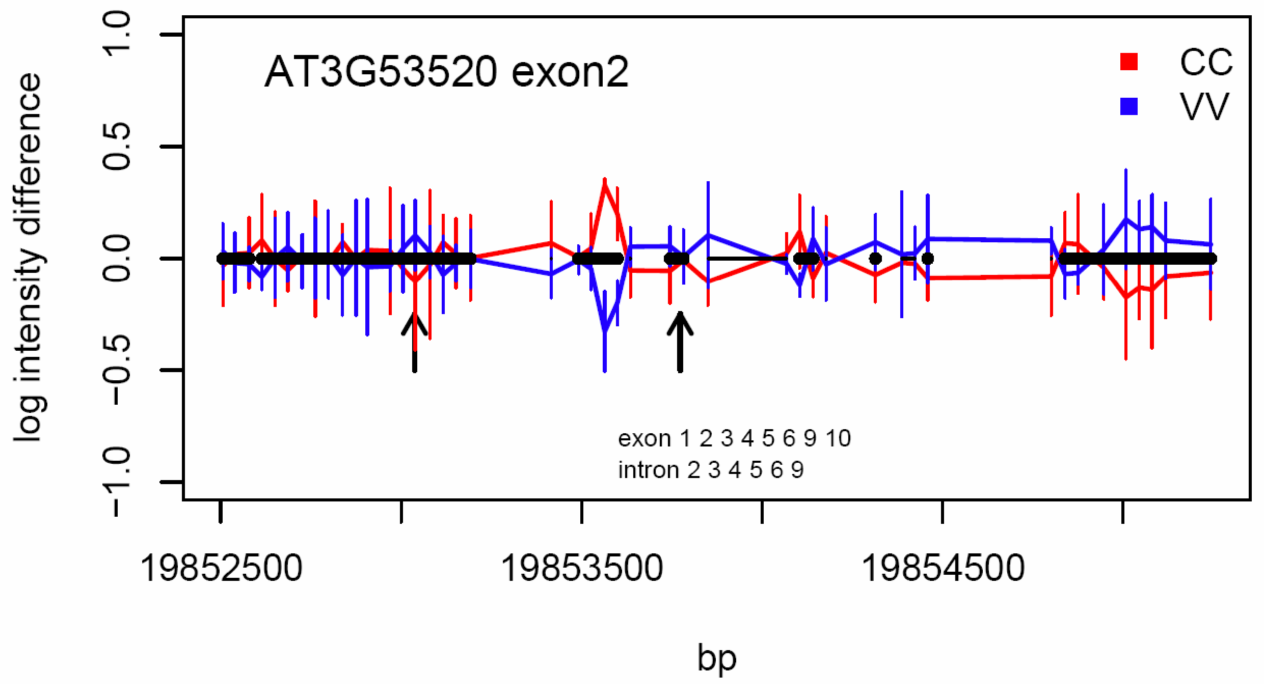

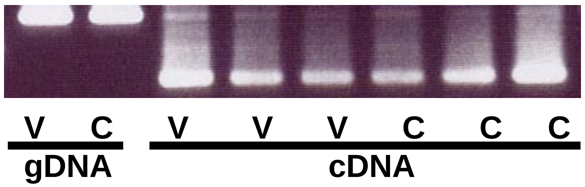

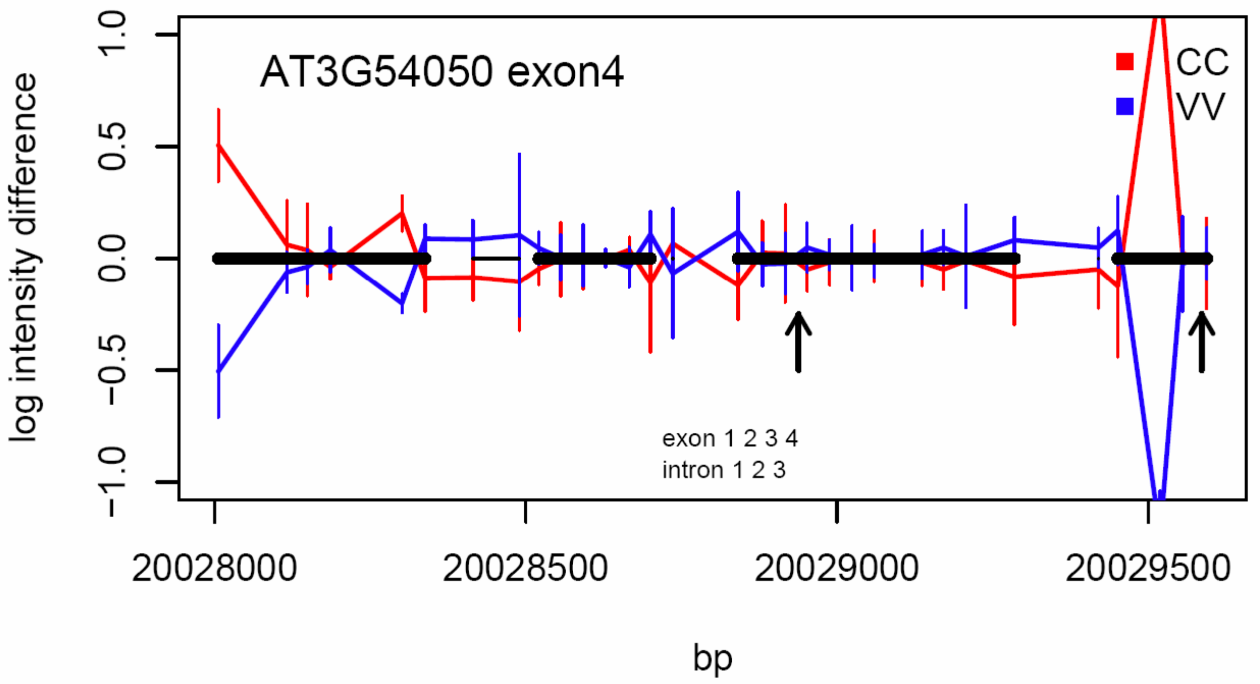
AT3G54050 exon4
CC
VV
log intensity difference
1.0
0.5
0.0
−0.5
−1.0
exon 1 2 3 4
intron 1 2 3
20028000
20028500
20029000
20029500
bp

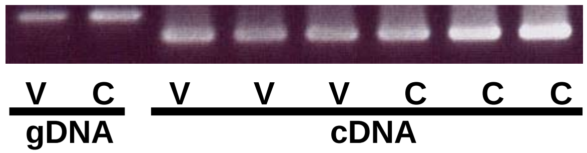
V C V V V C C C
gDNA
cDNA

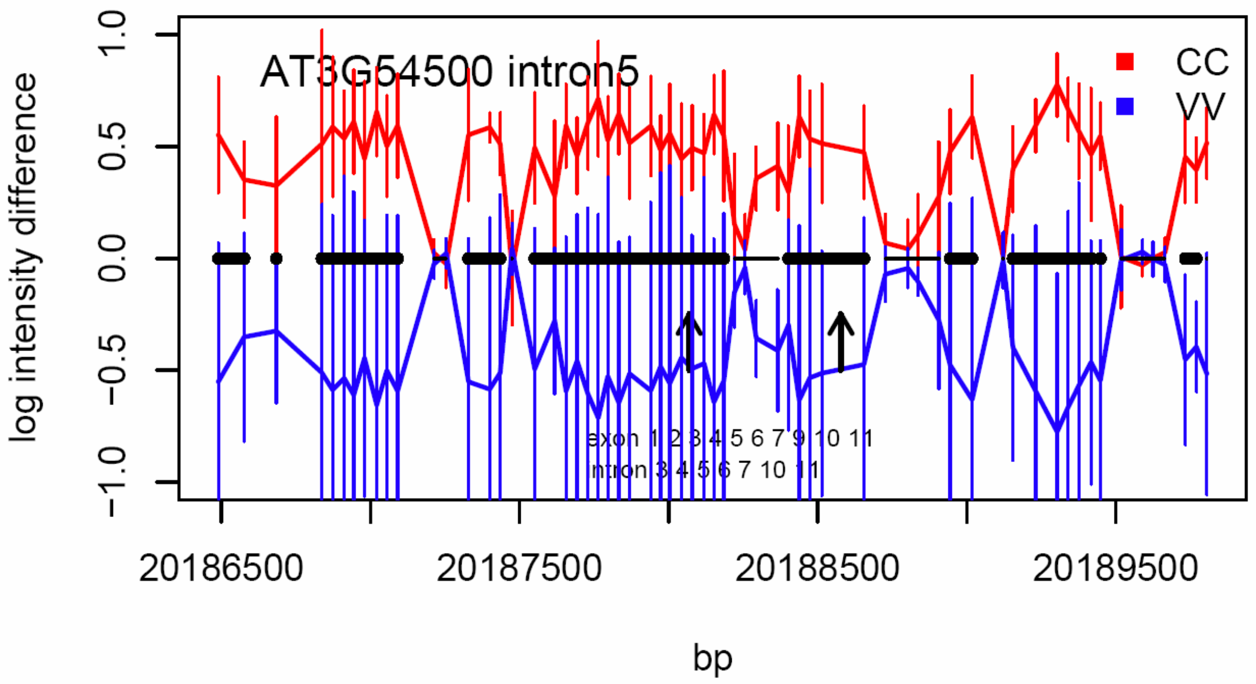
AT3G54500 intron5
CC
VV
log intensity difference
1.0
0.5
0.0
−0.5
−1.0
exon 1 2 3 4 5 6 7 9 10 11
intron 3 4 5 6 7 10 11
20186500
20187500
20188500
20189500
bp

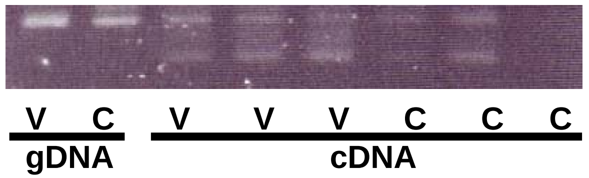
V C V V V C C C
gDNA
cDNA

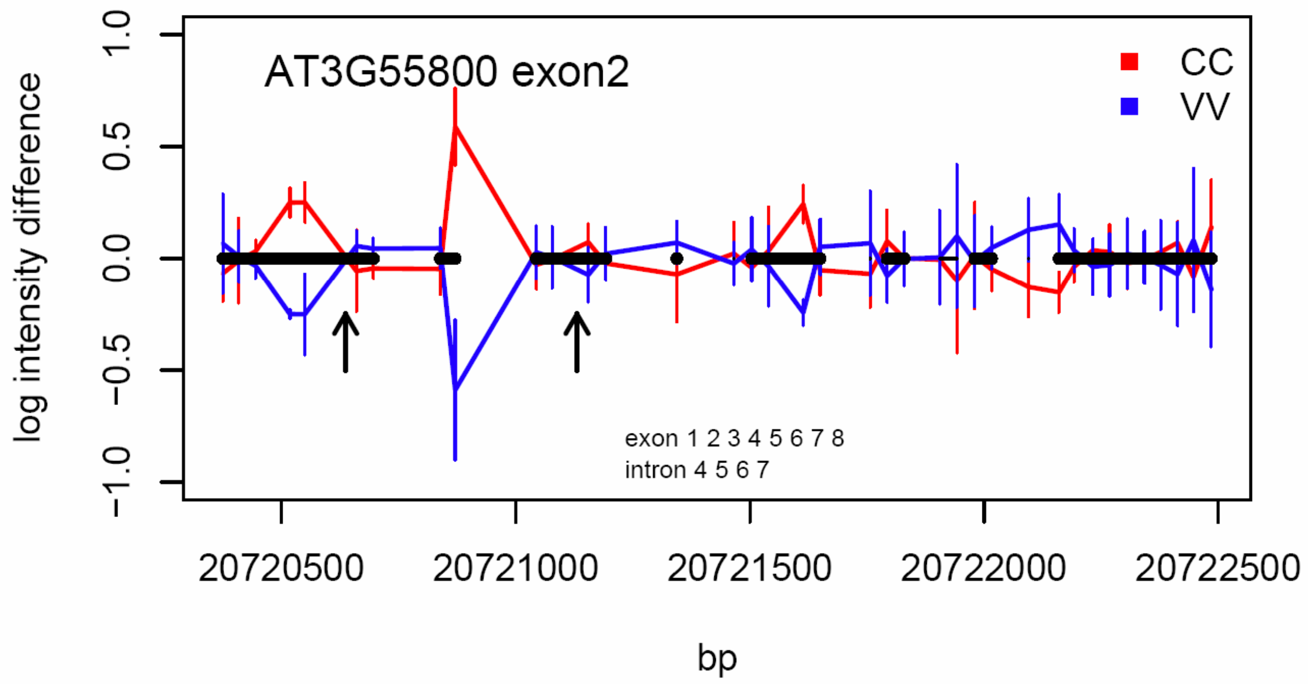
AT3G55800 exon2
CC
VV
log intensity difference
1.0
0.5
0.0
-0.5
-1.0
exon 1 2 3 4 5 6 7 8
intron 4 5 6 7
20720500
20721000
20721500
20722000
20722500
bp

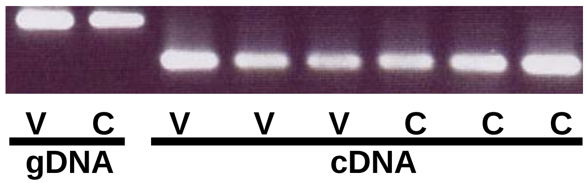
V C V V V C C C
gDNA
cDNA

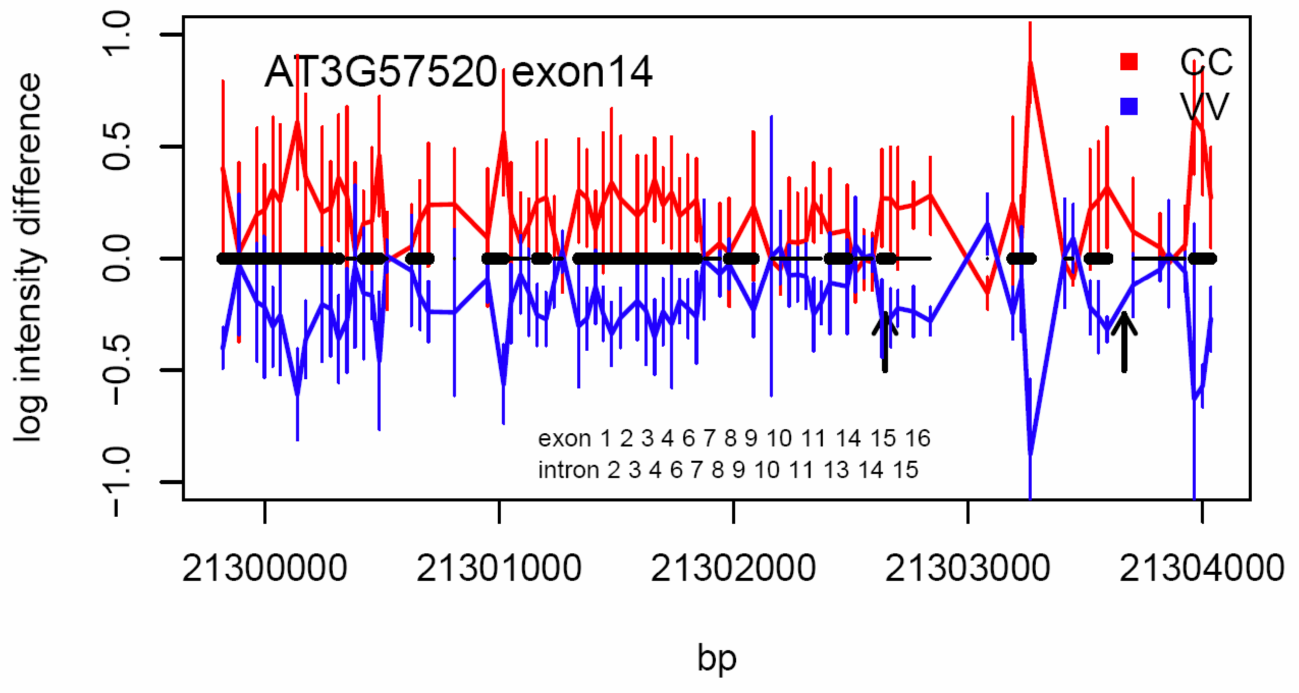
AT3G57520 exon14
CC
VV
log intensity difference
1.0
0.5
0.0
-0.5
-1.0
exon 1 2 3 4 6 7 8 9 10 11 14 15 16
intron 2 3 4 6 7 8 9 10 11 13 14 15
21300000
21301000
21302000
21303000
21304000
bp

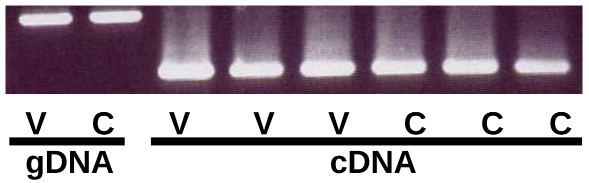
V C V V V C C C
gDNA
cDNA

√

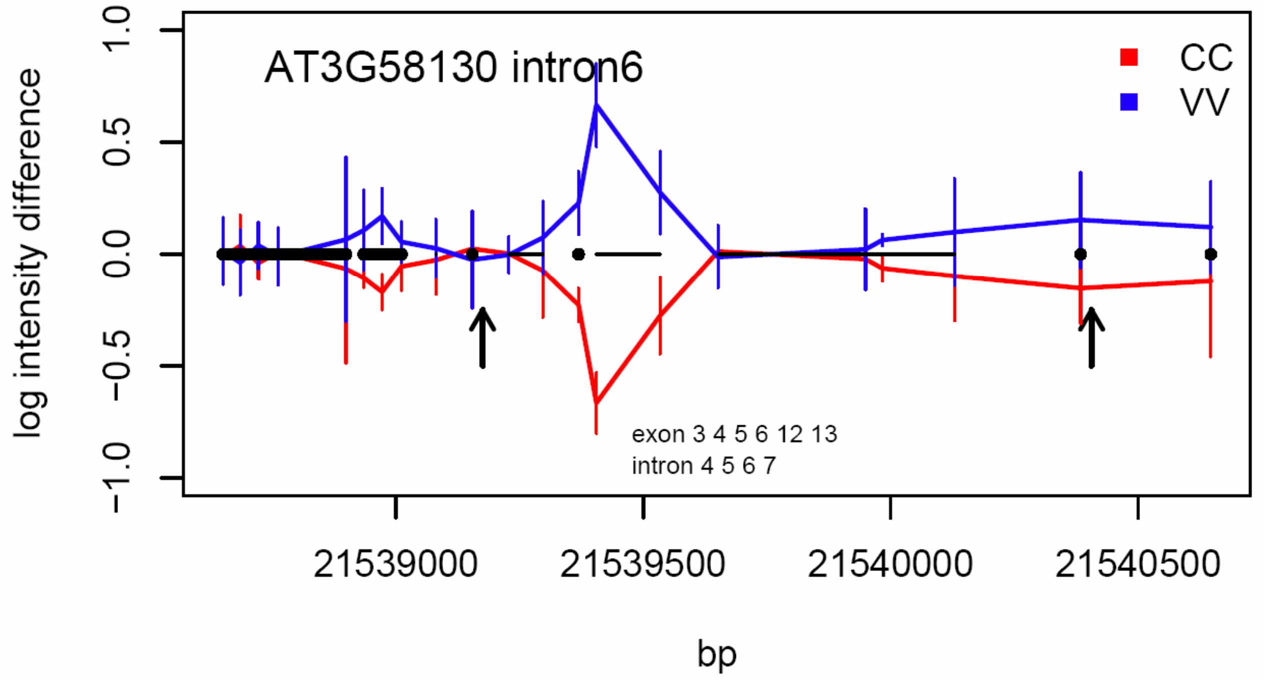
AT3G58130 intron6
CC
VV
log intensity difference
1.0
0.5
0.0
−0.5
−1.0
exon 3 4 5 6 12 13
intron 4 5 6 7
21539000
21539500
21540000
21540500
bp

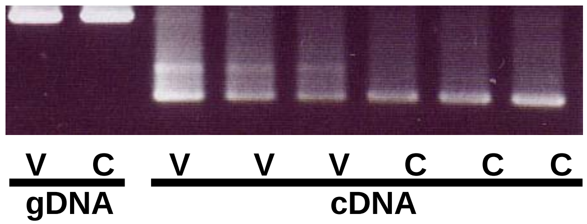
V C V V V C C C
gDNA
cDNA

Not included for analysis

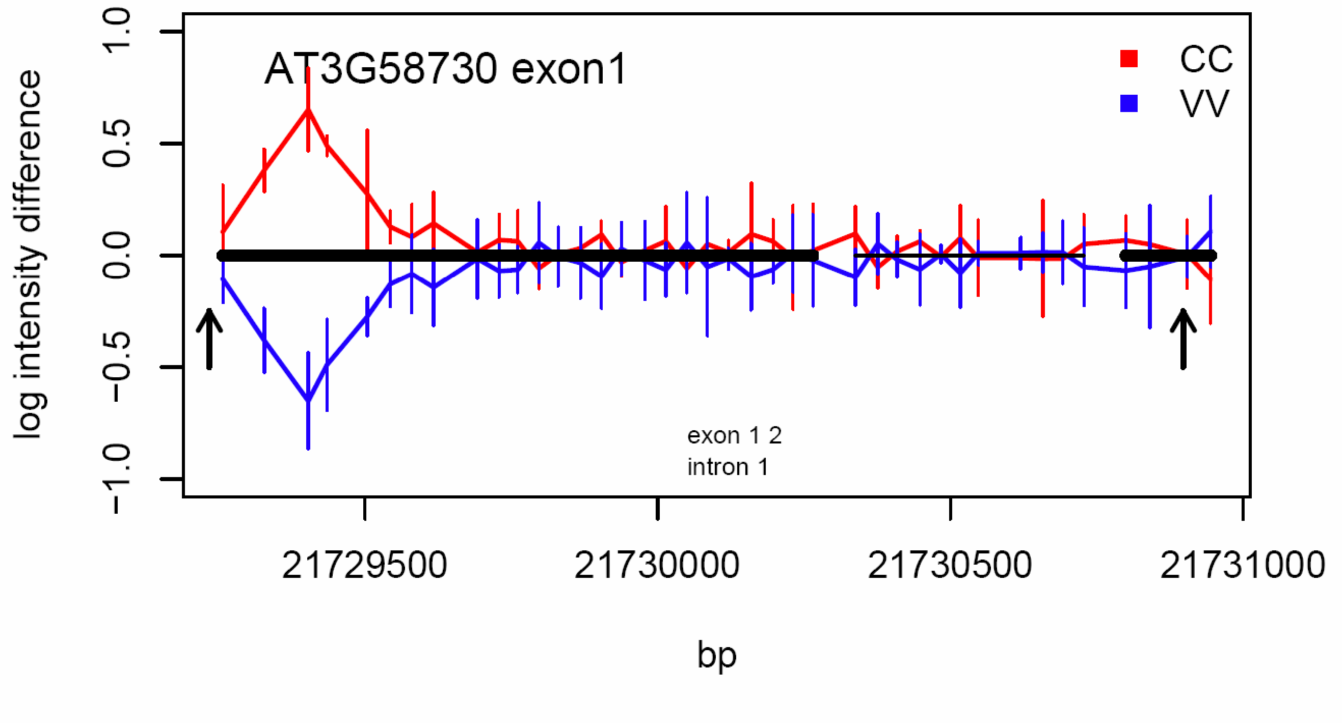

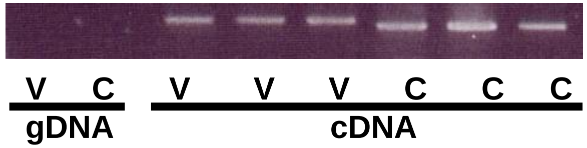

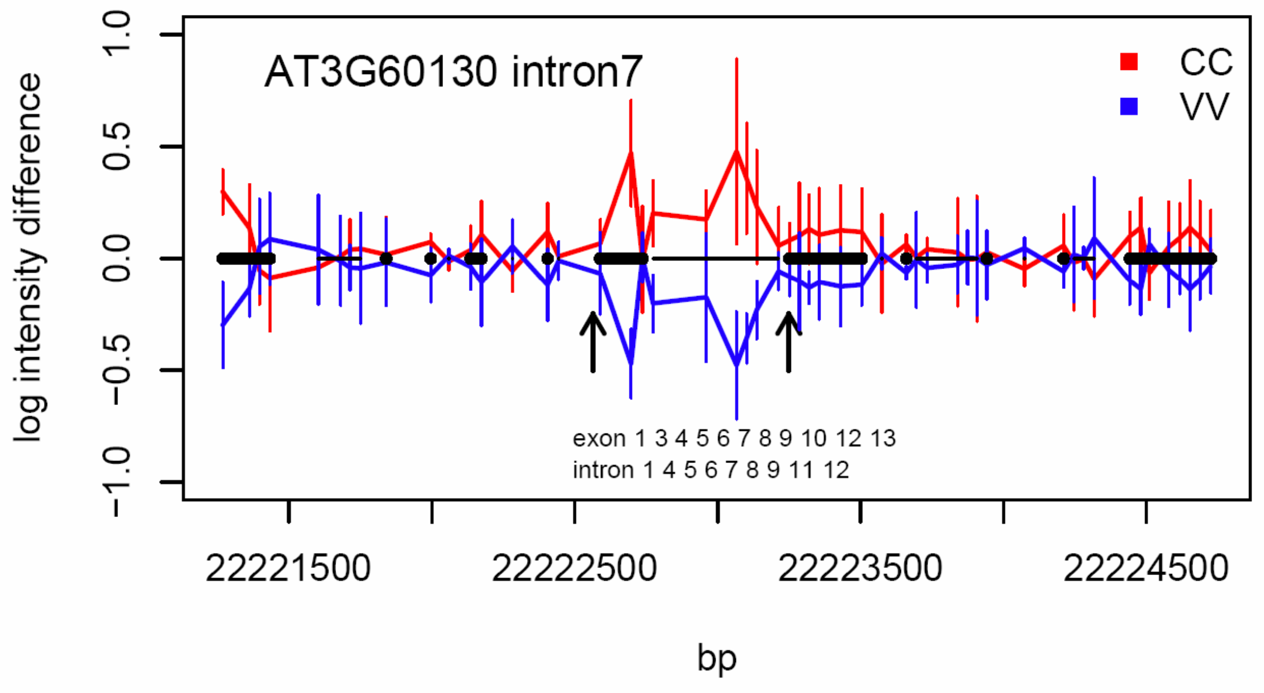
AT3G60130 intron7
CC
VV
log intensity difference
1.0
0.5
0.0
−0.5
−1.0
exon 1 3 4 5 6 7 8 9 10 12 13
intron 1 4 5 6 7 8 9 11 12
22221500
22222500
22223500
22224500
bp

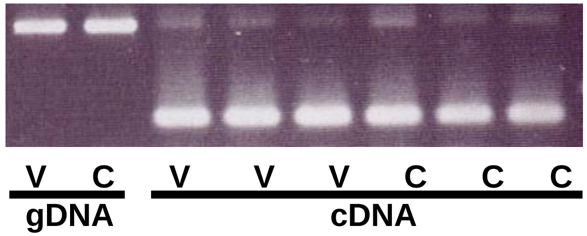
V C V V V C C C
gDNA
cDNA

√

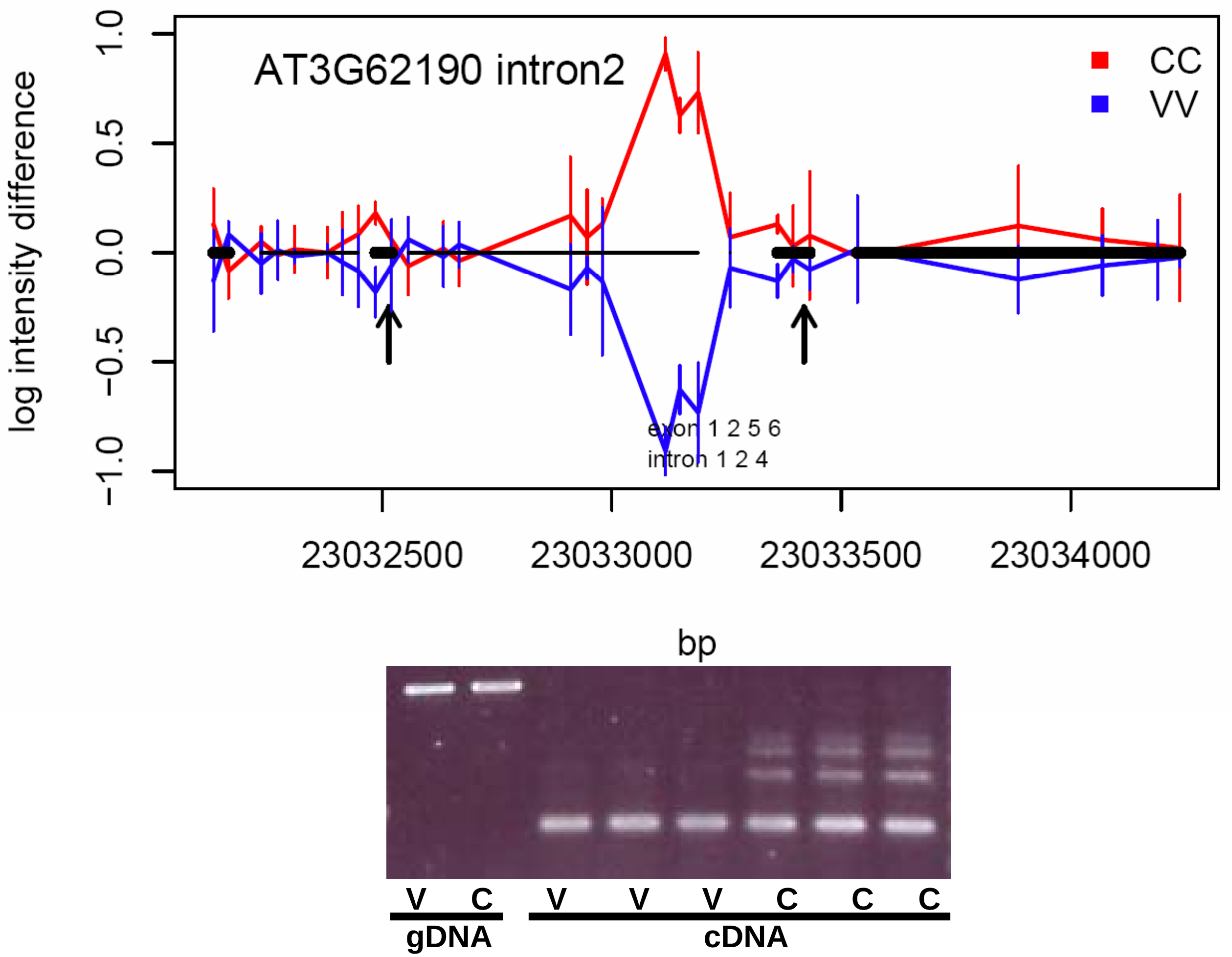

AT3G62190 intron2
CC
VV
log intensity difference
1.0
0.5
0.0
−0.5
−1.0
exon 1 2 5 6
intron 1 2 4
23032500
23033000
23033500
23034000
bp
V C V V V C C C
gDNA
cDNA

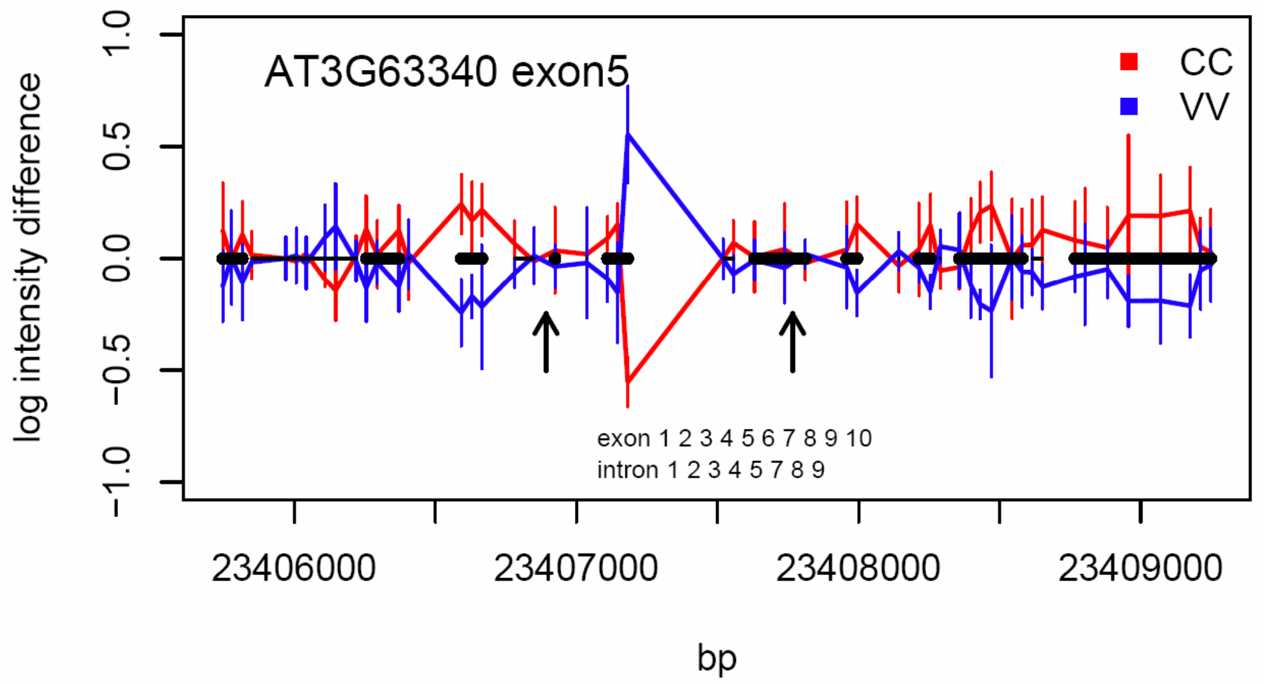

AT3G63340 exon5
CC
VV
log intensity difference
1.0
0.5
0.0
−0.5
−1.0
exon 1 2 3 4 5 6 7 8 9 10
intron 1 2 3 4 5 7 8 9
23406000
23407000
23408000
23409000
bp

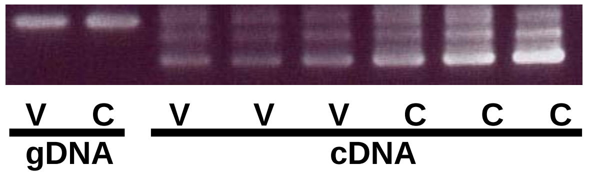

V C V V V C C C
gDNA
cDNA

Not included for analysis

√

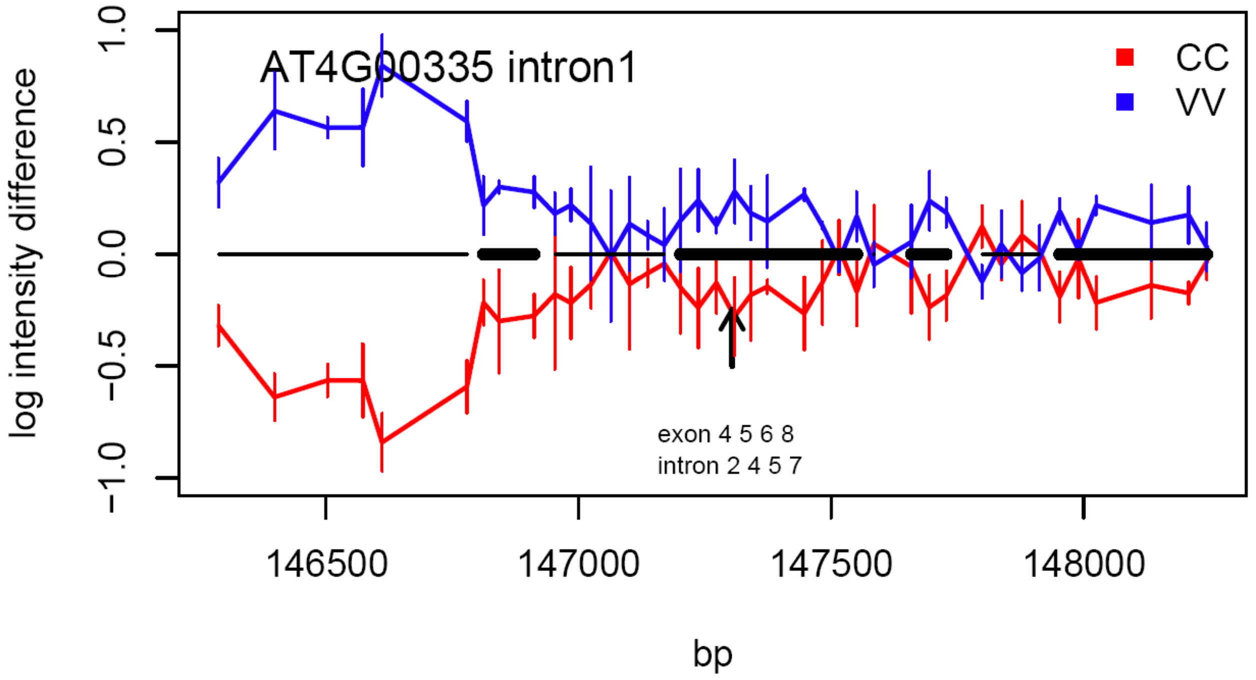

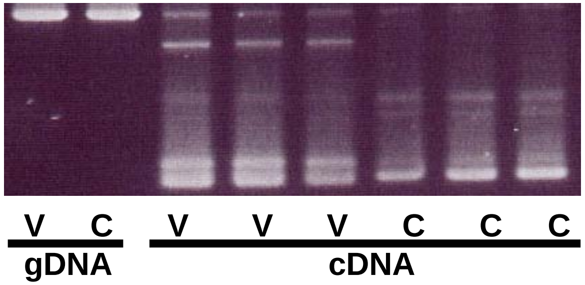

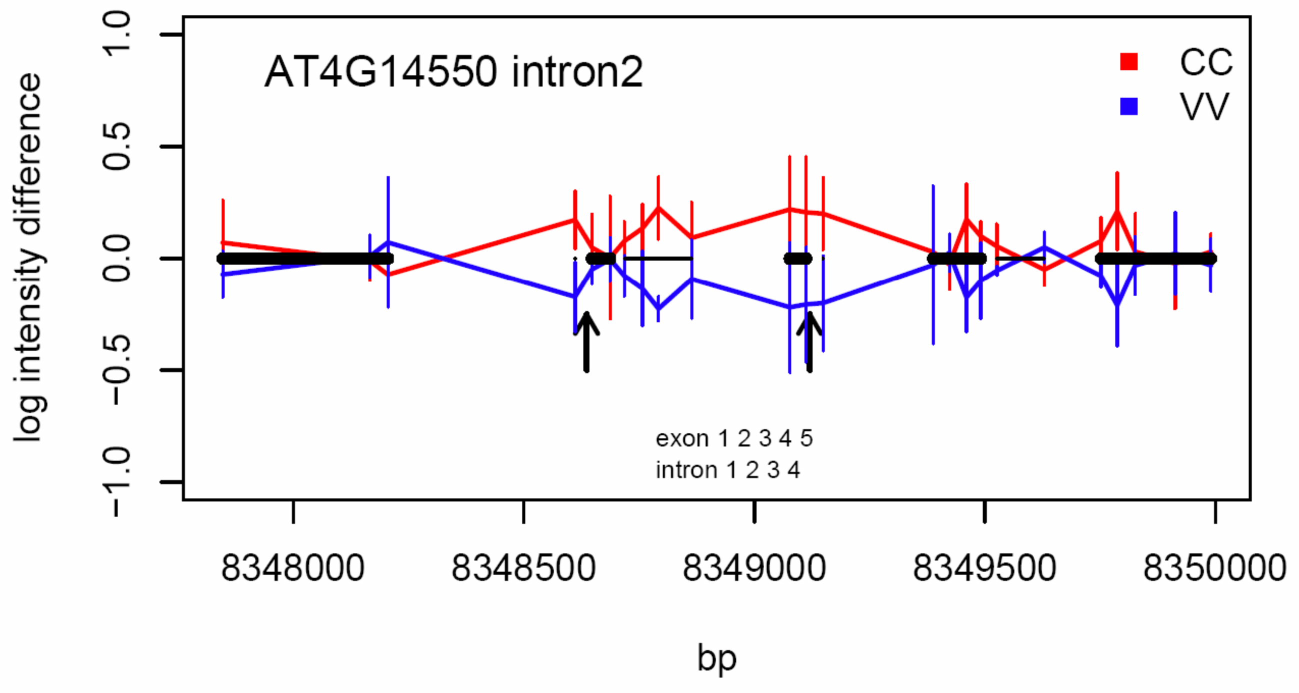
AT4G14550 intron2
CC
VV
log intensity difference
1.0
0.5
0.0
−0.5
−1.0
exon 1 2 3 4 5
intron 1 2 3 4
8348000
8348500
8349000
8349500
8350000
bp

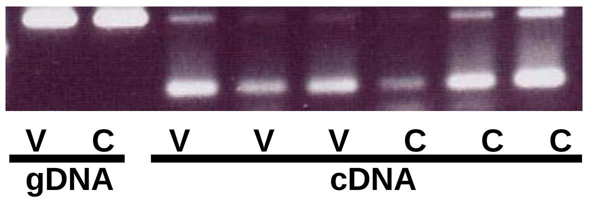
V C V V V C C C
gDNA
cDNA

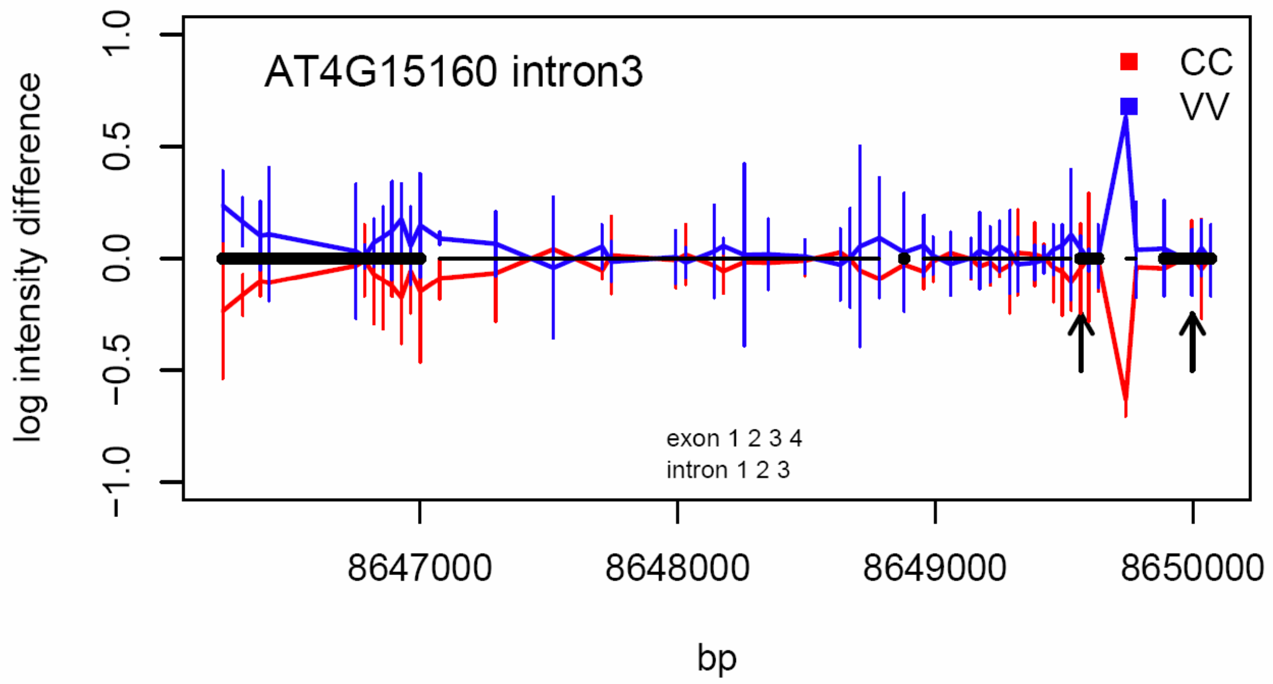
AT4G15160 intron3
CC
VV
log intensity difference
1.0
0.5
0.0
−0.5
−1.0
exon 1 2 3 4
intron 1 2 3
8647000
8648000
8649000
8650000
bp

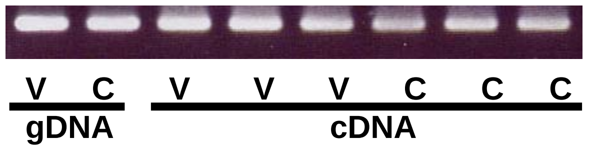
V C V V V C C C
gDNA
cDNA

√

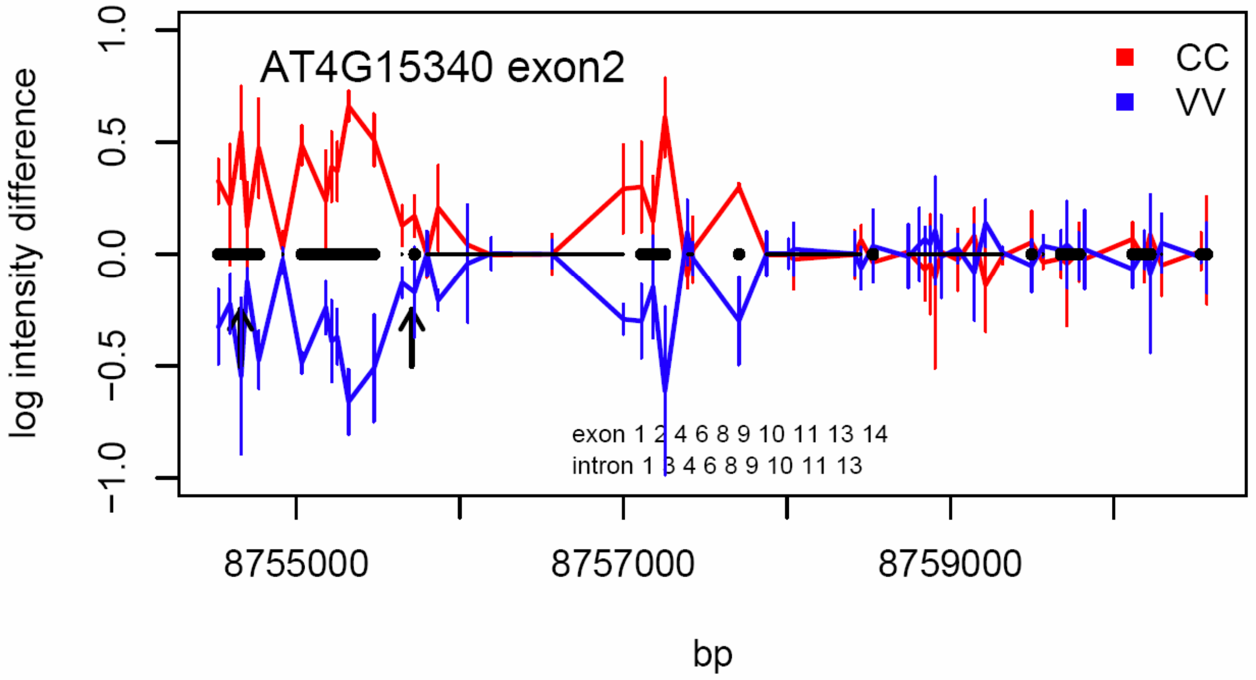
AT4G15340 exon2
CC
VV
log intensity difference
1.0
0.5
0.0
−0.5
−1.0
exon 1 2 4 6 8 9 10 11 13 14
intron 1 3 4 6 8 9 10 11 13
8755000
8757000
8759000
bp

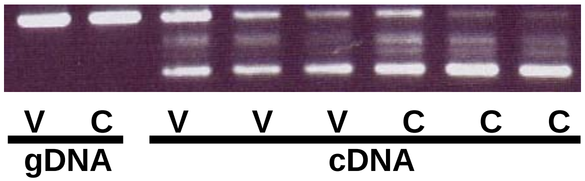
V C V V V C C C
gDNA
cDNA

Not included for analysis

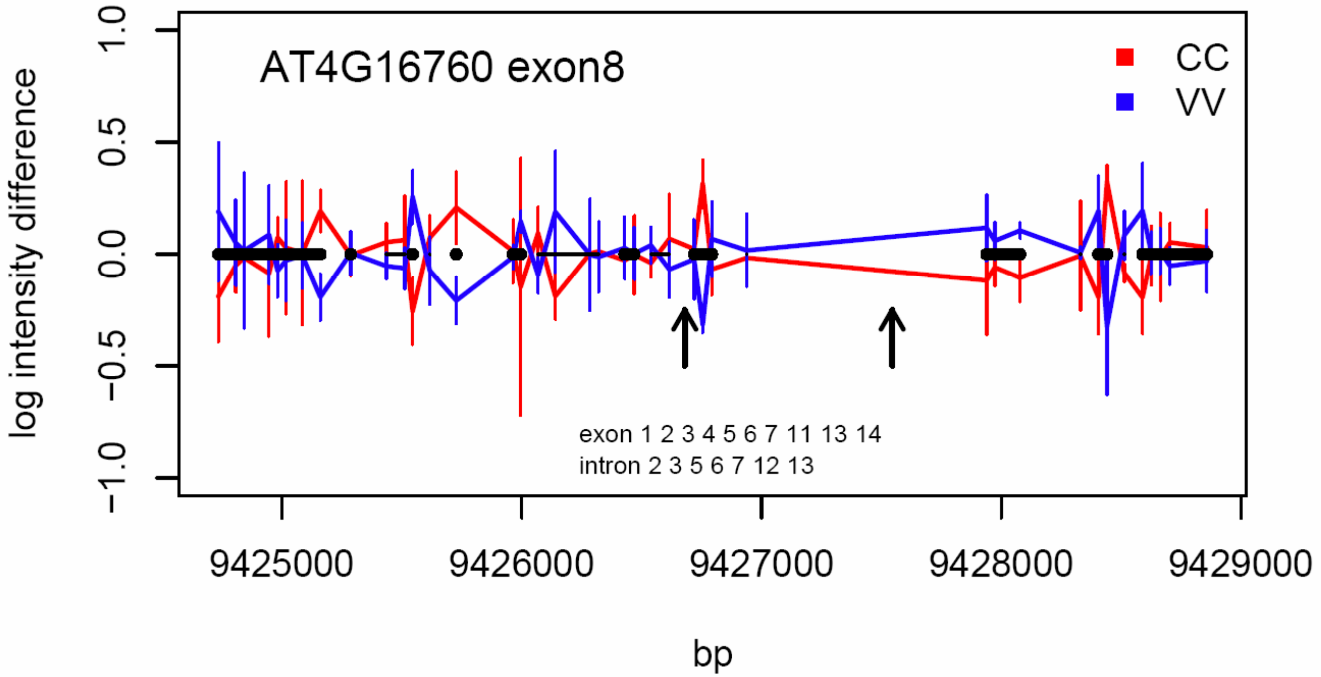

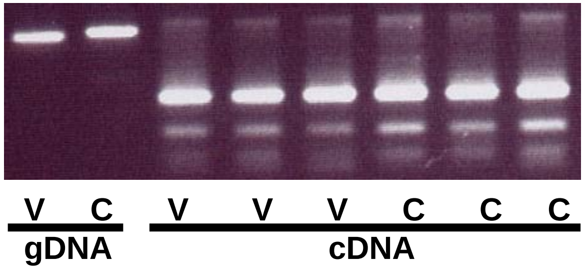

√

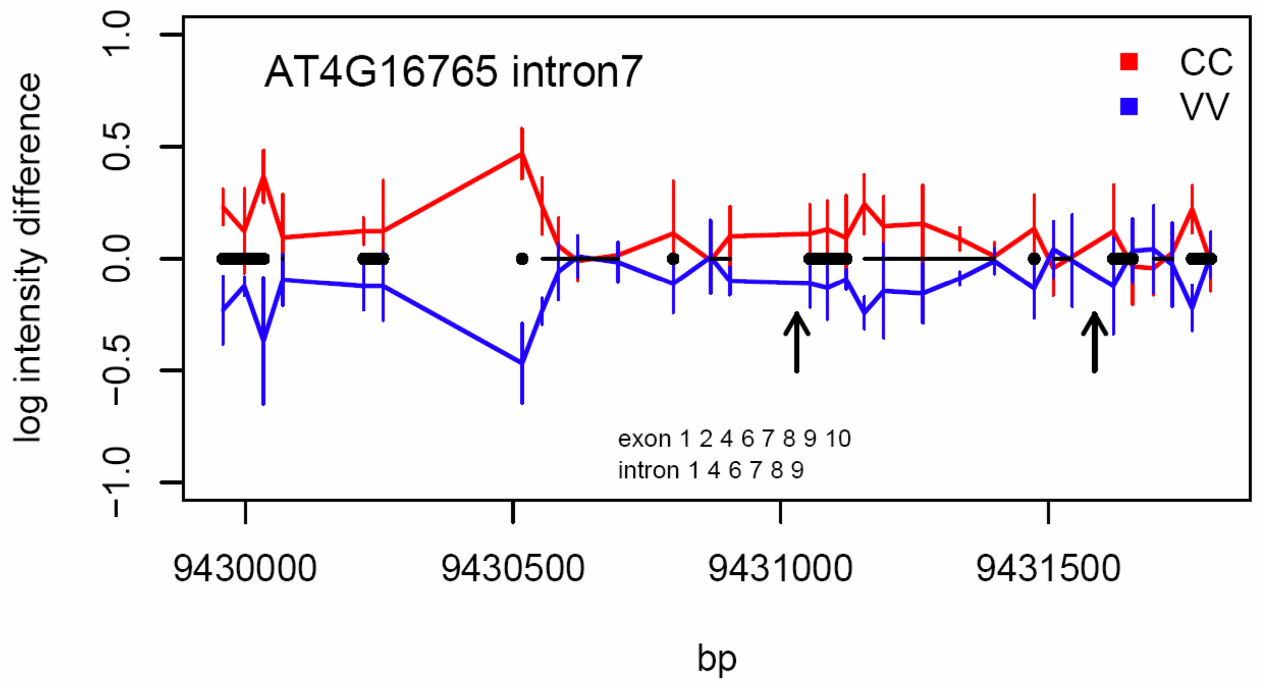
AT4G16765 intron7
CC
VV
log intensity difference
1.0
0.5
0.0
−0.5
−1.0
exon 1 2 4 6 7 8 9 10
intron 1 4 6 7 8 9
9430000
9430500
9431000
9431500
bp

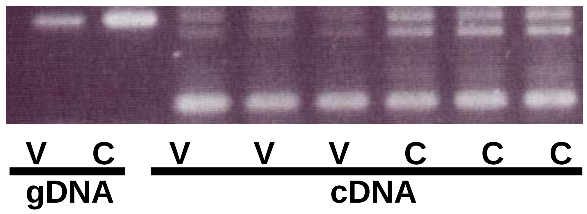
V C V V V C C C
gDNA
cDNA

√

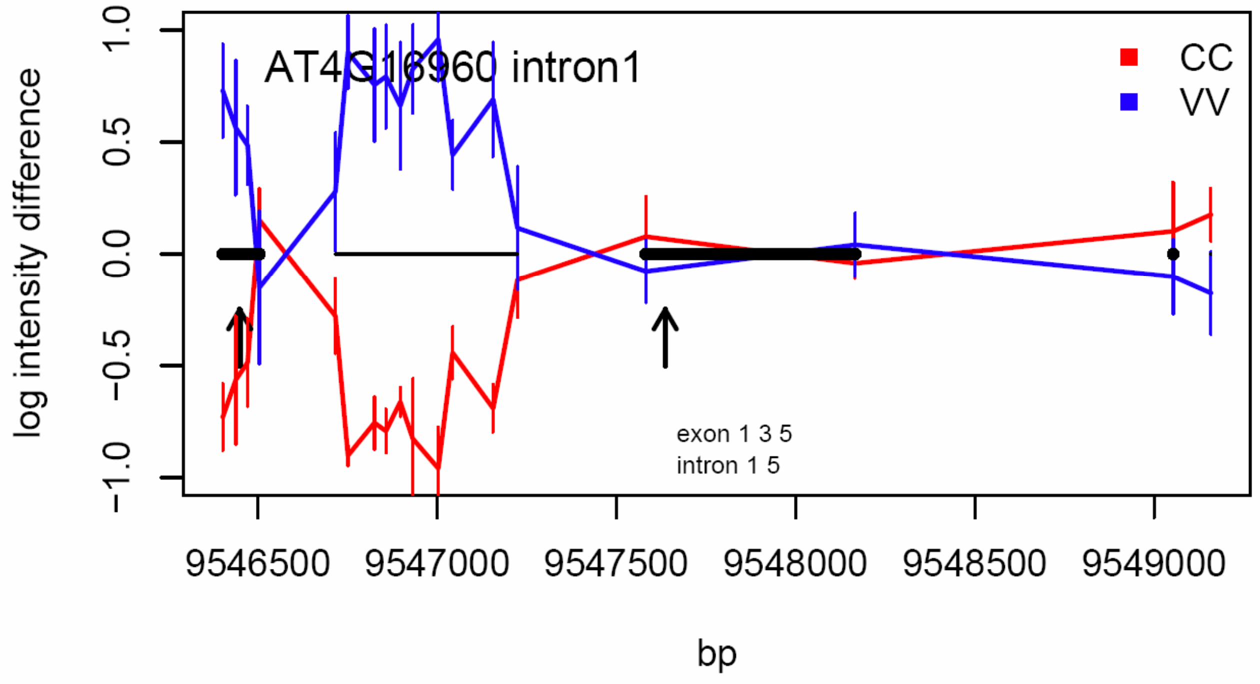
AT4G16960 intron1
CC
VV
log intensity difference
1.0
0.5
0.0
−0.5
−1.0
exon 1 3 5
intron 1 5
9546500
9547000
9547500
9548000
9548500
9549000
bp

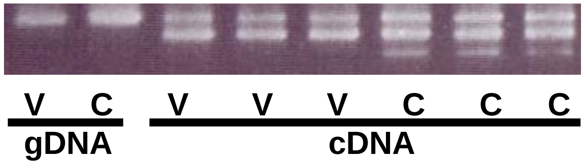
V C V V V C C C
gDNA
cDNA

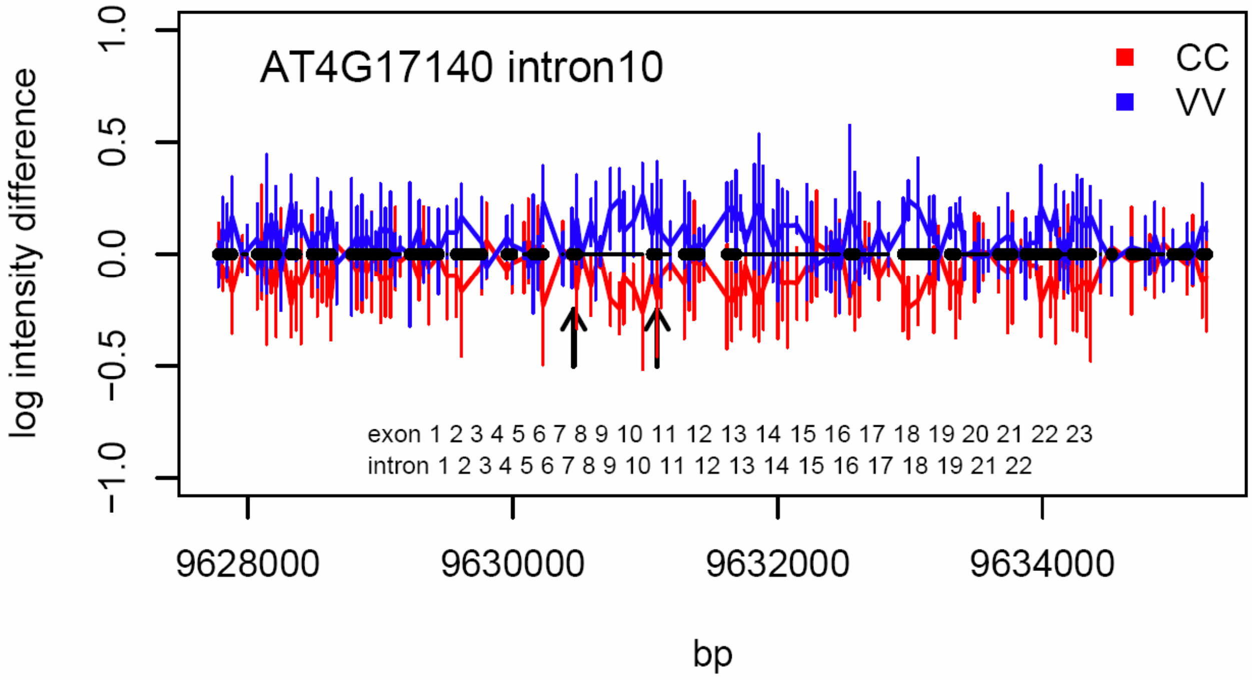
AT4G17140 intron10
CC
VV
log intensity difference
1.0
0.5
0.0
−0.5
−1.0
exon 1 2 3 4 5 6 7 8 9 10 11 12 13 14 15 16 17 18 19 20 21 22 23
intron 1 2 3 4 5 6 7 8 9 10 11 12 13 14 15 16 17 18 19 21 22
9628000
9630000
9632000
9634000
bp

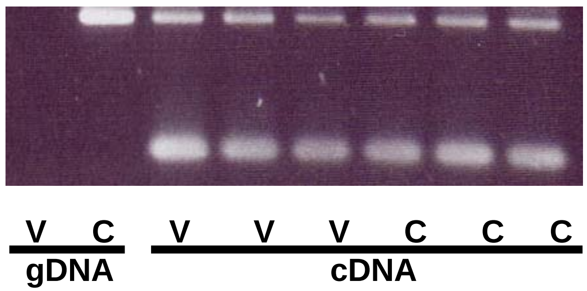
V C V V V C C C
gDNA
cDNA

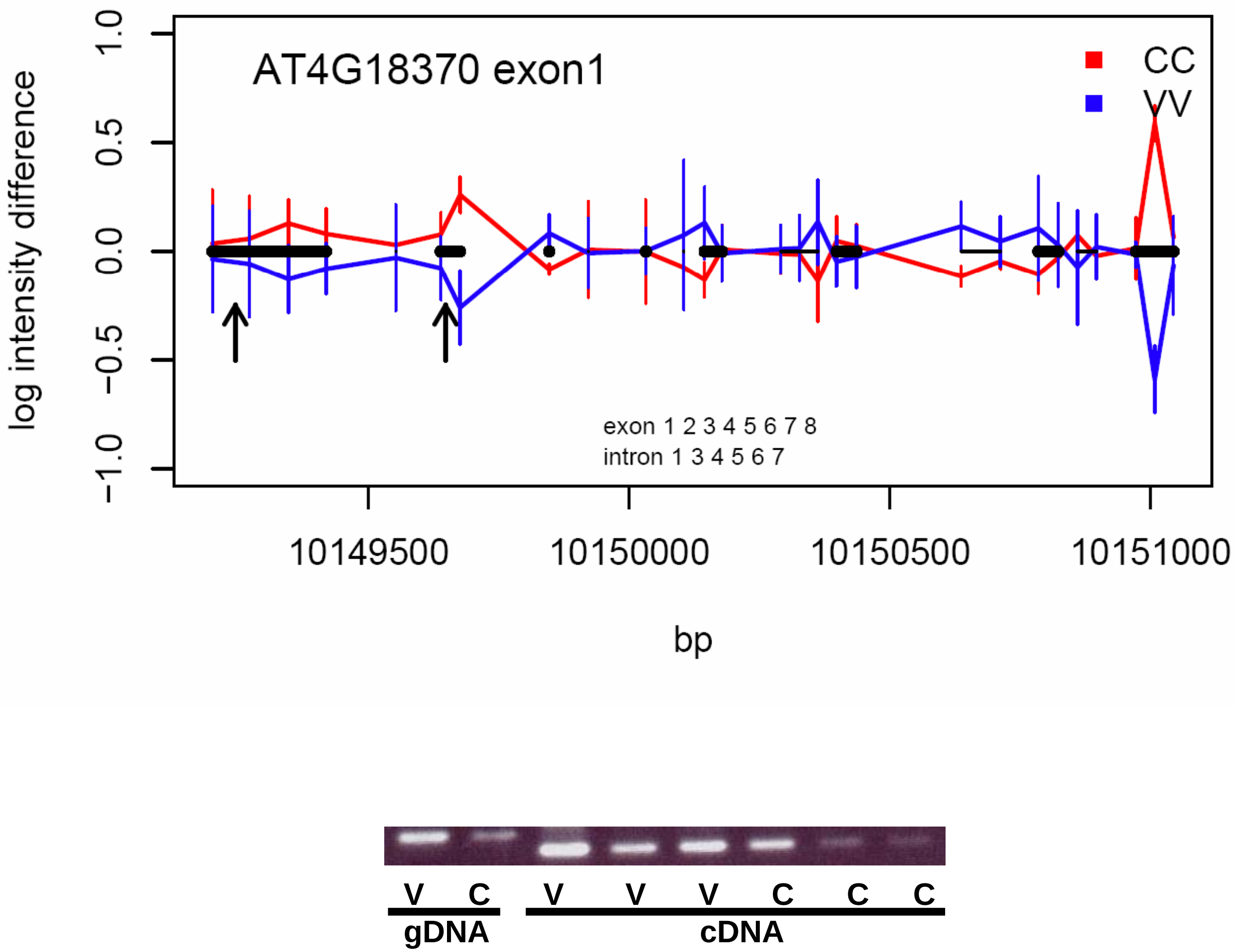
AT4G18370 exon1
CC
VV
log intensity difference
1.0
0.5
0.0
-0.5
-1.0
exon 1 2 3 4 5 6 7 8
intron 1 3 4 5 6 7
10149500
10150000
10150500
10151000
bp
V C V V V C C C
gDNA
cDNA

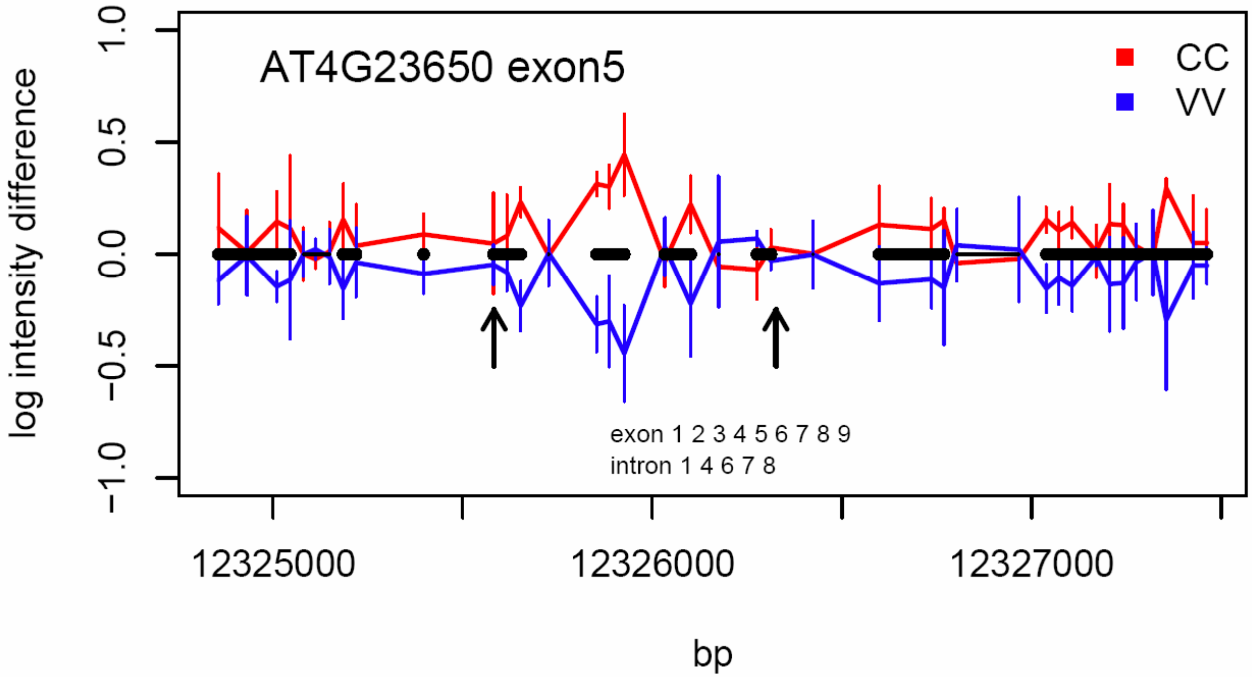

AT4G23650 exon5
CC
VV
log intensity difference
1.0
0.5
0.0
−0.5
−1.0
exon 1 2 3 4 5 6 7 8 9
intron 1 4 6 7 8
12325000
12326000
12327000
bp

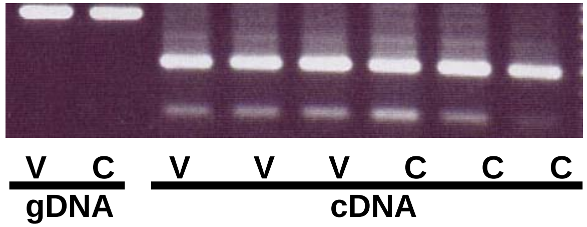

V C V V V C C C
gDNA
cDNA

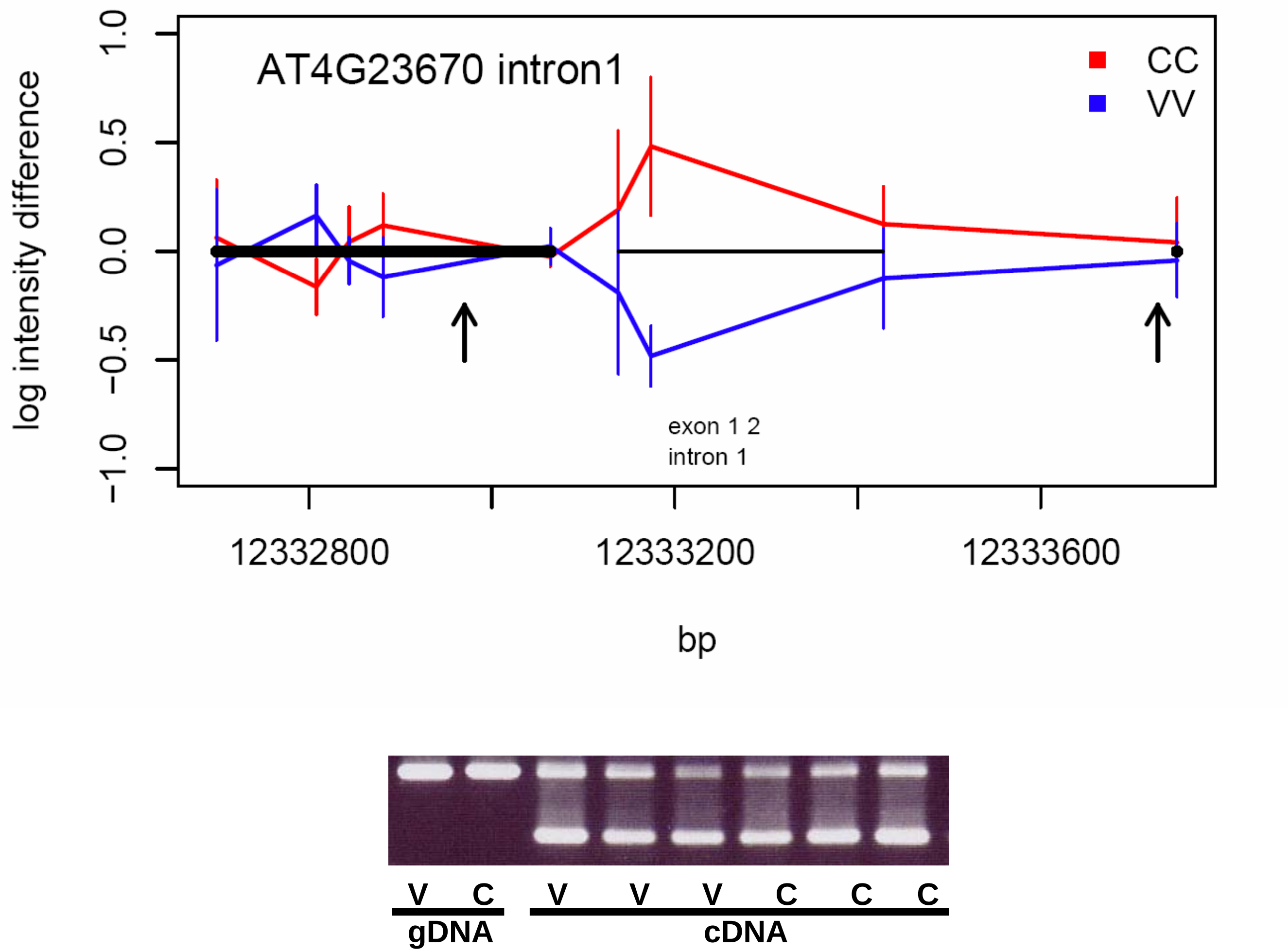
AT4G23670 intron1
CC
VV
log intensity difference
1.0
0.5
0.0
−0.5
−1.0
exon 1 2
intron 1
12332800
12333200
12333600
bp
V C
gDNA
V V V C C C
cDNA

√

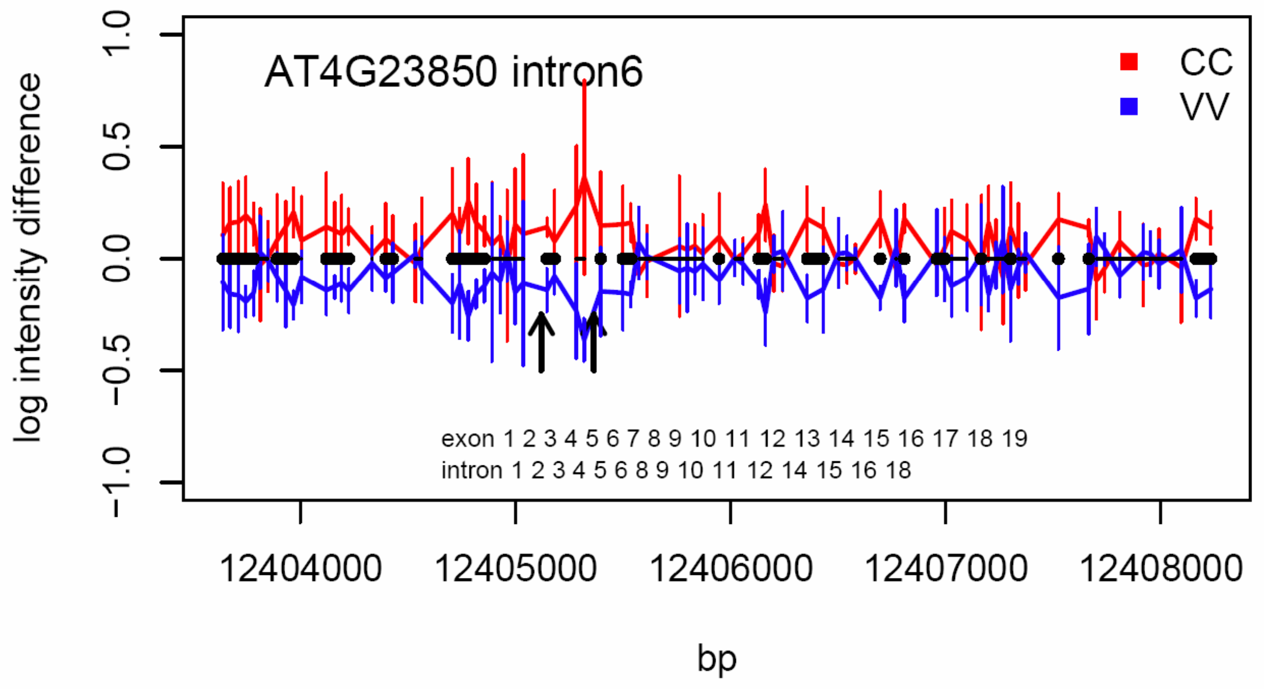
AT4G23850 intron6
CC
VV
log intensity difference
1.0
0.5
0.0
−0.5
−1.0
exon 1 2 3 4 5 6 7 8 9 10 11 12 13 14 15 16 17 18 19
intron 1 2 3 4 5 6 8 9 10 11 12 14 15 16 18
12404000
12405000
12406000
12407000
12408000
bp

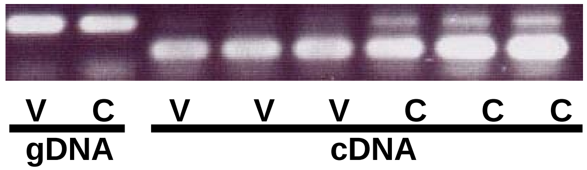
V C V V V C C C
gDNA
cDNA

√

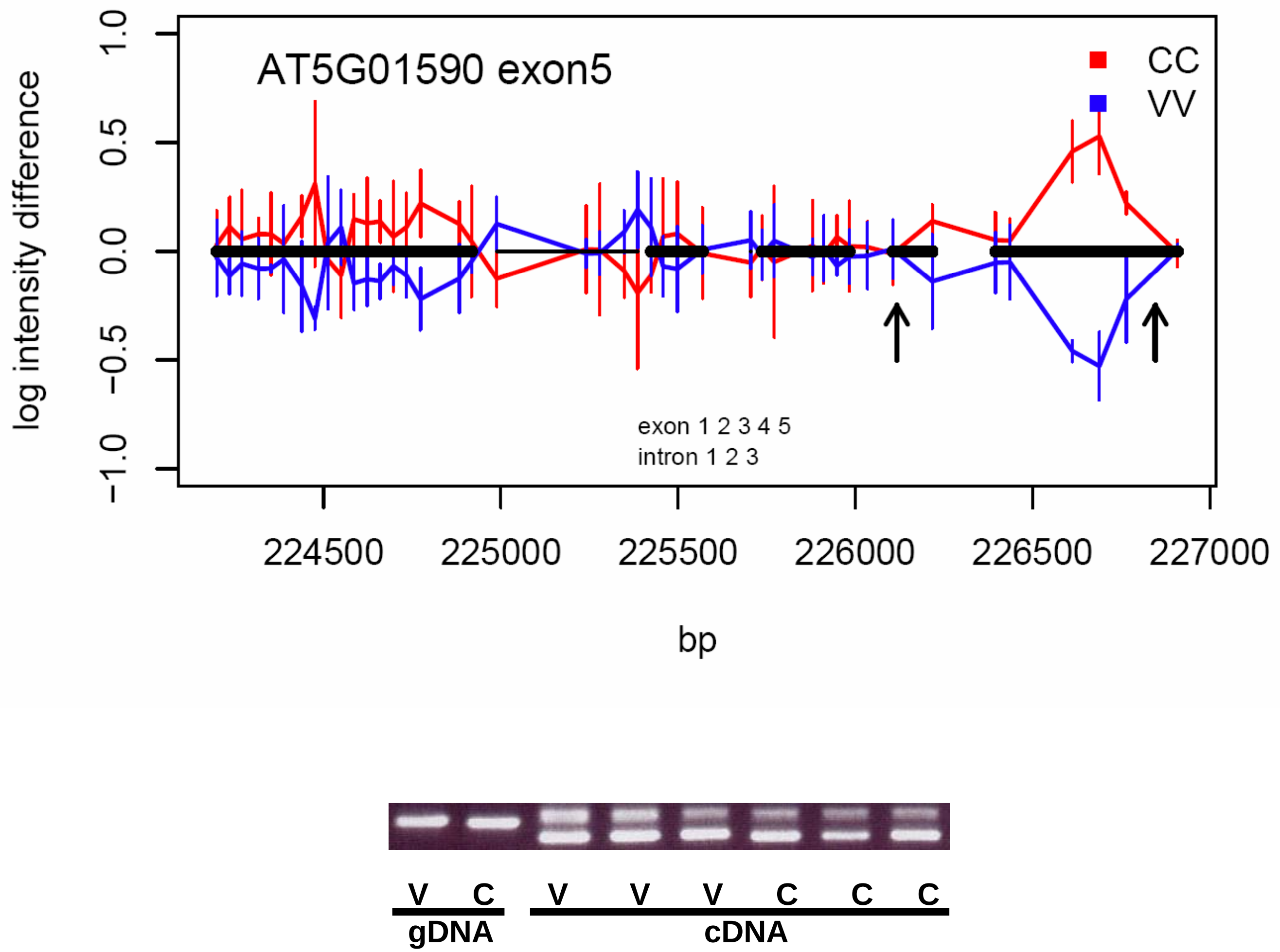
AT5G01590 exon5
CC
VV
log intensity difference
1.0
0.5
0.0
−0.5
−1.0
exon 1 2 3 4 5
intron 1 2 3
224500
225000
225500
226000
226500
227000
bp
V
C
V
V
V
C
C
C
gDNA
cDNA

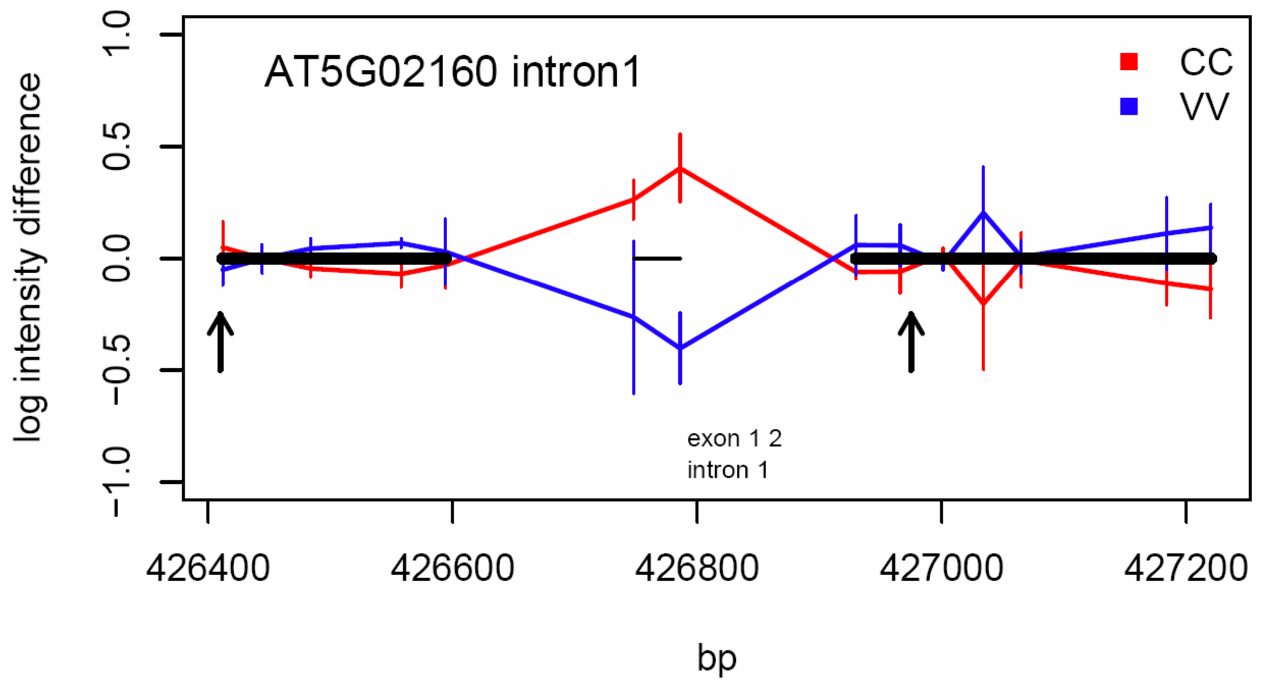
AT5G02160 intron1
CC
VV
log intensity difference
1.0
0.5
0.0
−0.5
−1.0
exon 1 2
intron 1
426400
426600
426800
427000
427200
bp

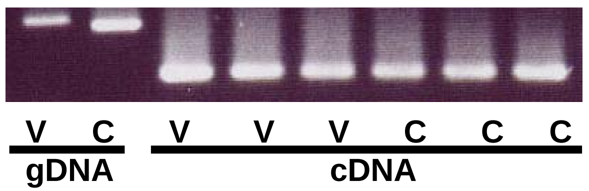
V C V V V C C C
gDNA
cDNA

√

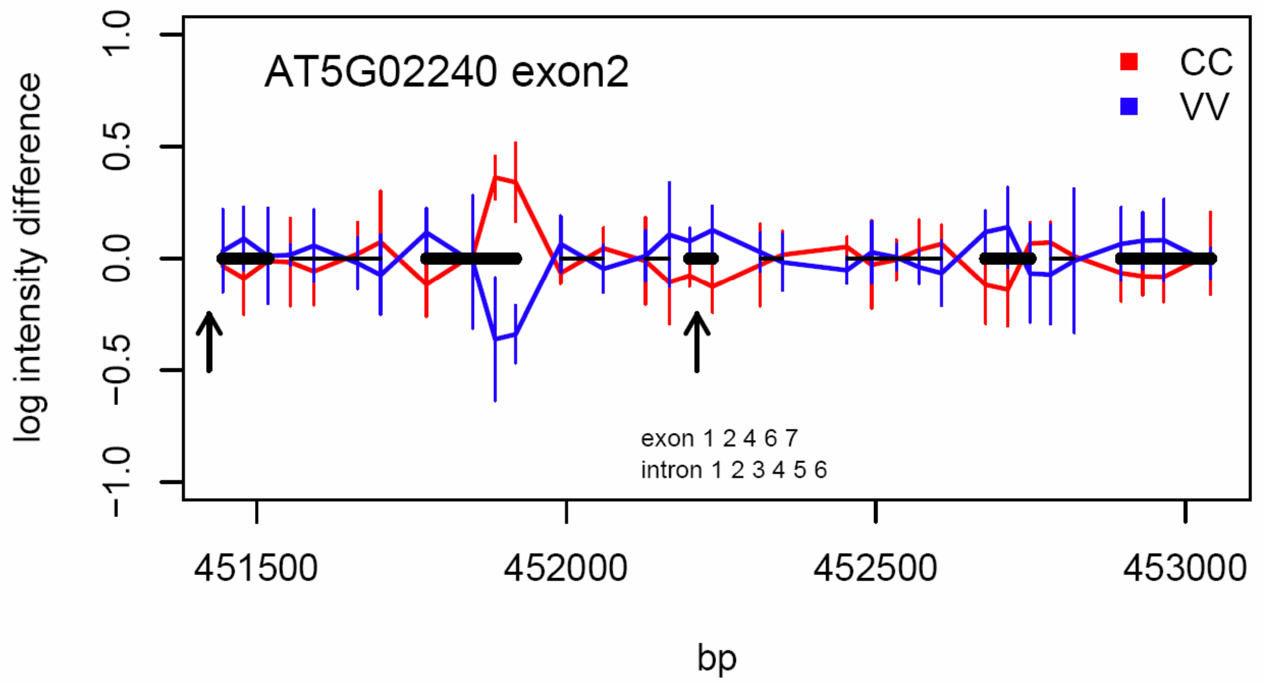

AT5G02240 exon2
CC
VV
log intensity difference
1.0
0.5
0.0
−0.5
−1.0
exon 1 2 4 6 7
intron 1 2 3 4 5 6
451500
452000
452500
453000
bp

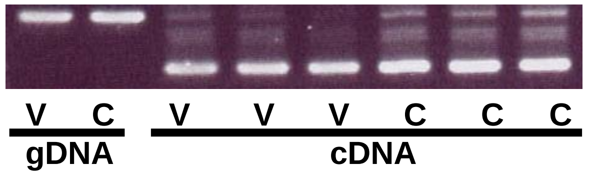

V C V V V C C C
gDNA
cDNA

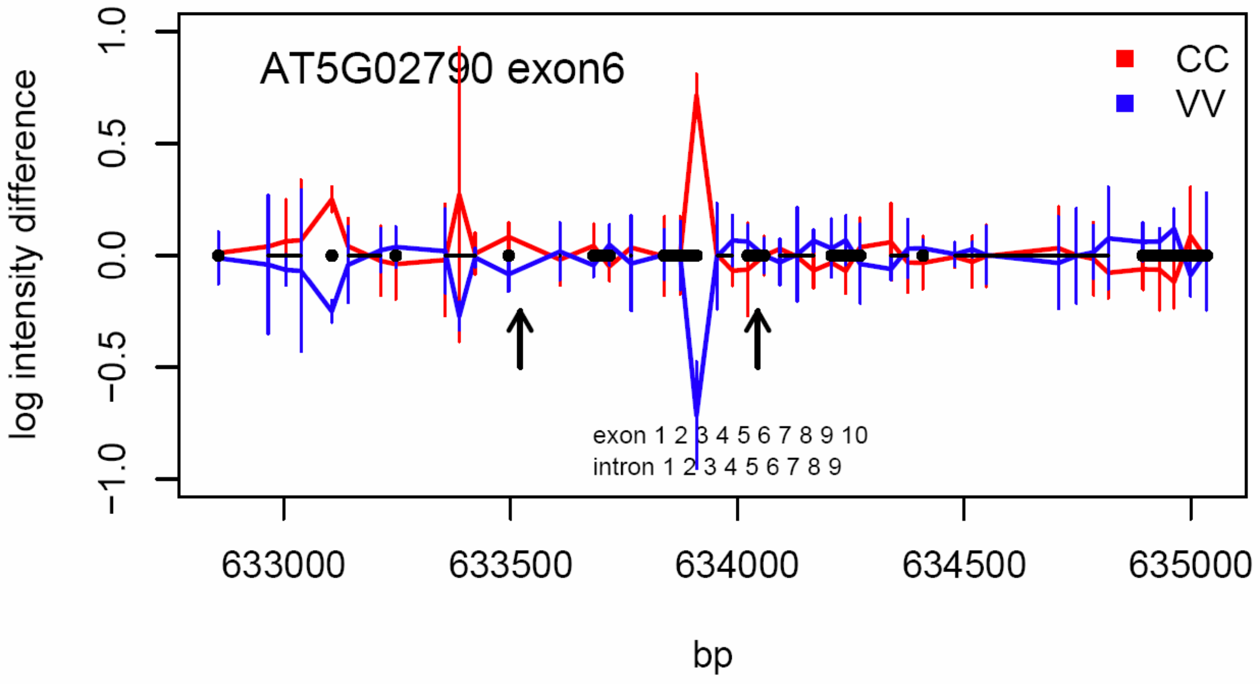
AT5G02790 exon6
CC
VV
log intensity difference
1.0
0.5
0.0
−0.5
−1.0
exon 1 2 3 4 5 6 7 8 9 10
intron 1 2 3 4 5 6 7 8 9
633000
633500
634000
634500
635000
bp

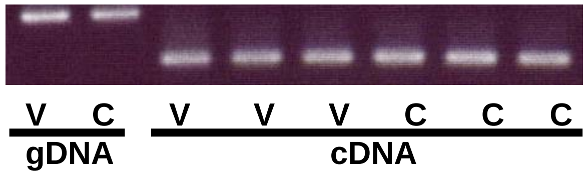
V C V V V C C C
gDNA
cDNA

√

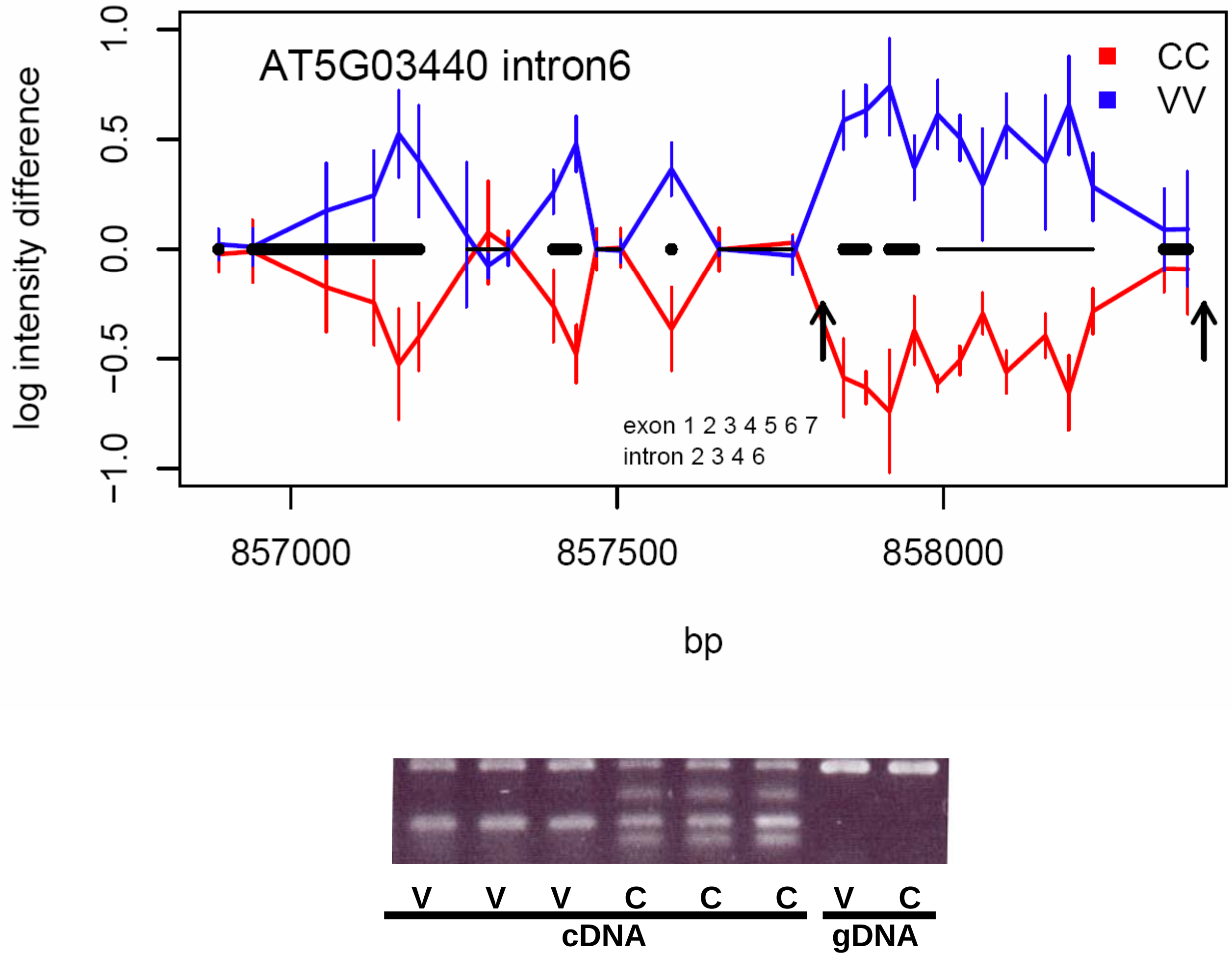

AT5G03440 intron6
CC
VV
log intensity difference
1.0
0.5
0.0
-0.5
-1.0
exon 1 2 3 4 5 6 7
intron 2 3 4 6
857000
857500
858000
bp
V V V C C C V C
cDNA
gDNA

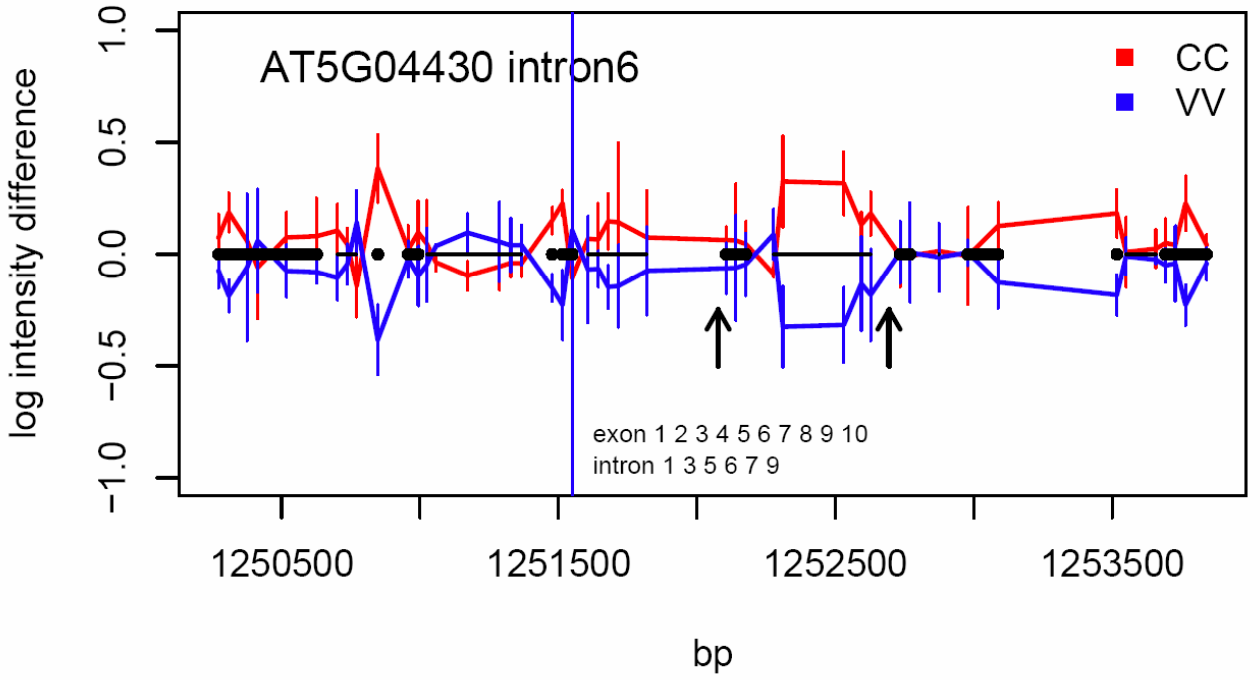
AT5G04430 intron6
CC
VV
log intensity difference
1.0
0.5
0.0
−0.5
−1.0
exon 1 2 3 4 5 6 7 8 9 10
intron 1 3 5 6 7 9
1250500
1251500
1252500
1253500
bp

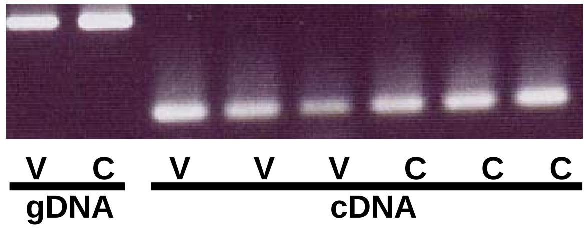
V C V V V C C C
gDNA
cDNA

√

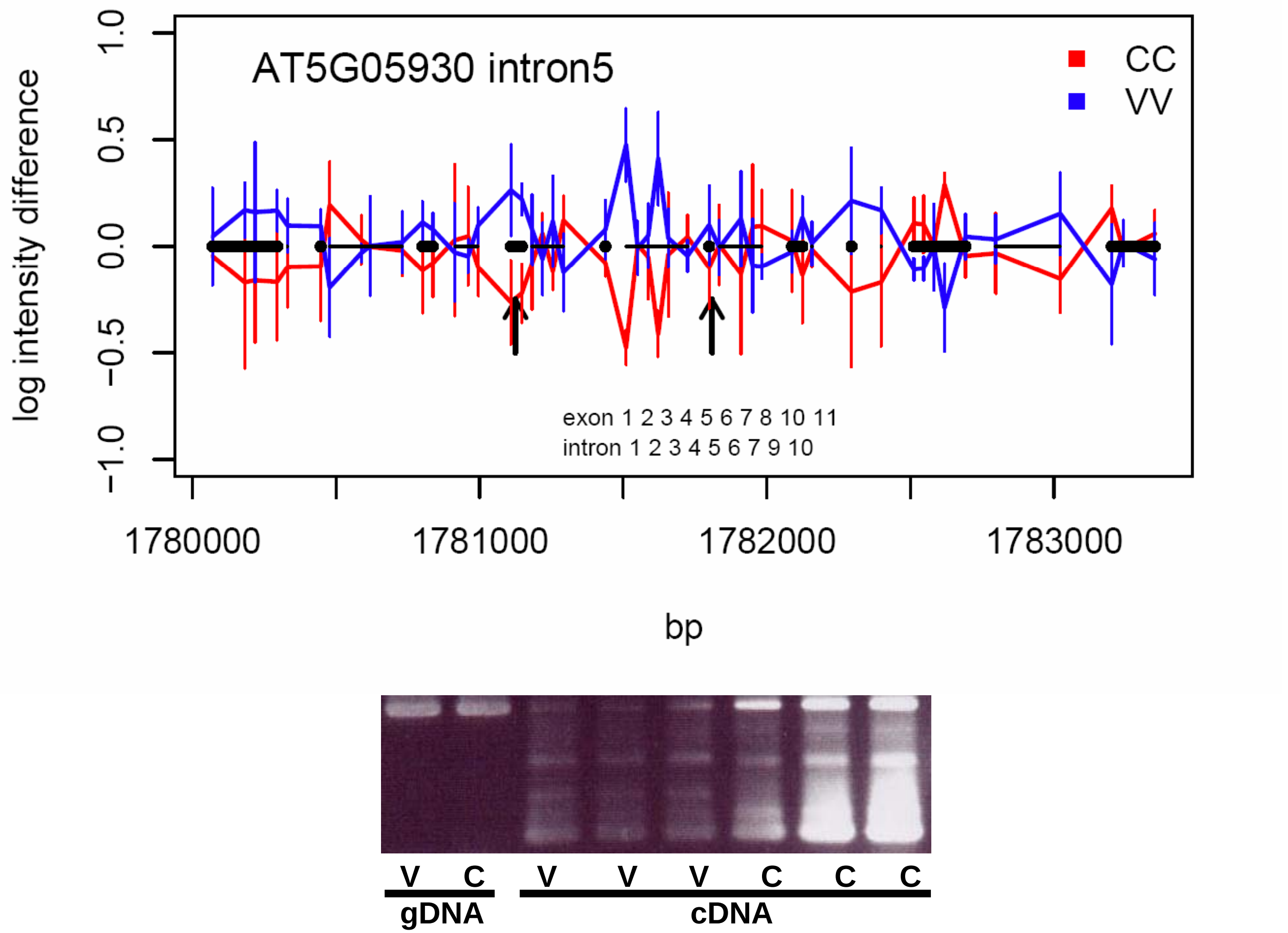
AT5G05930 intron5
CC
VV
log intensity difference
1.0
0.5
0.0
−0.5
−1.0
exon 1 2 3 4 5 6 7 8 10 11
intron 1 2 3 4 5 6 7 9 10
1780000
1781000
1782000
1783000
bp
V C V V V C C C
gDNA
cDNA

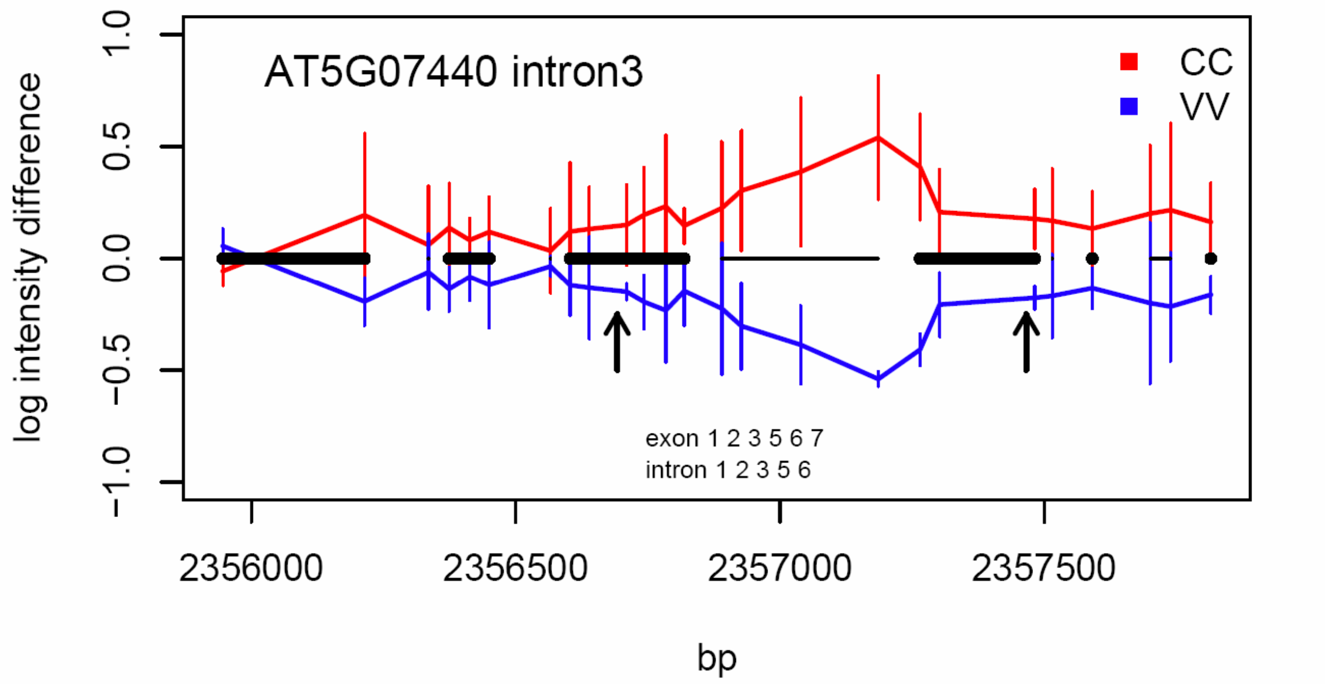
AT5G07440 intron3
CC
VV
log intensity difference
1.0
0.5
0.0
−0.5
−1.0
exon 1 2 3 5 6 7
intron 1 2 3 5 6
2356000
2356500
2357000
2357500
bp

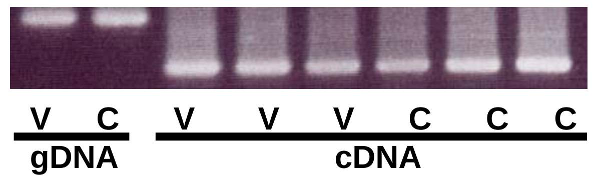
V C V V V C C C
gDNA
cDNA

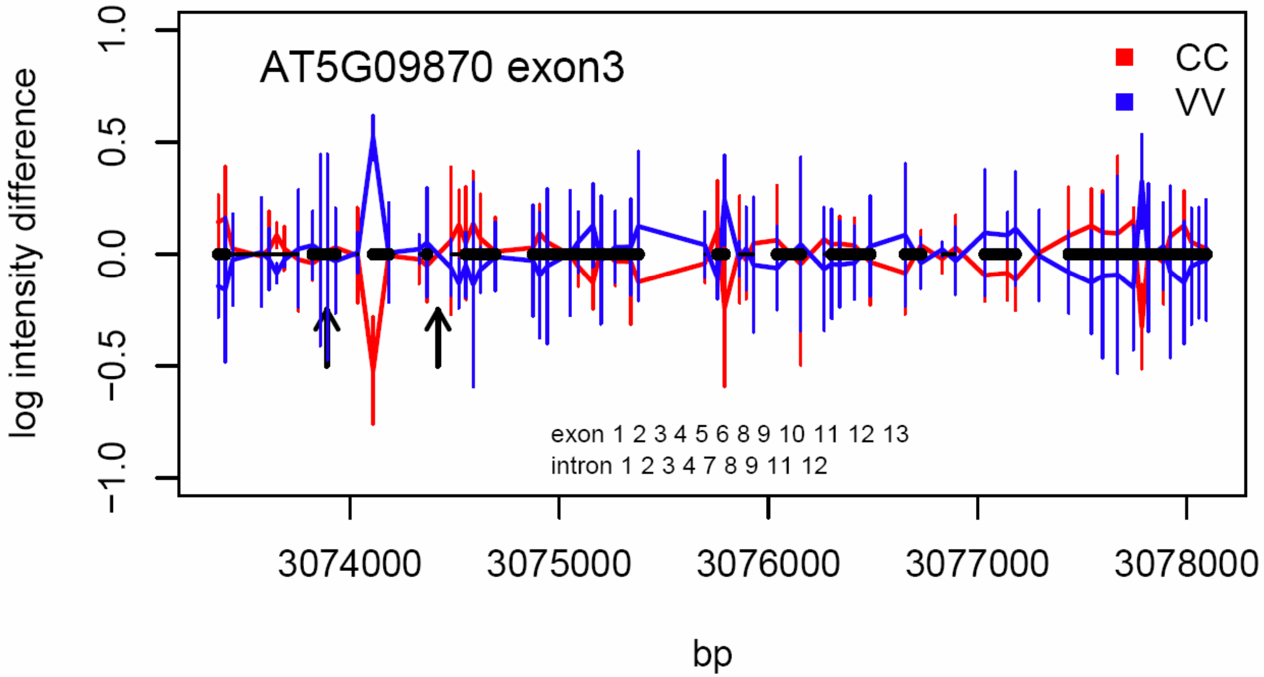

AT5G09870 exon3
CC
VV
log intensity difference
1.0
0.5
0.0
−0.5
−1.0
exon 1 2 3 4 5 6 8 9 10 11 12 13
intron 1 2 3 4 7 8 9 11 12
3074000
3075000
3076000
3077000
3078000
bp

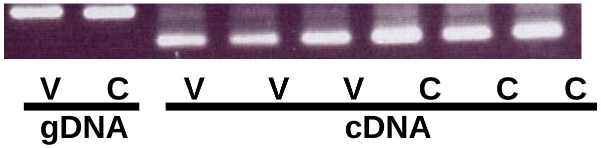

V C V V V C C C
gDNA
cDNA

Not included for analysis

√

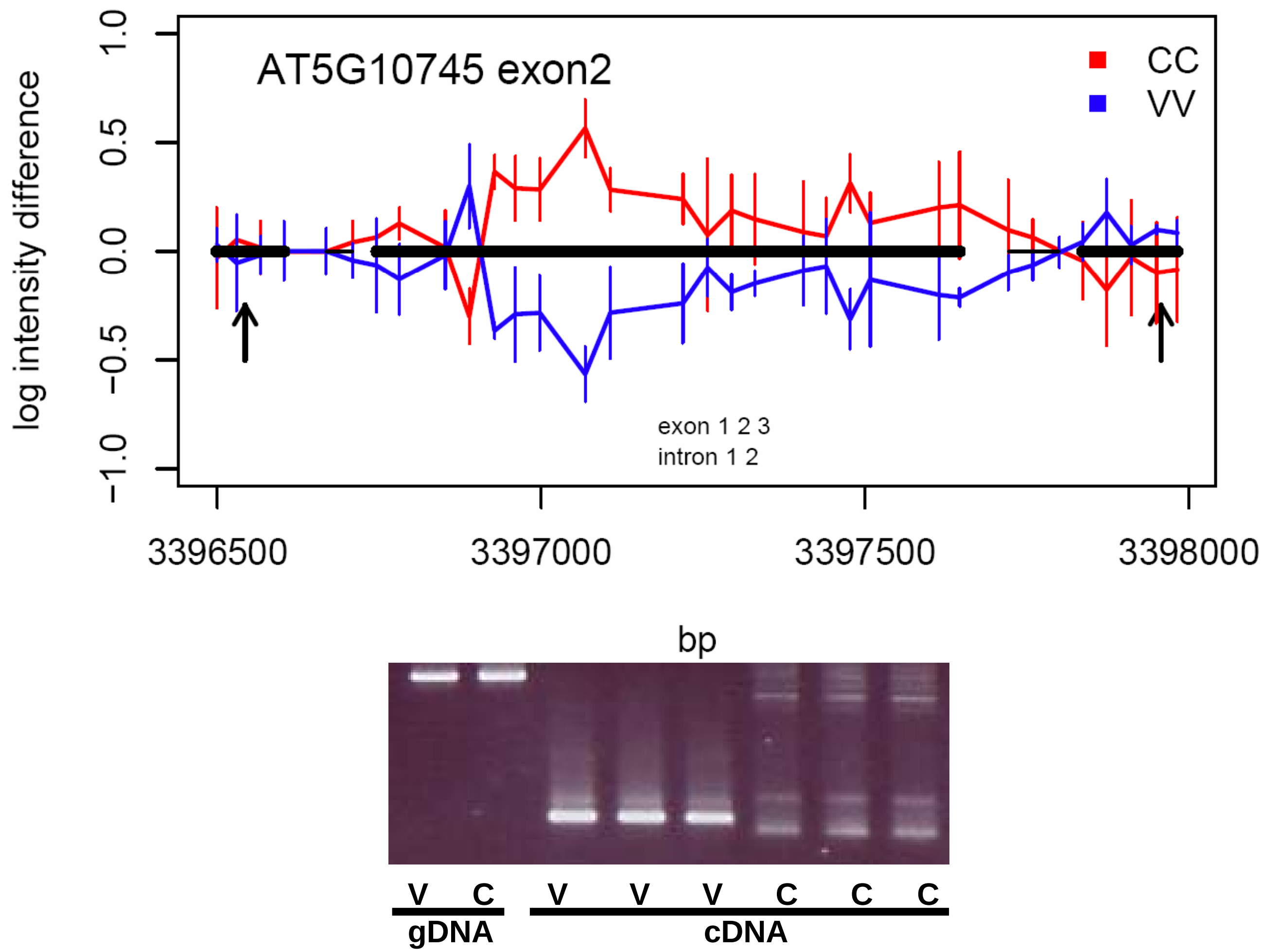

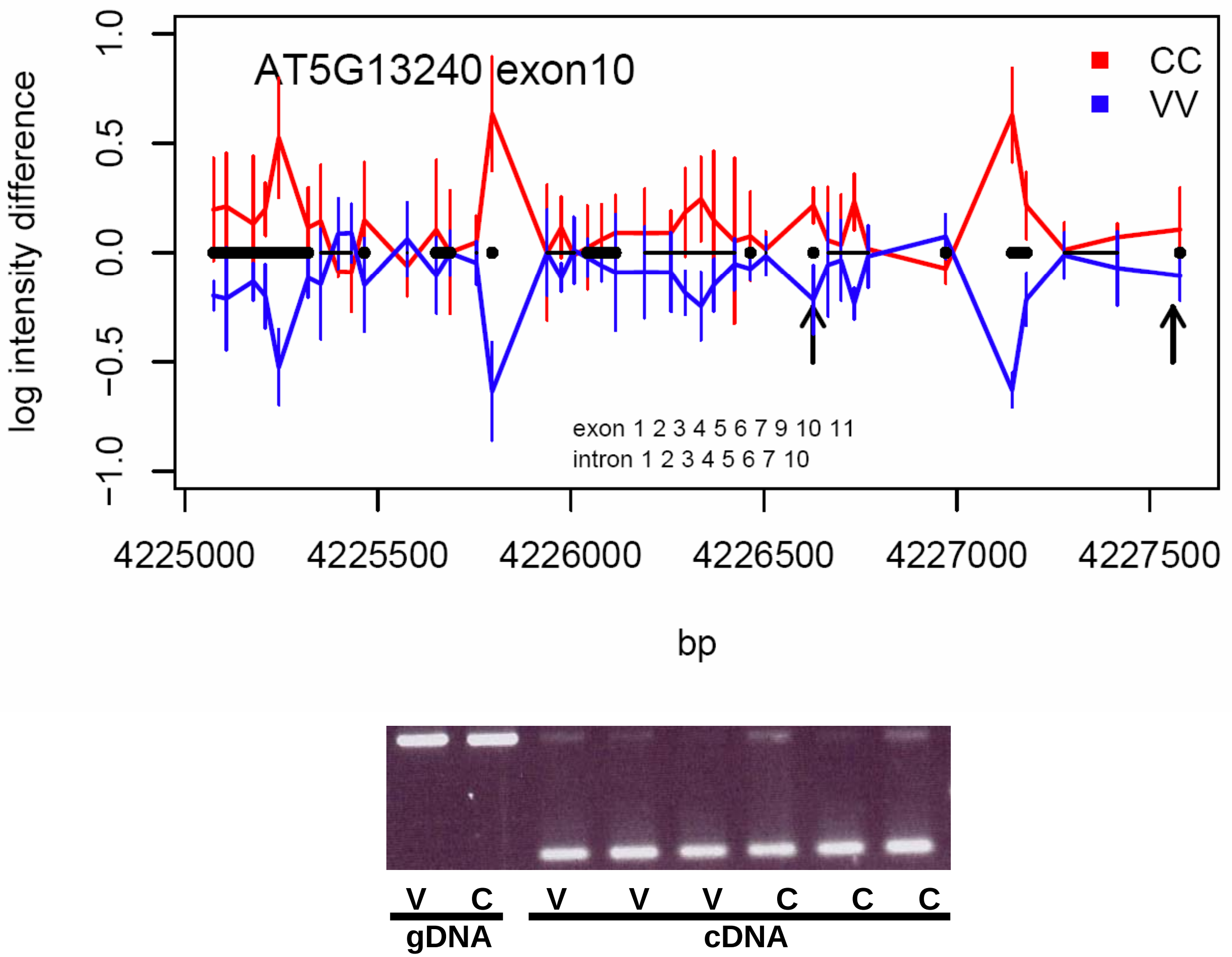
AT5G13240 exon10
CC
VV
log intensity difference
1.0
0.5
0.0
−0.5
−1.0
exon 1 2 3 4 5 6 7 9 10 11
intron 1 2 3 4 5 6 7 10
4225000
4225500
4226000
4226500
4227000
4227500
bp
V C V V V C C C
gDNA
cDNA

√

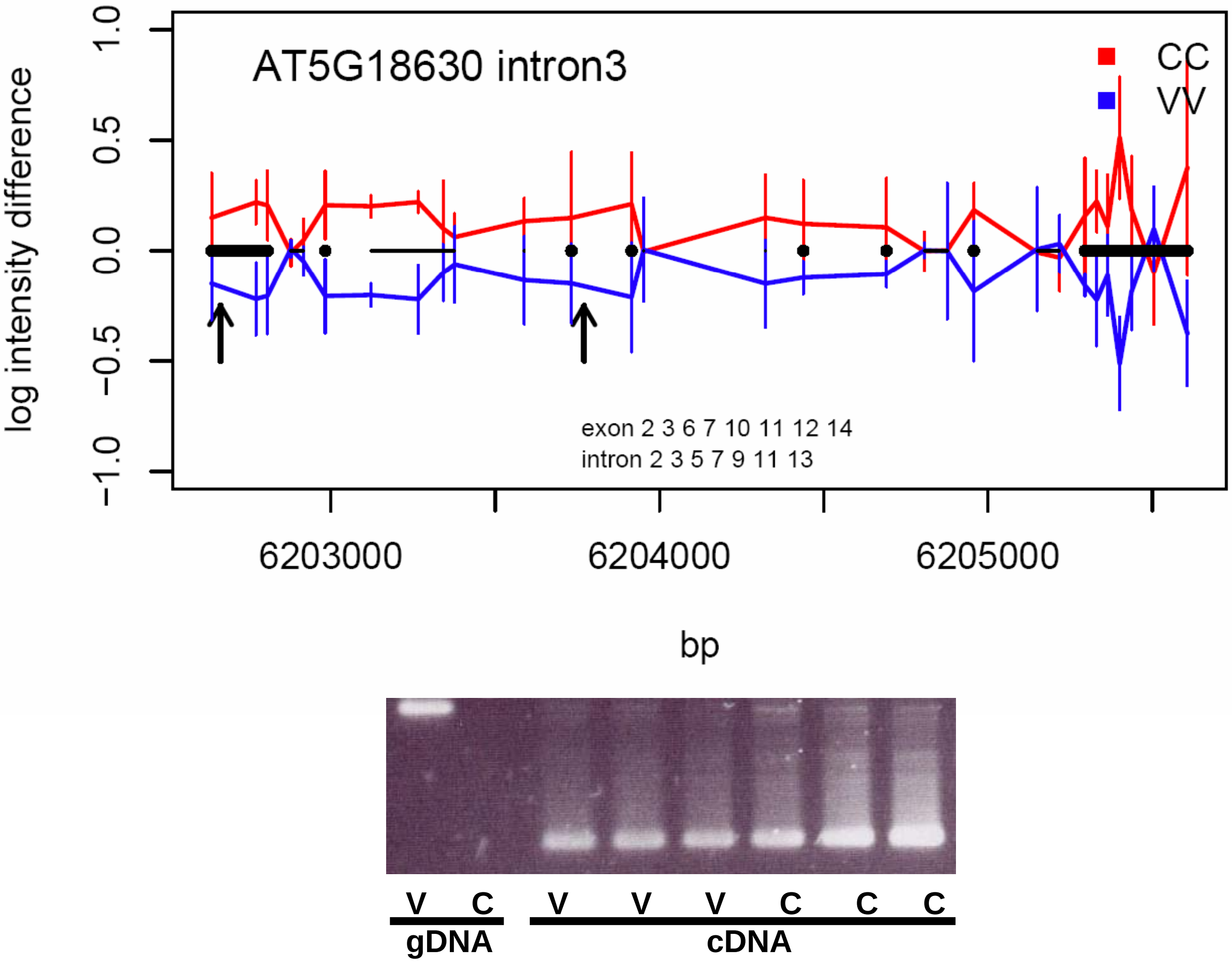
AT5G18630 intron3
CC
VV
log intensity difference
1.0
0.5
0.0
−0.5
−1.0
exon 2 3 6 7 10 11 12 14
intron 2 3 5 7 9 11 13
6203000
6204000
6205000
bp
V C V V V C C C
gDNA
cDNA

√

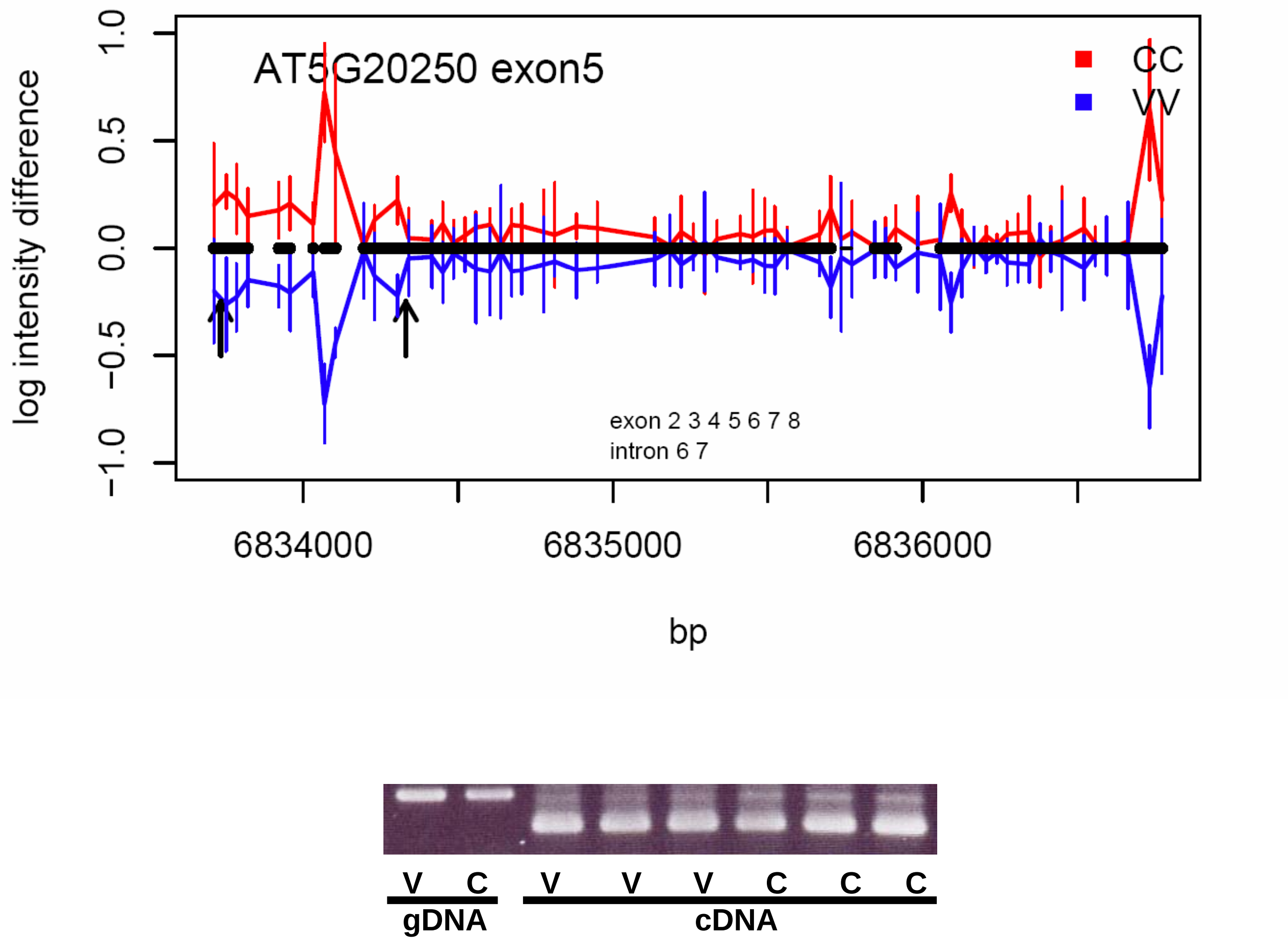

AT5G20250 exon5
CC
VV
log intensity difference
1.0
0.5
0.0
−0.5
−1.0
exon 2 3 4 5 6 7 8
intron 6 7
6834000
6835000
6836000
bp
V C V V V C C C
gDNA
cDNA

Not included for analysis

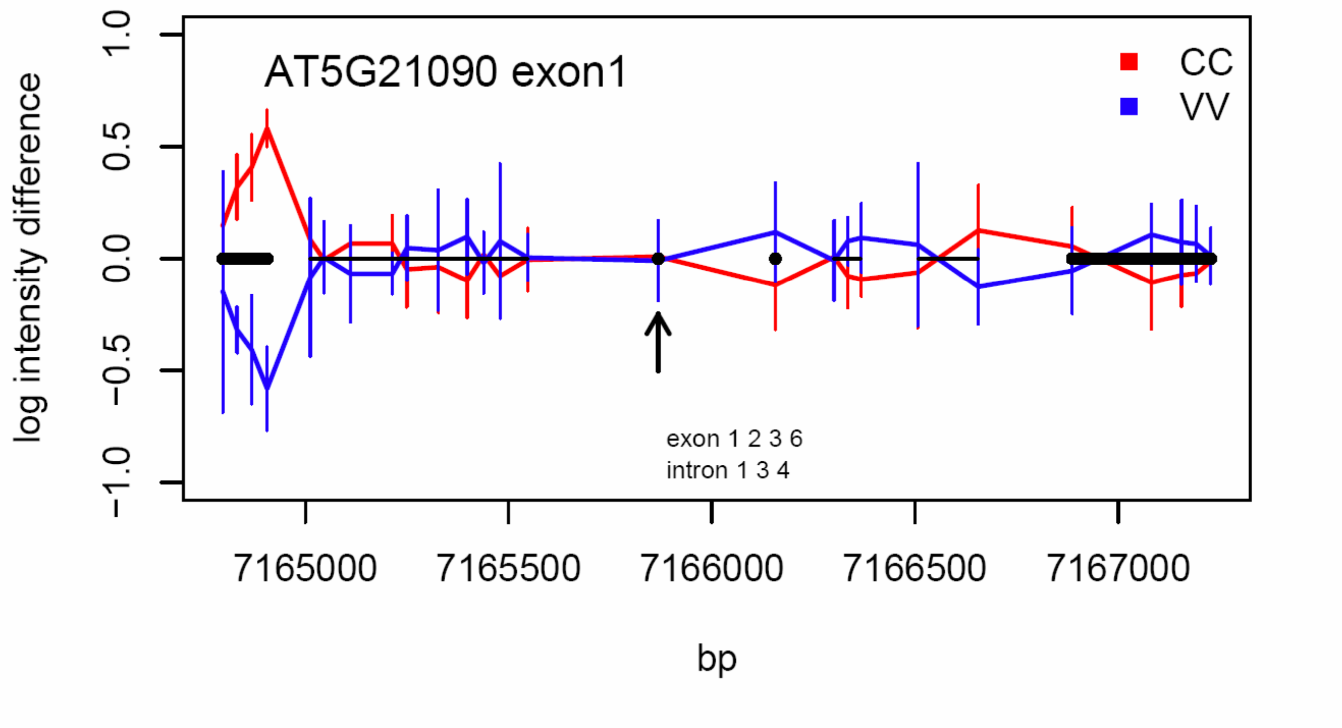

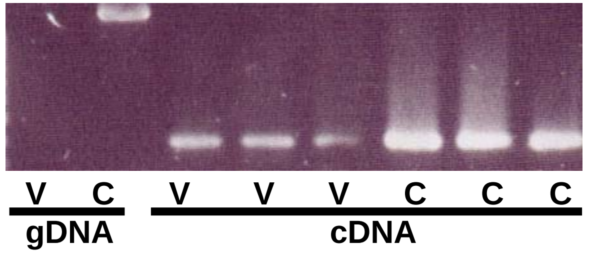

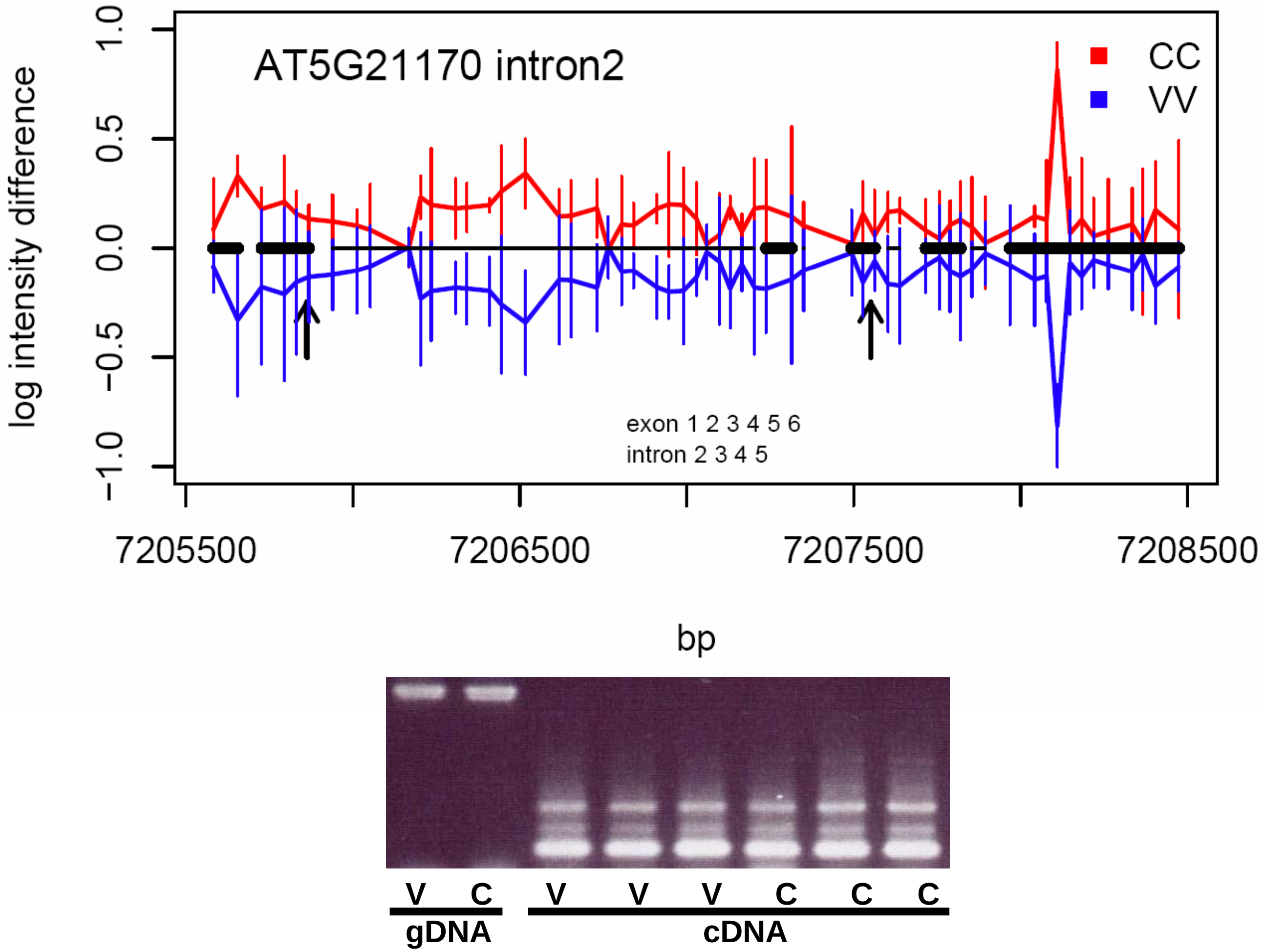
AT5G21170 intron2
CC
VV
log intensity difference
1.0
0.5
0.0
−0.5
−1.0
exon 1 2 3 4 5 6
intron 2 3 4 5
7205500
7206500
7207500
7208500
bp
V C V V V C C C
gDNA
cDNA

√

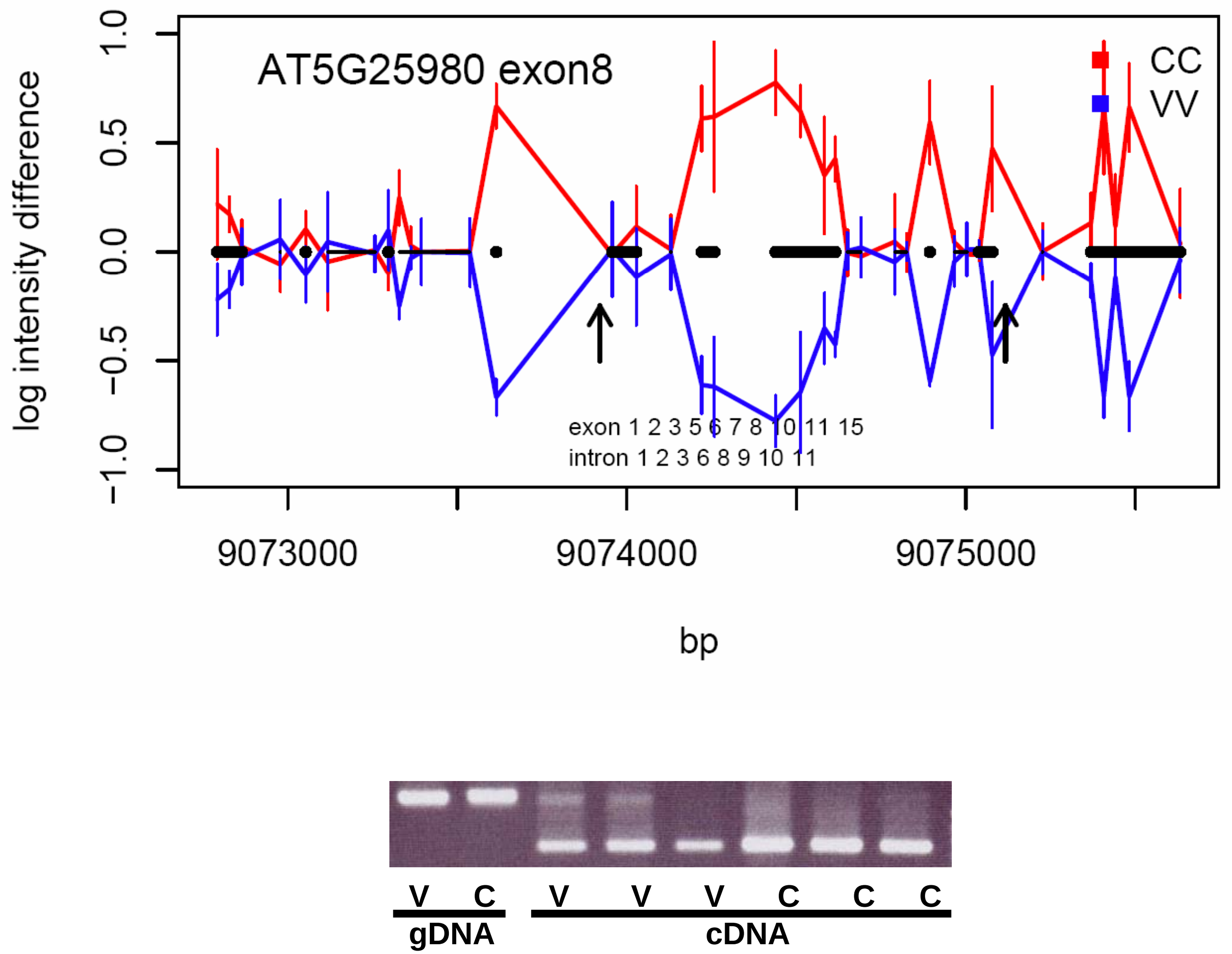
AT5G25980 exon8
CC
VV
log intensity difference
1.0
0.5
0.0
−0.5
−1.0
exon 1 2 3 5 6 7 8 10 11 15
intron 1 2 3 6 8 9 10 11
9073000
9074000
9075000
bp
V C V V V C C C
gDNA
cDNA

√

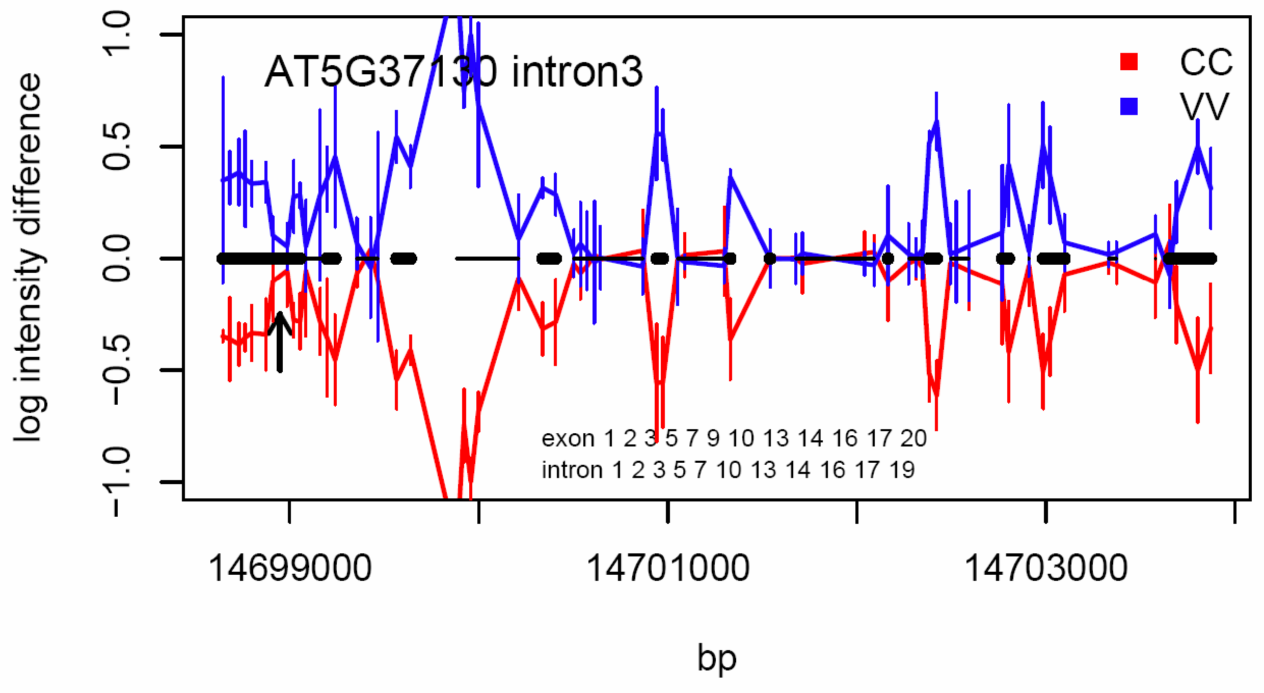
AT5G37130 intron3
CC
VV
log intensity difference
1.0
0.5
0.0
−0.5
−1.0
exon 1 2 3 5 7 9 10 13 14 16 17 20
intron 1 2 3 5 7 10 13 14 16 17 19
14699000
14701000
14703000
bp

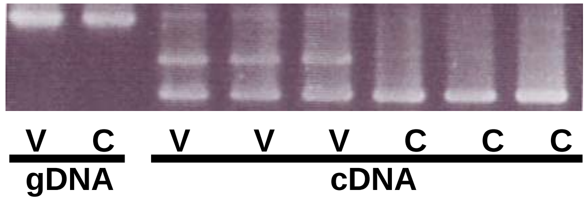
V C V V V C C C
gDNA
cDNA

Not included for analysis

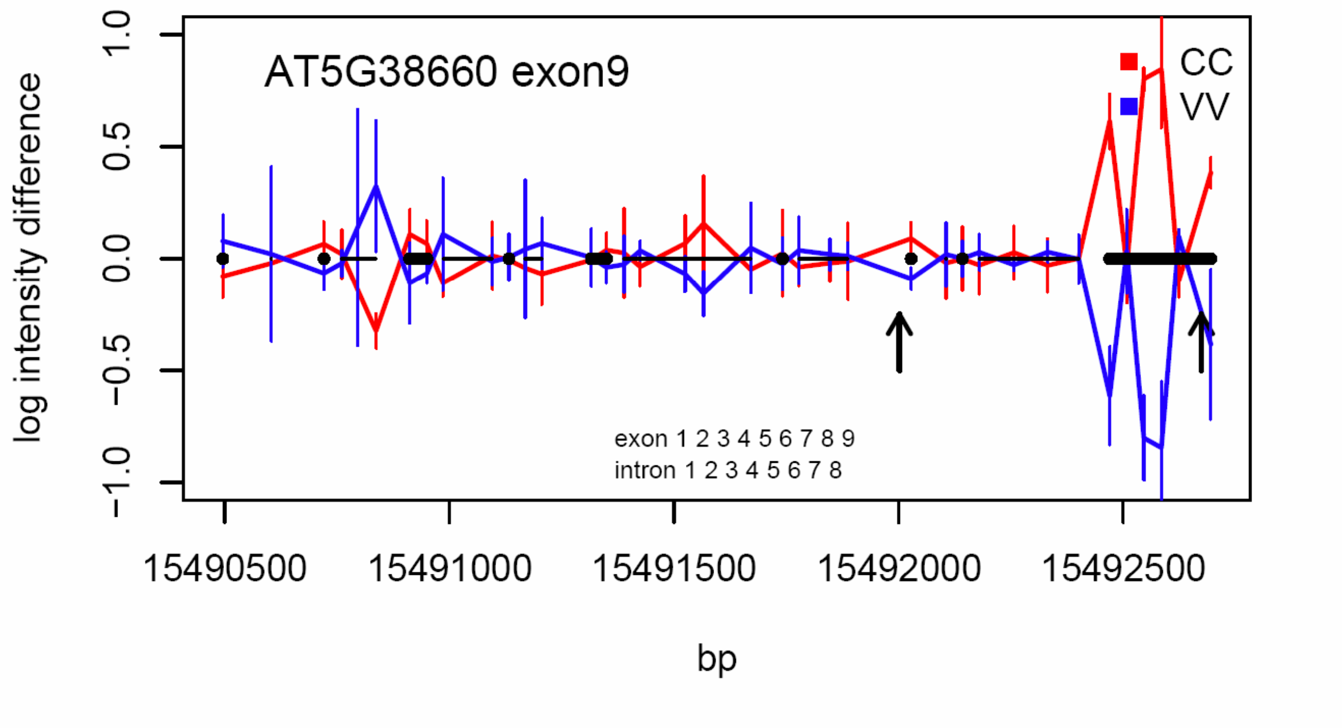

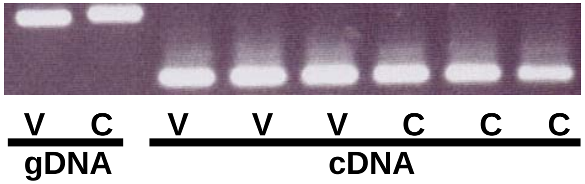

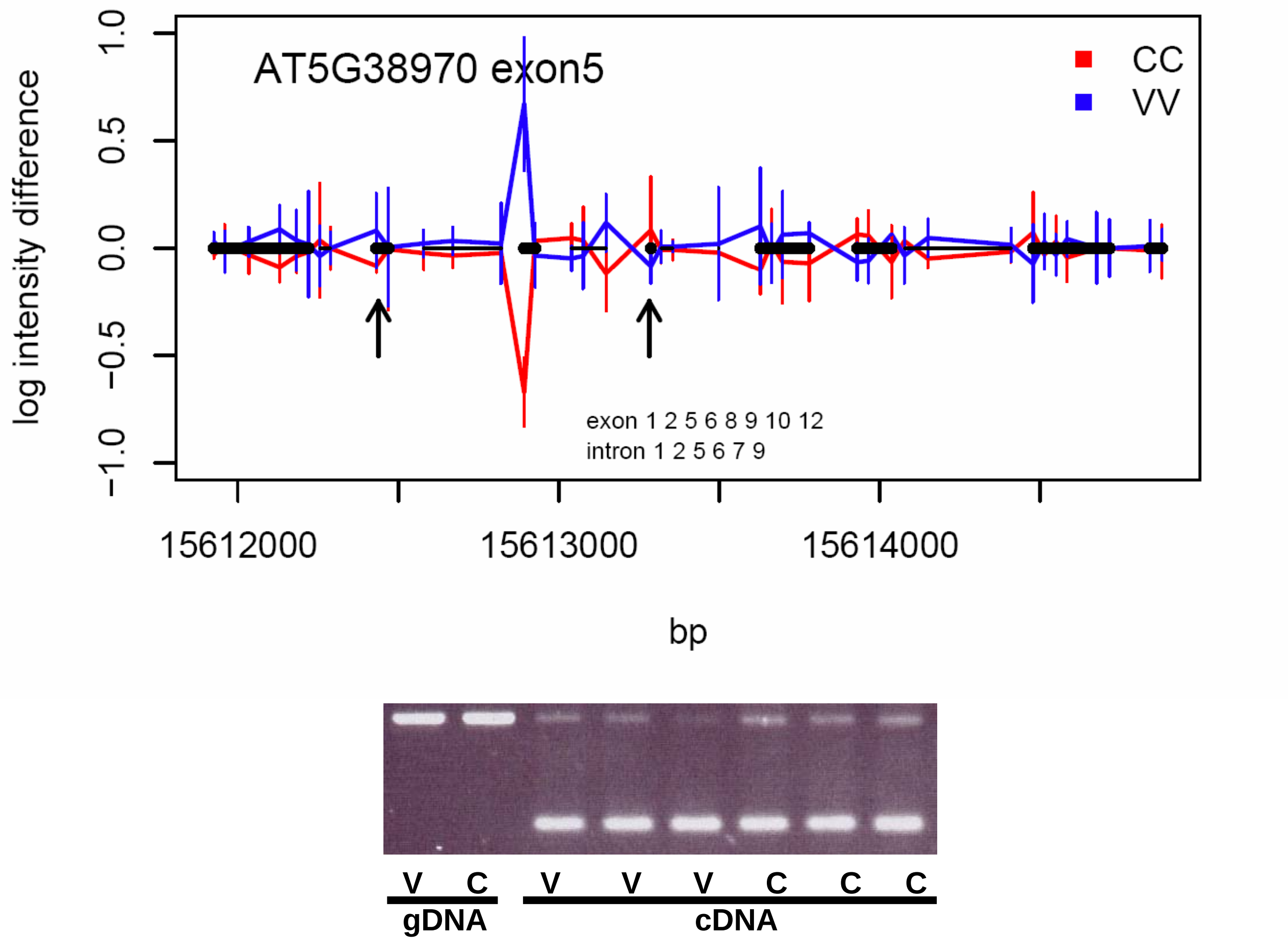
AT5G38970 exon5
CC
VV
log intensity difference
1.0
0.5
0.0
−0.5
−1.0
exon 1 2 5 6 8 9 10 12
intron 1 2 5 6 7 9
15612000
15613000
15614000
bp
V
C
V
V
V
C
C
C
gDNA
cDNA

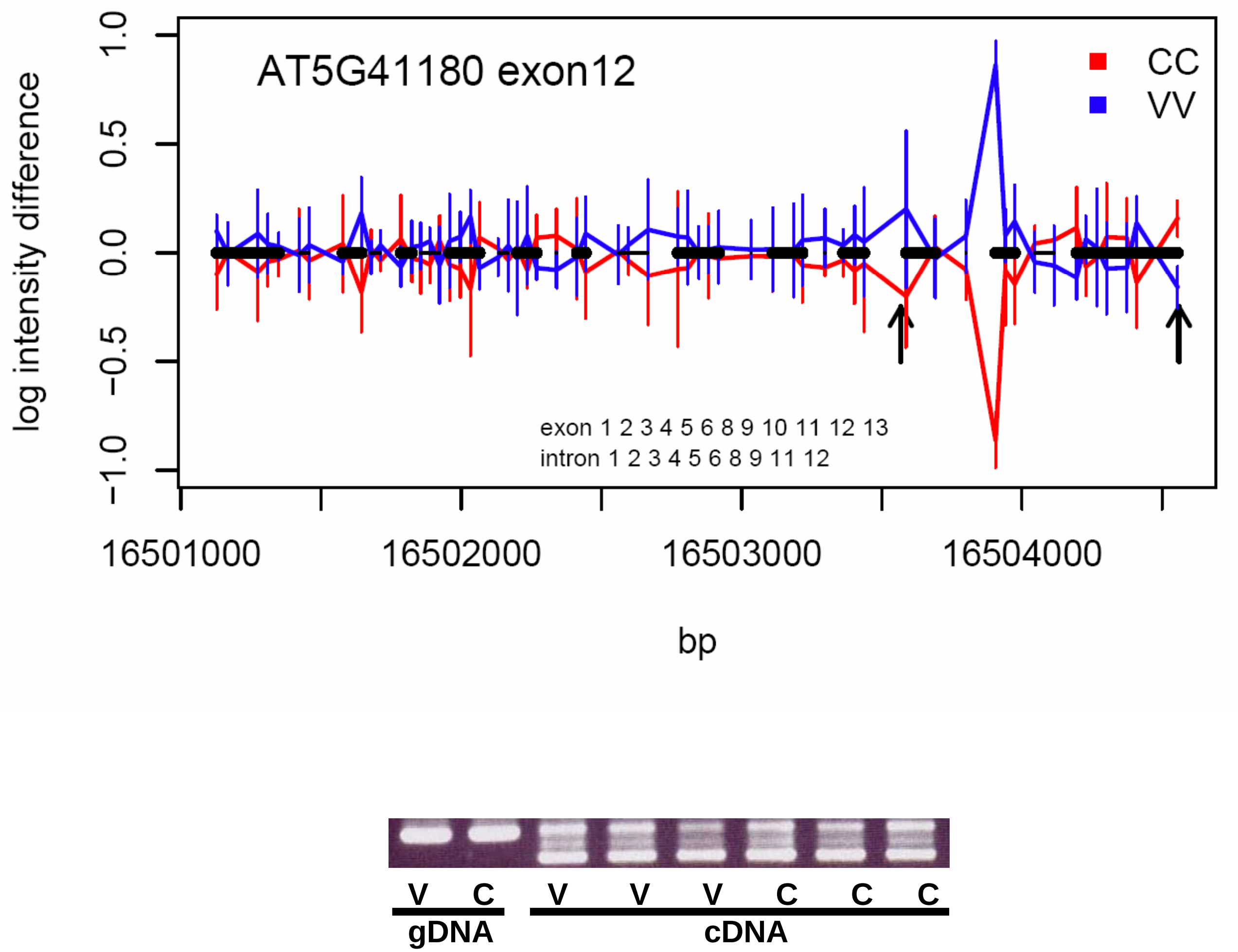
AT5G41180 exon12
CC
VV
log intensity difference
1.0
0.5
0.0
−0.5
−1.0
exon 1 2 3 4 5 6 8 9 10 11 12 13
intron 1 2 3 4 5 6 8 9 11 12
16501000
16502000
16503000
16504000
bp
V C V V V C C C
gDNA
cDNA

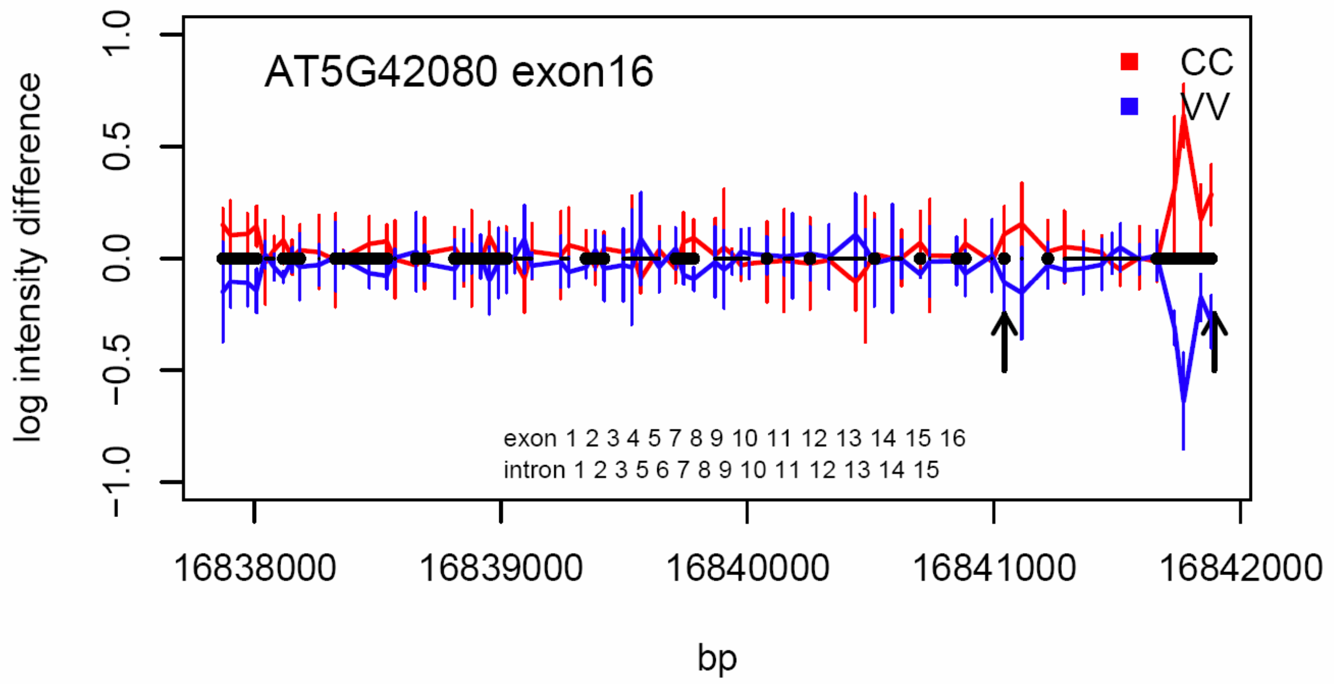

AT5G42080 exon16
CC
VV
log intensity difference
1.0
0.5
0.0
−0.5
−1.0
exon 1 2 3 4 5 7 8 9 10 11 12 13 14 15 16
intron 1 2 3 5 6 7 8 9 10 11 12 13 14 15
16838000
16839000
16840000
16841000
16842000
bp

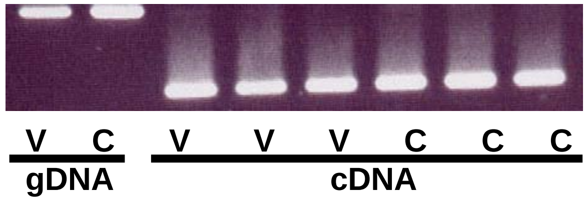

V C V V V C C C
gDNA
cDNA

√

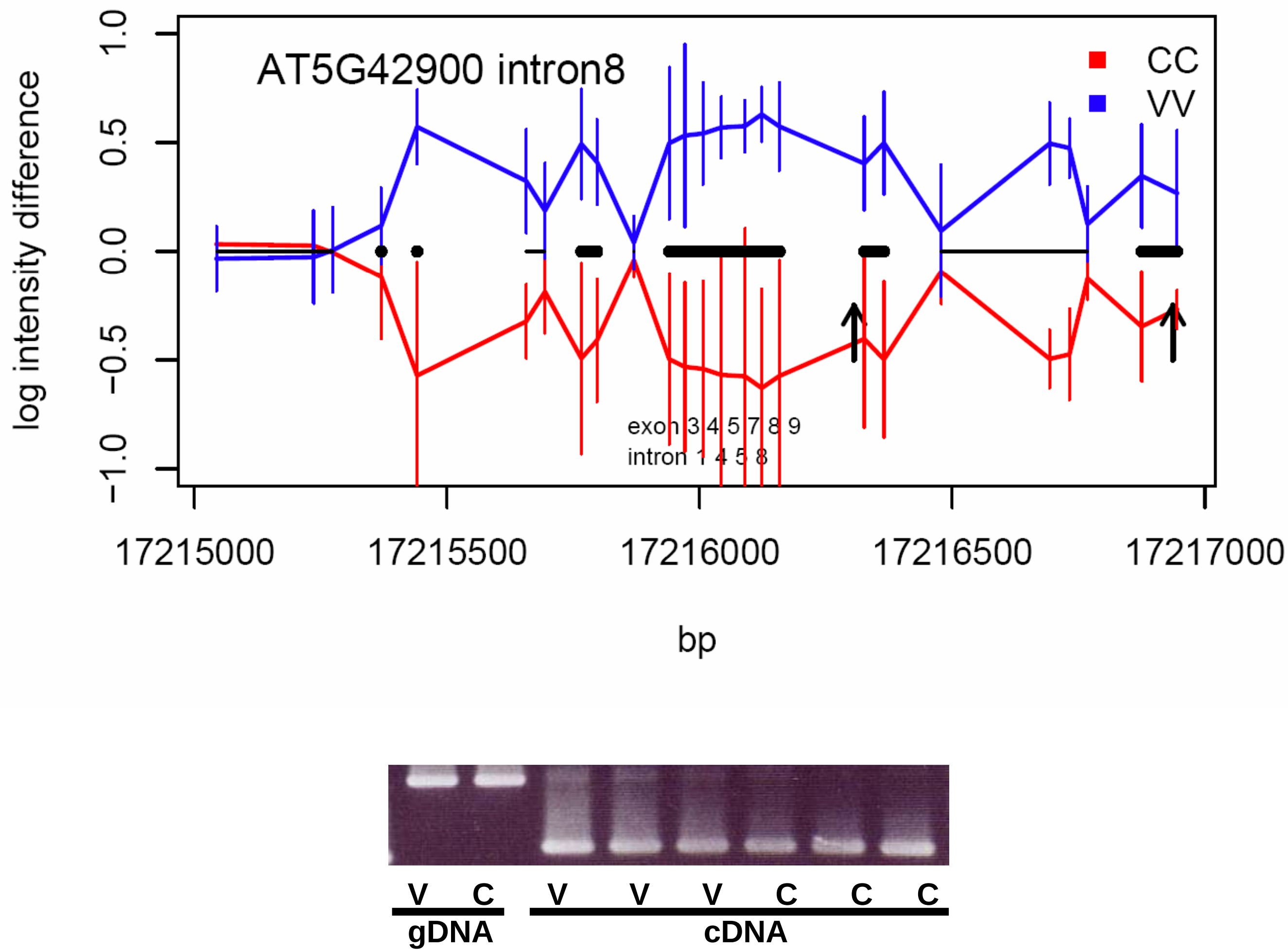
AT5G42900 intron8
CC
VV
log intensity difference
1.0
0.5
0.0
−0.5
−1.0
exon 3 4 5 7 8 9
intron 1 4 5 8
17215000
17215500
17216000
17216500
17217000
bp
V C V V V C C C
gDNA
cDNA

√

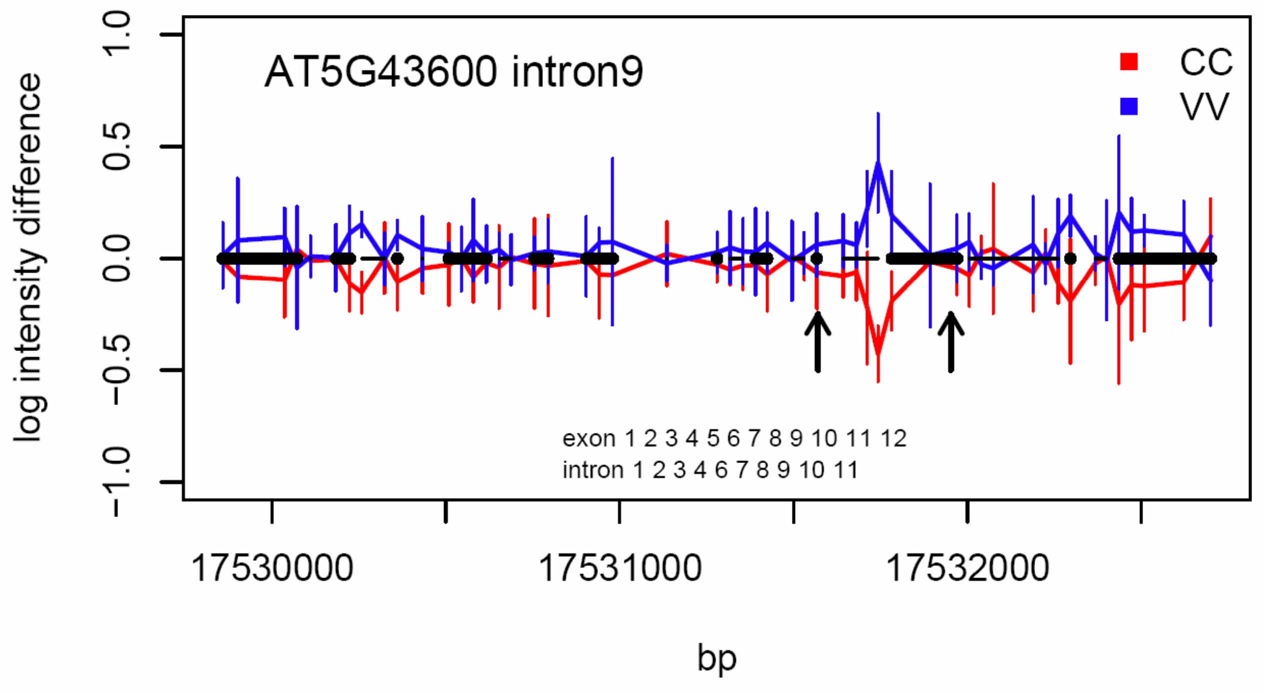
AT5G43600 intron9
CC
VV
log intensity difference
1.0
0.5
0.0
−0.5
−1.0
exon 1 2 3 4 5 6 7 8 9 10 11 12
intron 1 2 3 4 6 7 8 9 10 11
17530000
17531000
17532000
bp

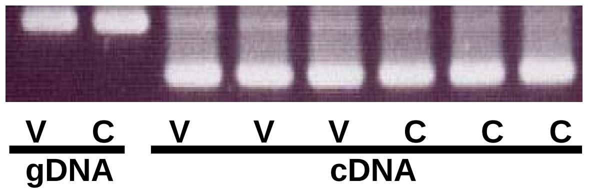
V C V V V C C C
gDNA
cDNA

Not included for analysis

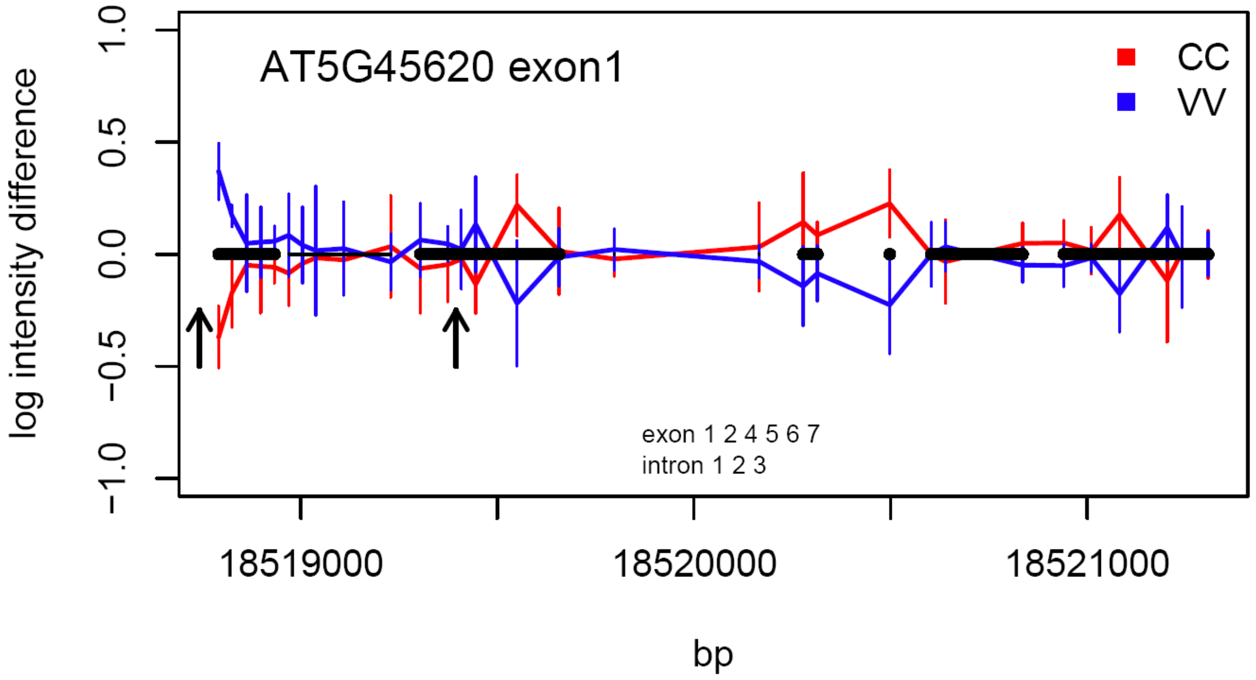

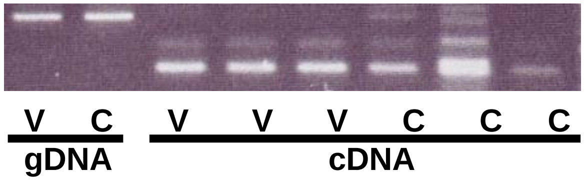

Not included for analysis

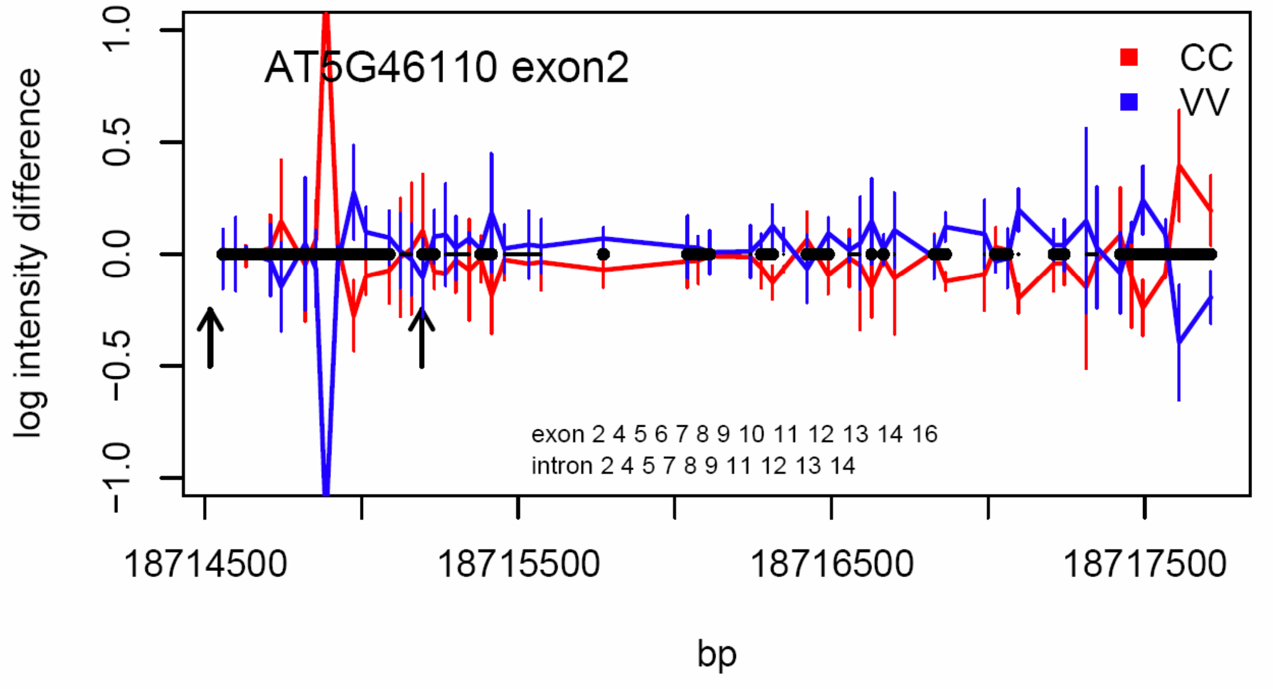

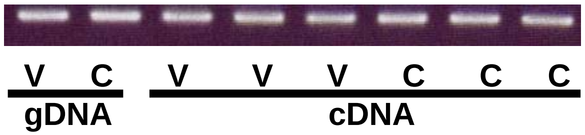

Not included for analysis

√

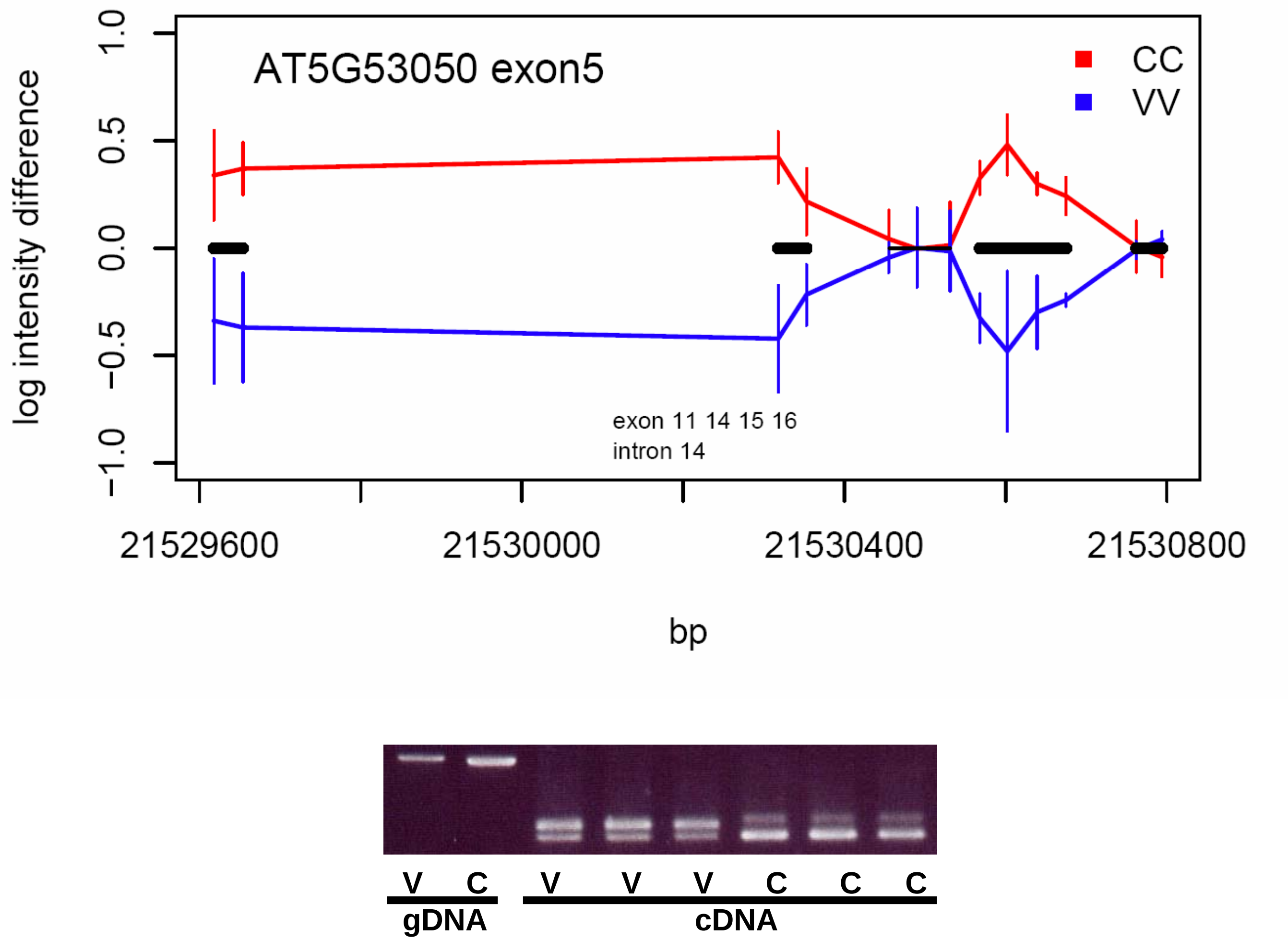

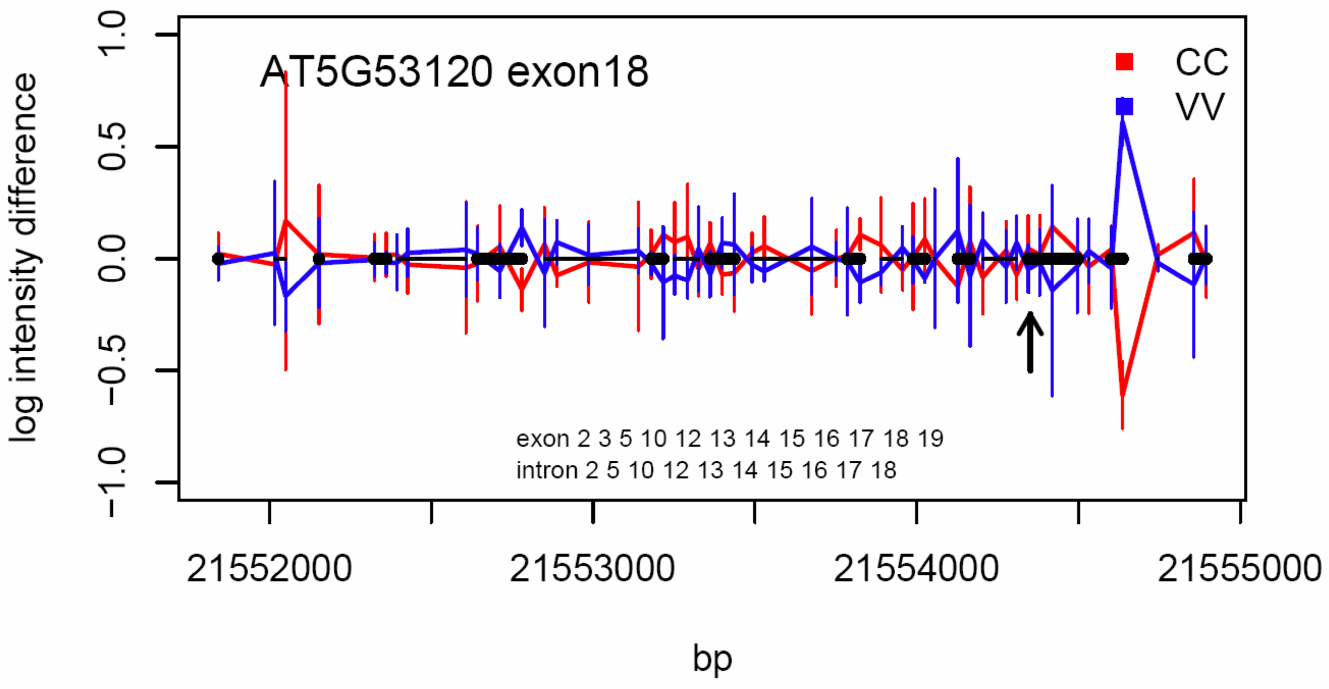
AT5G53120 exon18
CC
VV
log intensity difference
1.0
0.5
0.0
−0.5
−1.0
exon 2 3 5 10 12 13 14 15 16 17 18 19
intron 2 5 10 12 13 14 15 16 17 18
21552000
21553000
21554000
21555000
bp

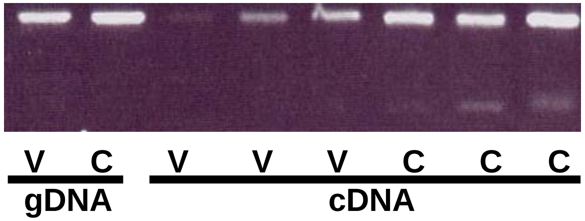
V C V V V C C C
gDNA
cDNA
